# Palladium-Catalyzed Allylation/Benzylation of *H*-Phosphinate Esters with Alcohols

**DOI:** 10.3390/molecules21101295

**Published:** 2016-09-28

**Authors:** Anthony Fers-Lidou, Olivier Berger, Jean-Luc Montchamp

**Affiliations:** Department of Chemistry and Biochemistry, TCU Box 298860, Texas Christian University, Fort Worth, TX 76129, USA; a.fers-lidou@outlook.fr (A.F.-L.); o.berger@tcu.edu (O.B.)

**Keywords:** palladium, cross-coupling, alcohols, *H*-phosphinate esters, allylation, benzylation

## Abstract

The Pd-catalyzed direct alkylation of *H*-phosphinic acids and hypophosphorous acid with allylic/benzylic alcohols has been described previously. Here, the extension of this methodology to *H*-phosphinate esters is presented. The new reaction appears general, although its scope is narrower than with the acids, and its mechanism is likely different. Various alcohols are examined in their reaction with phosphinylidene compounds R^1^R^2^P(O)H.

## 1. Introduction

**Scheme 1:**
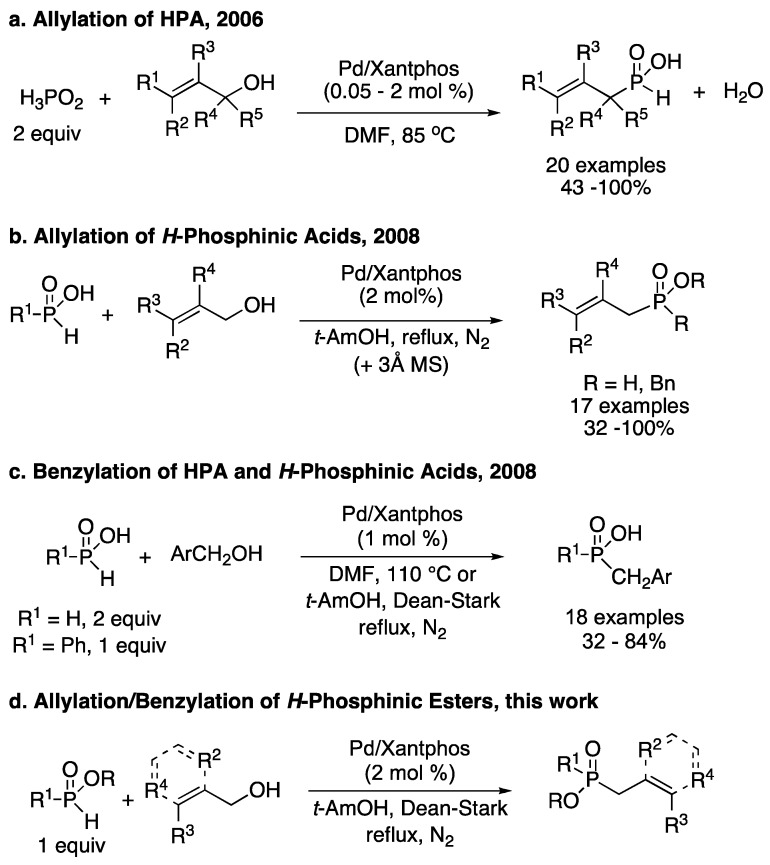
Carbon-phosphorus bond formation by allylation/benzylation with alcohols. Xantphos, 4,5-bis(diphenylphosphino)-9,9-dimethylxanthene; MS, molecular sieves; *t*-AmOH, *t*-amyl alcohol.

In the classic Pd-catalyzed Tsuji-Trost reaction, the allylic electrophile is an alcohol derivative (most often the acetate) and numerous nucleophiles can be employed easily [1]. More recently, the use of allylic alcohols has emerged [1,2]. Using alcohols for the direct allylation of nucleophiles is desirable because water is the only byproduct. In the context of carbon-phosphorus bond-formation, we disclosed 10 years ago the reaction between hypophosphorous acid (HPA) and allylic alcohols catalyzed by Pd to directly afford the corresponding allylic *H*-phosphinic acids (Scheme 1a) [3]. The reaction was subsequently extended to include *H*-phosphinic acids instead of HPA to form disubstituted phosphinic acids under slightly more forcing conditions (Scheme 1b) [4]. Around the same time, we also described the benzylation of HPA and *H*-phosphinic acids with benzylic alcohols under similar conditions (Scheme 1c) [5]. These reactions were discovered based on mechanistic reasoning that was supported by model studies [6]. It was thought that the reaction required a PO_2_H motif for Fisher esterification and tautomerization. More recently, we decided to reexamine this type of reaction, but using *H*-phosphinic esters as starting materials, and were surprised to observe a successful allylation, suggesting that a different mechanism could be operative. This manuscript describes these findings (Scheme 1d).

## 2. Results and Discussion

Based on our prior work in this area, we selected cinnamyl alcohol and butyl phenyl-*H*-phosphinate in equimolar amounts as the reacting partners for the initial investigation (Table 1). Cinnamyl alcohol was identified previously as a very reactive partner in our Pd-catalyzed allylation [3,4,5,6]. Additionally, based on our prior findings, *t*-amyl alcohol was selected as the solvent for the azeotropic removal of the water byproduct (reflux, Dean-Stark trap) [4,5]. Using palladium acetate as the catalyst, and without added ligand, the reaction failed to produce any detectable amount of product (entry 1). As we had found in our other couplings, Xantphos (4,5-bis(diphenylphosphino)-9,9-dimethylxanthene) performed superbly affording the desired product in nearly quantitative isolated yield (entry 2). Switching the solvent to toluene (still with a Dean-Stark trap) gave a satisfactory yield, albeit lower (entry 3). This was not entirely unexpected as *t*-amyl alcohol was initially identified for its ability to promote P(V) to P(III) tautomerization through hydrogen-bonding. Changing the salt from palladium acetate to palladium chloride resulted in a slightly lower yield (entry 4 vs. 2). On the other hand, tris(dibenzylideneacetone)dipalladium(0) (Pd_2_(dba)_3_) (as we had done in most of our prior work on allylation/benzylation) also gave a nearly quantitative yield of product (entry 5). Using 1,1’-bis(diphenylphosphino)ferrocene (Dppf) as the ligand did afford the desired product, but in significantly lower yield (entry 6). Thus, the results shown in Table 1 confirmed the catalyst system we had identified earlier for the cross-coupling of phosphinic acids.

Next, the scope of the allylation with cinnamyl alcohol was investigated with a variety of phosphinylidene compounds (Table 2). Not surprisingly, changing the ester group from *n*-butyl to cyclohexyl gave a good result (entry 2), while a benzyl ester gave a lower yield (entry 3), presumably because of competing transesterification. The less reactive cyclohexyl-octyl-*H*-phosphinate [7] reacted uneventfully (entry 4), while butyl cinnamyl-*H*-phosphinate afforded a quantitative yield of butyl bis(cinnamyl)phosphinate (entry 5). Other functionalized *H*-phosphinate esters were tested (entries 6–9) giving generally good results. However, the Ciba-Geigy reagent (entry 10) was unsatisfactory. The acetal moiety is acid sensitive and this result may point out to the presence of acidic species along the reaction coordinates. The rather special *H*-phosphinate DOPO (6*H*-dibenzo[*c*,*e*][1,2λ^5^]oxaphosphinine 6-oxide) [7] gave an excellent yield of product (entry 11). Other types of phosphinylidene were tested: diethyl *H*-phosphonate and diphenyl phosphine oxide both afforded the desired products in excellent yields (entries 12–13).

Whereas the cinnamyl moiety is a versatile functional group and its introduction appears quite general (Table 2), we next examined other allylic alcohols as well as benzylic alcohols. The results are gathered in Table 3.

Reactions with the simple allyl alcohol (2 equiv.) gave excellent results (>90% isolated yield) as shown in entries 1–3. The 2-methyl substituted version of cinnamyl alcohol, 2-methyl-3-phenyl-2-propen-1-ol (1 equiv.) also gave satisfactory results (entries 4–5). Methallyl alcohol, on the other hand, gave only a moderate yield of product (entry 6), but this result was not unexpected, as this alcohol also had given poor results with HPA [3]. Myrtenol also reacted successfully (entry 7). Next, the benzylation was investigated (entries 8–11) and products were obtained in moderate to good yields. Unfortunately, some other combinations of reactants did not afford the desired product in acceptable yields (for example: entries 12–14), thereby showing some limitations in scope. Electron-rich furfuryl alcohol (entry 12) may lead to slow oxidative-addition and an overall inefficient transformation. Acid sensitivity may become an issue: while this alcohol was successful with hypophosphorous acid [5], the more difficult the desired reaction, the more side reactions will be competing. Perhaps for a similar reason, none of the secondary allylic alcohols we tried reacted successfully. In entry 13, transesterification of the benzyl ester is a greater problem than it was in entry 8 because the reaction is slower than with cinnamyl alcohol (Table 2, entry 4). Similarly, diethyl *H*-phosphonate, which reacted satisfactorily with cinnamyl alcohol (Table 2, entry 12) also gave little product with benzyl alcohol (entry 14). Thus, marginal results are obtained when the reaction is slowed due to any of the following parameters (or combinations): less reactive allylic/benzylic electrophile (aromatics, of course, being much less reactive than alkenes), unfavorable tautomerization profile [7], transesterification of the phosphorus ester, or acid sensitivity of a reactant.

**Scheme 2:**
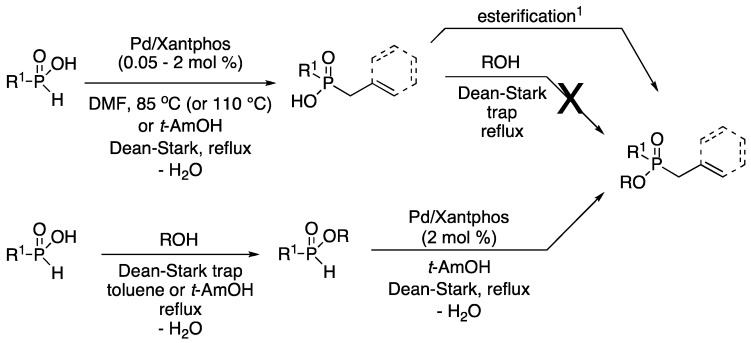
*H*-phosphinic acids versus *H*-phosphinic esters.^1^ SOCl_2_ then ROH; carbodiimide + ROH; RCHN_2_; R^1^COCl then ROH; Ag_2_O then alkyl halide (RX), etc.

Although the scope of this reaction seems more limited than the corresponding reaction of phosphinic acids, it can offer significant synthetic advantages. Since disubstituted phosphinic acids cannot be esterified easily through Fischer-like reactions with azeotropic water-removal [8,9], formation of their esters requires prior activation of the acid (P(O)OH to P(O)LVG + ROH, P(O)OH to P(O)(OAg) + RX, where LVG is a leaving group and RX an alkyl halide) or a diazoalkane. Therefore, the intermediacy of disubstituted phosphinic acids implies atom-wasteful procedures (Scheme 2). On the other hand, *H*-phosphinic acids can be esterified easily with an alcohol and, therefore, the resulting synthetic sequence is more convenient and environmentally friendly (Scheme 2).

**Scheme 3:**
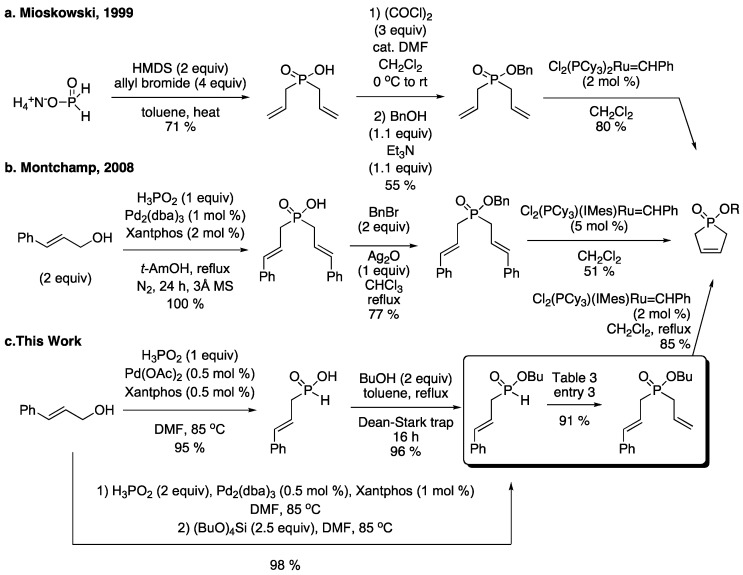
Comparison of methods for the synthesis of 3-phospholenic esters. HMDS: hexamethyldisilazane; IMes: 1,3-bis(2,4,6-trimethylphenyl)imidazol-2-ylidene.

An illustrative example is shown in Scheme 3 for the preparation of 3-phospholenic esters. The “classic” approach is illustrated by Miokowski’s work (Scheme 3a) [10]. The double allylation of bis-(trimethylsiloxy)phosphine afforded the symmetrical phosphinic acid. Subsequent esterification via P(O)Cl then provided the benzyl ester, which was converted to the phospholene ester by ring-closing metathesis (RCM). It should be noted that the attempted RCM reaction of diallylphosphinic acid failed completely. The overall sequence produced the phospholene in 31% isolated yield.

Next, our allylation methodology was used to produce bis(cinnamyl)phosphinic acid in quantitative yield [4]. Silver-promoted esterification gave the corresponding benzyl ester in 77% yield. Ring-closing metathesis afforded the desired heterocycle in 51% isolated yield. The moderate yield of this reaction may be attributed to the fact that both alkenes are substituted. In spite of this, the overall sequence gave the phospholene derivative in 39% yield.

Cinnamyl-*H*-phosphinic acid is easily synthesized, as we reported [11]. Low loading of Pd (0.5 mol %) can be employed to still deliver a very high 95% yield (Scheme 2c) [3]. Esterification with *n*-butanol under Dean-Stark conditions proceeded in 96% yield. The present allylation reaction (Table 3, entry 3) gave the disubstituted ester in 91% yield (Scheme 2c, in box). Subsequent RCM afforded the phospholene in 85% yield. Overall, the sequence in Scheme 3c produced the phospholene derivative in four steps and 70% overall yield, through catalytic reactions and with only water as the byproduct. An alternative using our silicate esterification [9] directly gives butyl cinnamyl-*H*-phosphinate from cinnamyl alcohol in 98% isolated yield [6]. Using this sequence, the phospholenic acid is still produced inexpensively in an outstanding 76% overall yield.

**Scheme 4:**
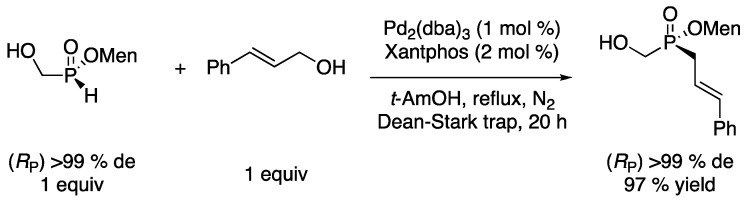
Stereospecific cinnamylation. Men: (−)-menthyl.

In addition to the advantages of Dean-Stark processes over the use of wasteful stoichiometric reagents, the present allylation of *H*-phosphinate esters can offer unique opportunities. For example (Scheme 4), the cinnamylation of optically-active menthyl hydroxymethyl-*H*-phosphinate proceeds with complete stereoselectivity in a nearly quantitative isolated yield [12].

**Scheme 5:**
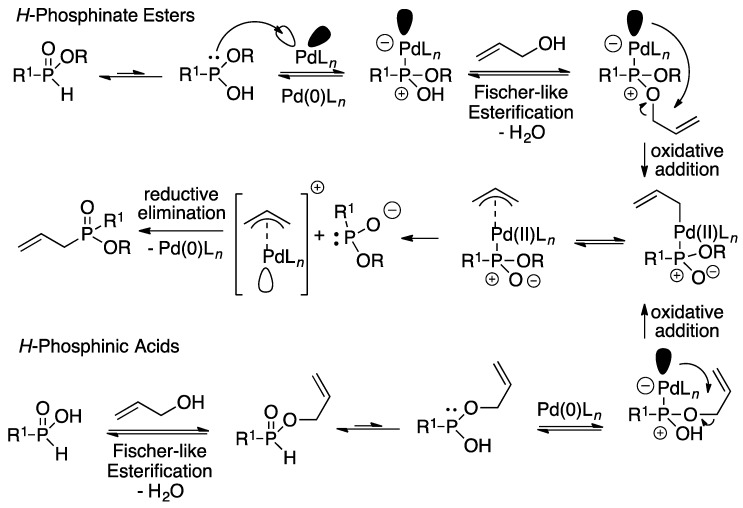
Proposed Mechanisms for the allylation of *H*-phosphinate esters (**top**) and phosphinic acids (**bottom**).

Mechanistically, the present reaction must proceed through a pathway different from the one we have proposed for the allylation of phosphinic acids [6]. Scheme 5 shows a proposed mechanism for the Pd-catalyzed allylation/benzylation of *H*-phosphinate esters.

The key difference between the two mechanisms (Scheme 5) is in the order of tautomerization and esterification. *H*-phosphinic esters are not esterified to the P(III) phosphonite under Dean-Stark conditions. Furthermore, while transesterification is possible in principle, this reaction is inefficient (in the absence of catalysts, particularly bases) and very difficult on esters like cyclohexyl. This would also result in the formation of disubstituted phosphinic acids. The fact that the reaction takes place with Pd(0) complexes but not with Pd(II) (Table 1) also points to a mechanism in which Lewis acidity is not key. Given those facts and some of the limitations in the scope discussed earlier, we propose the following (Scheme 5, top): the *H*-phosphinate ester must tautomerize to the P(III) form, which can then act as a ligand to the Pd(0) catalyst. The resulting putative intermediate would be sufficiently acidic to undergo Fischer-like esterification (with water being removed azeotropically). The esterification of the phosphinite-Pd complex would be a key step. The resulting Pd complex of the mixed phosphonite ester would then undergo oxidative addition (allylO to allylPd migration) to produce the classic Pd(II) intermediate in the Tsuji-Trost reaction. Subsequent formation of a π-allyl complex and attack of the phosphorus nucleophile would afford the allylated product with concomitant reductive elimination to regenerate the Pd(0) catalyst.

In the case of phosphinic acids (HPA or *H*-phosphinic, Scheme 5, bottom), first esterification to produce the allyl ester takes place in a well-established process. Tautomerization of the allyl-*H*-phosphinate ester leads to a phosphonite, which then complexes the Pd(0). Oxidative addition in this complex produces the same type of Pd(II) intermediate (R = H). The mechanism of phosphinic acid allylation (Scheme 5, bottom) is fully consistent with all our prior results [6]. Overall, the success or failure of the allylation depends on various parameters, like the tautomeric equilibrium of the phosphinylidene species, the nucleophilicity of the P(III) tautomer to complex the Pd, and the reactivity of the allylic/benzylic moiety towards oxidative addition.

## 3. Materials and Methods

### 3.1. General Procedure for the Allylation/Benzylation of H-Phosphinates and Related Compounds

To a solution of the appropriate *H*-phosphinate ester (1 equiv.) in *t*-amyl alcohol (10 mL), tris(dibenzylideneacetone)dipalladium(0) Pd_2_(dba)_3_ (1 mol %), Xantphos (2 mol %), and the corresponding alcohol (1 equiv.) were added. The reaction mixture was stirred at reflux for 24 h under N_2_ in a flask equipped with a Dean-Stark trap. After cooling down the reaction to room temperature (rt), the solvent was removed under vacuum and the residue obtained was purified by column chromatography on silica gel using a mixture of hexane/ethyl acetate to afford the different products. The NMR spectra of the products can be found in the Supplementary Materials.

*Butyl cinnamyl phenylphosphinate (**Table 2**, Entry 1)* [13]. General procedure was used with cinnamyl alcohol (0.13 mL, 1 mmol, 1 equiv.) and butyl phenyl-*H*-phosphinate (198 mg, 1 mmol, 1 equiv.). The crude obtained was purified by column chromatography (hexane/ethyl acetate 100:0 to 0:100) to afford the product as a yellow oil (310 mg, 99%). ^31^P-NMR (CDCl_3_, 162 MHz) δ = 39.1 (s); ^1^H-NMR (CDCl_3_, 400 MHz) δ = 7.73–7.80 (m, 2H), 7.47–7.54 (m, 1H), 7.40–7.46 (m, 2H), 7.21–7.27 (m, 4H), 7.14–7.20 (m, 1H), 6.33 (dd, *J* = 5.0 and 15.8 Hz, 1H), 6.04–6.15 (m, 1H), 3.93 (dm, *J* = 95 Hz, 2H), 2.89 (dd, *J* = 7.6 and 18.5 Hz, 2H), 1.63 (quint., *J* = 6.8 Hz, 2H), 1.37 (dsextuplet, *J* = 1.7 and 7.5 Hz, 2H), 0.87 (t, *J* = 7.4 Hz, 3H).

*Cyclohexyl cinnamyl phenylphosphinate (**Table 2**, Entry 2)*. General procedure was used with cinnamyl alcohol (0.13 mL, 1 mmol, 1 equiv.) and cyclohexyl phenyl-*H*-phosphinate (224 mg, 1 mmol, 1 equiv.). The crude obtained was purified by column chromatography (hexane/ethyl acetate 100:0 to 0:100) to afford the product as a yellow oil (311 mg, 91%). ^31^P-NMR (CDCl_3_, 162 MHz) δ = 37.7 (s); ^1^H-NMR (CDCl_3_, 400 MHz) δ = 7.76–7.84 (m, 2H), 7.49–7.55 (m, 1H), 7.41–7.47 (m, 2H), 7.23–7.30 (m, 4H), 7.16–7.22 (m, 1H), 6.35 (dd, *J* = 5.0 and 15.8 Hz, 1H), 6.06–6.17 (m, 1H), 4.30–4.41 (m, 1H), 2.89 (ddd, *J* = 1.0, 7.6 and 18.4 Hz, 2H), 1.97–2.07 (m, 1H), 1.57–1.79 (m, 4H), 1.39–1.52 (m, 2H), 1.15–1.36 (m, 3H); ^13^C-NMR (101 MHz, CDCl_3_): δ = 137.0 (d, *J*_PCCCC_ = 3.3 Hz), 134.9 (d, *J*_PCC_ = 13.2 Hz), 132.2 (d, *J*_PCCCC_ = 2.7 Hz), 131.7 (d, *J*_PCCC_ = 9.6 Hz, 2C), 131.5 (d, *J*_PC_ = 128 Hz), 128.5 (2C), 128.4 (d, *J*_PCC_ = 12.5 Hz, 2C), 127.4, 126.1, 126.1, 118.9 (d, *J*_PCCC_ = 10.3 Hz), 74.7 (d, *J*_POC_ = 6.9 Hz), 35.9 (d, *J*_PC_ = 97.3 Hz), 34.2 (d, *J*_POCC_ = 2.9 Hz), 33.7 (d, *J*_POC_ = 4.1 Hz), 25.1, 23.6, 23.6; HRMS (EI+) *m*/*z* calcd for C_21_H_26_O_2_P ([M + H]^+^) 341.1665, found 341.1675.

*Benzyl cinnamyl phenylphosphinate (**Table 2**, Entry 3)*. General procedure was used with cinnamyl alcohol (0.13 mL, 1 mmol, 1 equiv.) and benzyl phenyl-*H*-phosphinate (232 mg, 1 mmol, 1 equiv.). The crude obtained was purified by column chromatography (hexane/ethyl acetate 100:0 to 0:100) to afford the product as an orange oil (248 mg, 71%). ^31^P-NMR (CDCl_3_, 162 MHz) δ = 41.4 (s); ^1^H-NMR (CDCl_3_, 400 MHz) δ = 7.80–7.87 (m, 2H), 7.54–7.60 (m, 1H), 7.45–7.52 (m, 2H), 7.31–7.40 (m, 5H), 7.26–7.31 (m, 4H), 7.20–7.25 (m, 1H), 6.38 (dd, *J* = 5.2 and 15.9 Hz, 1H), 6.08–6.19 (m, 1H), 5.18 (dd, *J* = 7.3 and 11.8 Hz, 1H), 4.88 (dd, *J* = 7.2 and 11.8 Hz, 1H), 2.99 (dd, *J* = 7.6 and 18.7 Hz, 2H); ^13^C-NMR (101 MHz, CDCl_3_): δ = 136.9 (d, *J*_PCCCC_ = 3.5 Hz), 136.4 (d, *J*_POCC_ = 6.8 Hz), 135.3 (d, *J*_PCC_ = 13.3 Hz), 132.6 (d, *J*_PCCCC_ = 2.5 Hz), 131.9 (d, *J*_PCCC_ = 9.7 Hz, 2C), 130.1 (d, *J*_PC_ = 125 Hz), 128.7 (d, *J*_PCC_ = 12.3 Hz, 2C), 128.6 (2C), 128.5 (2C), 128.4, 127.9 (2C), 127.6, 126.3, 126.2, 118.4 (d, *J*_PCCC_ = 10.4 Hz), 66.3 (d, *J*_POC_ = 6.4 Hz), 35.3 (d, *J*_PC_ = 96.3 Hz); HRMS (EI+) *m*/*z* calcd for C_22_H_22_O_2_P ([M + H]^+^) 349.1357, found 349.1379.

*Cyclohexyl cinnamyl octylphosphinate (**Table 2**, Entry 4)*. General procedure was used with cinnamyl alcohol (0.13 mL, 1 mmol, 1 equiv.) and cyclohexyl octyl-*H*-phosphinate (260 mg, 1 mmol, 1 equiv.). The crude obtained was purified by column chromatography (hexane/ethyl acetate 50:50 to 0:100) to afford the product as an orange oil (328 mg, 87%). ^31^P-NMR (CDCl_3_, 162 MHz) δ = 51.6 (s); ^1^H-NMR (CDCl_3_, 400 MHz) δ = 7.24–7.35 (m, 4H), 7.16–7.22 (m, 1H), 6.47 (dd, *J* = 4.5 and 15.8 Hz, 1H), 6.10–6.22 (m, 1H), 4.34–4.46 (m, 1H), 2.71 (dd, *J* = 7.7 and 17.3 Hz, 2H), 1.83–1.94 (m, 2H), 1.42–1.75 (m, 8H), 1.15–1.37 (m, 14 H), 0.84 (t, *J* = 6.8 Hz, 3 H); ^13^C-NMR (101 MHz, CDCl_3_): δ = 136.8 (d, *J*_PCCCC_ = 3.0 Hz), 134.4 (d, *J*_PCC_ = 12.6 Hz), 128.5 (2C), 127.5, 126.1, 126.1, 119.7 (d, *J*_PCCC_ = 9.4 Hz), 73.7 (d, *J*_POC_ = 7.0 Hz), 34.6 (d, *J*_PC_ = 82.2 Hz), 34.2 (d, *J*_POCC_ = 5.6 Hz), 34.1, 31.8, 30.8 (d, *J*_PCC_ = 15.1 Hz), 29.0, 29.0, 28.3 (d, *J*_PC_ = 93.1 Hz), 25.2, 23.7 (2C), 22.6, 21.6 (d, *J*_PCCC_ = 4.2 Hz), 14.1; HRMS (EI+) *m*/*z* calcd for C_23_H_38_O_2_P ([M + H]^+^) 377.2609, found 377.2531.

*Butyl bis cinnamylphosphinate (**Table 2**, Entry 5)*. General procedure was used with cinnamyl alcohol (0.13 mL, 1 mmol, 1 equiv.) and butyl cinnamyl-*H*-phosphinate (238 mg, 1 mmol, 1 equiv.). The crude obtained was purified by column chromatography (hexane/ethyl acetate 50:50 to 0:100) to afford the product as an orange oil (354 mg, 100%). ^31^P-NMR (CDCl_3_, 162 MHz) δ = 49.9 (s); ^1^H-NMR (CDCl_3_, 400 MHz) δ = 7.21–7.33 (m, 8H), 7.14–7.20 (m, 2H), 6.45 (dd, *J* = 4.6 and 15.8 Hz, 2H), 6.11–6.22 (m, 2H), 4.02 (dt, *J* = 6.8 and 7.0 Hz, 2H), 2.73 (dd, *J* = 7.6 and 17.5 Hz, 4H), 1.61 (quint., *J* = 7.2 Hz, 2H), 1.36 (sextuplet, *J* = 7.5 Hz, 2H), 0.88 (t, *J* = 7.4 Hz, 3H); ^13^C-NMR (101 MHz, CDCl_3_): δ = 136.7 (d, *J*_PCCCC_ = 3.0 Hz, 2C), 135.0 (d, *J*_PCC_ = 13.1 Hz, 2C), 128.6 (4C), 127.7 (2C), 126.2 (2C), 126.2 (2C), 118.9 (d, *J*_PCCC_ = 9.6 Hz, 2C), 64.6 (d, *J*_POC_ = 7.2 Hz), 33.3 (d, *J*_PC_ = 88.7 Hz, 2C), 32.8 (d, *J*_POCC_ = 5.6 Hz), 18.8, 13.7; HRMS (EI+) *m*/*z* calcd for C_22_H_28_O_2_P ([M + H]^+^) 355.1827, found 355.1831.

*Butyl 2-[(4-methoxyphenyl)ethyl] cinnamylphosphinate (**Table 2**, Entry 6)*. General procedure was used with cinnamyl alcohol (0.13 mL, 1 mmol, 1 equiv.) and butyl 2-[4-methoxyphenyl)ethyl]-*H*-phosphinate (256 mg, 1 mmol, 1 equiv.). The crude obtained was purified by column chromatography (hexane/ethyl acetate 100:0 to 0:100) to afford the product as an orange oil (261 mg, 70%). ^31^P-NMR (CDCl_3_, 162 MHz) δ = 52.3 (s); ^1^H-NMR (CDCl_3_, 400 MHz) δ = 7.29–7.38 (m, 4H), 7.22–7.28 (m, 1H), 7.10–7.15 (m, 2H), 6.81–6.87 (m, 2H), 6.45 (dd, *J* = 4.6 and 15.8 Hz, 1H), 6.11–6.22 (m, 1H), 4.06 (dt, *J* = 6.6 and 6.7 Hz, 2H), 3.78 (s, 3H), 2.83–2.99 (m, 2H), 2.73 (ddd, *J* = 0.7, 7.2 and 16.8 Hz, 2H), 2.01–2.12 (m, 2H), 1.68 (quint., *J* = 7.5 Hz, 2H), 1.43 (sextuplet, *J* = 7.5 Hz, 2H), 0.95 (t, *J* = 7.4 Hz, 3H); ^13^C-NMR (101 MHz, CDCl_3_): δ = 158.2, 136.7 (d, *J*_PCCCC_ = 3.2 Hz), 134.8 (d, *J*_PCCC_ = 12.9 Hz), 133.0 (d, *J*_PCC_ = 14.6 Hz), 129.1 (2C), 128.6 (2C), 127.7, 126.2, 126.2, 119.1 (d, *J*_PCCC_ = 9.4 Hz, 2C), 114.0 (2C), 64.4 (d, *J*_POC_ = 7.0 Hz), 55.3, 34.0 (d, *J*_PC_ = 86.0 Hz), 32.8 (d, *J*_POCC_ = 5.7 Hz), 29.7 (d, *J*_PC_ = 90.4 Hz), 27.0 (d, *J*_PCC_ = 3.5 Hz), 18.9, 13.7; HRMS (EI+) *m*/*z* calcd for C_22_H_29_O_3_P ([M + H]^+^) 373.1933, found 373.1855.

*Butyl (2-ethylphthalimide) cinnamylphosphinate* *(**Table 2**, Entry 7)*. General procedure was used with cinnamyl alcohol (1.7 mL, 12.94 mmol, 1 equiv.) and butyl (2-ethylphthalimide)-*H*-phosphinate (3.3 g, 12.94 mmol, 1 equiv.). The crude obtained was purified by column chromatography (dichloromethane/acetone 100:0 to 90:10) to afford the product as an orange oil (4.87 g, 100%). ^31^P-NMR (CDCl_3_, 162 MHz) δ = 49.1 (s); ^1^H-NMR (CDCl_3_, 400 MHz) δ = 7.82–7.90 (m, 2H), 7.68–7.75 (m, 2H), 7.20–7.44 (m, 5H), 6.62 (dd, *J* = 4.7 and 15.8 Hz, 1H), 6.16–6.27 (m, 1H), 3.95–4.12 (m, 4H), 2.88 (dd, *J* = 7.5 and 17.5 Hz, 2H), 2.17–2.34 (m, 2H), 1.58 (quint., *J* = 7.1 Hz, 2H), 1.35 (sextuplet, *J* = 7.4 Hz, 2H), 0.89 (t, *J* = 7.4 Hz, 3H); ^13^C-NMR (101 MHz, CDCl_3_): δ = 167.8 (2C), 136.6 (d, *J*_PCCCC_ = 3.2 Hz), 135.3 (d, *J*_PCC_ = 13.1 Hz), 134.1 (2C), 132.0 (2C), 128.6 (2C), 127.7, 126.3, 126.3, 123.4 (2C), 118.5 (d, *J*_PCCC_ = 9.6 Hz), 64.6 (d, *J*_POC_ = 7.0 Hz), 33.7 (d, *J*_PC_ = 88.4 Hz), 32.6 (d, *J*_PCC_ = 5.9 Hz), 31.7, 26.5 (d, *J*_PC_ = 89.7 Hz), 18.8, 13.6; HRMS (EI+) *m*/*z* calcd for C_23_H_27_NO_4_P ([M + H]^+^) 412.1678, found 412.1676.

*Butyl (acetoxymethyl) cinnamylphosphinate (**Table 2**, Entry 8)*. General procedure was used with cinnamyl alcohol (0.13 mL, 1 mmol, 1 equiv.) and butyl (acetoxymethyl)-*H*-phosphinate (194 mg, 1 mmol, 1 equiv.). The crude obtained was purified by column chromatography (hexane/ethyl acetate 100:0 to 0:100) to afford the product as an orange oil (239 mg, 77%). ^31^P-NMR (CDCl_3_, 162 MHz) δ = 43.6 (s); ^1^H-NMR (CDCl_3_, 400 MHz) δ = 7.27–7.32 (m, 2H), 7.21–7.27 (m, 2H), 7.14–7.20 (m, 1H), 6.50 (dd, *J* = 5.0 and 15.9 Hz, 1H), 6.06–6.17 (m, 1H), 4.43 (dd, *J* = 7.8 and 14.5 Hz, 1H), 4.27 (dd, *J* = 4.2 and 14.5 Hz, 1H), 4.03 (dm, *J* = 39.6 Hz, 2H), 2.79 (ddd, *J* = 1.0, 7.7 and 18.5 Hz, 2H), 2.03 (s, 3H), 1.60 (quint., *J* = 7.5 Hz, 2H), 1.34 (sextuplet, *J* = 7.5 Hz, 2H), 0.86 (t, *J* = 7.4 Hz, 3H); ^13^C-NMR (101 MHz, CDCl_3_): δ = 170.0 (d, *J*_PCOC_ = 6.8 Hz), 136.5 (d, *J*_PCCCC_ = 3.5 Hz), 135.5 (d, *J*_PCC_ = 13.3 Hz), 128.6 (2C), 127.8, 126.3, 126.2, 117.5 (d, *J*_PCCC_ = 10.2 Hz), 65.2 (d, *J*_POC_ = 6.9 Hz), 57.9 (d, *J*_PC_ = 108 Hz), 32.6 (d, *J*_POCC_ = 5.8 Hz), 32.5 (d, *J*_PC_ = 93.1 Hz), 20.5 (d, *J*_POCCC_ = 8.8 Hz), 18.7, 13.6; HRMS (EI+) *m*/*z* calcd for C_16_H_24_O_4_P ([M + H]^+^) 311.1407, found 311.1401.

*(R_p_)/(S_p_)-Menthyl cinnamyl(hydroxymethyl)phosphinate* *(**Table 2**, Entry 9)* [14]. To a solution of *(R_p_)/(S_p_)*-menthyl (hydroxymethyl)-*H*-phosphinate (2.34 g, 10 mmol, 1 equiv, 56:44 diastereoisomeric ratio) in *t*-amyl alcohol (30 mL), Pd_2_(dba)_3_ (92 mg, 0.1 mmol, 1 mol %), Xantphos (116 mg, 0.2 mmol, 2 mol %), and cinnamyl alcohol (1.3 mL, 10 mmol, 1 equiv.) were added. The reaction mixture was stirred at reflux for 20 h under N_2_ in a flask equipped with a Dean-Stark trap. After cooling down the reaction to rt, the solvent was removed under vacuum and the residue obtained was purified by column chromatography (dichloromethane/acetone 100:0 to 90:10) to afford the product as a white solid (3.19 g, 91%, 56:44 diastereoisomeric ratio). ^31^P-NMR (162 MHz, CDCl_3_): δ = 48.4 (s, 56%) and 48.1 (s, 44%).

*6-(Cinnamyl)-6H-dibenzo[c,e][1,2λ^5^]oxaphosphinine-6-oxide (**Table 2**, Entry 11)*. General procedure was used with cinnamyl alcohol (0.13 mL, 1 mmol, 1 equiv.) and DOPO (216 mg, 1 mmol, 1 equiv.). The crude obtained was purified by column chromatography (hexane/ethyl acetate 50:50 to 0:100) to afford the product as a yellow solid (318 mg, 96%). ^31^P-NMR (CDCl_3_, 162 MHz) δ = 27.4 (s); ^1^H-NMR (CDCl_3_, 400 MHz) δ = 7.89–7.99 (m, 2H), 7.68–7.74 (m, 1H), 7.49–7.55 (m, 1H), 7.36–7.42 (m, 1H), 7.22–7.30 (m, 6H), 7.16–7.21 (m, 2H), 6.35 (dd, *J* = 5.3 and 15.8 Hz, 1H), 6.02–6.13 (m, 1H), 3.08 (ddt, *J* = 1.3, 7.6 and 18.2 Hz, 2H); ^13^C-NMR (101 MHz, CDCl_3_): δ = 149.6 (d, *J*_POC_ = 8.2 Hz), 136.5 (d, *J*_PCCCC_ = 3.6 Hz), 135.9 (d, *J*_PCCC_ = 6.1 Hz), 135.8 (d, *J*_PCC_ = 13.8 Hz), 133.4 (d, *J*_PCCCC_ = 2.3 Hz), 130.7, 130.5 (d, *J*_PCCC_ = 10.2 Hz), 128.5 (2C), 128.4 (d, *J*_PCC_ = 13.0 Hz), 127.7 (d, *J*_PCCCC_ = 2.2 Hz), 126.2, 126.2, 125.2, 124.6, 124.2 (d, *J*_PC_ = 120 Hz), 123.8 (d, *J*_PCCC_ = 9.6 Hz), 122.3 (d, *J*_PCCC_ = 10.5 Hz), 120.4 (d, *J*_PCCC_ = 6.3 Hz), 117.2 (d, *J*_PCC_ = 11.0 Hz), 34.5 (d, *J*_PC_ = 93.8 Hz); HRMS (EI+) *m*/*z* calcd for C_21_H_18_O_2_P ([M + H]^+^) 333.1039, found 333.1048.

*Diethyl cinnamylphosphonate (**Table 2**, Entry 12)* [15]. General procedure was used with cinnamyl alcohol (0.13 mL, 1 mmol, 1 equiv.) and diethylphosphite (138 mg, 1 mmol, 1 equiv.). The crude obtained was purified by column chromatography (hexane/ethyl acetate 50:50 to 0:100) to afford the product as an orange oil (240 mg, 94%). ^31^P-NMR (CDCl_3,_ 162 MHz) δ = 34.8 (s); ^1^H-NMR (CDCl_3,_ 400 MHz) δ = 7.34–7.40 (m, 2H), 7.28–7.34 (m, 2H), 7.21–7.26 (m, 1H), 6.53 (dd, *J* = 5.1 and 15.8 Hz, 1H), 6.12–6.23 (m, 1H), 4.08–4.18 (m, 4H), 2.76 (dd, *J* = 7.6 and 22.2 Hz, 2H), 1.32 (t, *J* = 6.1 Hz, 6H).

*Cinnamyl diphenylphosphine oxide (**Table 2**, Entry 13)* [16]. General procedure was used with cinnamyl alcohol (0.13 mL, 1 mmol, 1 equiv.) and diphenyl phosphine oxide (202 mg, 1 mmol, 1 equiv.). The crude obtained was purified by column chromatography (hexane/ethyl acetate 100:0 to 0:100) to afford the product as a yellow oil (292 mg, 92%). ^31^P-NMR (CDCl_3_, 162 MHz) δ = 28.9 (s); ^1^H-NMR (CDCl_3_, 400 MHz) δ = 7.75–7.82 (m, 4H), 7.46–7.58 (m, 6H), 7.18–7.29 (m, 5H), 6.44 (dd, *J* = 4.5 and 15.8 Hz, 1H), 6.14–6.25 (m, 1H), 3.31 (ddd, *J* = 1.2, 7.5 and 15.0 Hz, 2H).

*Butyl allyl phenylphosphinate (**Table 3**, Entry 1)* [17]. General procedure was used with allyl alcohol (0.13 mL, 2 mmol, 2 equiv.) and butyl phenyl-*H*-phosphinate (198 mg, 1 mmol, 1 equiv.). The crude obtained was purified by column chromatography (hexane/ethyl acetate 100:0 to 0:100) to afford the product as a yellow oil (234 mg, 98%). ^31^P-NMR (CDCl_3_, 162 MHz) δ = 35.1 (s); ^1^H-NMR (CDCl_3_, 400 MHz) δ = 7.66–7.75 (m, 2H), 7.45–7.51 (m, 1H), 7.36–7.43 (m, 2H), 5.61–5.75 (m, 1H), 4.95–5.09 (m, 1H), 3.93–4.03 (m, 1H), 3.69–3.80 (m, 1H), 3.31 (dd, *J* = 7.0 and 18.5 Hz, 2H), 1.58 (quint., *J* = 7.1 Hz, 2H), 1.32 (sextuplet, *J* = 1.9 and 7.4 Hz, 2H), 0.83 (t, *J* = 7.4 Hz, 3H).

*Cyclohexyl allyl phenylphosphinate (**Table 3**, Entry 2)*. General procedure was used with allyl alcohol (0.13 mL, 2 mmol, 2 equiv.) and cyclohexyl phenyl-*H*-phosphinate (224 mg, 1 mmol, 1 equiv.). The crude obtained was purified by column chromatography (hexane/ethyl acetate 100:0 to 0:100) to afford the product as an orange oil (247 mg, 94%). ^31^P-NMR (CDCl_3_, 162 MHz) δ = 37.7 (s); ^1^H-NMR (CDCl_3_, 400 MHz) δ = 7.70–7.79 (m, 2H), 7.46–7.52 (m, 1H), 7.38–7.45 (m, 2H), 5.64–5.78 (m, 1H), 4.97–5.11 (m, 2H), 4.22–4.33 (m, 1H), 2.65–2.76 (m, 2H), 1.92–2.02 (m, 1H), 1.51–1.75 (m, 4H), 1.34–1.47 (m, 2H), 1.12–1.32 (m, 3H); ^13^C-NMR (101 MHz, CDCl_3_): δ = 132.1 (d, *J*_PCCCC_ = 2.7 Hz), 131.7 (d, *J*_PCCC_ = 9.6 Hz, 2C), 131.4 (d, *J*_PC_ = 126 Hz), 128.3 (d, *J*_PCC_ = 12.5 Hz, 2C), 127.4 (d, *J*_PCCC_ = 9.2 Hz), 120.1 (d, *J*_PCC_ = 13.0 Hz), 74.6 (d, *J*_POC_ = 6.9 Hz), 36.6 (d, *J*_PC_ = 97.5 Hz), 34.2 (d, *J*_POCC_ = 3.1 Hz), 33.7 (d, *J*_POCC_ = 4.3 Hz), 25.1, 23.6, 23.6; HRMS (EI+) *m*/*z* calcd for C_15_H_22_O_2_P ([M + H]^+^) 265.1352, found 265.1359.

*Butyl allyl cinnamylphosphinate (**Table 3**, Entry 3)* [6]. General procedure was used with allyl alcohol (0.15 mL, 2 mmol, 2 equiv.) and butyl cinnamyl-*H*-phosphinate (238 mg, 1 mmol, 1 equiv.). The crude obtained was purified by column chromatography (hexane/ethyl acetate 100:0 to 0:100) to afford the product as an orange oil (254 mg, 91%). ^31^P-NMR (CDCl_3_, 162 MHz) δ = 48.0 (s); ^1^H-NMR (CDCl_3_, 400 MHz) δ = 7.23–7.34 (m, 4H), 7.15–7.21 (m, 1H), 6.46 (dd, *J* = 4.7 and 15.8 Hz, 1H), 6.08–6.19 (m, 1H), 5.72–5.86 (m, 1H), 5.14–5.23 (m, 2H), 4.00 (dt, *J* = 6.8 and 6.9 Hz, 2H), 2.72 (ddd, *J* = 0.7, 7.6 and 17.6 Hz, 2H), 2.60 (dd, *J* = 7.5 and 17.2 Hz, 2H), 1.60 (quint., *J* = 7.1 Hz, 2H), 1.35 (sextuplet, *J* = 7.5 Hz, 2H), 0.88 (t, *J* = 7.4 Hz, 3H); ^13^C-NMR (101 MHz, CDCl_3_): δ = 136.7 (d, *J*_PCCCC_ = 3.3 Hz), 135.0 (d, *J*_PCC_ = 12.9 Hz), 128.6 (2C), 127.7 (d, *J*_PCCC_ = 8.0 Hz), 127.6, 126.2, 126.1, 120.3 (d, *J*_PCC_ = 12.6 Hz), 118.8 (d, *J*_PCCC_ = 9.6 Hz), 64.5 (d, *J*_POC_ = 7.2 Hz), 33.8 (d, *J*_PC_ = 88.7 Hz), 32.9 (d, *J*_PC_ = 89.0 Hz), 32.7 (d, *J*_POCC_ = 5.7 Hz), 18.8, 13.6.

*Cyclohexyl 3-[(2-methyl-2-propene)phenyl] phenylphosphinate (**Table 3**, Entry 4)*. General procedure was used with *trans*-2-methyl-3-phenyl-2-propen-1-ol (0.15 mL, 1 mmol, 1 equiv.) and cyclohexyl phenyl-*H*-phosphinate (224 mg, 1 mmol, 1 equiv.). The crude obtained was purified by column chromatography (hexane/ethyl acetate 100:0 to 0:100) to afford the product as an orange oil (330 mg, 93%). ^31^P-NMR (CDCl_3_, 162 MHz) δ = 39.5 (s); ^1^H-NMR (CDCl_3_, 400 MHz) δ = 7.77–7.85 (m, 2H), 7.48–7.54 (m, 1H), 7.40–7.47 (m, 2H), 7.22–7.29 (m, 2H), 7.11–7.17 (m, 1H), 7.03–7.09 (m, 2H), 6.09 (d, *J* = 5.5 Hz, 1H), 4.29–4.40 (m, 1H), 2.76–2.91 (m, 2H), 1.98–2.07 (m, 1H), 1.92 (dd, *J* = 1.3 and 3.5 Hz, 3H), 1.59–1.79 (m, 4H), 1.39–1.49 (m, 2H), 1.17–1.36 (m, 3H); ^13^C-NMR (101 MHz, CDCl_3_): δ = 137.8 (d, *J*_PCCCC_ = 3.7 Hz), 132.1 (d, *J*_PCCCC_ = 2.7 Hz), 131.9 (d, *J*_PCCC_ = 9.5 Hz, 2C), 131.6 (d, *J*_PC_ = 125 Hz), 130.0 (d, *J*_PCC_ = 11.5 Hz), 129.3 (d, *J*_PCCC_ = 10.3 Hz), 128.7 (d, *J*_PCCCCC_ = 2.9 Hz, 2C), 128.3 (d, *J*_PCC_ = 12.4 Hz, 2C), 128.0 (2C), 126.3, 74.7 (d, *J*_POC_ = 7.0 Hz), 42.8 (d, *J*_PC_ = 95.2 Hz), 34.3 (d, *J*_POCC_ = 2.9 Hz), 33.7 (d, *J*_POCC_ = 4.3 Hz), 25.2, 23.6 (2C), 19.5 (d, *J*_CCC_ = 2.1 Hz); HRMS (EI+) *m*/*z* calcd for C_22_H_28_O_2_P ([M + H]^+^) 355.1827, found 355.1849.

*Butyl 3-[(2-methyl-2-propene)phenyl] cinnamylphosphinate (**Table 3**, Entry 5)*. General procedure was used with *trans*-2-methyl-3-phenyl-2-propen-1-ol (0.15 mL, 1 mmol, 1 equiv.) and butyl cinnamyl-*H*-phosphinate (238 mg, 1 mmol, 1 equiv.). The crude obtained was purified by column chromatography (hexane/ethyl acetate 50:50 to 0:100) to afford the product as an orange oil (283 mg, 77%). ^31^P-NMR (CDCl_3_, 162 MHz) δ = 48.2 (s); ^1^H-NMR (CDCl_3_, 400 MHz) δ = 7.19–7.38 (m, 10H), 6.54 (dd, *J* = 4.4 and 15.8 Hz, 1H), 6.43 (d, *J* = 4.8 Hz, 1H), 6.19–6.30 (m, 1H), 4.10 (dt, *J* = 6.7 and 6.8 Hz, 2H), 2.84 (dd, *J* = 7.6 and 17.1 Hz, 2H), 2.77 (d, *J* = 17.3 Hz, 2H), 2.07 (d, *J* = 3.1 Hz, 3H), 1.68 (quint., *J* = 7.4 Hz, 2H), 1.42 (sextuplet, *J* = 7.5 Hz, 2H), 0.93 (t, *J* = 7.4 Hz, 3H); ^13^C-NMR (101 MHz, CDCl_3_): δ = 137.5 (d, *J*_PCCCC_ = 3.5 Hz), 136.8 (d, *J*_PCCCC_ = 3.1 Hz), 135.0 (d, *J*_PCC_ = 12.7 Hz), 129.9 (d, *J*_PCCC_ = 11.7 Hz), 129.8 (d, *J*_PCC_ = 10.0 Hz), 128.9, 128.8, 128.6 (2C), 128.2 (2C), 127.7, 126.6, 126.2, 126.2, 119.2 (d, *J*_PCCC_ = 9.9 Hz), 64.6 (d, *J*_POC_ = 7.2 Hz), 40.6 (d, *J*_PC_ = 86.7 Hz), 33.5 (d, *J*_PC_ = 88.0 Hz), 32.9 (d, *J*_POCC_ = 5.7 Hz), 19.6 (d, *J*_PCCC_ = 1.9 Hz), 18.9, 13.7; HRMS (EI+) *m*/*z* calcd for C_23_H_30_O_2_P ([M + H]^+^) 369.1983, found 369.1906.

*Butyl (2-methylprop-2-ene) cinnamylphosphinate (**Table 3**, Entry 6)*. General procedure was used with methylallyl alcohol (0.154 mL, 1 mmol, 1 equiv.) and butyl cinnamyl-*H*-phosphinate (238 mg, 1 mmol, 1 equiv.). The crude obtained was purified by column chromatography (hexane/ethyl acetate 100:0 to 0:100) to afford the product as an orange oil (148 mg, 51%). ^31^P-NMR (CDCl_3_, 162 MHz) δ = 47.4 (s); ^1^H-NMR (CDCl_3_, 400 MHz) δ = 7.30–7.39 (m, 4H), 7.22–7.28 (m, 1H), 6.52 (dd, *J* = 4.6 and 15.8 Hz, 1H), 6.14–6.25 (m, 1H), 4.97–5.01 (m, 1H), 4.95 (d, *J* = 4.5 Hz, 1H), 4.06 (dt, *J* = 6.8 Hz, 2H), 2.81 (dd, *J* = 7.6 and 17.3 Hz, 2H), 2.62 (d, *J* = 17.4 Hz, 2H), 1.92 (t, *J* = 1.2 Hz, 3H), 1.66 (quint., *J* = 7.5 Hz, 2H), 1.41 (sextuplet, *J* = 7.6 Hz, 2H), 0.93 (t, *J* = 7.4 Hz, 3H); ^13^C-NMR (101 MHz, CDCl_3_): δ = 136.8 (d, *J*_PCCCC_ = 3.3 Hz), 136.7 (d, *J*_PCCC_ = 9.0 Hz), 134.9 (d, *J*_PCC_ = 13.1 Hz), 128.6 (2C), 127.6, 126.2, 126.2, 119.2 (d, *J*_PCCC_ = 9.7 Hz), 115.7 (d, *J*_PCC_ = 10.9 Hz), 64.6 (d, *J*_POC_ = 7.2 Hz), 37.6 (d, *J*_PC_ = 87.4 Hz), 33.1 (d, *J*_PC_ = 88.3 Hz), 32.8 (d, *J*_POCC_ = 5.8 Hz), 24.1 (d, *J*_PCCC_ = 2.2 Hz), 18.8, 13.7; HRMS (EI+) *m*/*z* calcd for C_17_H_26_O_2_P ([M + H]^+^) 293.1670, found 293.1693.

*Butyl myrtenyl phenylphosphinate (**Table 3**, Entry 7)*. General procedure was used with myrtenol (0.17 mL, 1 mmol, 1 equiv.) and butyl phenyl-*H*-phosphinate (198 mg, 1 mmol, 1 equiv.). The crude obtained was purified by column chromatography (hexane/ethyl acetate 100:0 to 0:100) to afford the product as an orange oil (246 mg, 74%). ^31^P-NMR (CDCl_3_, 162 MHz) δ = 40.3 (s); ^1^H-NMR (CDCl_3_, 400 MHz) δ = 7.64–7.72 (m, 2H), 7.41–7.47 (m, 1H), 7.33–7.40 (m, 2H), 5.15–5.21 (m, 1H), 3.88–3.97 (m, 1H), 3.61–3.71 (m, 1H), 2.52–2.76 (m, 2H), 2.17–2.26 (m, 1H), 2.00–2.12 (m, 3H), 1.87–1.96 (m, 1H), 1.53 (quint., *J* = 7.2 Hz, 2H), 1.30 (sextuplet, *J* = 7.5 Hz, 2H), 1.14 (d, *J* = 10.4 Hz, 3H), 0.99 (dd, *J* = 8.7 and 20.1 Hz, 1H), 0.81 (t, *J* = 7.6 Hz, 3H), 0.72 (d, *J* = 4.0 Hz, 3H); ^13^C-NMR (101 MHz, CDCl_3_): δ = 137.8 (d, *J*_PCC_ = 10.4 Hz), 132.0 (d, *J*_PCCCC_ = 2.8 Hz, 0.5C), 132.0 (d, *J*_PCCCC_ = 2.8 Hz, 0.5C), 131.8 (d, *J*_PCCC_ = 9.7 Hz), 131.8 (d, *J*_PCCC_ = 9.8 Hz), 131.0 (d, *J*_PC_ = 123 Hz), 131.0 (d, *J*_PC_ = 123 Hz), 128.2 (d, *J*_PCC_ = 12.3 Hz, 2C), 122.2 (d, *J*_PCCC_ = 12.6 Hz, 0.5C), 122.2 (d, *J*_PCCC_ = 12.6 Hz, 0.5C), 64.1 (d, *J*_POC_ = 7.0 Hz, 0.5C), 64.0 (d, *J*_POC_ = 7.0 Hz, 0.5C), 46.9 (d, *J*_POCC_ = 2.8 Hz, 0.5C), 46.7 (d, *J*_POCC_ = 2.4 Hz, 0.5C), 40.1 (0.5C), 40.1 (0.5C), 38.7 (d, *J*_PC_ = 97.6 Hz), 37.9 (d, *J*_PCCCC_ = 2.2 Hz, 0.5C), 37.9 (d, *J*_POCC_ = 2.2 Hz, 0.5C), 32.5 (0.5C), 32.5 (0.5C), 31.6 (d, *J*_PCCCC_ = 2.4 Hz, 0.5C), 31.5 (d, *J*_PCCCC_ = 2.0 Hz, 0.5C), 26.1 (0.5C), 26.1 (0.5C), 21.0 (0.5C), 20.9 (0.5C), 18.7, 13.6; HRMS (EI+) *m*/*z* calcd for C_20_H_30_O_2_P ([M + H]^+^) 333.1983, found 333.1906.

*Cyclohexyl benzyl octylphosphinate (**Table 3**, Entry 8)* [1]. General procedure was used with benzyl alcohol (0.11 mL, 1 mmol, 1 equiv.) and cyclohexyl octyl-*H*-phosphinate (260 mg, 1 mmol, 1 equiv.). The crude obtained was purified by column chromatography (hexane/ethyl acetate 50:50 to 0:100) to afford the product as an orange oil (226 mg, 65%). ^31^P-NMR (CDCl_3,_ 162 MHz) δ = 52.1 (s); ^1^H-NMR (CDCl_3_, 400 MHz) δ = 7.22–7.37 (m, 5H), 4.30–4.42 (m, 1H), 3.13 (d, *J* = 16.7 Hz, 2H), 1.86–1.95 (m, 2H), 1.40–1.84 (m, 10H), 1.17–1.37 (m, 12H), 0.89 (t, *J* = 6.9 Hz, 3H).

*Butyl benzyl cinnamylphosphinate (**Table 3**, Entry 9)*. General procedure was used with benzyl alcohol (0.11 mL, 1 mmol, 1 equiv.) and butyl cinnamyl-*H*-phosphinate (238 mg, 1 mmol, 1 equiv.). The crude obtained was purified by column chromatography (hexane/ethyl acetate 100:0 to 0:100) to afford the product as an orange oil (187 mg, 57%). ^31^P-NMR (CDCl_3_, 162 MHz) δ = 48.5 (s); ^1^H-NMR (CDCl_3_, 400 MHz) δ = 7.21–7.39 (m, 10H), 6.45 (dd, *J* = 4.2 and 15.8 Hz, 1H), 6.08–6.20 (m, 1H), 3.93–4.07 (m, 2H), 3.20 (d, *J* = 16.8 Hz, 2H), 2.70 (dd, *J* = 7.6 and 17.2 Hz, 2H), 2.01–2.12 (m, 2H), 1.62 (quint., *J* = 7.3 Hz, 2H), 1.37 (sextuplet, *J* = 7.5 Hz, 2H), 0.91 (t, *J* = 7.4 Hz, 3H); ^13^C-NMR (101 MHz, CDCl_3_): δ = 136.8 (d, *J*_PCCCC_ = 3.1 Hz), 135.1 (d, *J*_PCCC_ = 13.0 Hz), 131.6 (d, *J*_PCC_ = 7.3 Hz), 129.9 (d, *J*_PCCC_ = 5.8 Hz, 2C), 128.7 (d, *J*_PCCCC_ = 2.6 Hz, 2C), 128.6 (2C), 127.7, 127.0 (d, *J*_PCCCCC_ = 3.0 Hz), 126.2, 126.2, 118.9 (d, *J*_PCCC_ = 9.8 Hz), 64.8 (d, *J*_POC_ = 7.0 Hz), 36.0 (d, *J*_PC_ = 87.1 Hz), 33.2 (d, *J*_PC_ = 88.0 Hz), 32.8 (d, *J*_POCC_ = 5.9 Hz), 27.0 (d, *J*_PCC_ = 3.5 Hz), 18.8, 13.7; HRMS (EI+) *m*/*z* calcd for C_20_H_26_O_2_P ([M + H]^+^) 329.1665, found 329.1671.

*Cyclohexyl 1-(naphthylmethyl) phenylphosphinate (**Table 3**, Entry 10)*. General procedure was used with 1-naphthalenemethanol (158 mg, 1 mmol, 1 equiv.) and cyclohexyl phenyl-*H*-phosphinate (224 mg, 1 mmol, 1 equiv.). The crude obtained was purified by column chromatography (hexane/ethyl acetate 100:0 to 0:100) to afford the product as an orange oil (266 mg, 73%). ^31^P-NMR (CDCl_3_, 162 MHz) δ = 35.5 (s); ^1^H-NMR (CDCl_3_, 400 MHz) δ = 7.95–8.02 (m, 1H), 7.77–7.84 (m, 1H), 7.69–7.75 (m, 1H), 7.56–7.65 (m, 2H), 7.39–7.48 (m, 3H), 7.29–7.37 (m, 3H), 7.22–7.28 (m, 1H), 4.22–4.33 (m, 1H), 3.76 (d, *J* = 17.8 Hz, 2H), 1.77–1.86 (m, 1H), 1.45–1.67 (m, 4H), 1.09–1.44 (m, 5H); ^13^C-NMR (101 MHz, CDCl_3_): δ = 133.8 (d, *J*_PCCCC_ = 2.1 Hz), 132.2 (d, *J*_PCCCC_ = 4.3 Hz), 132.1 (d, *J*_PCCCC_ = 2.2 Hz), 132.0 (d, *J*_PCCC_ = 9.5 Hz, 2C), 131.4 (d, *J*_PC_ = 124 Hz), 128.6 (d, *J*_PCCC_ = 6.7 Hz), 128.4, 128.4 (d, *J*_PCC_ = 8.0 Hz), 128.2 (d, *J*_PCC_ = 12.4 Hz, 2C), 127.5 (d, *J*_PCCCC_ = 3.8 Hz), 125.7, 125.5, 125.2 (d, *J*_PCCCC_ = 3.6 Hz), 124.6, 74.9 (d, *J*_POC_ = 6.8 Hz), 35.9 (d, *J*_PC_ = 96.1 Hz), 34.0 (d, *J*_POCC_ = 2.6 Hz), 33.5 (d, *J*_POCC_ = 4.0 Hz), 25.1, 23.4 (2C); HRMS (EI+) *m*/*z* calcd for C_22_H_28_O_2_P ([M + H]^+^) 355.1827, found 355.1849.

*Butyl (1-methylnaphthalene) cinnamylphosphinate (**Table 3**, Entry 11)*. General procedure was used with 1-naphthalenemethanol (316 mg, 2 mmol, 2 equiv.) and butyl cinnamyl-*H*-phosphinate (238 mg, 1 mmol, 1 equiv.). The crude obtained was purified by column chromatography (hexane/ethyl acetate 100:0 to 0:100) to afford the product as an orange oil (186 mg, 49%). ^31^P-NMR (CDCl_3,_ 162 MHz) δ = 47.5 (s); ^1^H-NMR (CDCl_3_, 400 MHz) δ = 8.09–8.14 (m, 1H), 7.85–7.90 (m, 1H), 7.76–7.82 (m, 1H), 7.49–7.58 (m, 2H), 7.42–7.48 (m, 1H), 7.29–7.36 (m, 5H), 7.23–7.29 (m, 1H), 6.40 (dd, *J* = 4.5 and 15.8 Hz, 1H), 6.09–6.20 (m, 1H), 3.83–4.01 (m, 2H), 3.62–3.78 (m, 2H), 2.75 (dd, *J* = 7.6 and 16.8 Hz, 2H), 1.51 (quint., *J* = 7.4 Hz, 2H), 1.27 (sextuplet, *J* = 7.6 Hz, 2H), 0.85 (t, *J* = 7.4 Hz, 3H); ^13^C-NMR (101 MHz, CDCl_3_): δ = 136.7 (d, *J*_PCCCC_ = 3.1 Hz), 135.0 (d, *J*_PCC_ = 12.8 Hz), 134.0 (d, *J*_PCCCC_ = 2.4 Hz), 132.1 (d, *J*_PCCC_ = 4.6 Hz), 128.8, 128.6 (2C), 128.5 (d, *J*_PCCCC_ = 6.7 Hz), 128.1 (d, *J*_PCC_ = 8.2 Hz), 127.9 (d, *J*_PCCCC_ = 3.8 Hz), 127.7, 126.2 (3C), 125.9, 125.4 (d, *J*_PCCCC_ = 3.6 Hz), 124.4 (d, *J*_PCCCCC_ = 1.2 Hz), 118.9 (d, *J*_PCCC_ = 9.9 Hz), 64.9 (d, *J*_POC_ = 7.3 Hz), 33.7 (d, *J*_PC_ = 88.2 Hz), 33.2 (d, *J*_PC_ = 87.7 Hz), 32.7 (d, *J*_POCC_ = 5.9 Hz), 18.7, 13.6; HRMS (EI+) *m*/*z* calcd for C_24_H_28_O_2_P ([M + H]^+^) 379.1827, found 379.1750.

### 3.2. 1-Butoxy-3-Phospholene 1-Oxide (Scheme 3c) [6]

To a solution of cinnamyl phosphinic acid [13] (3.64 g, 20 mmol, 1 equiv.) in toluene (40 mL), 1-butanol (3.7 mL, 40 mmol, 2 equiv.) was added and the reaction was stirred for 16 h at reflux in a flask equipped with a Dean-Stark trap. After cooling down the reaction to room temperature, the solvent was removed under vacuum. The residue obtained was dissolved in ethyl acetate and the organic layer was washed with an aqueous saturated solution of NaHCO_3_ and brine, dried over MgSO_4_, filtered, and concentrated under vacuum. The residue obtained was purified by column chromatography (hexane/ethyl acetate 80:0 to 20:80) to afford butyl cinnamyl-*H*-phosphinate as a yellow oil (4.59 g, 96%) [1]. ^31^P-NMR (CDCl_3_, 162 MHz) δ = 36.2 (dm, *J* = 543 Hz); ^1^H-NMR (CDCl_3_, 400 MHz) δ = 7.31–7.41 (m, 4H), 7.23–7.29 (m, 1H), 7.07 (dt, *J* = 1.9 and 543 Hz, 1H), 6.57 (dd, *J* = 6.0 and 15.9 Hz, 1H), 6.06–6.18 (m, 1H), 4.12–4.21 (m, 1H), 4.00–4.09 (m, 1H), 2.83 (dd, *J* = 7.6 and 16.8 Hz, 2H), 1.71 (quint., *J* = 7.1 Hz, 2H), 1.43 (sextuplet, *J* = 7.5 Hz, 2H), 0.95 (t, *J* = 7.4 Hz, 3H).

General procedure was used with allyl alcohol (0.15 mL, 2 mmol, 2 equiv.) and butyl cinnamyl-*H*-phosphinate (238 mg, 1 mmol, 1 equiv.). The crude obtained was purified by column chromatography (hexane/ethyl acetate 100:0 to 0:100) to afford butyl allyl cinnamylphosphinate as an orange oil (254 mg, 91%) [6]. ^31^P-NMR (CDCl_3_, 162 MHz) δ = 48.0 (s); ^1^H-NMR (CDCl_3_, 400 MHz) δ = 7.23–7.34 (m, 4H), 7.15–7.21 (m, 1H), 6.46 (dd, *J* = 4.7 and 15.8 Hz, 1H), 6.08–6.19 (m, 1H), 5.72–5.86 (m, 1H), 5.14–5.23 (m, 2H), 4.00 (dt, *J* = 6.8 and 6.9 Hz, 2H), 2.72 (ddd, *J* = 0.7, 7.6 and 17.6 Hz, 2H), 2.60 (dd, *J* = 7.5 and 17.2 Hz, 2H), 1.60 (quint., *J* = 7.1 Hz, 2H), 1.35 (sextuplet, *J* = 7.5 Hz, 2H), 0.88 (t, *J* = 7.4 Hz, 3H); ^13^C-NMR (101 MHz, CDCl_3_): δ = 136.7 (d, *J*_PCCCC_ = 3.3 Hz), 135.0 (d, *J*_PCC_ = 12.9 Hz), 128.6 (2C), 127.7 (d, *J*_PCCC_ = 8.0 Hz), 127.6, 126.2, 126.1, 120.3 (d, *J*_PCC_ = 12.6 Hz), 118.8 (d, *J*_PCCC_ = 9.6 Hz), 64.5 (d, *J*_POC_ = 7.2 Hz), 33.8 (d, *J*_PC_ = 88.7 Hz), 32.9 (d, *J*_PC_ = 89.0 Hz), 32.7 (d, *J*_POCC_ = 5.7 Hz), 18.8, 13.6.

To a solution of butyl allyl cinnamylphosphinate (92 mg, 0.33 mmol, 1 equiv.) in dichloromethane (50 mL), [1,3-bis(2,4,6-trimethylphenyl)-2-imidazolidinylidene] dichloro(phenylmethylene)-(tricyclohexylphosphine)ruthenium (5.6 mg, 0.0066 mmol, 0.02 equiv.) was added and the mixture was placed under N_2_. The reaction was heated at reflux for 12 h and then allowed to cool down to rt and treated with activated charcoal (0.1 g). The resulting suspension was stirred for 12 h at rt, suction-filtered through a Celite pad in a Buchner funnel, and concentrated under vacuum. The crude obtained was purified by column chromatography (hexane/ethyl acetate 100:0 to 0:100) to afford an orange oil (48 mg, 85%). ^31^P-NMR (CDCl_3_, 162 MHz) δ = 74.2 (s); ^1^H-NMR (CDCl_3_, 400 MHz) δ = 5.91 (d, *J* = 33.0 Hz, 2H), 4.04 (dt, *J* = 6.9 and 7.0 Hz, 2H), 2.35–2.50 (m, 4H), 1.67 (quint., *J* = 7.1 Hz, 2H), 1.40 (sextuplet, *J* = 7.5 Hz, 2H), 0.93 (t, *J* = 7.4 Hz, 3H); ^13^C-NMR (101 MHz, CDCl_3_): δ = 127.0 (d, *J*_PCC_ = 15.4 Hz, 2C), 64.7 (d, *J*_POC_ = 6.7 Hz), 32.6 (d, *J*_POCC_ = 6.0 Hz), 29.2 (d, *J*_PC_ = 91.2 Hz, 2C), 18.8, 13.6.

### 3.3. (R_p_)-Menthyl Cinnamyl(hydroxymethyl)phosphinate (Scheme 4) [14]

To a solution of *(R_p_)*-menthyl (hydroxymethyl)-*H*-phosphinate (468 mg, 2 mmol, 1 equiv., >99% diastereoisomeric excess) in *t*-amyl alcohol (10 mL), Pd_2_(dba)_3_ (18.3 mg, 0.02 mmol, 1 mol %), Xantphos (23.2 mg, 0.04 mmol, 2 mol %) and cinnamyl alcohol (0.26 mL, 2 mmol, 1 equiv.) were added. The reaction mixture was stirred at reflux for 20 h under N_2_ in a flask equipped with a Dean-Stark trap. After cooling down the reaction to rt, the solvent was removed under vacuum and the residue obtained was purified by column chromatography (dichloromethane/acetone 100:0 to 90:10) to afford the product as a white solid (681 mg, 97%, >99% diastereoisomeric excess). Mp = 145–146 °C; ^31^P-NMR (162 MHz, CDCl_3_): δ = 48.8 (s); ^1^H-NMR (400 MHz, CDCl_3_): δ = 7.19–7.39 (m, 5H), 6.55 (dd, *J* = 4.7 and 15.8 Hz, 1H), 6.12–6.27 (m, 1H), 4.20–4.34 (m, 1H), 3.87 (s, 2H), 3.64 (s, 1H), 2.85 (dd, *J* = 7.6 and 17.6 Hz, 2H), 2.06–2.22 (m, 2H), 1.60–1.71 (m, 2H), 1.28–1.54 (m, 2H), 1.15 (q, *J* = 11.7 Hz, 1H), 0.74–1.07 (m, 2H), 0.91 (d, *J* = 6.4 Hz, 3H), 0.86 (d, *J* = 6.8 Hz, 3H), 0.77 (d, *J* = 7.0 Hz, 3H); ^13^C-NMR (101 MHz, CDCl_3_): δ = 136.8 (d, *J*_PCCCC_ = 3.3 Hz), 135.0 (d, *J*_PCC_ = 12.2 Hz), 128.5 (2C), 127.5, 126.2 (d, *J*_PCCCCC_ = 1.7 Hz, 2C), 118.4 (d, *J*_PCCC_ = 10.5 Hz), 76.7 (d, *J*_POC_ = 8.3 Hz), 59.5 (d, *J*_PC_ = 106 Hz), 48.6 (d, *J*_POCC_ = 5.6 Hz, 2C), 43.5, 34.0, 31.6 (d, *J*_PC_ = 87.3 Hz), 31.5, 25.5, 22.7, 22.1, 21.0, 15.5; HRMS (EI+) *m*/*z* calcd for C_20_H_31_O_3_P ([M]^+^) 350.2011, found 350.2012; ${\left[\alpha \right]}_{D}^{24}\;$−51.6° (*c* 1 g/100 mL, chloroform).

## 4. Conclusions

We have described the Pd-catalyzed allylation/benzylation of *H*-phosphinate esters using alcohols as the electrophilic partner. While the scope of this reaction is somewhat narrower than when phosphinic acids are used, it still constitutes a useful synthetic methodology for the synthesis of disubstituted phosphinic acid derivatives. In addition, because *H*-phosphinate esters can be produced easily by Dean-Stark esterification of the corresponding acids, it avoids the need for cumbersome esterification procedures on disubstituted phosphinic acids. Therefore, when the present reaction is successful, the sequence esterification-allylation/benzylation is superior to the reverse sequence allylation/benzylation-esterification. A possible mechanism is also proposed.

## Figures and Tables

**Table 1:** Test reaction between butyl phenyl-*H*-phosphinate and cinnamyl alcohol. 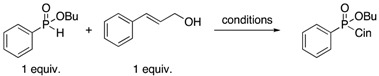

Entry	Solvent ^a^	Temp. (°C) ^a^	Pd Salt (mol %)	Ligand (mol %)	Yield (%) ^b^
1	*t*-AmOH	102	Pd(OAc)_2_ (2)	None (-)	0 ^c^
2	*t*-AmOH	102	Pd(OAc)_2_ (2)	Xantphos (2) ^d^	99
3	toluene	111	Pd(OAc)_2_ (2)	Xantphos (2) ^d^	88
4	*t*-AmOH	102	PdCl_2_ (2)	Xantphos (2) ^d^	90
5	*t*-AmOH	102	Pd_2_(dba)_3_ (1) ^e^	Xantphos (2) ^d^	99
6	*t*-AmOH	102	Pd_2_(dba)_3_ (1) ^e^	Dppf (2) ^f^	67

^a^ Reflux, Dean-Stark trap; ^b^ Isolated yield after column chromatography over silica gel (Note: the ^31^P-NMR yield of the crude before purification is comparable); ^c^ No product in the ^31^P-NMR spectrum; ^d^ Xantphos, 4,5-bis(diphenylphosphino)-9,9-dimethylxanthene; ^e^ Pd_2_(dba)_3_, tris(dibenzylideneacetone)dipalladium(0); dba, dibenzylideneacetone; ^f^ Dppf, 1,1’-bis(diphenylphosphino)ferrocene.

**Table 2:** Allylation of various phosphinylidene compounds with cinnamyl alcohol. 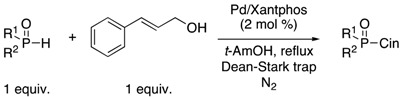

Entry	R^1^	R^2^	Isolated Yield (%) ^a^
1	Ph	OBu	99
2	Ph	OCy	91
3	Ph	OBn	77
4	Oct	OCy	87
5	PhCH=CHCH_2_ (Cin)	OBu	100
6	4-MeOC_6_H_4_CH_2_CH_2_	OBu	70
7	PhtNCH_2_CH_2_	OBu	100
8	AcOCH_2_	OBu	77
9	HOCH_2_	OMen ^b,c^	91
10	MeC(OEt)_2_	OEt	47 ^d,e^
11	DOPO ^f^		96
12	OEt	OEt	94
13	Ph	Ph	92

^a^ Isolated yield after column chromatography over silica gel. Unless otherwise noted, the palladium salt is Pd_2_(dba)_3_ (1 mol %); ^b^ Men, (−)-menthyl; ^c^ 50:50 diastereoisomeric mixture; ^d^ Pd(OAc)_2_ (2 mol %) was used; ^e^ ^31^P-NMR yield; ^f^ DOPO, 6*H*-dibenzo[*c*,*e*][1,2λ^5^]oxaphosphinine 6-oxide.

**Table 3:** Allylation and benzylation of various phosphinylidene compounds.

Entry	Phosphorus P(O)H Compound	Alcohol	Product	Isolated Yield (%) ^a^
1	PhP(O)(OBu)H	H_2_C=CHCH_2_OH (2 equiv.)	PhP(O)(OBu)CH_2_CH=CH_2_	98
2	PhP(O)(OCy)H	H_2_C=CHCH_2_OH (2 equiv.)	PhP(O)(OCy)CH_2_CH=CH_2_	94
3	CinP(O)(OBu)H	H_2_C=CHCH_2_OH (2 equiv.)	CinP(O)(OBu)CH_2_CH=CH_2_	91
4	PhP(O)(OCy)H	2-Methyl-3-phenyl-2-propen-1-ol (1 equiv.)	PhP(O)(OCy)CH_2_C(Me)=CHPh	93
5	CinP(O)(OBu)H	2-Methyl-3-phenyl-2-propen-1-ol (1 equiv.)	CinP(O)(OBu)CH_2_C(Me)=CHPh	77
6	CinP(O)(OBu)H	Methallyl alcohol (1 equiv.)	CinP(O)(OBu)CH_2_C(Me)=CH_2_	51
7	PhP(O)(OBu)H	Myrtenol (1 equiv.)	PhP(O)(OBu)C_10_H_15_	74
8	OctP(O)(OCy)H	PhCH_2_OH (1 equiv.)	OctP(O)(OCy)CH_2_Ph	65
9	CinP(O)(OBu)H	PhCH_2_OH (1 equiv.)	CinP(O)(OBu)CH_2_Ph	57
10	PhP(O)(OCy)H	1-NpCH_2_OH (5 equiv.)	PhP(O)(OCy)CH_2_-1-Np	73
11	CinP(O)(OBu)H	1-NpCH_2_OH (1 equiv.)	CinP(O)(OBu)CH_2_-1-Np	49
12	OctP(O)(OCy)H	Furfuryl alcohol (2 equiv.)	OctP(O)(OCy)CH_2_(OC_4_H_3_)	24 ^b^
13	OctP(O)(OBn)H	PhCH_2_OH (1 equiv.)	OctP(O)(OBn)CH_2_Ph	18 ^b^
14	(EtO)_2_P(O)H	PhCH_2_OH (2 equiv.)	(EtO)_2_P(O)CH_2_Ph	23 ^b^

^a^ Isolated yield after column chromatography over silica gel. The palladium salt is Pd_2_(dba)_3_ (1 mol %); ^b^ ^31^P-NMR yield.

## Supplementary Materials

Supplementary materials can be accessed at: http://www.mdpi.com/1420-3049/21/10/1295/s1.

## Abbreviations

The following abbreviations are used in this manuscript:

HPA	hypophosphorous acid (H_3_PO_2_)
MS	molecular sieves
*t*-AmOH	*t*-amyl alcohol
Pd_2_(dba)_3_	tris(dibenzylideneacetone)dipalladium(0)
Dba	dibenzylideneacetone
Dppf	1,1’-bis(diphenylphosphino)ferrocene
Xantphos	4,5-bis(diphenylphosphino)-9,9-dimethylxanthene
Cin	cinnamyl
Cy	cyclohexyl
Np	naphthyl
Men	(-)-menthyl
DOPO	6*H*-dibenzo[*c*,*e*][1,2λ^5^]oxaphosphinine 6-oxide
HMDS	hexamethyldisilazane
IMes	1,3-bis(2,4,6-trimethylphenyl)imidazol-2-ylidene
RCM	ring closing metathesis
