# Supplementary material for: Palladium-Catalyzed Allylation/Benzylation of H-Phosphinate Esters with Alcohols
Source: Molecules. 2016 Sep 28;21(10):1295. doi: 10.3390/molecules21101295 (PMC6273960; doi:10.3390/molecules21101295)

Supplementary Materials: Palladium-Catalyzed Allylation/Benzylation of *H*-Phosphinate Esters With Alcohols

Anthony Fers-Lidou, Olivier Berger and Jean-Luc Montchamp

List of contents: Page

General Chemistry S2

Spectra S3

General Chemistry:

^{1}H NMR spectra were recorded on a 400-MHz Bruker Avance spectrometer (Bruker Daltonics Inc., Billerica, Massachusetts, USA). Chemical shifts for ^{1}H NMR spectra (in parts per million) relative to internal tetramethylsilane (Me_4Si, δ = 0.00 ppm, s = singulet, d = doublet, m = multiplet, dd = doublet of doublets, dm = doublet of multiplets, quint. = quintuplet, t = triplet, ddd = doublet of doublet of doublets, dt = doublet of triplets, ddt = doublet of doublet of triplets,) with deuterated chloroform. ^{13}C NMR spectra were recorded at 75.5 or 101 MHz. Chemical shifts for ^{13}C NMR spectra are reported (in parts per million) relative to $CDCl_3$ (δ = 77.0 ppm). ^{31}P NMR spectra were recorded at 121.5 or 162 MHz, and chemical shifts reported (in parts per million) relative to external 85% phosphoric acid (δ = 0.0 ppm). Flash chromatography experiments were carried out on Silica Gel premium Rf grade (60 Å, 40−75 µm, Sorbent Technologies, Norcross, Georgia, USA). Ethyl acetate/hexane mixtures or dichloromethane/acetone were used as the eluent for chromatographic purifications. Whatman TLC plates (60 Å, thermo Fisher Scientific, Waltham, Massachusetts, USA) were visualized by UV or immersion in permanganate potassium (3 g $KMnO_4$, 20 g K_2CO_3, 5 mL 5% aq. NaOH and 300 mL of water) followed by heating. High resolution mass spectra (HRMS) were obtained either by direct probe (EI/CI) and analyzed by magnetic sector, or by electrospray using a TOF analyzer.

Reagent and solvents:

All starting materials were purchased from commercial sources and used as received. The solvents were distilled under N_2 and dried according to standard procedures (THF from Na/ benzophenone ketyl; DMF from $MgSO_4$; CH_3CN, toluene and dichloromethane from CaH_2).

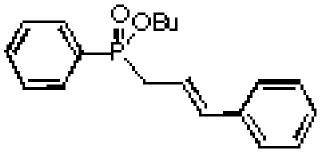

Table 2, entry 1
$^{31}P/^{1}H$ NMR decoupled

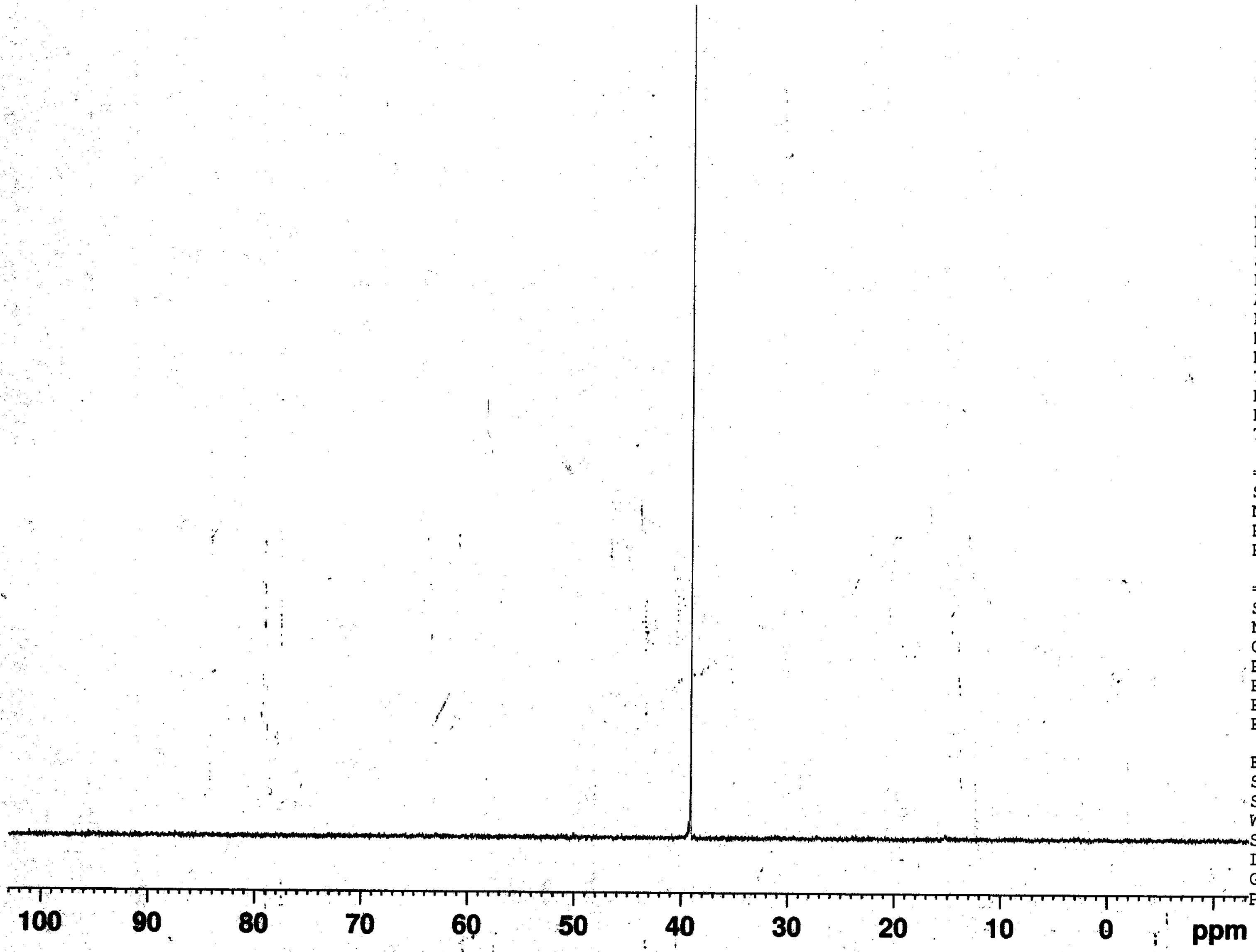

Current Data Parameters
NAME AFL001bis
EXPNO 1
PROCNO 1

F2 - Acquisition Parameters
Date_ 20150609
Time 8.52
INSTRUM spect
PROBHD 5 mm PABBO BB/
PULPROG zgpg30
TD 65536
SOLVENT DMSO
NS 16
DS 4
SWH 64102.563 Hz
FIDRES 0.978127 Hz
AQ 0.5111808 sec
RG 203.57
DW 7.800 usec
DE 6.50 usec
TE 295.0 K
D1 2.00000000 sec
D11 0.03000000 sec
TD0 1

======== CHANNEL f1 ========
SFO1 161.9674942 MHz
NUC1 31P
P1 14.25 usec
PLW1 15.00000000 W

======== CHANNEL f2 ========
SFO2 400.1316005 MHz
NUC2 1H
CPDPRG[2 waltz16
PCPD2 90.00 usec
PLW2 10.00000000 W
PLW12 0.31604999 W
PLW13 0.25600001 W

F2 - Processing parameters
SI 32768
SF 161.9755930 MHz
WDW EM
SSB 0
LB 1.00 Hz
GB 0
PC 1.40

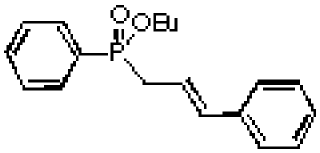

Table 2, entry 1
$^{31}P/^{1}H$ NMR coupled

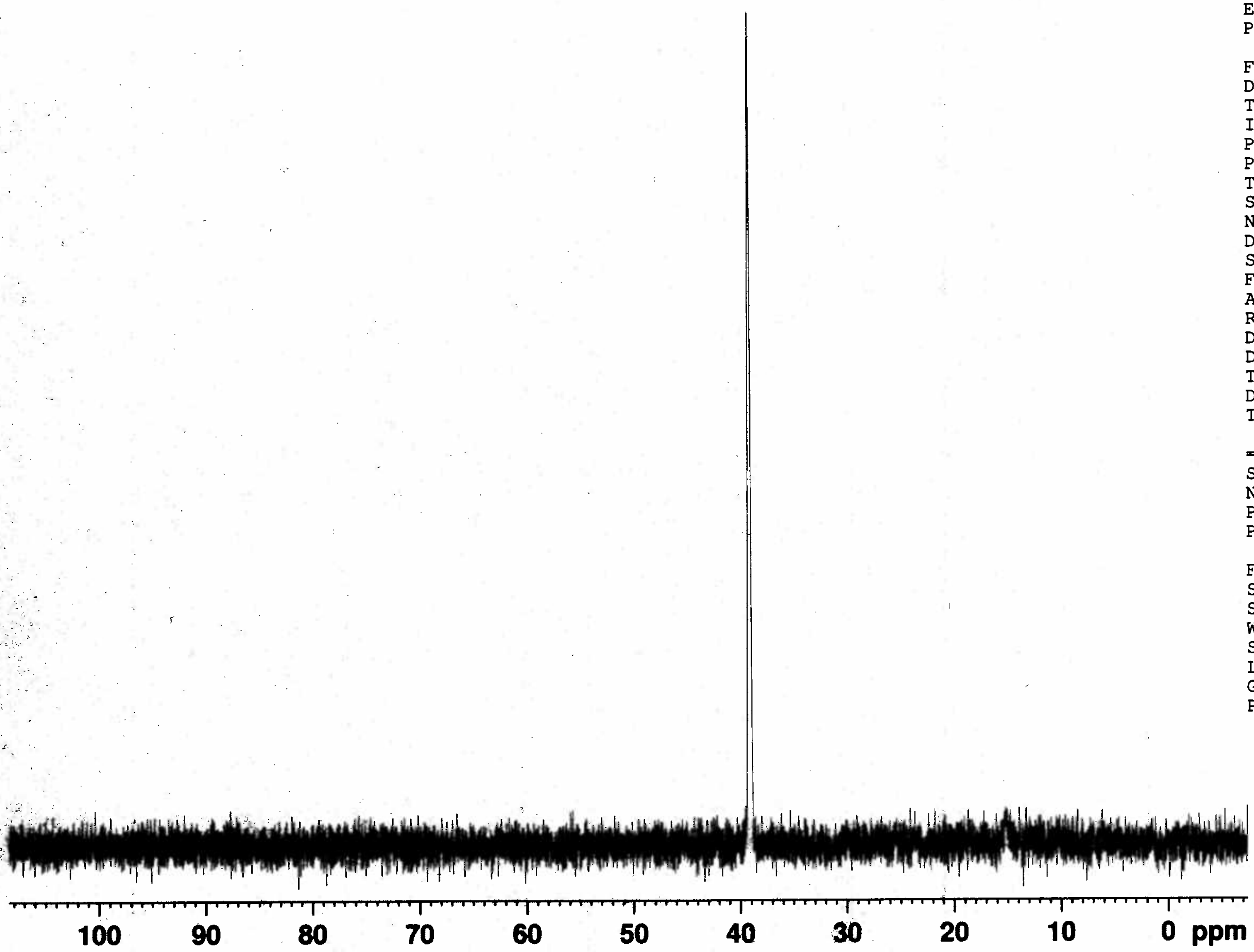

Current Data Parameters
NAME AFL001bis
EXPNO 2
PROCNO 1

F2 - Acquisition Parameters
Date_ 20150609
Time 8.57
INSTRUM spect
PROBHD 5 mm PABBO BB/
PULPROG zg30
TD 65536
SOLVENT DMSO
NS 32
DS 4
SWH 64102.563 Hz
FIDRES 0.978127 Hz
AQ 0.5111808 sec
RG 203.57
DW 7.800 usec
DE 6.50 usec
TE 294.1 K
D1 2.00000000 sec
TD0 1

======== CHANNEL f1 ========
SFO1 161.9674942 MHz
NUC1 31P
P1 14.25 usec
PLW1 15.00000000 W

F2 - Processing parameters
SI 32768
SF 161.9755930 MHz
WDW EM
SSB 0
LB 1.00 Hz
GB 0
PC 1.40

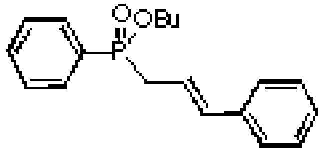

Table 2, entry 1
^1H NMR

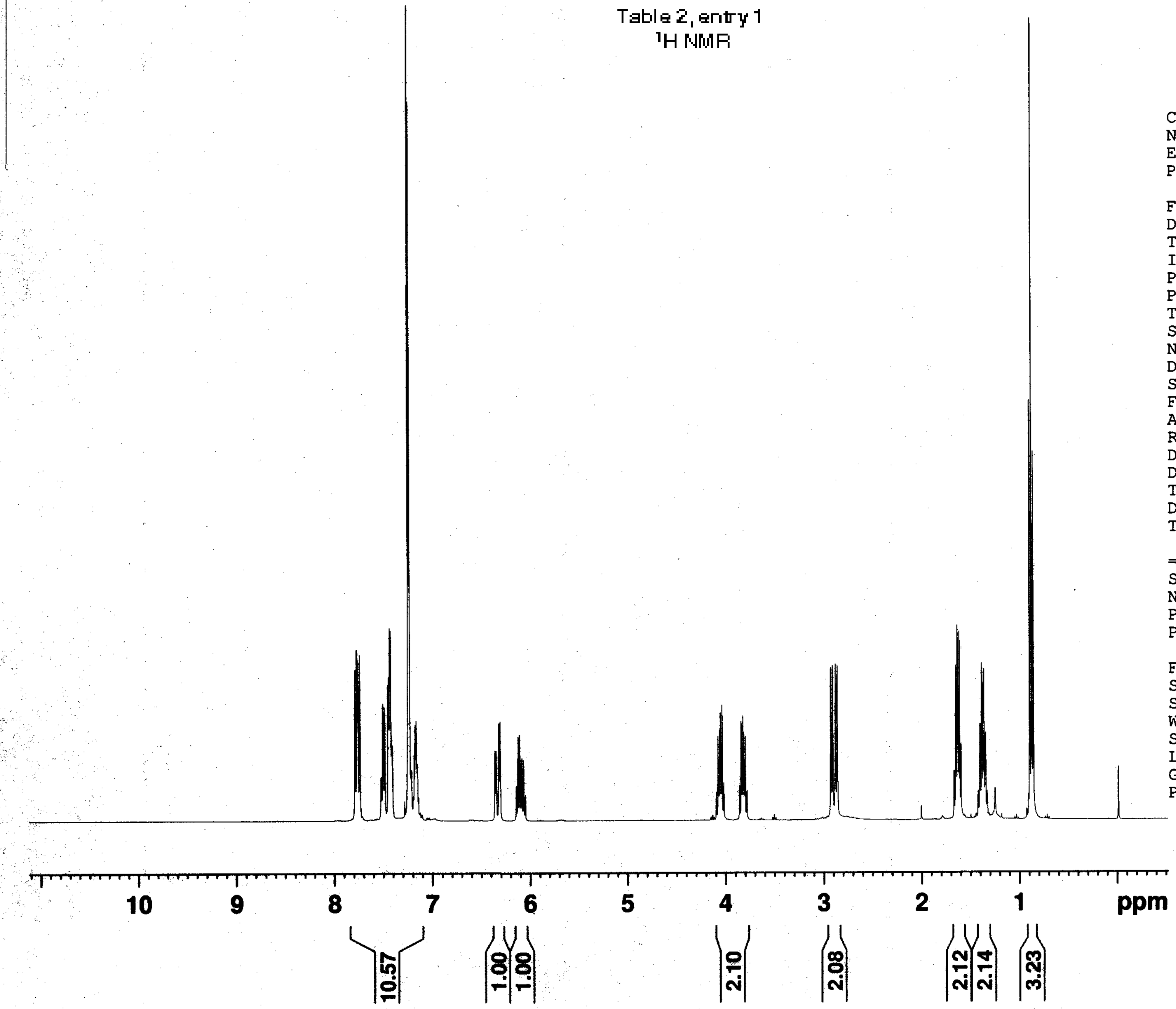


```
Current Data Parameters
NAME              AFL001NEW
EXPNO                     3
PROCNO                    1

F2 - Acquisition Parameters
Date_              20150618
Time                  10.18
INSTRUM               spect
PROBHD     5 mm PABBO BB/
PULPROG                zg30
TD                    65536
SOLVENT               CDCl3
NS                       16
DS                        2
SWH                8012.820 Hz
FIDRES             0.122266 Hz
AQ                4.0894465 sec
RG                    16.39
DW                   62.400 usec
DE                     6.50 usec
TE                    294.1 K
D1               1.00000000 sec
TD0                       1

======== CHANNEL f1 ========
SFO1            400.1324710 MHz
NUC1                     1H
P1                    10.00 usec
PLW1            25.00300026 W

F2 - Processing parameters
SI                    65536
SF              400.1300000 MHz
WDW                      EM
SSB      0
LB                     0.30 Hz
GB       0
PC                     1.00
```

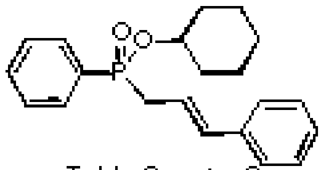

Table 2, entry 2
^{31}P/^{1}H NMR decoupled

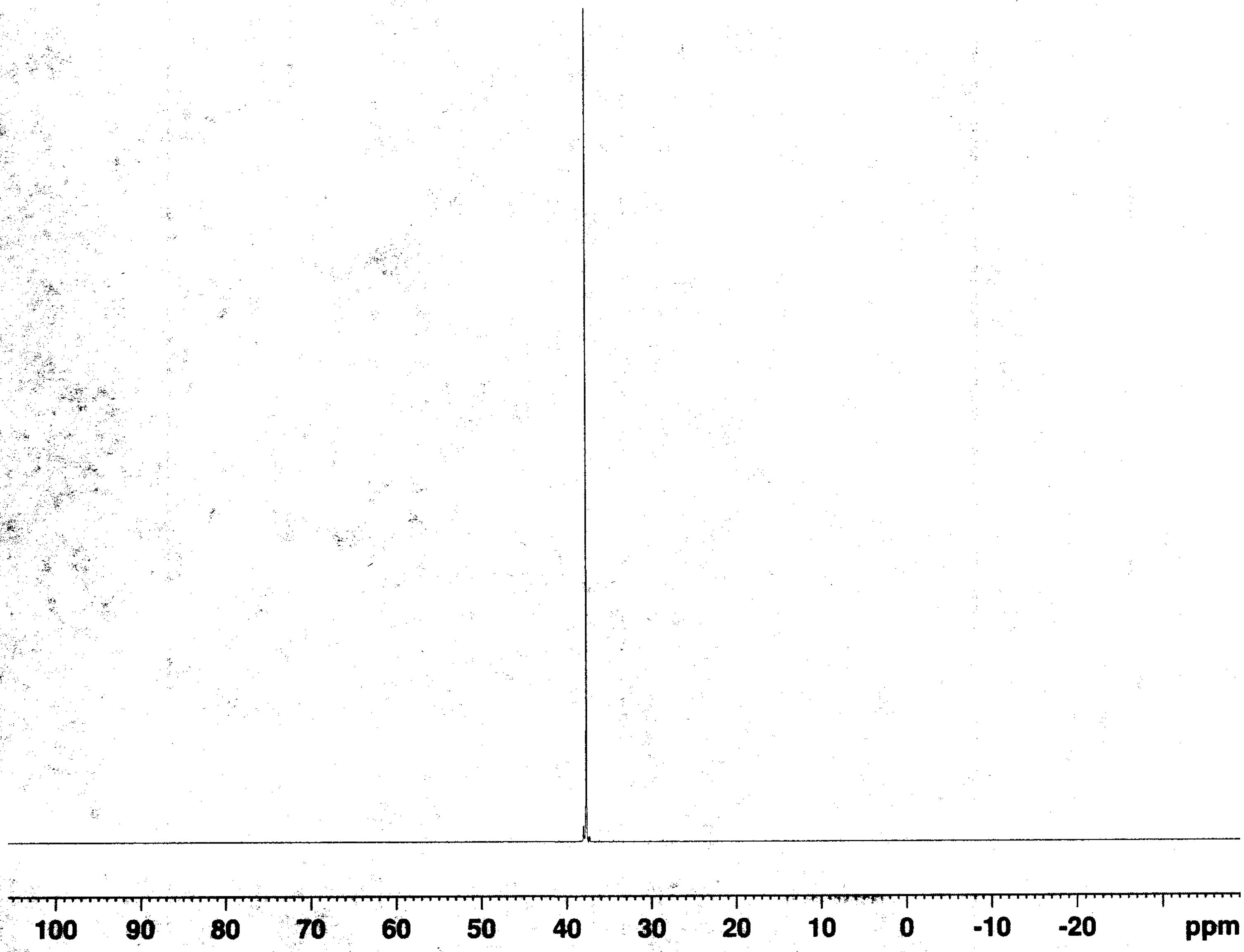


```
Current Data Parameters
NAME              AFL041
EXPNO                  3
PROCNO                 1

F2 - Acquisition Parameters
Date_           20150716
Time                9.02
INSTRUM            spect
PROBHD   5 mm PABBO BB/
PULPROG          zgpg30
TD                65536
SOLVENT          Acetone
NS                    16
DS                     4
SWH           64102.563 Hz
FIDRES         0.978127 Hz
AQ            0.5111808 sec
RG               203.57
DW                7.800 usec
DE                 6.50 usec
TE                294.4 K
D1           2.00000000 sec
D11          0.03000000 sec
TD0                    1

======== CHANNEL f1 ========
SFO1        161.9674942 MHz
NUC1                31P
P1                14.25 usec
PLW1        15.00000000 W

======== CHANNEL f2 ========
SFO2        400.1316005 MHz
NUC2                 1H
CPDPRG[2        waltz16
PCPD2             90.00 usec
PLW2        10.00000000 W
PLW12        0.31604999 W
PLW13        0.25600001 W

F2 - Processing parameters
SI                32768
SF          161.9755930 MHz
WDW                  EM
SSB      0
LB                 1.00 Hz
GB       0
PC                 1.40
```

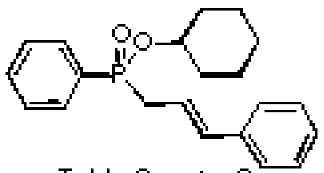

Table 2, entry 2
$^{31}P/^{1}H$ NMR coupled

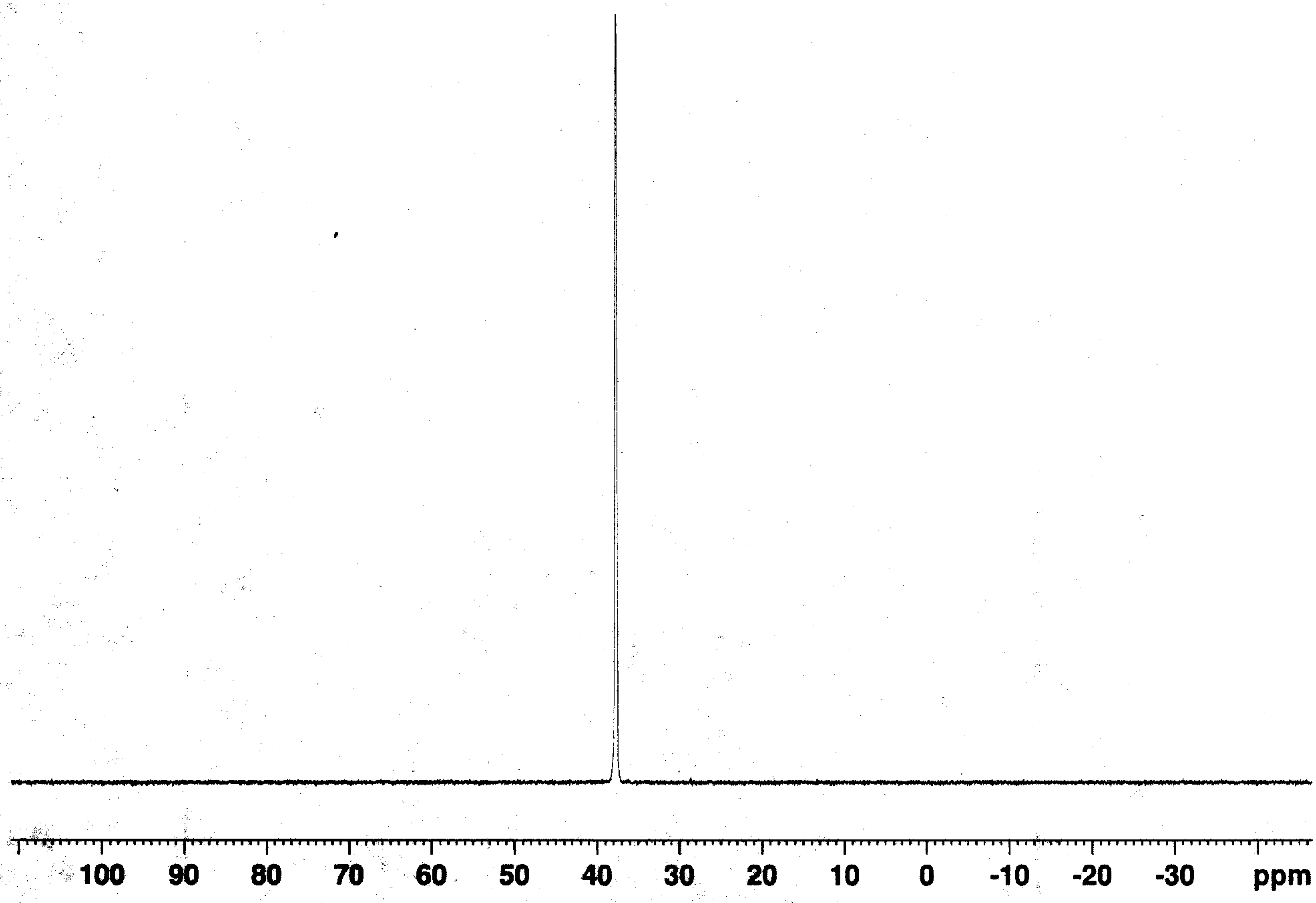


```
Current Data Parameters
NAME                    5
EXPNO                   4
PROCNO                  1

F2 - Acquisition Parameters
Date_            20150716
Time                 9.04
INSTRUM             spect
PROBHD   5 mm PABBO BB/
PULPROG              zg30
TD                  65536
SOLVENT           Acetone
NS                     19
DS                      4
SWH             64102.563 Hz
FIDRES           0.978127 Hz
AQ              0.5111808 sec
RG                 203.57
DW                  7.800 usec
DE                   6.50 usec
TE                  294.0 K
D1             2.00000000 sec
TD0                     1

======== CHANNEL f1 ========
SFO1          161.9674942 MHz
NUC1                  31P
P1                  14.25 usec
PLW1          15.00000000 W

F2 - Processing parameters
SI                  32768
SF            161.9755930 MHz
WDW                    EM
SSB      0
LB                   1.00 Hz
GB       0
PC                   1.40
```

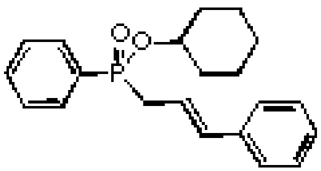

Table 2, entry 2
^{1}H NMR

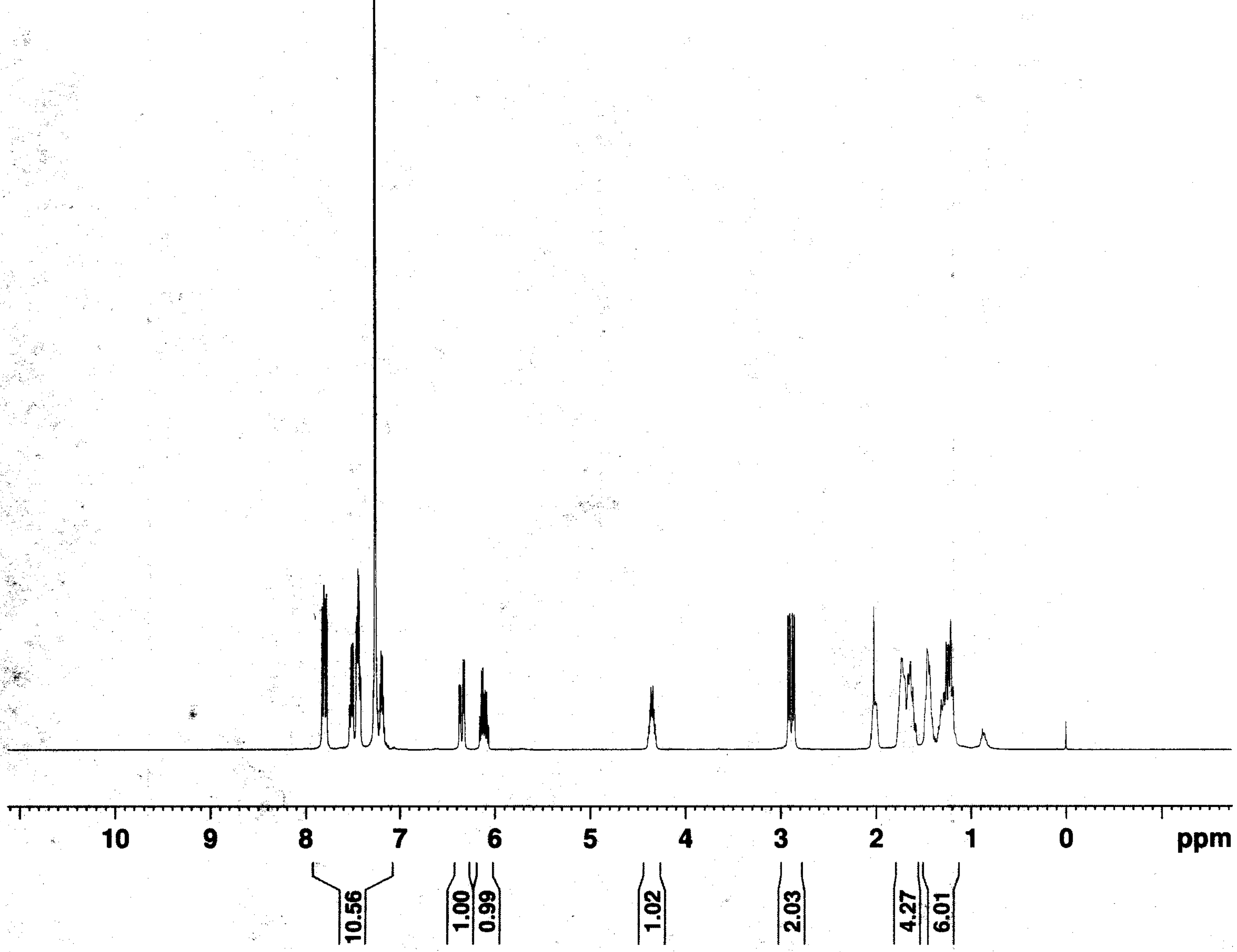


```
Current Data Parameters
NAME                 AFL041
EXPNO                     5
PROCNO                    1

F2 - Acquisition Parameters
Date_              20150716
Time                   9.09
INSTRUM               spect
PROBHD   5 mm PABBO BB/
PULPROG                zg30
TD                    65536
SOLVENT               CDCl3
NS                       16
DS                        2
SWH            8012.820 Hz
FIDRES         0.122266 Hz
AQ            4.0894465 sec
RG                    16.39
DW             62.400 usec
DE               6.50 usec
TE                  293.9 K
D1           1.00000000 sec
TD0                       1

======== CHANNEL f1 ========
SFO1        400.1324710 MHz
NUC1                     1H
P1               10.00 usec
PLW1        25.00300026 W

F2 - Processing parameters
SI                    65536
SF          400.1300000 MHz
WDW                      EM
SSB      0
LB                  0.30 Hz
GB       0
PC                     1.00
```

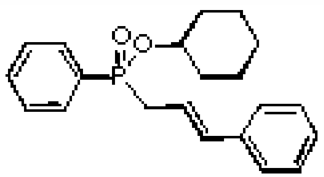

Table 2, entry 2
^{13}C NMR

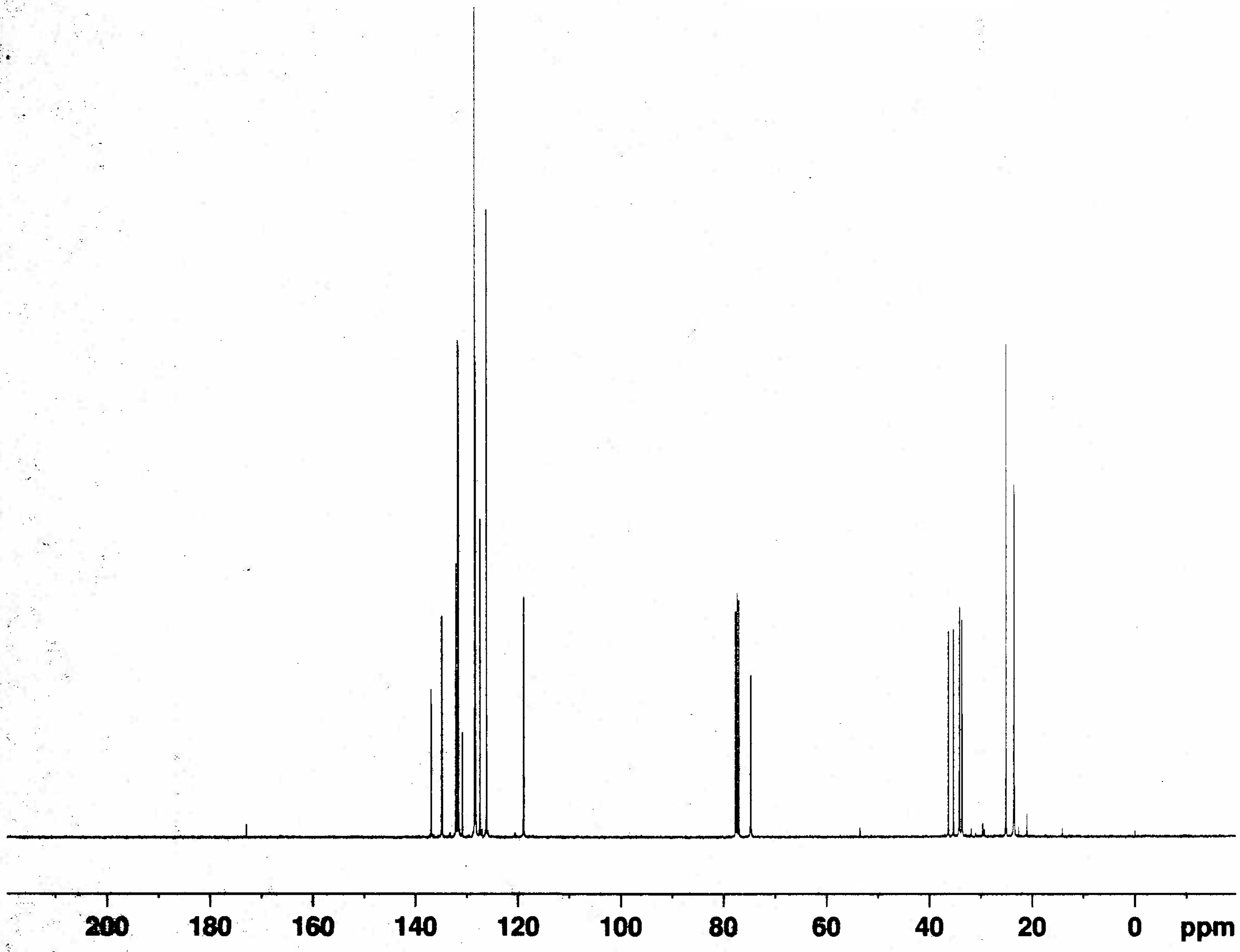


```
Current Data Parameters
NAME              AFL041
EXPNO                  6
PROCNO                 1

F2 - Acquisition Parameters
Date_           20150727
Time               18.21
INSTRUM            spect
PROBHD   5 mm PABBO BB/
PULPROG          zgpg30
TD                 65536
SOLVENT            CDCl3
NS                   676
DS                     4
SWH            24038.461 Hz
FIDRES          0.366798 Hz
AQ             1.3631488 sec
RG                203.57
DW                20.800 usec
DE                  6.50 usec
TE                 296.2 K
D1            2.00000000 sec
D11           0.03000000 sec
TD0                    1

======== CHANNEL f1 ========
SFO1         100.6228293 MHz
NUC1                 13C
P1                 10.00 usec
PLW1         45.00000000 W

======== CHANNEL f2 ========
SFO2         400.1316005 MHz
NUC2                  1H
CPDPRG[2         waltz16
PCPD2              90.00 usec
PLW2         10.00000000 W
PLW12         0.31604999 W
PLW13         0.25600001 W

F2 - Processing parameters
SI                 32768
SF           100.6127685 MHz
WDW                   EM
SSB       0
LB                  1.00 Hz
GB        0
PC                  1.40
```

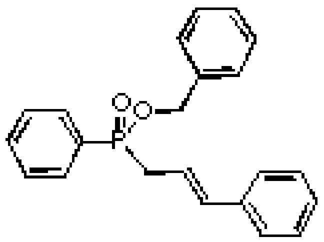

Table 2, entry 3

$^{31}P/^{1}H$ NMR decoupled

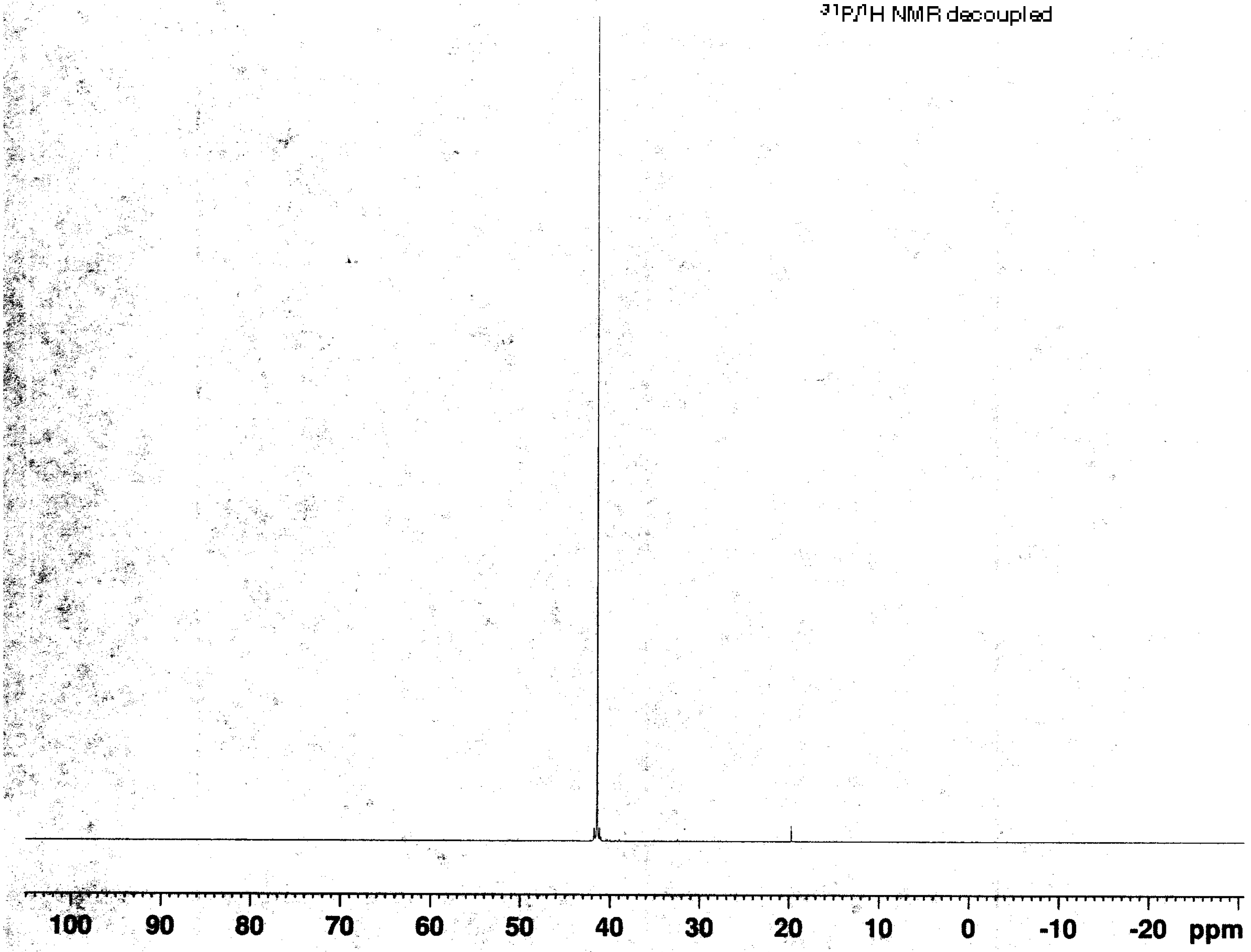


```
Current Data Parameters
NAME              AFL046
EXPNO                  3
PROCNO                 1

F2 - Acquisition Parameters
Date_           20150720
Time               14.17
INSTRUM            spect
PROBHD    5 mm PABBO BB/
PULPROG          zgpg30
TD                 65536
SOLVENT            CDCl3
NS                    16
DS                     4
SWH            64102.563 Hz
FIDRES          0.978127 Hz
AQ             0.5111808 sec
RG                203.57
DW                 7.800 usec
DE                  6.50 usec
TE                 295.7 K
D1            2.00000000 sec
D11           0.03000000 sec
TD0                    1

======== CHANNEL f1 ========
SFO1         161.9674942 MHz
NUC1                 31P
P1                 14.25 usec
PLW1         15.00000000 W

======== CHANNEL f2 ========
SFO2         400.1316005 MHz
NUC2                  1H
CPDPRG[2         waltz16
PCPD2              90.00 usec
PLW2         10.00000000 W
PLW12         0.31604999 W
PLW13         0.25600001 W

F2 - Processing parameters
SI                 32768
SF           161.9755930 MHz
WDW                   EM
SSB       0
LB                  1.00 Hz
GB        0
PC                  1.40
```

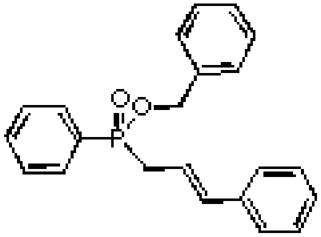

Table 2, entry 3
^{31}P/^{1}H NMR coupled

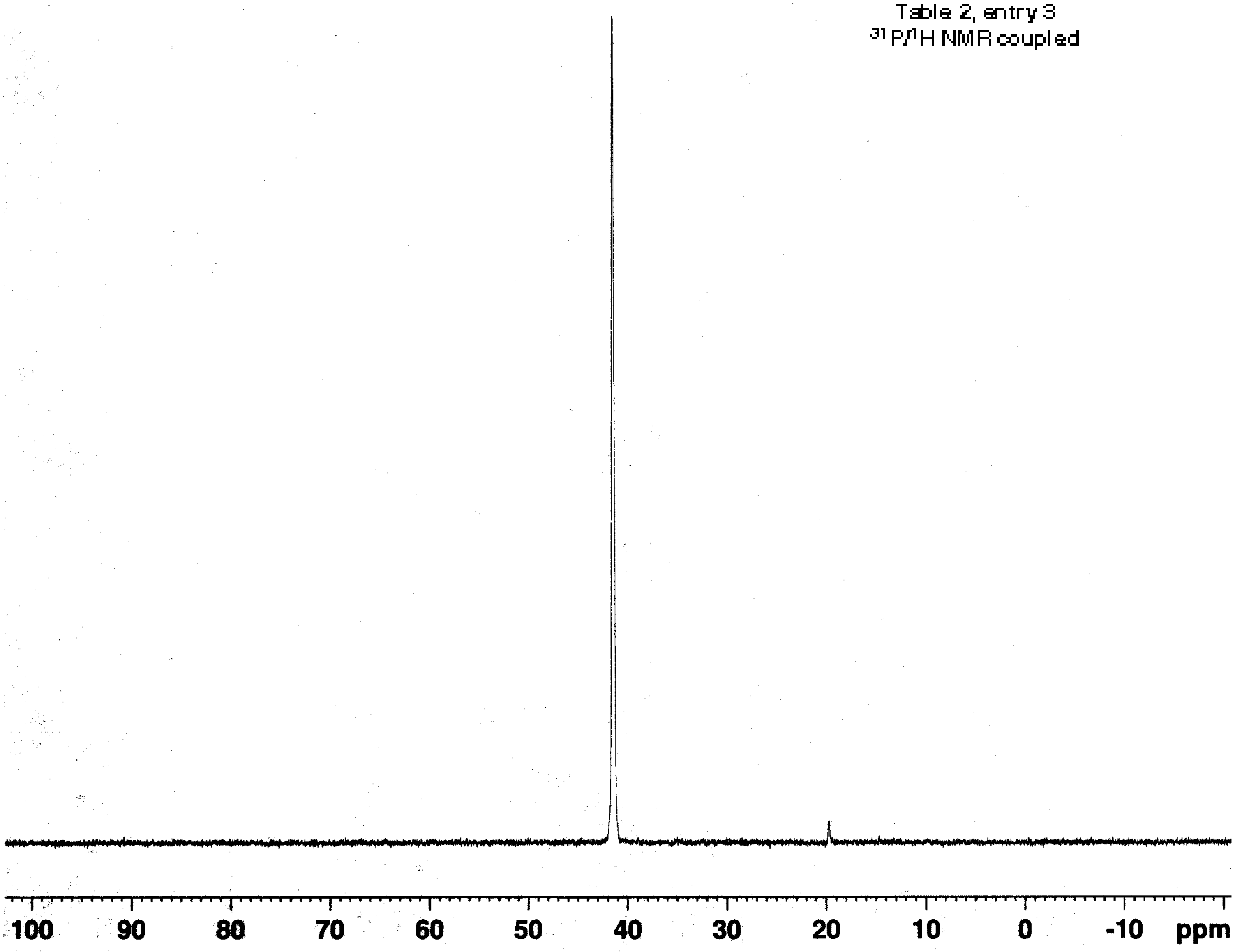


```
Current Data Parameters
NAME              AFL046
EXPNO                  4
PROCNO                 1

F2 - Acquisition Parameters
Date_           20150720
Time               14.19
INSTRUM            spect
PROBHD   5 mm PABBO BB/
PULPROG             zg30
TD                 65536
SOLVENT            CDCl3
NS                    16
DS                     4
SWH            64102.563 Hz
FIDRES          0.978127 Hz
AQ             0.5111808 sec
RG                203.57
DW                 7.800 usec
DE                  6.50 usec
TE                 295.2 K
D1            2.00000000 sec
TD0                    1

======== CHANNEL f1 ========
SFO1         161.9674942 MHz
NUC1                 31P
P1                 14.25 usec
PLW1         15.00000000 W

F2 - Processing parameters
SI                 32768
SF           161.9755930 MHz
WDW                   EM
SSB      0
LB                  1.00 Hz
GB       0
PC                  1.40
```

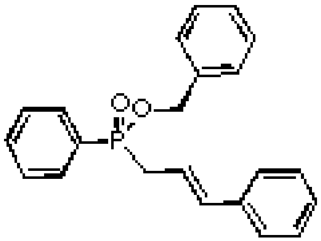

Table 2, entry 3
^1H NMR

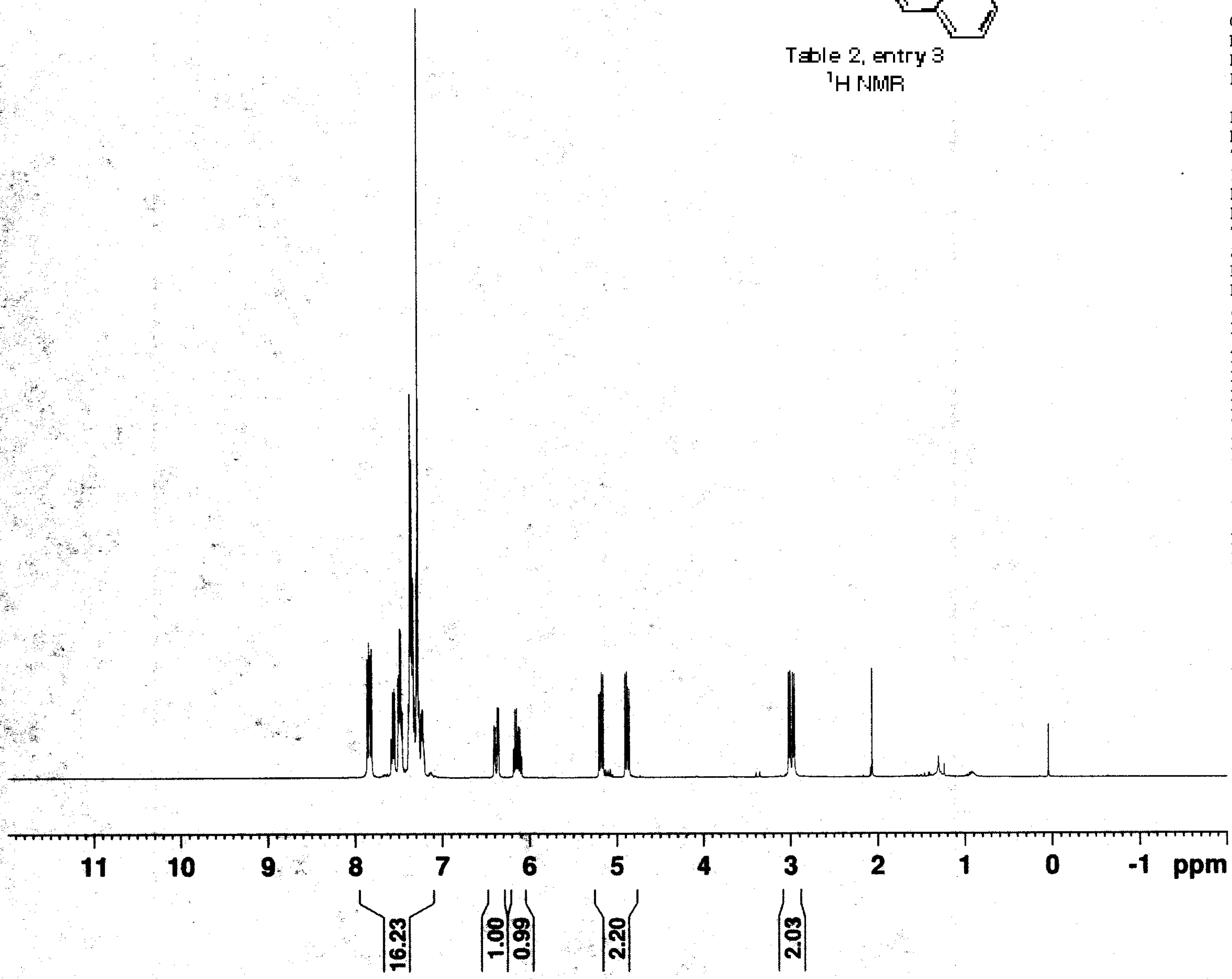


```
Current Data Parameters
NAME                AFL046
EXPNO                    5
PROCNO                   1

F2 - Acquisition Parameters
Date_             20150720
Time                 14.25
INSTRUM              spect
PROBHD   5 mm PABBO BB/
PULPROG               zg30
TD                   65536
SOLVENT              CDCl3
NS                      13
DS                       2
SWH               8012.820 Hz
FIDRES            0.122266 Hz
AQ               4.0894465 sec
RG                   32.38
DW                  62.400 usec
DE                    6.50 usec
TE                   295.2 K
D1              1.00000000 sec
TD0                      1

======== CHANNEL f1 ========
SFO1          400.1324710 MHz
NUC1                    1H
P1                   10.00 usec
PLW1          25.00300026 W

F2 - Processing parameters
SI                   65536
SF            400.1300000 MHz
WDW                     EM
SSB      0
LB                    0.30 Hz
GB       0
PC                    1.00
```

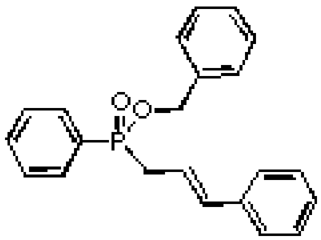

Table 2, entry 3
^{13}C NMR

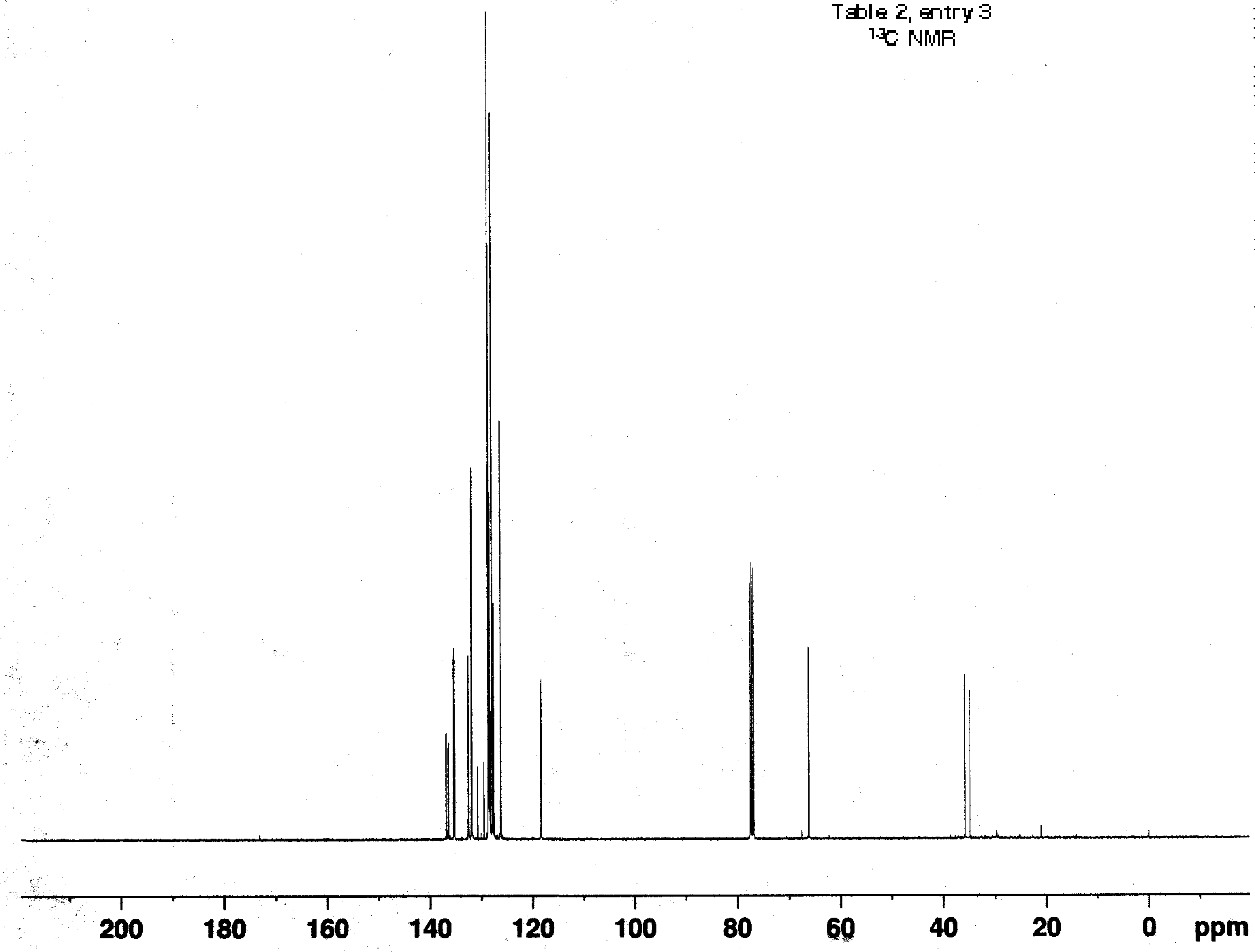


```
Current Data Parameters
NAME                AFL046
EXPNO                    6
PROCNO                   1

F2 - Acquisition Parameters
Date_             20150725
Time                 17.50
INSTRUM              spect
PROBHD     5 mm PABBO BB/
PULPROG            zgpg30
TD                  65536
SOLVENT             CDCl3
NS                   1024
DS                      4
SWH             24038.461 Hz
FIDRES           0.366798 Hz
AQ              1.3631488 sec
RG                 203.57
DW                 20.800 usec
DE                   6.50 usec
TE                  295.9 K
D1             2.00000000 sec
D11            0.03000000 sec
TD0                     1

======== CHANNEL f1 ========
SFO1         100.6228293 MHz
NUC1                 13C
P1                 10.00 usec
PLW1         45.00000000 W

======== CHANNEL f2 ========
SFO2         400.1316005 MHz
NUC2                  1H
CPDPRG[2         waltz16
PCPD2              90.00 usec
PLW2         10.00000000 W
PLW12         0.31604999 W
PLW13         0.25600001 W

F2 - Processing parameters
SI                 32768
SF           100.6127685 MHz
WDW                   EM
SSB      0
LB                  1.00 Hz
GB       0
PC                  1.40
```

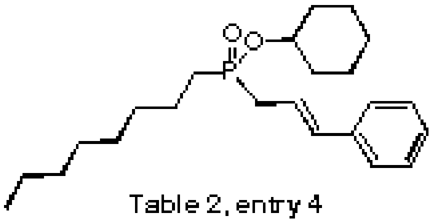

Table 2, entry 4

$^{31}P/^{1}H$ NMR decoupled

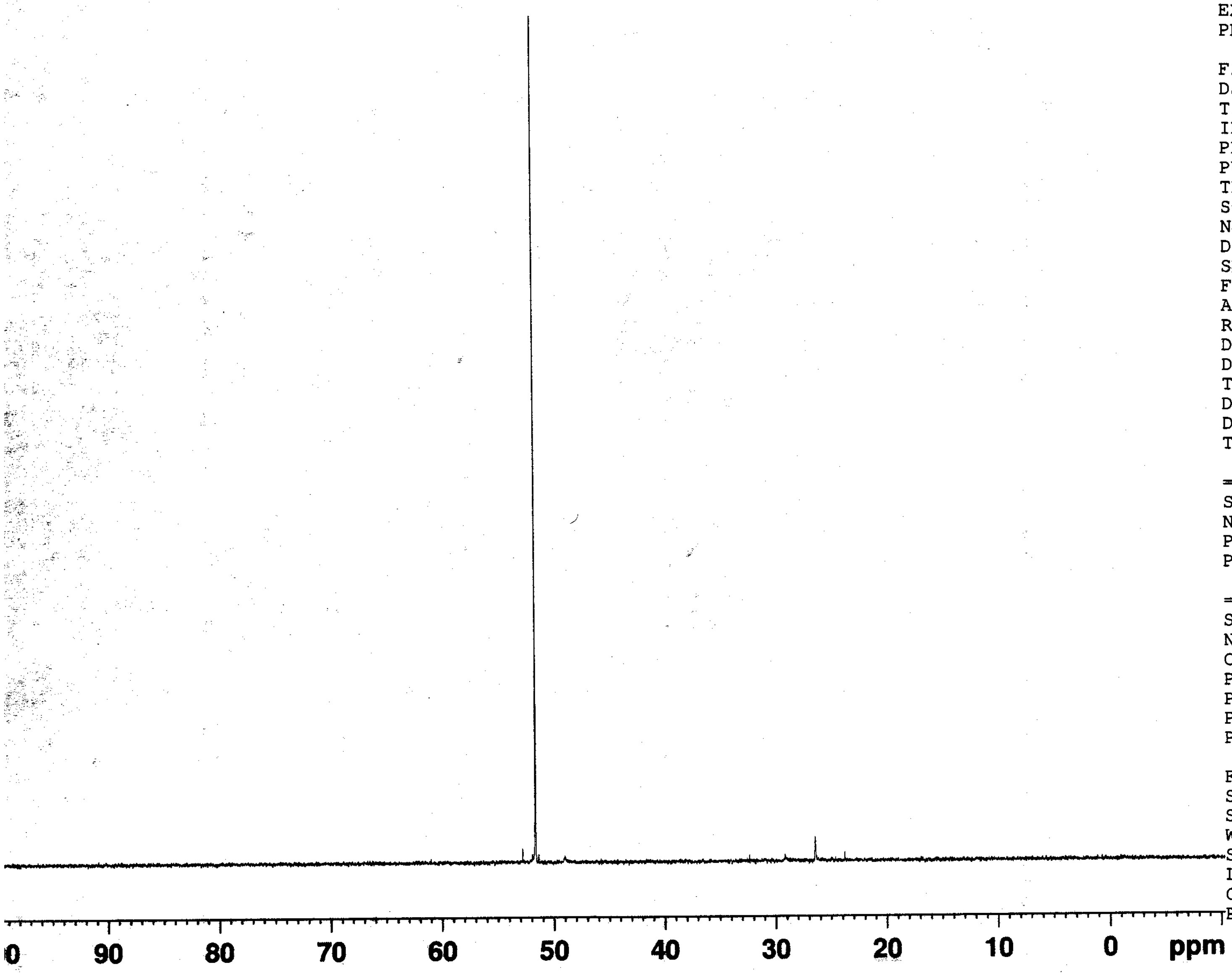

Current Data Parameters
NAME AFL002ter
EXPNO 1
PROCNO 1

F2 - Acquisition Parameters

Date_	20150609
Time	9.00
INSTRUM	spect
PROBHD	5 mm PABBO BB/
PULPROG	zgpg30
TD	65536
SOLVENT	DMSO
NS	16
DS	4
SWH	64102.563 Hz
FIDRES	0.978127 Hz
AQ	0.5111808 sec
RG	203.57
DW	7.800 usec
DE	6.50 usec
TE	294.5 K
D1	2.00000000 sec
D11	0.03000000 sec
TD0	1

======== CHANNEL f1 ========

SFO1	161.9674942 MHz
NUC1	31P
P1	14.25 usec
PLW1	15.00000000 W

======== CHANNEL f2 ========

SFO2	400.1316005 MHz
NUC2	1H
CPDPRG[2	waltz16
PCPD2	90.00 usec
PLW2	10.00000000 W
PLW12	0.31604999 W
PLW13	0.25600001 W

F2 - Processing parameters

SI	32768
SF	161.9755930 MHz
WDW	EM
SSB	0
LB	1.00 Hz
GB	0
PC	1.40

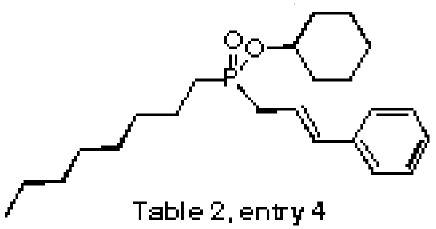

Table 2, entry 4
$^{31}P/^{1}H$ NMR coupled

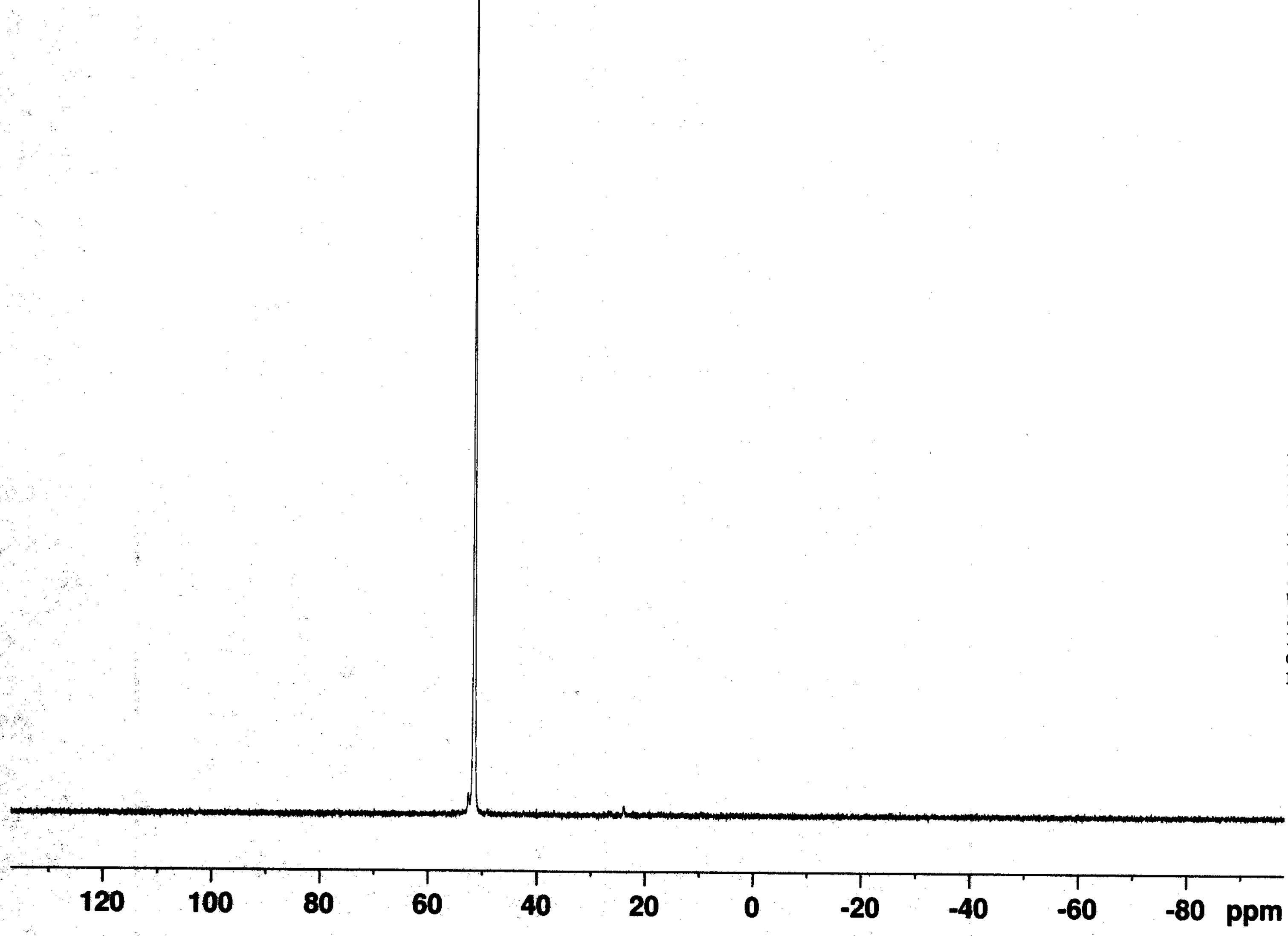


```
Current Data Parameters
NAME             AFL002NEW
EXPNO                    1
PROCNO                   1

F2 - Acquisition Parameters
Date_             20150617
Time                 11.32
INSTRUM              spect
PROBHD    5 mm PABBO BB/
PULPROG               zg30
TD                   65536
SOLVENT            Acetone
NS                      21
DS                       4
SWH              64102.563 Hz
FIDRES            0.978127 Hz
AQ               0.5111808 sec
RG                  203.57
DW                   7.800 usec
DE                    6.50 usec
TE                   294.2 K
D1              2.00000000 sec
TD0                      1

======== CHANNEL f1 ========
SFO1           161.9674942 MHz
NUC1                   31P
P1                   14.25 usec
PLW1           15.00000000 W

F2 - Processing parameters
SI                   32768
SF             161.9755930 MHz
WDW                     EM
SSB        0
LB                    1.00 Hz
GB         0
PC                    1.40
```

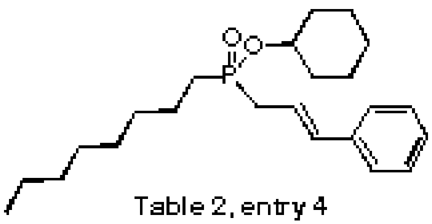

Table 2, entry 4
^{1}H NMR

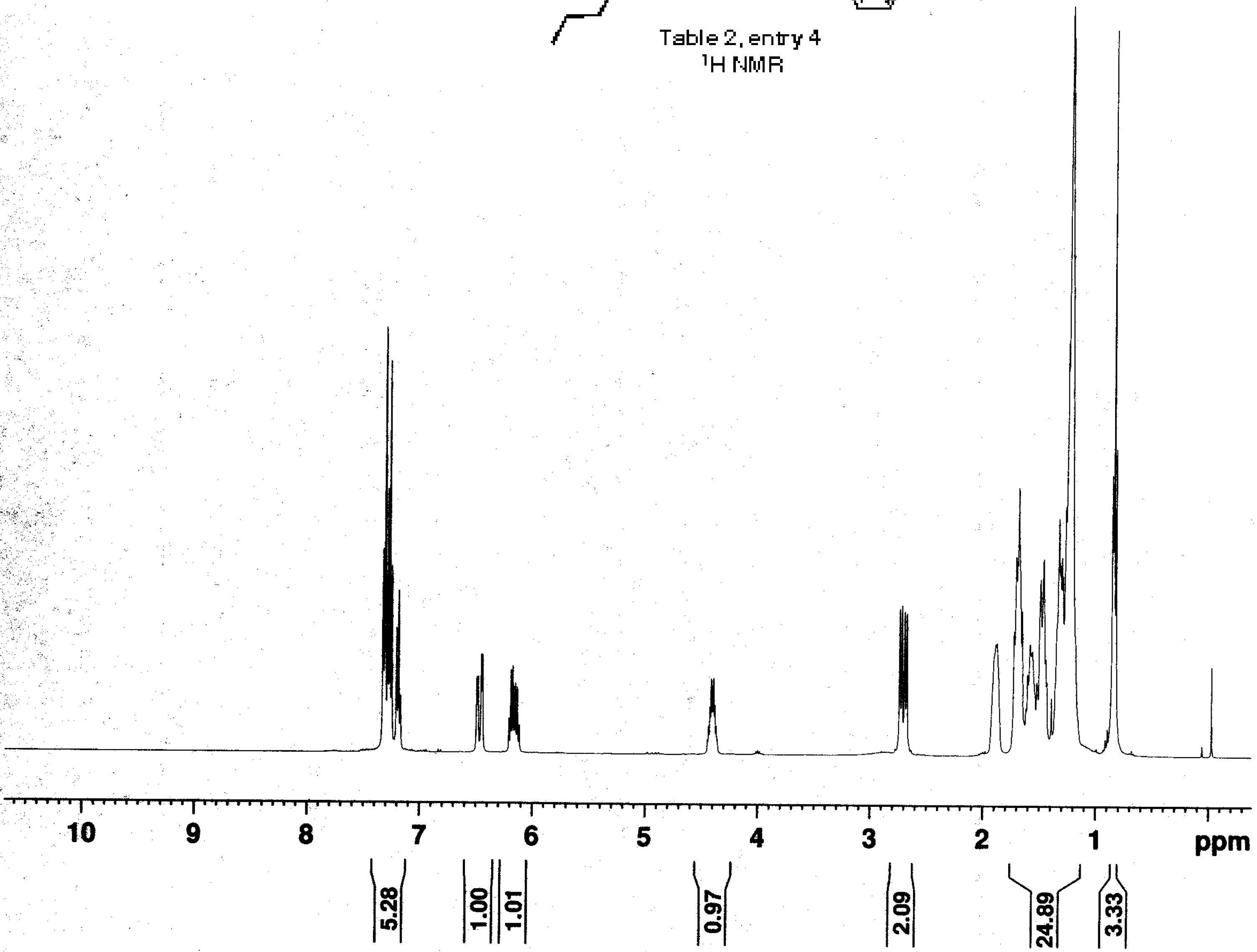

Current Data Parameters
NAME AFL002NEW
EXPNO 2
PROCNO 1

F2 - Acquisition Parameters
Date_ 20150617
Time 11.38
INSTRUM spect
PROBHD 5 mm PABBO BB/
PULPROG zg30
TD 65536
SOLVENT CDCl3
NS 16
DS 2
SWH 8012.820 Hz
FIDRES 0.122266 Hz
AQ 4.0894465 sec
RG 11.05
DW 62.400 usec
DE 6.50 usec
TE 294.1 K
D1 1.00000000 sec
TD0 1

======== CHANNEL f1 ========
SFO1 400.1324710 MHz
NUC1 1H
P1 10.00 usec
PLW1 25.00300026 W

F2 - Processing parameters
SI 65536
SF 400.1300000 MHz
WDW EM
SSB 0
LB 0.30 Hz
GB 0
PC 1.00

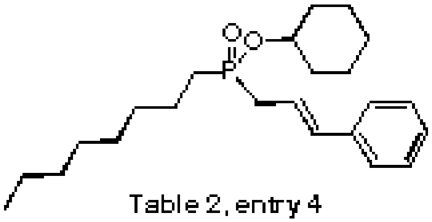

Table 2, entry 4
^{13}C NMR

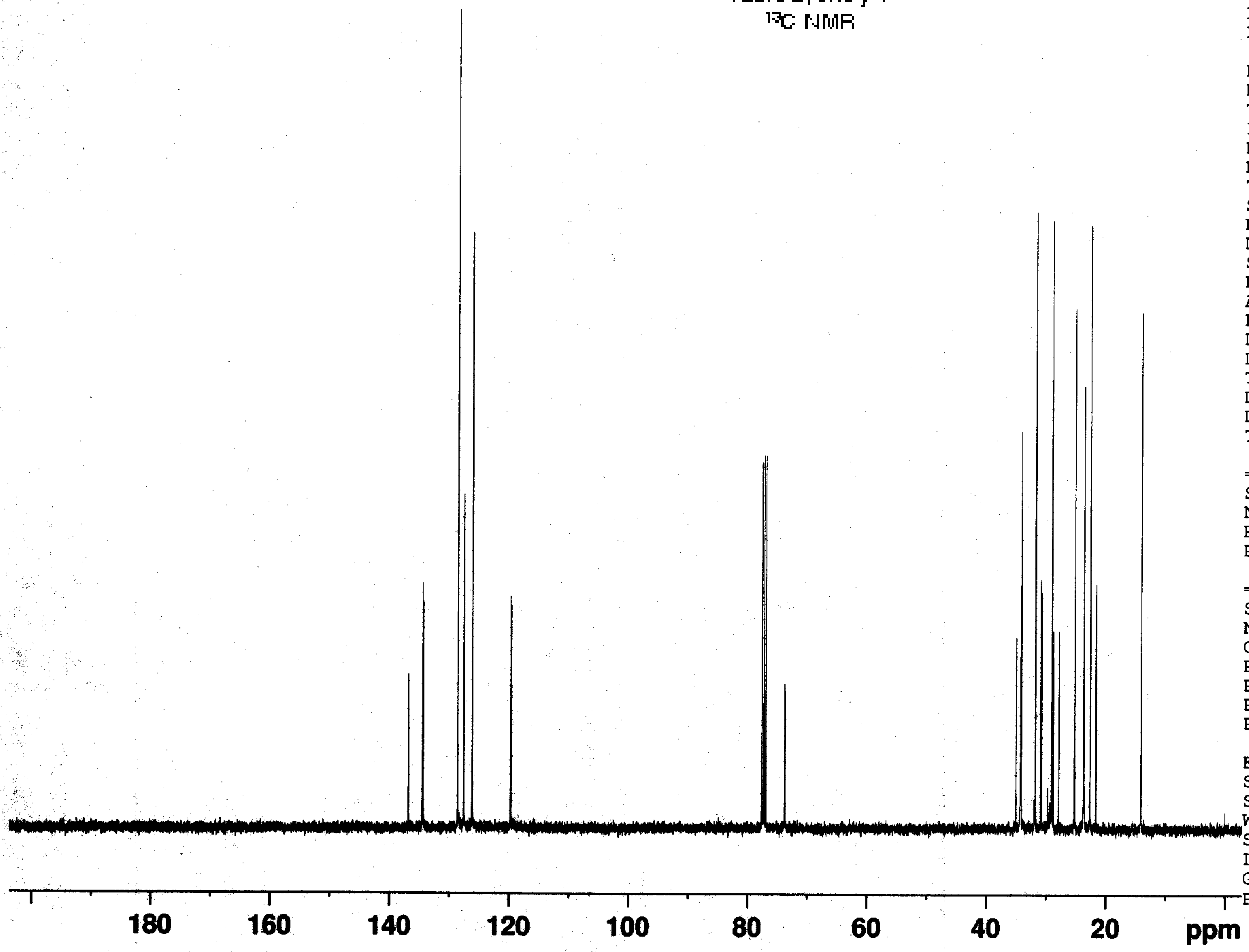


```
Current Data Parameters
NAME            AFL002NEW
EXPNO                   3
PROCNO                  1

F2 - Acquisition Parameters
Date_            20150617
Time                11.49
INSTRUM             spect
PROBHD   5 mm PABBO BB/
PULPROG           zgpg30
TD                 65536
SOLVENT            CDCl3
NS                    56
DS                     4
SWH            24038.461 Hz
FIDRES          0.366798 Hz
AQ             1.3631488 sec
RG                203.57
DW                20.800 usec
DE                  6.50 usec
TE                 294.8 K
D1            2.00000000 sec
D11           0.03000000 sec
TD0                    1

======== CHANNEL f1 ========
SFO1         100.6228293 MHz
NUC1                 13C
P1                 10.00 usec
PLW1         45.00000000 W

======== CHANNEL f2 ========
SFO2         400.1316005 MHz
NUC2                  1H
CPDPRG[2         waltz16
PCPD2              90.00 usec
PLW2         10.00000000 W
PLW12         0.31604999 W
PLW13         0.25600001 W

F2 - Processing parameters
SI                 32768
SF           100.6127685 MHz
WDW                   EM
SSB      0
LB                  1.00 Hz
GB       0
PC                  1.40
```

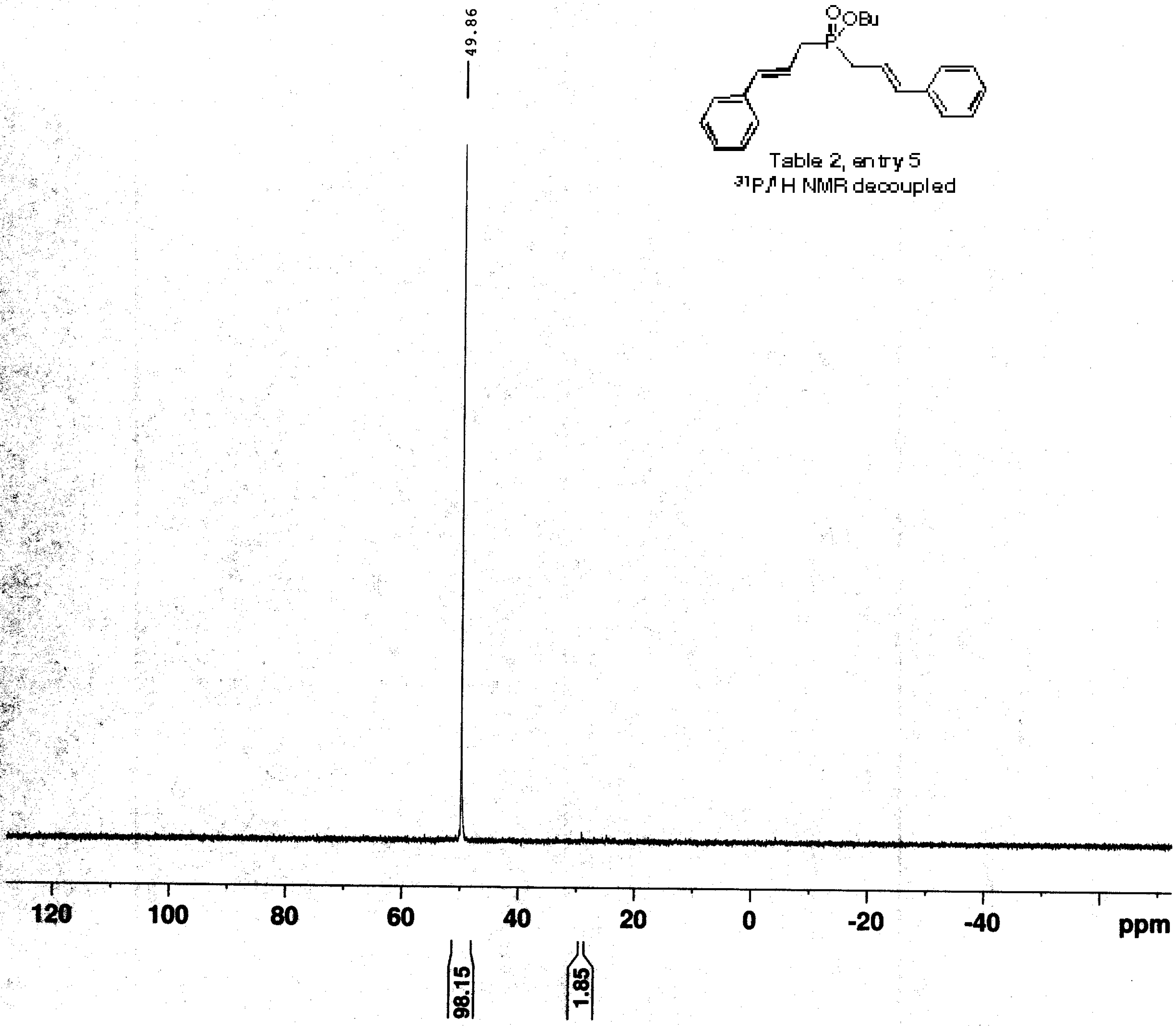

Table 2, entry 5
$^{31}P/^{1}H$ NMR decoupled

Current Data Parameters
NAME AFL003
EXPNO 1
PROCNO 1

F2 - Acquisition Parameters
Date_ 20150604
Time 14.15
INSTRUM spect
PROBHD 5 mm PABBO BB/
PULPROG zgpg30
TD 65536
SOLVENT CDCl3
NS 16
DS 4
SWH 64102.563 Hz
FIDRES 0.978127 Hz
AQ 0.5111808 sec
RG 203.57
DW 7.800 usec
DE 6.50 usec
TE 294.7 K
D1 2.00000000 sec
D11 0.03000000 sec
TD0 1

======== CHANNEL f1 ========
SFO1 161.9674942 MHz
NUC1 31P
P1 14.25 usec
PLW1 15.00000000 W

======== CHANNEL f2 ========
SFO2 400.1316005 MHz
NUC2 1H
CPDPRG[2 waltz16
PCPD2 90.00 usec
PLW2 10.00000000 W
PLW12 0.31604999 W
PLW13 0.25600001 W

F2 - Processing parameters
SI 32768
SF 161.9755930 MHz
WDW EM
SSB 0
LB 1.00 Hz
GB 0
PC 1.40

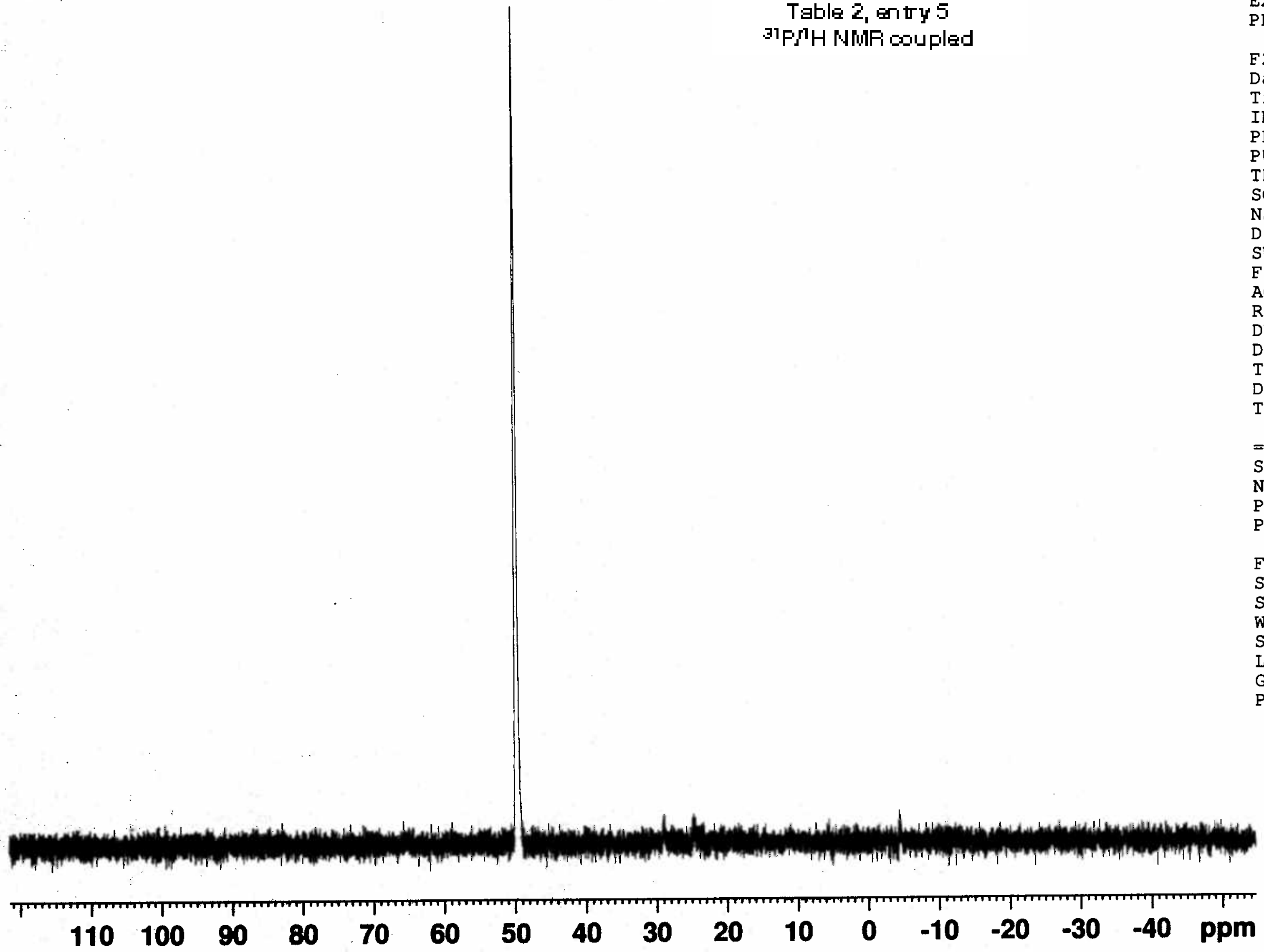

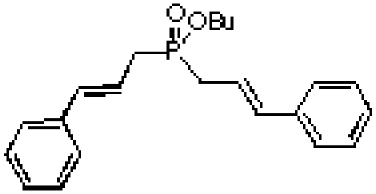

Table 2, entry 5
$^{31}P/^{1}H$ NMR coupled

```
Current Data Parameters
NAME                AFL003
EXPNO                    2
PROCNO                   1

F2 - Acquisition Parameters
Date_             20150604
Time                 14.17
INSTRUM              spect
PROBHD   5 mm PABBO BB/
PULPROG               zg30
TD                   65536
SOLVENT              CDCl3
NS                      32
DS                       4
SWH             64102.563 Hz
FIDRES           0.978127 Hz
AQ              0.5111808 sec
RG                  203.57
DW                   7.800 usec
DE                    6.50 usec
TE                   294.2 K
D1              2.00000000 sec
TD0                      1

======== CHANNEL f1 ========
SFO1          161.9674942 MHz
NUC1                   31P
P1                   14.25 usec
PLW1           15.00000000 W

F2 - Processing parameters
SI                   32768
SF            161.9755930 MHz
WDW                     EM
SSB          0
LB                    1.00 Hz
GB           0
PC                    1.40
```

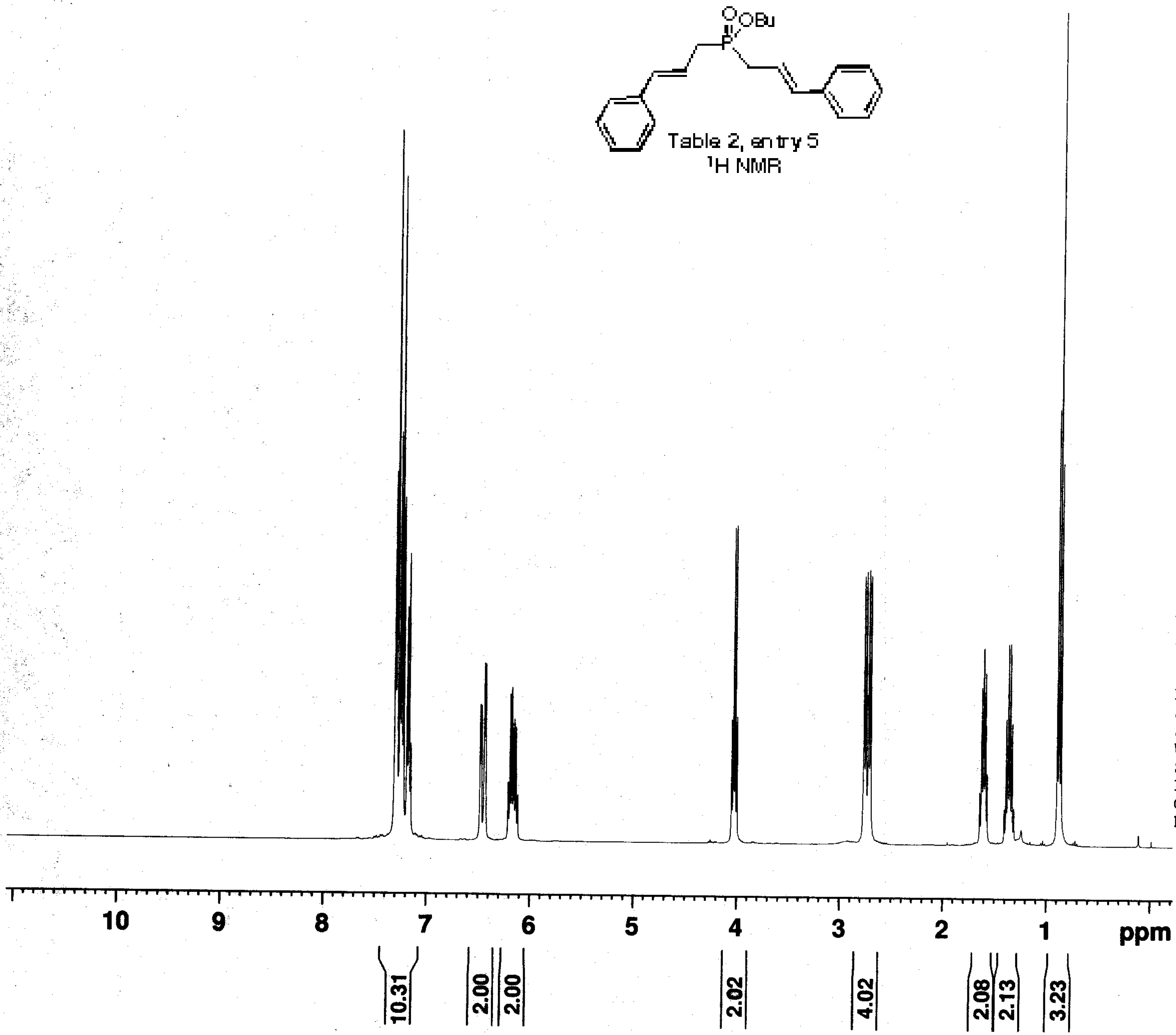

Current Data Parameters
NAME AFL003rab
EXPNO 4
PROCNO 1

F2 - Acquisition Parameters
Date_ 20150819
Time 10.24
INSTRUM spect
PROBHD 5 mm PABBO BB/
PULPROG zg30
TD 65536
SOLVENT CDCl3
NS 13
DS 2
SWH 8012.820 Hz
FIDRES 0.122266 Hz
AQ 4.0894465 sec
RG 10.22
DW 62.400 usec
DE 6.50 usec
TE 294.9 K
D1 1.00000000 sec
TD0 1

======== CHANNEL f1 ========
SFO1 400.1324710 MHz
NUC1 1H
P1 10.00 usec
PLW1 25.00300026 W

F2 - Processing parameters
SI 65536
SF 400.1300000 MHz
WDW EM
SSB 0
LB 0.30 Hz
GB 0
PC 1.00

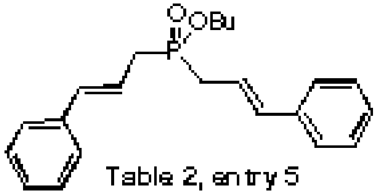

Table 2, entry 5

^{13}C NMR

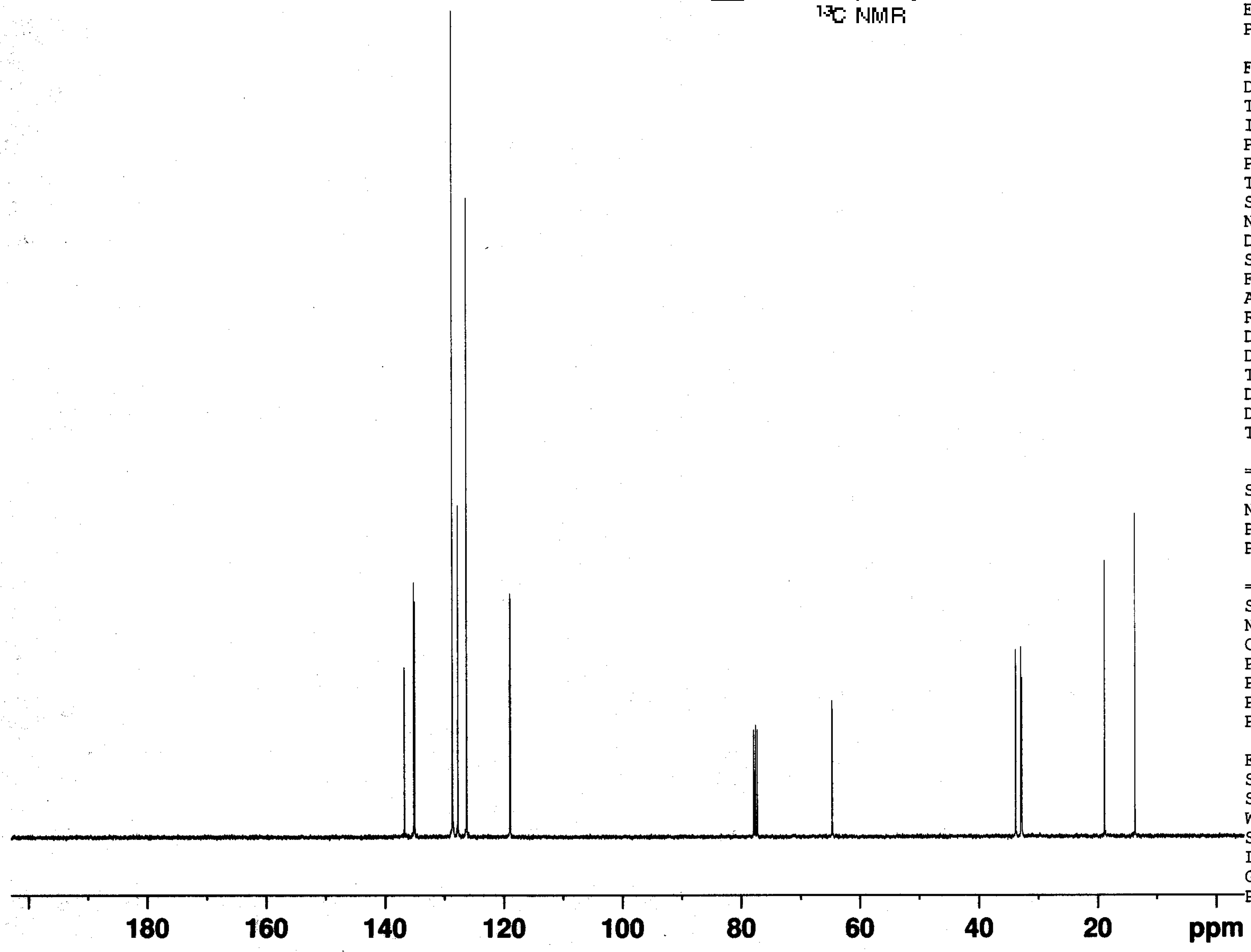


```
Current Data Parameters
NAME              AFL003rab
EXPNO                     5
PROCNO                    1

F2 - Acquisition Parameters
Date_              20150819
Time                  10.33
INSTRUM               spect
PROBHD    5 mm PABBO BB/
PULPROG             zgpg30
TD                    65536
SOLVENT               CDC13
NS                       41
DS                        4
SWH               24038.461 Hz
FIDRES             0.366798 Hz
AQ                1.3631488 sec
RG                   203.57
DW                   20.800 usec
DE                     6.50 usec
TE                    295.6 K
D1               2.00000000 sec
D11              0.03000000 sec
TD0                       1

======== CHANNEL f1 ========
SFO1           100.6228293 MHz
NUC1                    13C
P1                    10.00 usec
PLW1           45.00000000 W

======== CHANNEL f2 ========
SFO2           400.1316005 MHz
NUC2                     1H
CPDPRG[2           waltz16
PCPD2                 90.00 usec
PLW2           10.00000000 W
PLW12           0.31604999 W
PLW13           0.25600001 W

F2 - Processing parameters
SI                    32768
SF             100.6127685 MHz
WDW                      EM
SSB       0
LB                     1.00 Hz
GB        0
PC                     1.40
```

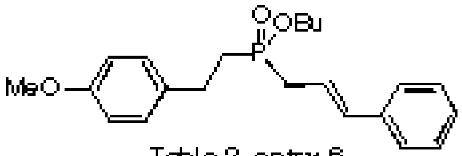

Table 2, entry 6

$^{31}P/^{1}H$ NMR decoupled

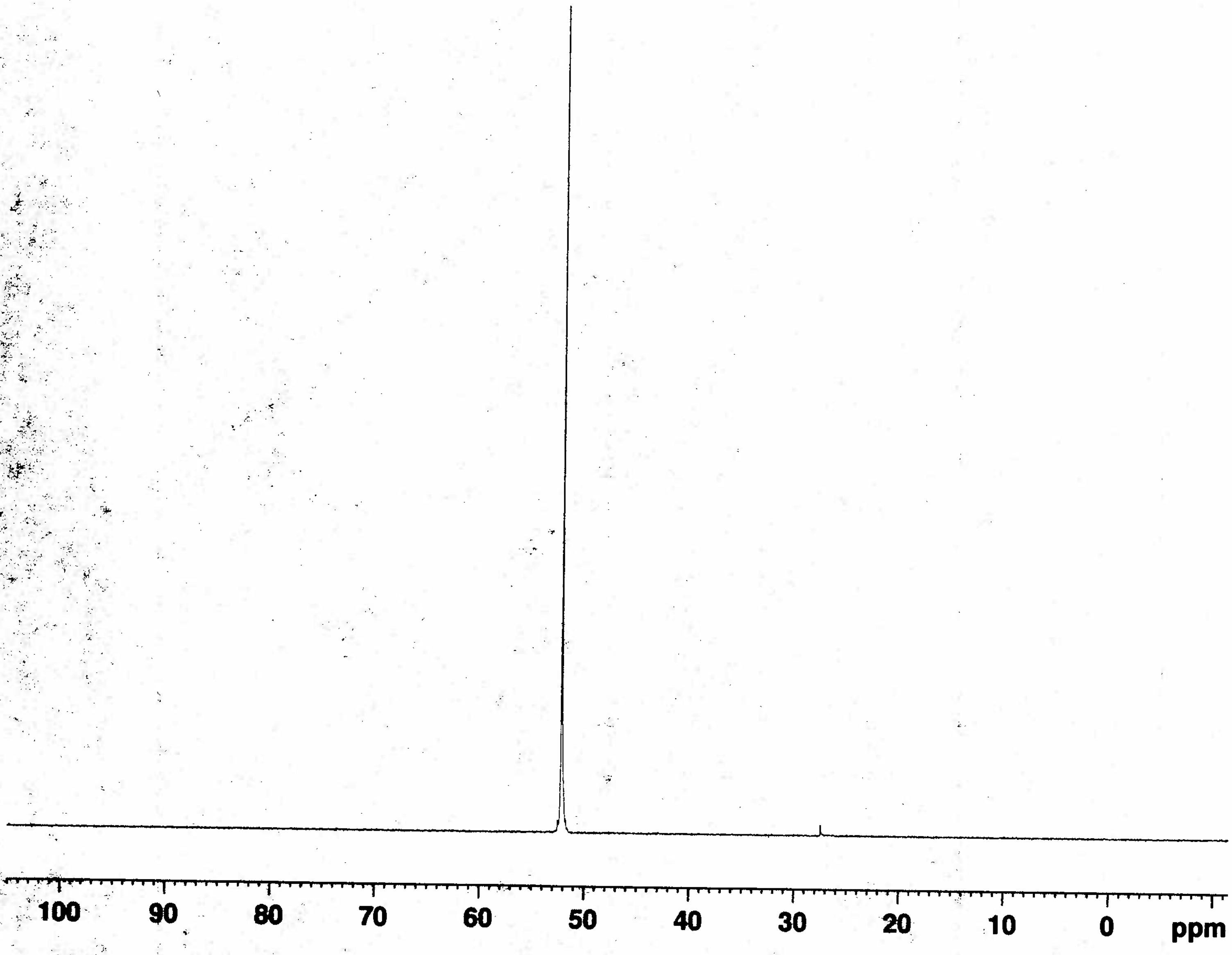

Current Data Parameters
NAME AFL047
EXPNO 3
PROCNO 1

F2 - Acquisition Parameters

Date_	20150721
Time	11.31
INSTRUM	spect
PROBHD	5 mm PABBO BB/
PULPROG	zgpg30
TD	65536
SOLVENT	CDCl3
NS	16
DS	4
SWH	64102.563 Hz
FIDRES	0.978127 Hz
AQ	0.5111808 sec
RG	203.57
DW	7.800 usec
DE	6.50 usec
TE	295.0 K
D1	2.00000000 sec
D11	0.03000000 sec
TD0	1

======== CHANNEL f1 ========

SFO1	161.9674942 MHz
NUC1	31P
P1	14.25 usec
PLW1	15.00000000 W

======== CHANNEL f2 ========

SFO2	400.1316005 MHz
NUC2	1H
CPDPRG[2	waltz16
PCPD2	90.00 usec
PLW2	10.00000000 W
PLW12	0.31604999 W
PLW13	0.25600001 W

F2 - Processing parameters

SI	32768
SF	161.9755930 MHz
WDW	EM
SSB	0
LB	1.00 Hz
GB	0
PC	1.40

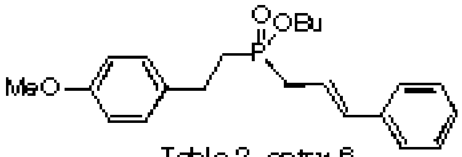

Table 2, entry 6

$^{31}P/^{1}H$ NMR coupled

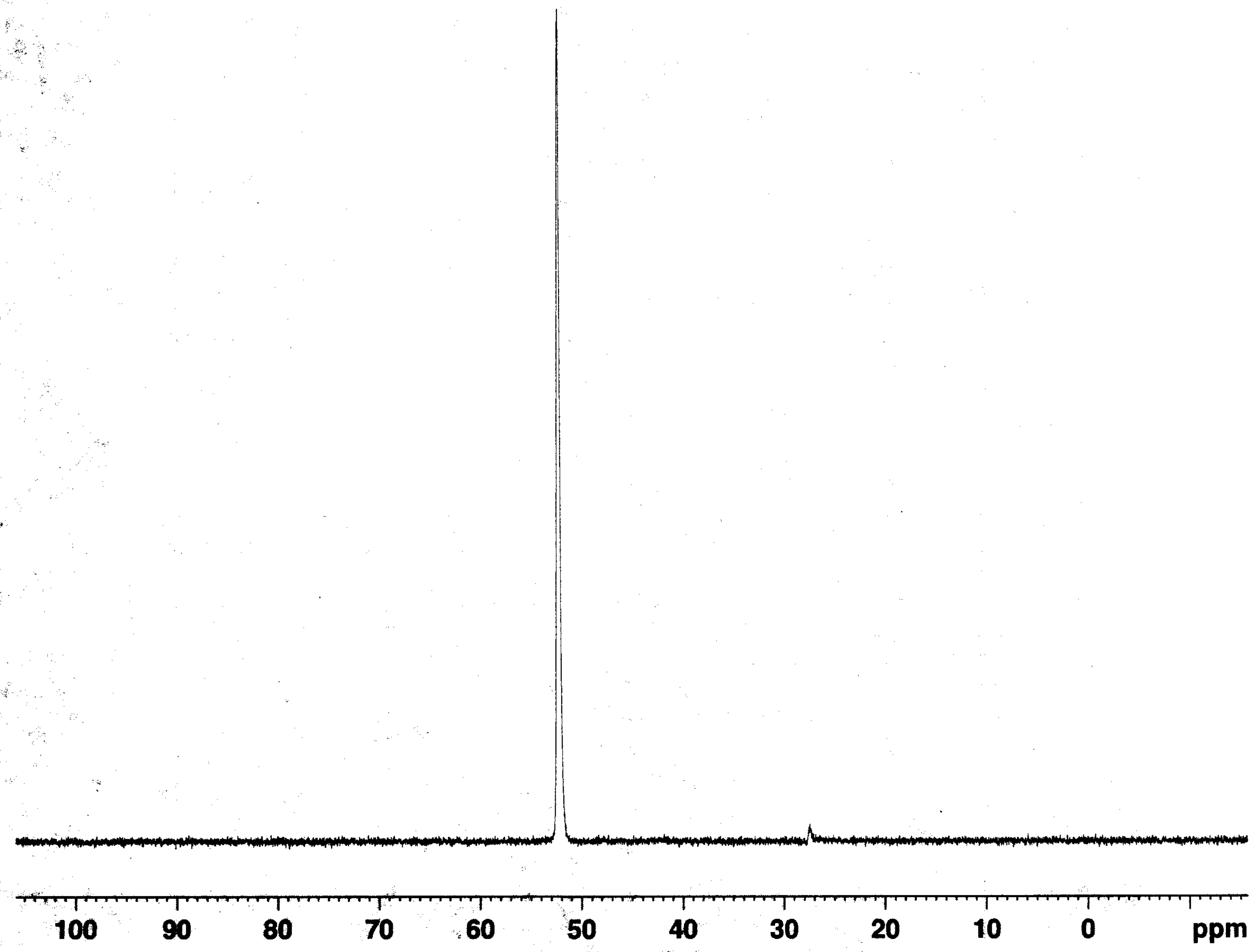


```
Current Data Parameters
NAME              AFL047
EXPNO                  4
PROCNO                 1

F2 - Acquisition Parameters
Date_           20150721
Time               11.33
INSTRUM            spect
PROBHD   5 mm PABBO BB/
PULPROG             zg30
TD                 65536
SOLVENT            CDCl3
NS                    30
DS                     4
SWH            64102.563 Hz
FIDRES          0.978127 Hz
AQ             0.5111808 sec
RG                203.57
DW                 7.800 usec
DE                  6.50 usec
TE                 294.6 K
D1            2.00000000 sec
TD0                    1

======== CHANNEL f1 ========
SFO1         161.9674942 MHz
NUC1                 31P
P1                 14.25 usec
PLW1         15.00000000 W

F2 - Processing parameters
SI                 32768
SF           161.9755930 MHz
WDW                   EM
SSB      0
LB                  1.00 Hz
GB       0
PC                  1.40
```

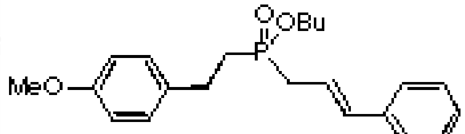

Table 2, entry 6
^{1}H NMR

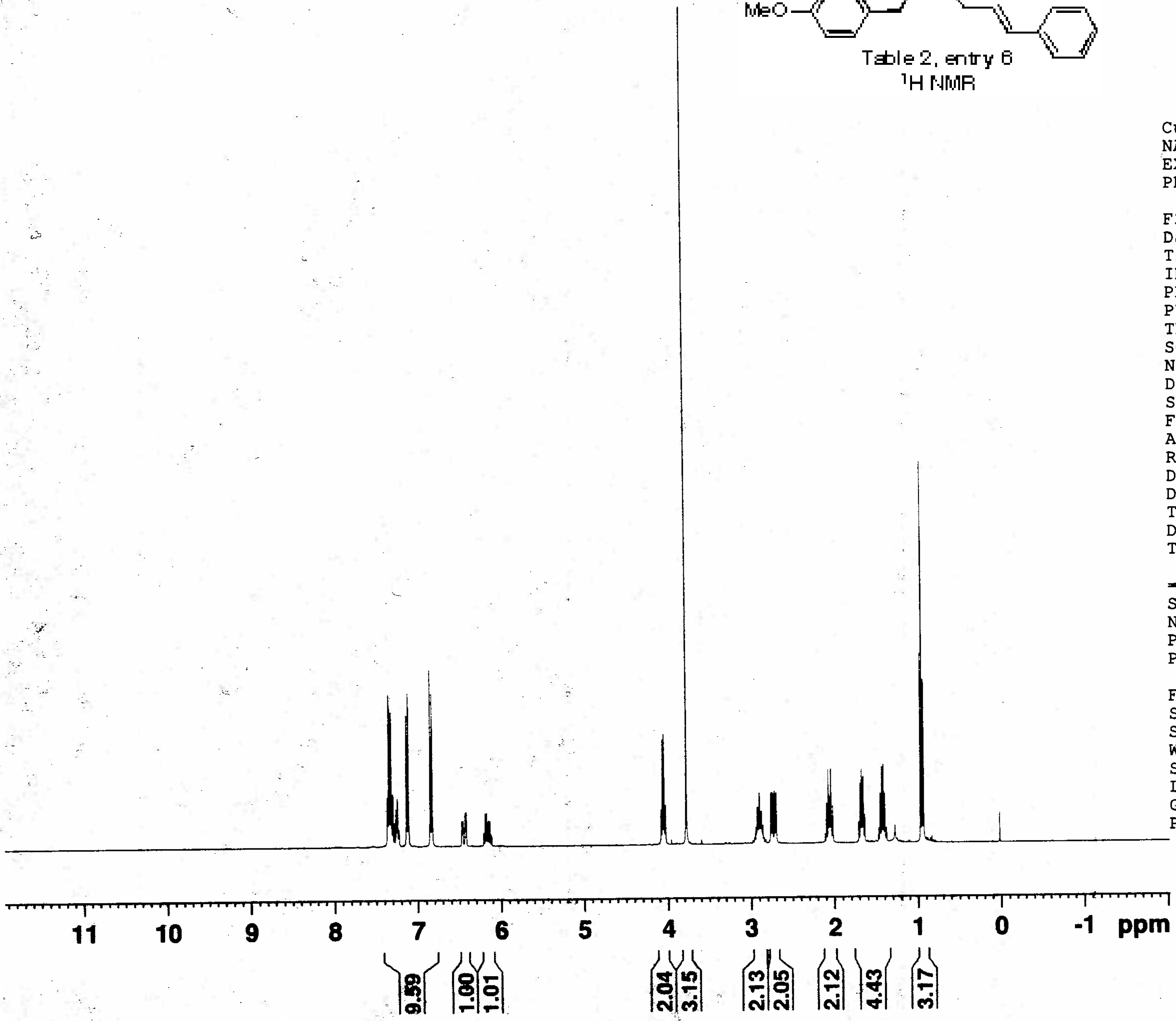

Current Data Parameters
NAME AFL047
EXPNO 5
PROCNO 1

F2 - Acquisition Parameters
Date_ 20150721
Time 11.40
INSTRUM spect
PROBHD 5 mm PABBO BB/
PULPROG zg30
TD 65536
SOLVENT CDCl3
NS 16
DS 2
SWH 8012.820 Hz
FIDRES 0.122266 Hz
AQ 4.0894465 sec
RG 32.38
DW 62.400 usec
DE 6.50 usec
TE 294.6 K
D1 1.00000000 sec
TD0 1

======== CHANNEL f1 ========
SFO1 400.1324710 MHz
NUC1 1H
P1 10.00 usec
PLW1 25.00300026 W

F2 - Processing parameters
SI 65536
SF 400.1300000 MHz
WDW EM
SSB 0
LB 0.30 Hz
GB 0
PC 1.00

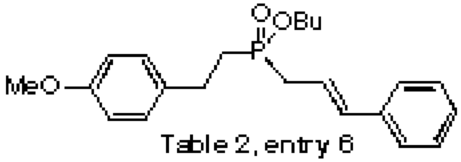

Table 2, entry 6
^{13}C NMR

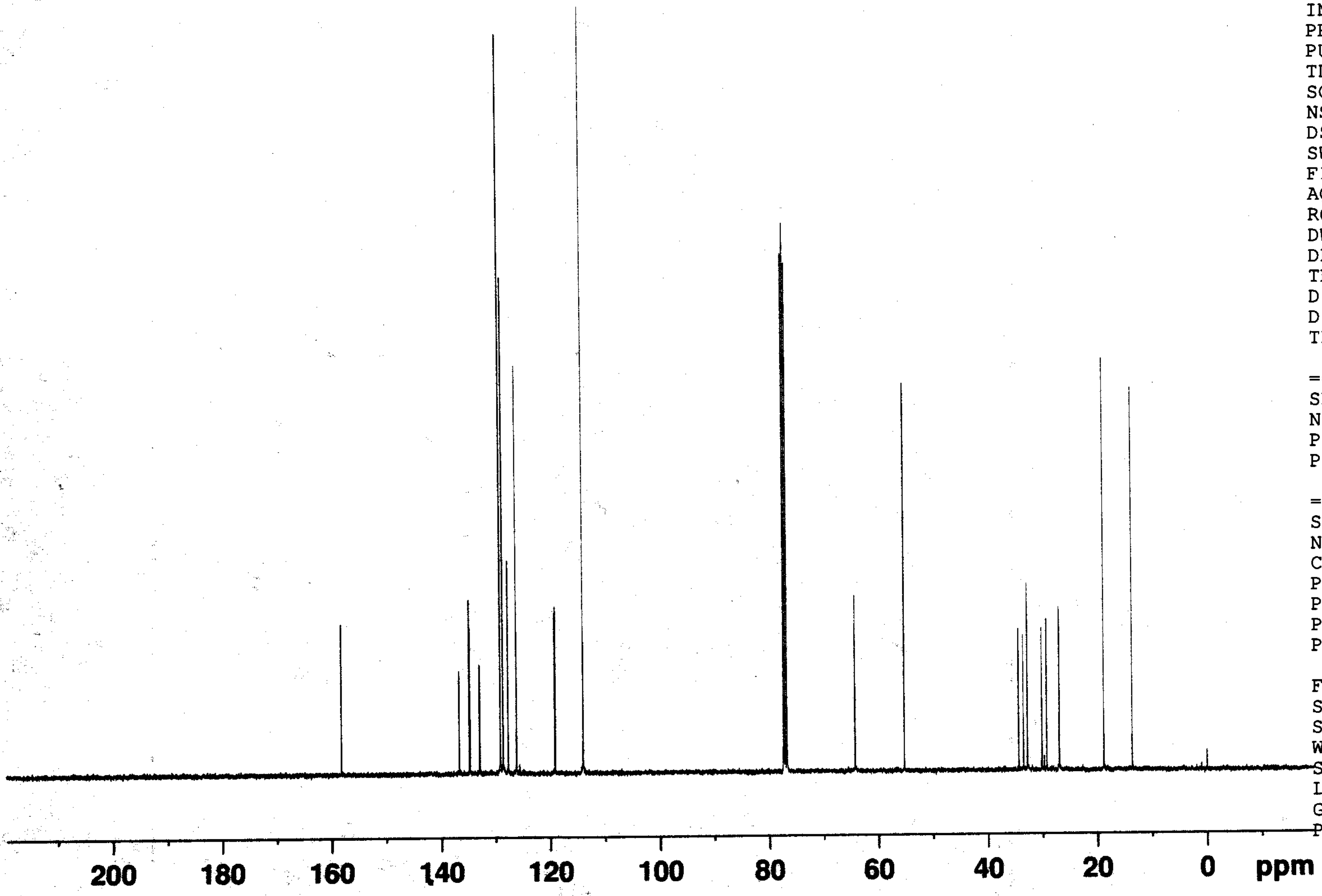

Current Data Parameters
NAME AFL047
EXPNO 6
PROCNO 1

F2 - Acquisition Parameters
Date_ 20150826
Time 10.34
INSTRUM spect
PROBHD 5 mm PABBO BB/
PULPROG zgpg30
TD 65536
SOLVENT CDCl3
NS 682
DS 4
SWH 24038.461 Hz
FIDRES 0.366798 Hz
AQ 1.3631488 sec
RG 203.57
DW 20.800 usec
DE 6.50 usec
TE 295.0 K
D1 2.00000000 sec
D11 0.03000000 sec
TD0 1

======== CHANNEL f1 ========
SFO1 100.6228293 MHz
NUC1 13C
P1 10.00 usec
PLW1 45.00000000 W

======== CHANNEL f2 ========
SFO2 400.1316005 MHz
NUC2 1H
CPDPRG[2 waltz16
PCPD2 90.00 usec
PLW2 10.00000000 W
PLW12 0.31604999 W
PLW13 0.25600001 W

F2 - Processing parameters
SI 32768
SF 100.6127685 MHz
WDW EM
SSB 0
LB 1.00 Hz
GB 0
PC 1.40

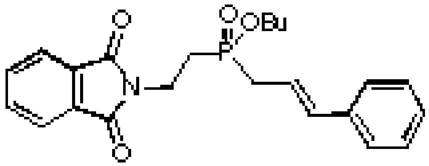

Table 2, entry 7
$^{31}P/^{1}H$ NMR decoupled

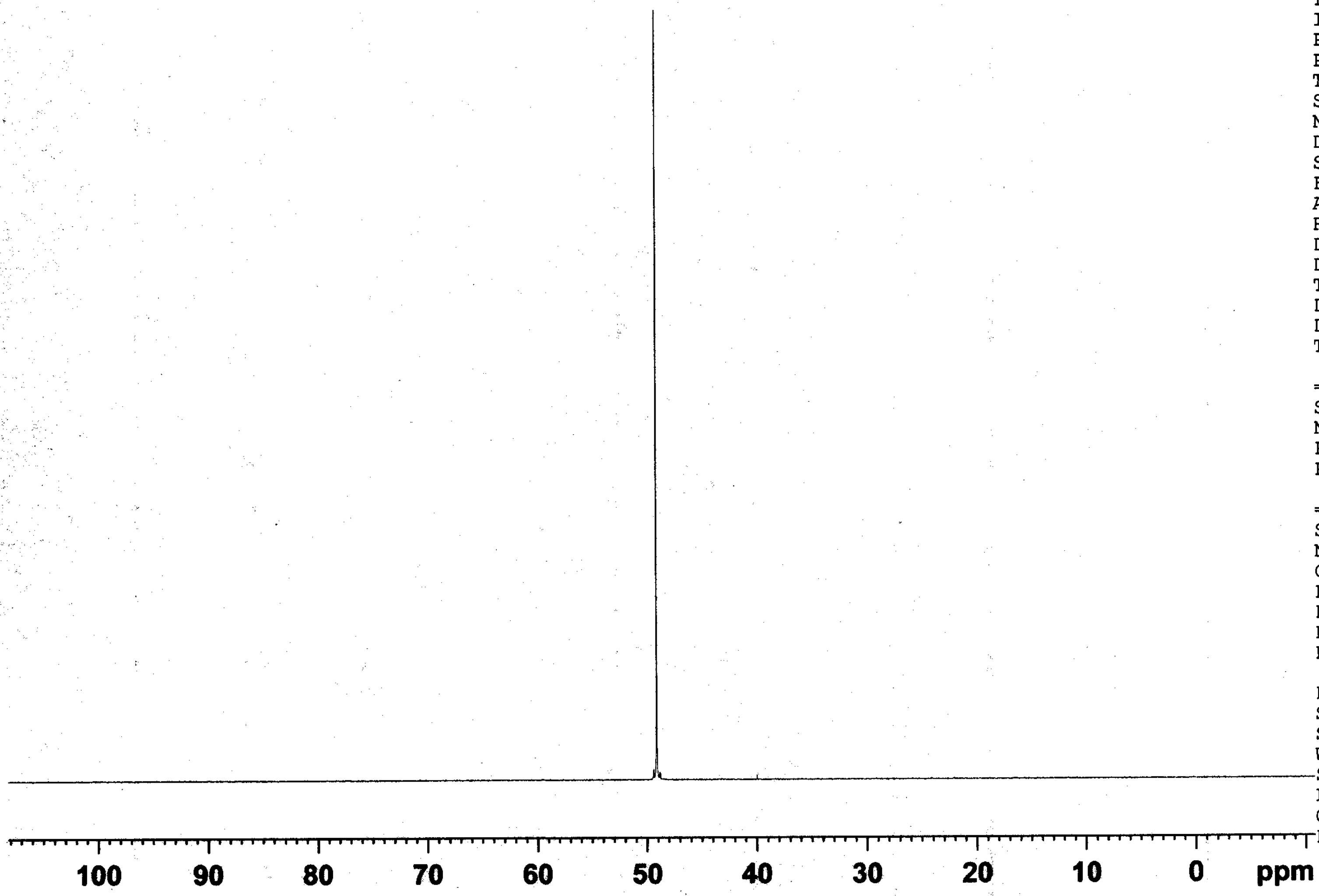

Current Data Parameters	
NAME	OB 1977 pure
EXPNO	1
PROCNO	1

F2 - Acquisition Parameters	
Date_	20150409
Time	17.05
INSTRUM	spect
PROBHD	5 mm PABBO BB/
PULPROG	zgpg30
TD	65536
SOLVENT	CDCl3
NS	16
DS	4
SWH	64102.563 Hz
FIDRES	0.978127 Hz
AQ	0.5111808 sec
RG	203.57
DW	7.800 usec
DE	6.50 usec
TE	294.4 K
D1	2.00000000 sec
D11	0.03000000 sec
TD0	1

======== CHANNEL f1 ========	
SFO1	161.9674942 MHz
NUC1	31P
P1	14.25 usec
PLW1	15.00000000 W

======== CHANNEL f2 ========	
SFO2	400.1316005 MHz
NUC2	1H
CPDPRG[2	waltz16
PCPD2	90.00 usec
PLW2	10.00000000 W
PLW12	0.31604999 W
PLW13	0.25600001 W

F2 - Processing parameters	
SI	32768
SF	161.9755930 MHz
WDW	EM
SSB	0
LB	1.00 Hz
GB	0
PC	1.40

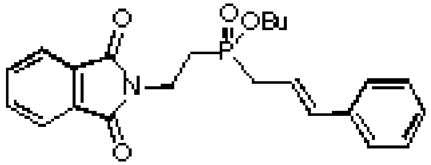

Table 2, entry 7

$^{31}P/^{1}H$ NMR coupled

Current Data Parameters
NAME OB 1977 pure
EXPNO 2
PROCNO 1

F2 - Acquisition Parameters
Date_ 20150409
Time 17.07
INSTRUM spect
PROBHD 5 mm PABBO BB/
PULPROG zg30
TD 65536
SOLVENT CDCl3
NS 32
DS 4
SWH 64102.563 Hz
FIDRES 0.978127 Hz
AQ 0.5111808 sec
RG 203.57
DW 7.800 usec
DE 6.50 usec
TE 294.1 K
D1 2.00000000 sec
TD0 1

======== CHANNEL f1 ========
SFO1 161.9674942 MHz
NUC1 31P
P1 14.25 usec
PLW1 15.00000000 W

F2 - Processing parameters
SI 32768
SF 161.9755930 MHz
WDW EM
SSB 0
LB 1.00 Hz
GB 0
PC 1.40

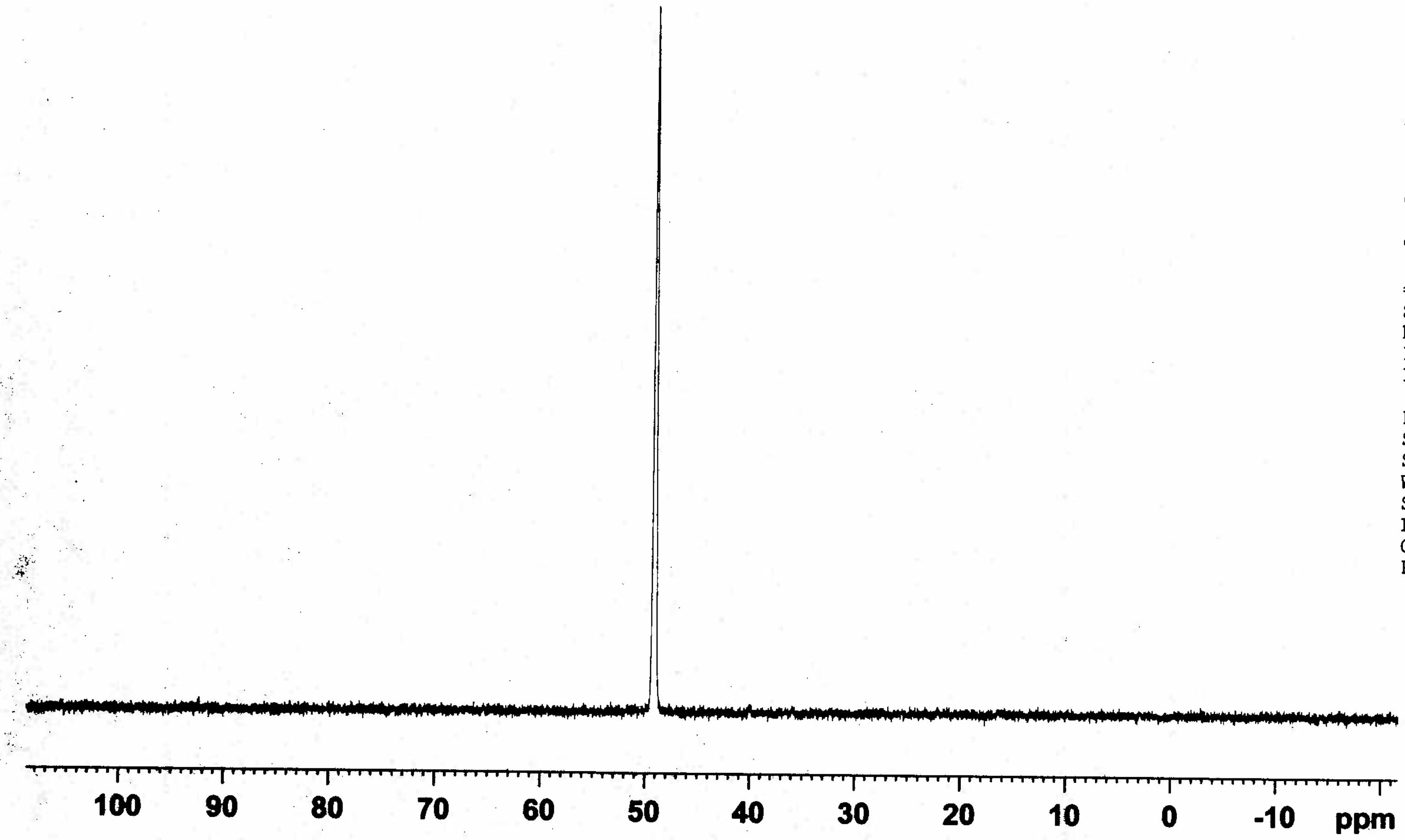

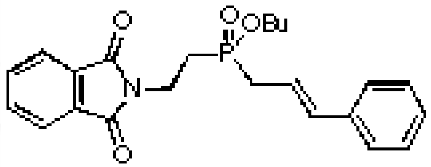

Table 2, entry 7
^{1}H NMR

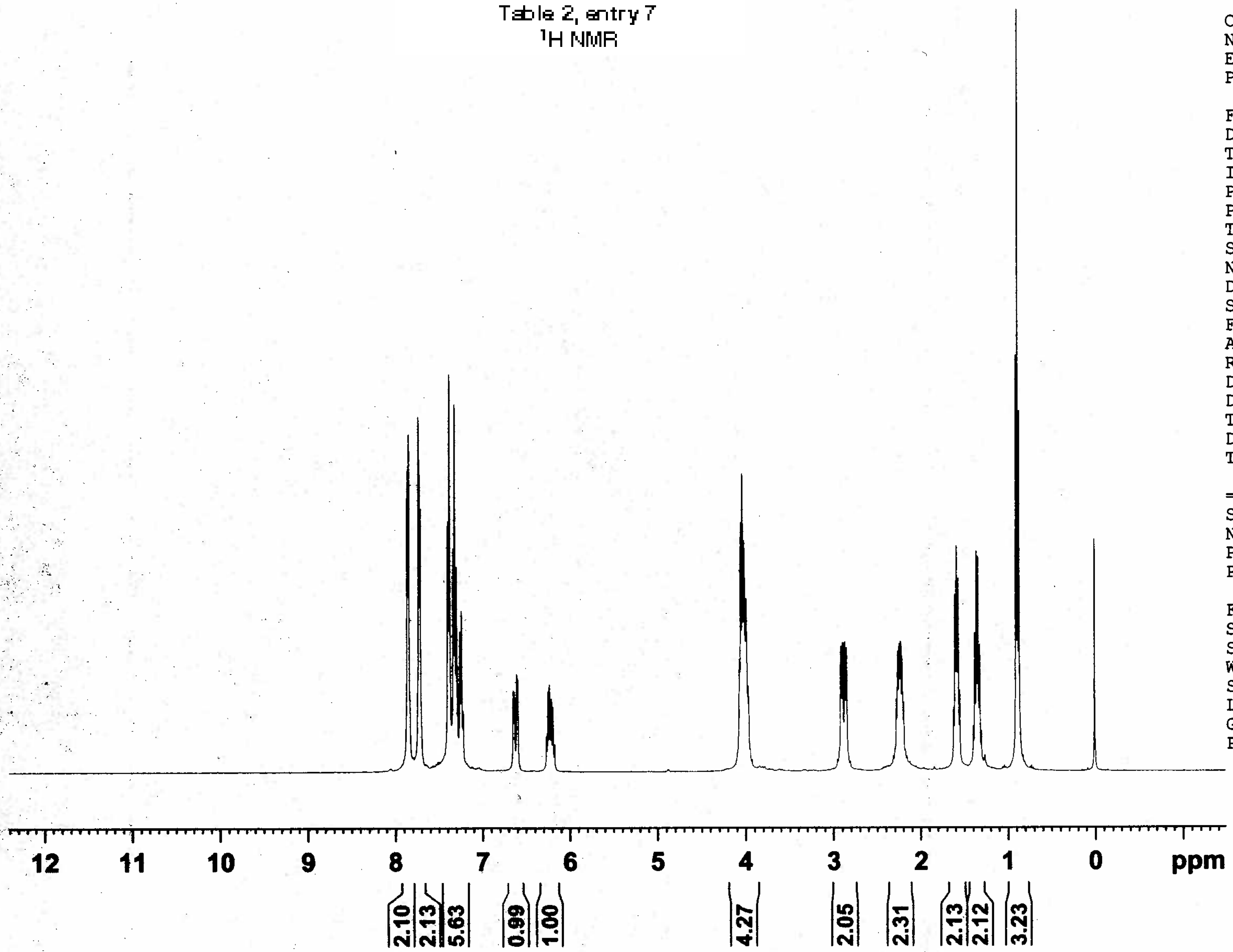

Current Data Parameters	
NAME	OB 1977 pure
EXPNO	3
PROCNO	1
F2 - Acquisition Parameters	
Date_	20150409
Time	17.08
INSTRUM	spect
PROBHD	5 mm PABBO BB/
PULPROG	zg30
TD	65536
SOLVENT	CDCl3
NS	9
DS	2
SWH	8012.820 Hz
FIDRES	0.122266 Hz
AQ	4.0894465 sec
RG	51.43
DW	62.400 usec
DE	6.50 usec
TE	294.2 K
D1	1.00000000 sec
TD0	1
======== CHANNEL f1 ========	
SFO1	400.1324710 MHz
NUC1	1H
P1	10.00 usec
PLW1	25.00300026 W
F2 - Processing parameters	
SI	65536
SF	400.1300000 MHz
WDW	EM
SSB	0
LB	0.30 Hz
GB	0
PC	1.00

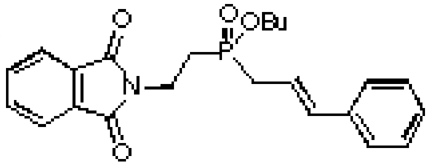

Table 2, entry 7
^{13}C NMR

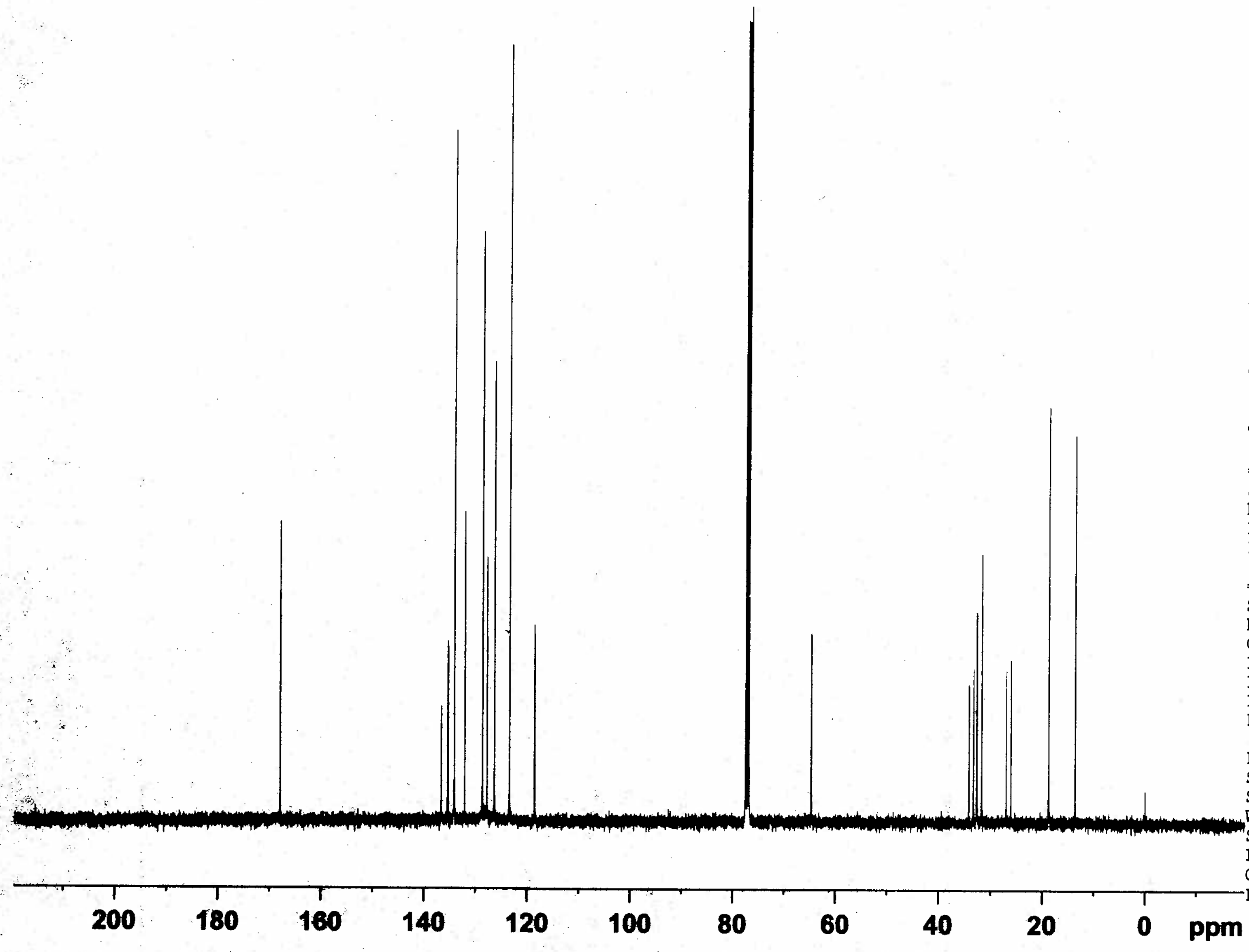

BRUKER

Current Data Parameters
NAME OB 1977 pure
EXPNO 4
PROCNO 1

F2 - Acquisition Parameters
Date_ 20150409
Time 17.30
INSTRUM spect
PROBHD 5 mm PABBO BB/
PULPROG zgpg30
TD 65536
SOLVENT CDCl3
NS 332
DS 4
SWH 24038.461 Hz
FIDRES 0.366798 Hz
AQ 1.3631488 sec
RG 203.57
DW 20.800 usec
DE 6.50 usec
TE 294.9 K
D1 2.00000000 sec
D11 0.03000000 sec
TD0 1

======== CHANNEL f1 ========
SFO1 100.6228293 MHz
NUC1 13C
P1 10.00 usec
PLW1 45.00000000 W

======== CHANNEL f2 ========
SFO2 400.1316005 MHz
NUC2 1H
CPDPRG[2 waltz16
PCPD2 90.00 usec
PLW2 10.00000000 W
PLW12 0.31604999 W
PLW13 0.25600001 W

F2 - Processing parameters
SI 32768
SF 100.6127685 MHz
WDW EM
SSB 0
LB 1.00 Hz
GB 0
PC 1.40

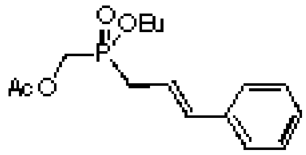

Table 2, entry 8
$^{31}P/^{1}H$ NMR decoupled

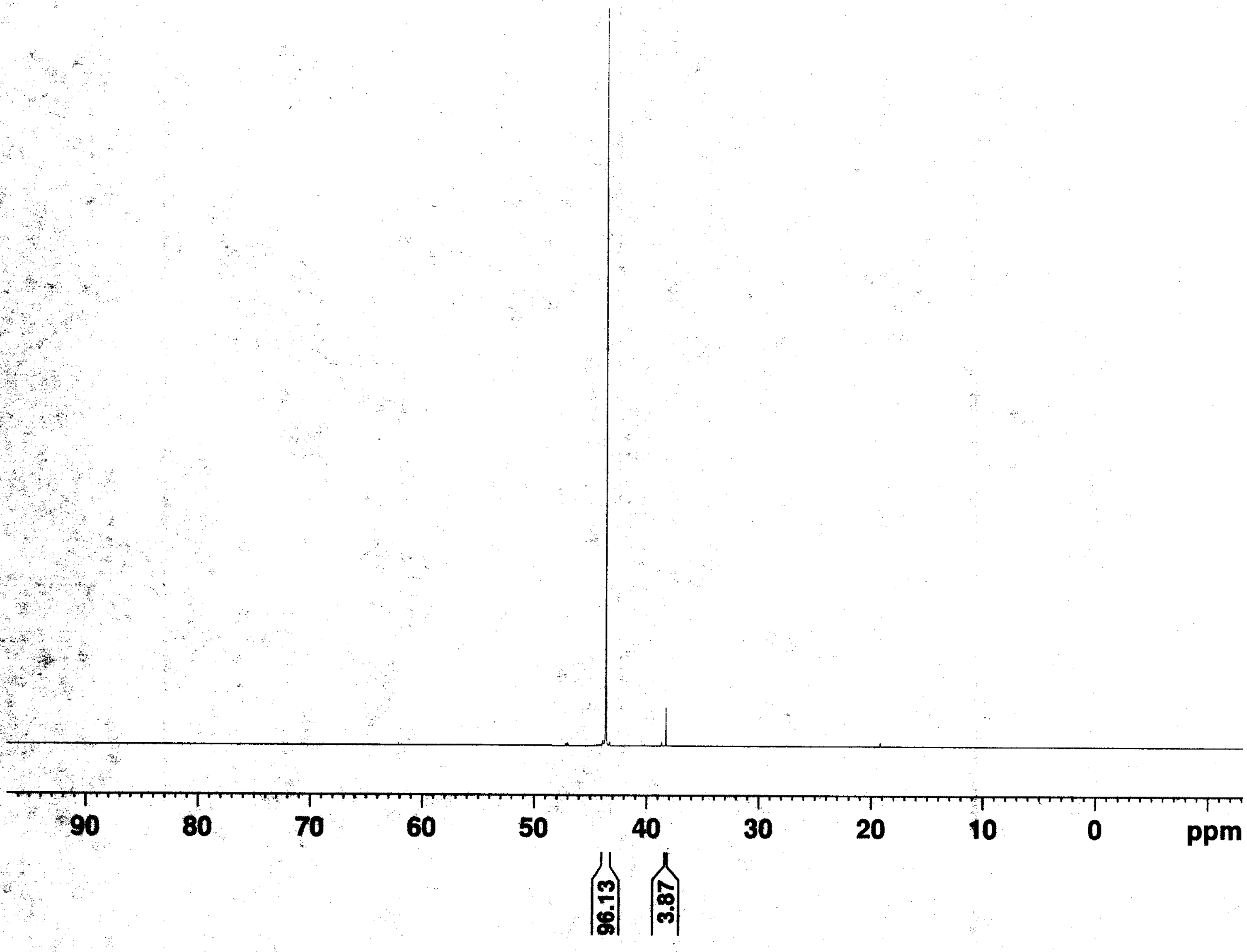

Current Data Parameters
NAME AFL043
EXPNO 3
PROCNO 1

F2 - Acquisition Parameters
Date_ 20150720
Time 14.00
INSTRUM spect
PROBHD 5 mm PABBO BB/
PULPROG zgpg30
TD 65536
SOLVENT CDCl3
NS 16
DS 4
SWH 64102.563 Hz
FIDRES 0.978127 Hz
AQ 0.5111808 sec
RG 203.57
DW 7.800 usec
DE 6.50 usec
TE 295.6 K
D1 2.00000000 sec
D11 0.03000000 sec
TD0 1

======== CHANNEL f1 ========
SFO1 161.9674942 MHz
NUC1 31P
P1 14.25 usec
PLW1 15.00000000 W

======== CHANNEL f2 ========
SFO2 400.1316005 MHz
NUC2 1H
CPDPRG[2 waltz16
PCPD2 90.00 usec
PLW2 10.00000000 W
PLW12 0.31604999 W
PLW13 0.25600001 W

F2 - Processing parameters
SI 32768
SF 161.9755930 MHz
WDW EM
SSB 0
LB 1.00 Hz
GB 0
PC 1.40

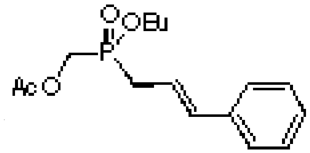

Table 2, entry 8
^{31}P/^{1}H NMR coupled

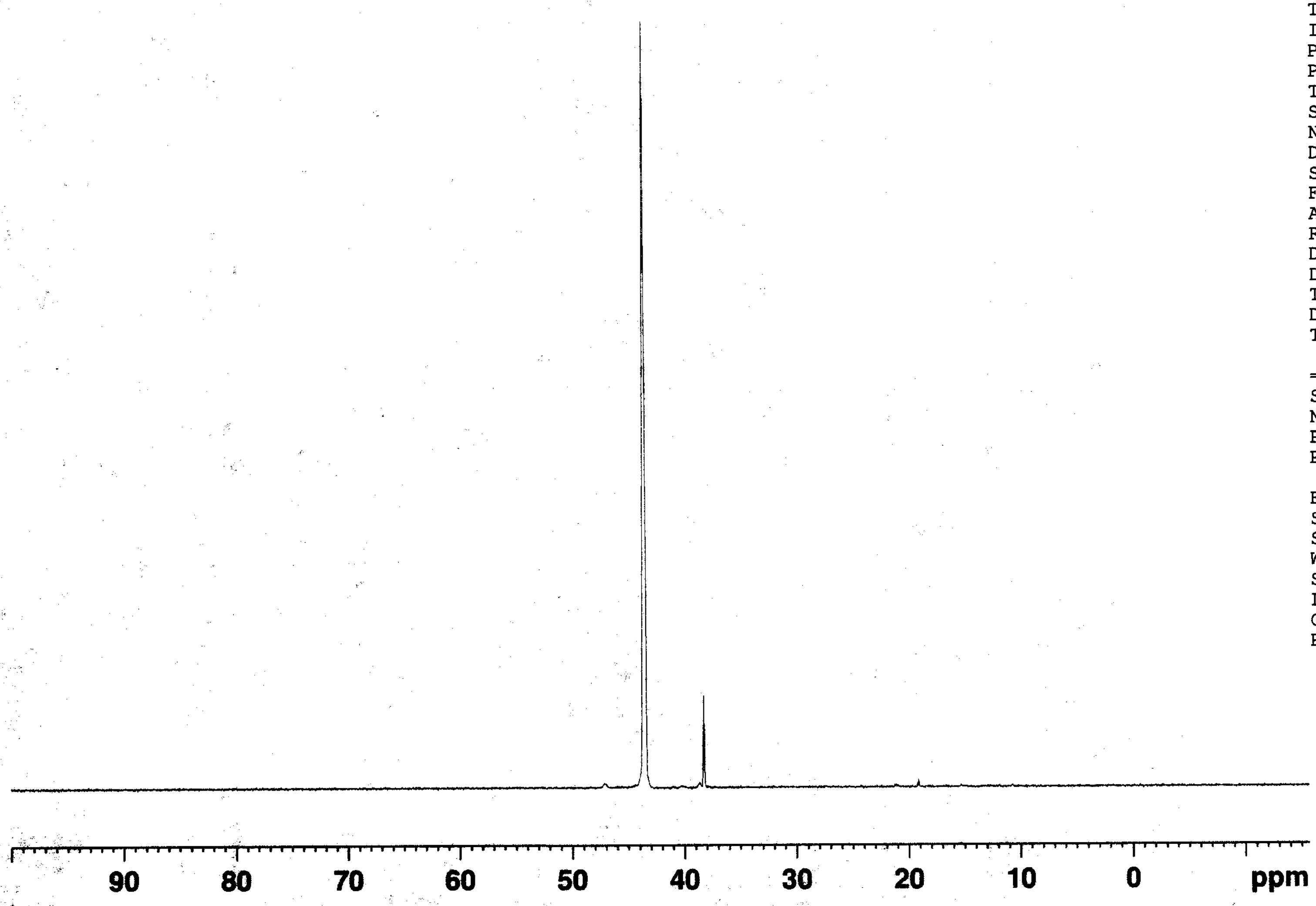

Current Data Parameters
NAME AFL043
EXPNO 4
PROCNO 1

F2 - Acquisition Parameters
Date_ 20150720
Time 14.04
INSTRUM spect
PROBHD 5 mm PABBO BB/
PULPROG zg30
TD 65536
SOLVENT CDCl3
NS 32
DS 4
SWH 64102.563 Hz
FIDRES 0.978127 Hz
AQ 0.5111808 sec
RG 203.57
DW 7.800 usec
DE 6.50 usec
TE 295.2 K
D1 2.00000000 sec
TD0 1

======== CHANNEL f1 ========
SFO1 161.9674942 MHz
NUC1 31P
P1 14.25 usec
PLW1 15.00000000 W

F2 - Processing parameters
SI 32768
SF 161.9755930 MHz
WDW EM
SSB 0
LB 1.00 Hz
GB 0
PC 1.40

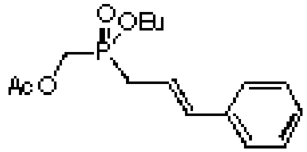

Table 2, entry 8

^{1}H NMR

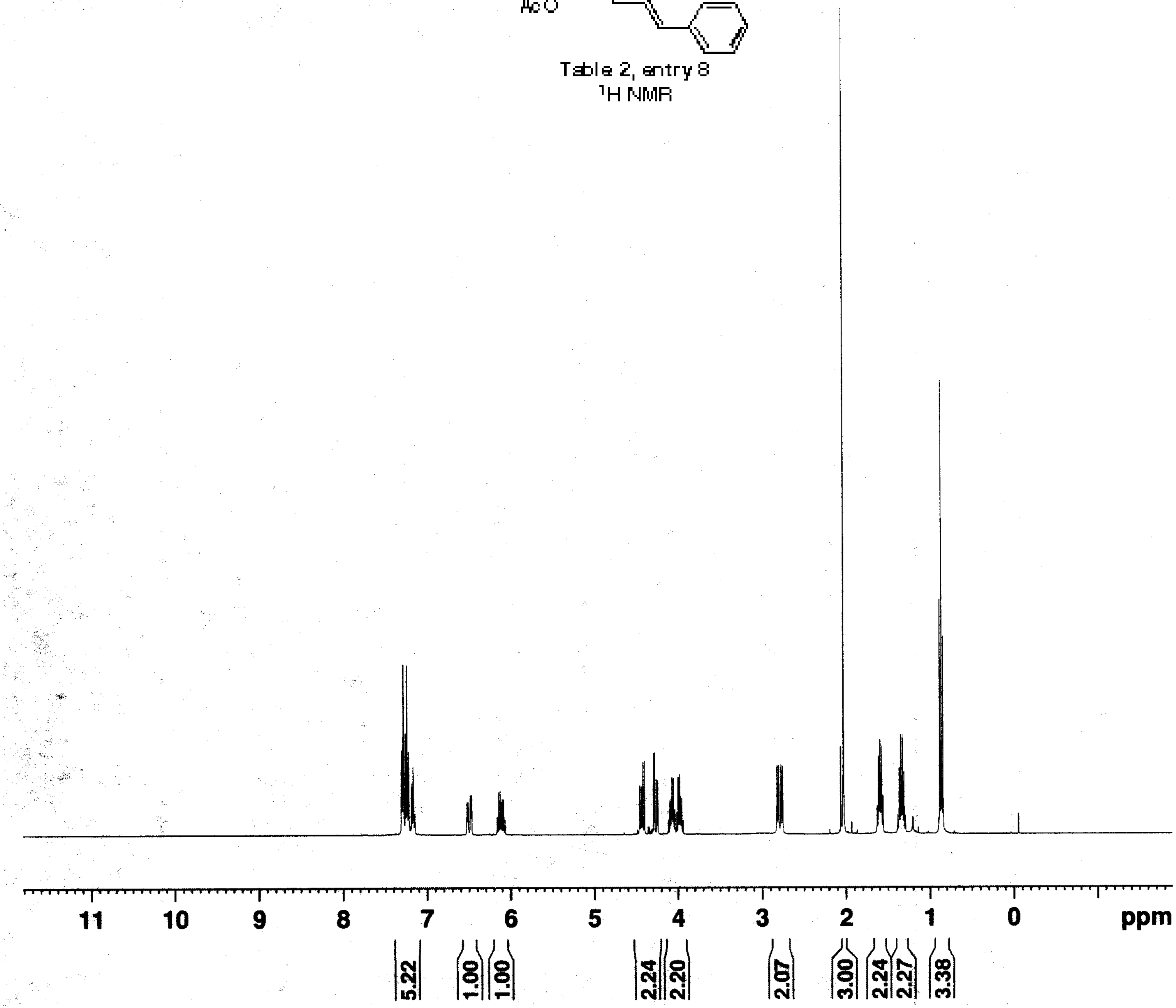


```
Current Data Parameters
NAME              AFL043
EXPNO                  5
PROCNO                 1

F2 - Acquisition Parameters
Date_           20150720
Time               14.11
INSTRUM            spect
PROBHD   5 mm PABBO BB/
PULPROG             zg30
TD                 65536
SOLVENT            CDCl3
NS                    12
DS                     2
SWH             8012.820 Hz
FIDRES          0.122266 Hz
AQ             4.0894465 sec
RG                 13.94
DW                62.400 usec
DE                  6.50 usec
TE                 295.2 K
D1            1.00000000 sec
TD0                    1

======== CHANNEL f1 ========
SFO1         400.1324710 MHz
NUC1                  1H
P1                 10.00 usec
PLW1         25.00300026 W

F2 - Processing parameters
SI                 65536
SF           400.1300000 MHz
WDW                   EM
SSB       0
LB                  0.30 Hz
GB        0
PC                  1.00
```

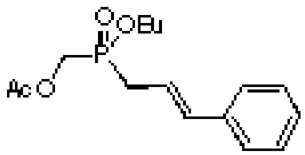

Table 2, entry 8
^{13}C NMR

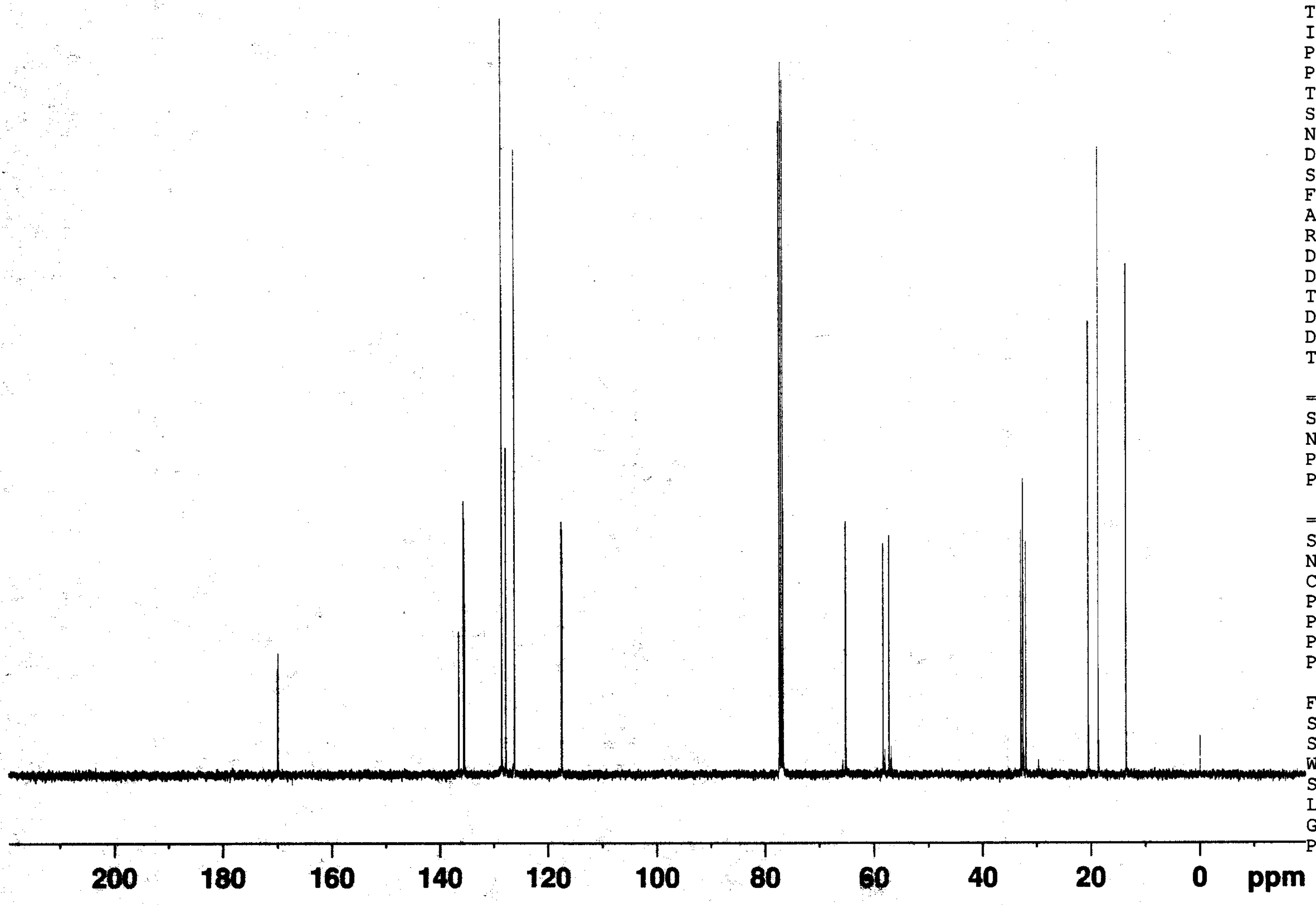


```
Current Data Parameters
NAME              AFL043
EXPNO                  6
PROCNO                 1

F2 - Acquisition Parameters
Date_           20150827
Time                9.40
INSTRUM            spect
PROBHD   5 mm PABBO BB/
PULPROG          zgpg30
TD                65536
SOLVENT           CDCl3
NS                  402
DS                    4
SWH           24038.461 Hz
FIDRES         0.366798 Hz
AQ            1.3631488 sec
RG               203.57
DW               20.800 usec
DE                 6.50 usec
TE                295.4 K
D1           2.00000000 sec
D11          0.03000000 sec
TD0                   1

======== CHANNEL f1 ========
SFO1        100.6228293 MHz
NUC1                13C
P1                10.00 usec
PLW1        45.00000000 W

======== CHANNEL f2 ========
SFO2        400.1316005 MHz
NUC2                 1H
CPDPRG[2         waltz16
PCPD2             90.00 usec
PLW2        10.00000000 W
PLW12        0.31604999 W
PLW13        0.25600001 W

F2 - Processing parameters
SI                32768
SF          100.6127685 MHz
WDW                  EM
SSB      0
LB                 1.00 Hz
GB       0
PC                 1.40
```

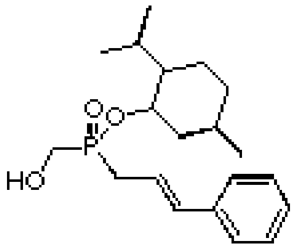

Table 2, entry 9
$^{31}P/^{1}H$ NMR decoupled

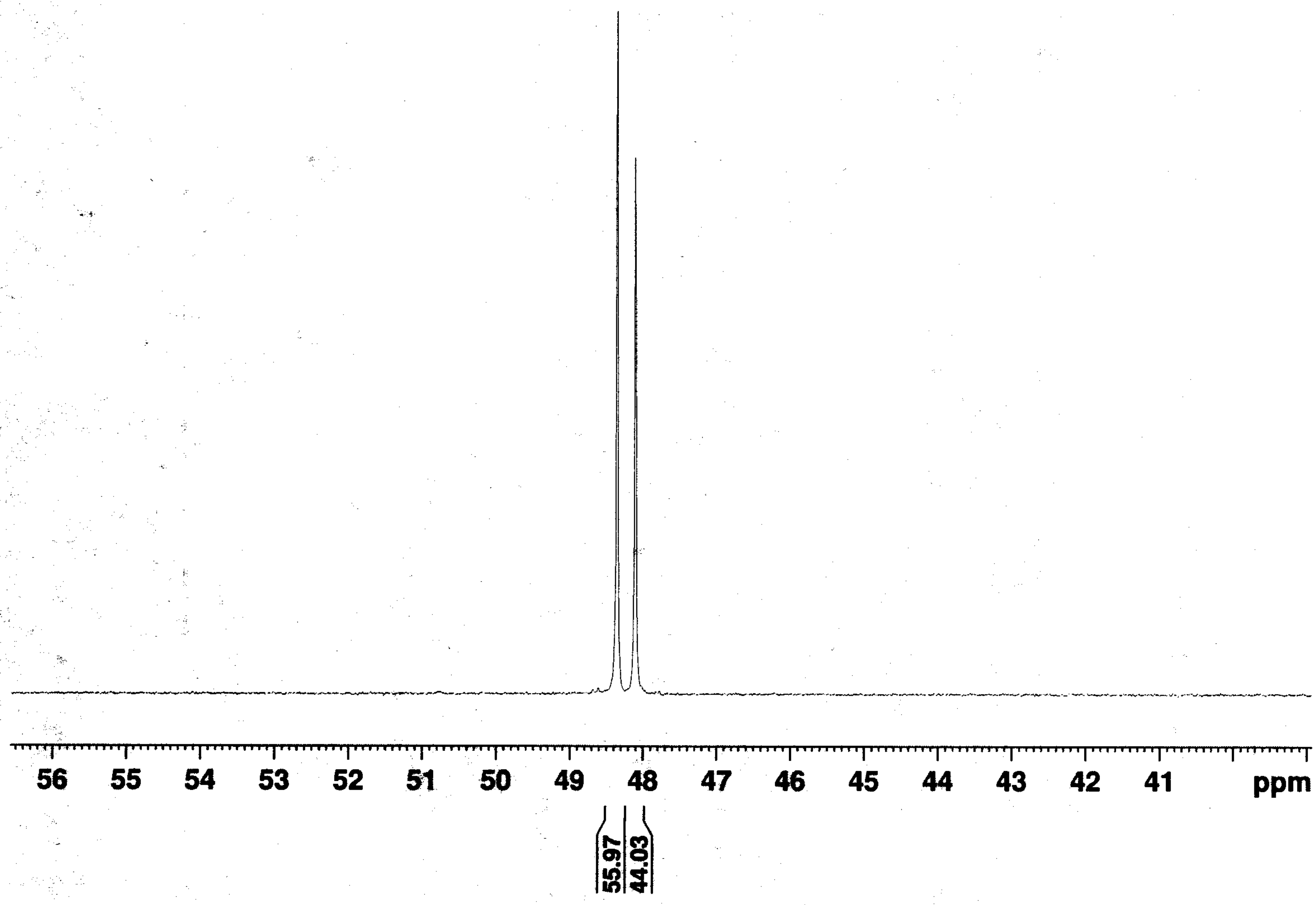

Current Data Parameters
NAME   OB 2071 1st crystallization
EXPNO   1
PROCNO   1

F2 - Acquisition Parameters
Date_   20150825
Time   10.09
INSTRUM spect
PROBHD   5 mm PABBO BB/
PULPROG zgpg30
TD   65536
SOLVENT CDC13
NS   16
DS   4
SWH   64102.563 Hz
FIDRES   0.978127 Hz
AQ   0.5111808 sec
RG   203.57
DW   7.800 usec
DE   6.50 usec
TE   294.9 K
D1   2.00000000 sec
D11   0.03000000 sec
TD0   1

======== CHANNEL f1 ========
SFO1   161.9674942 MHz
NUC1   31P
P1   14.25 usec
PLW1   15.00000000 W

======== CHANNEL f2 ========
SFO2   400.1316005 MHz
NUC2   1H
CPDPRG[2 waltz16
PCPD2   90.00 usec
PLW2   10.00000000 W
PLW12   0.31604999 W
PLW13   0.25600001 W

F2 - Processing parameters
SI   32768
SF   161.9755930 MHz
WDW   EM
SSB   0
LB   1.00 Hz
GB   0
PC   1.40

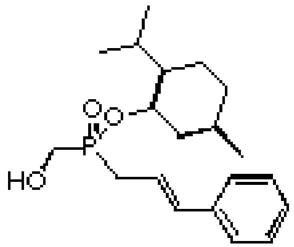

Table 2, entry 9
$^{31}P/^{1}H$ NMR coupled

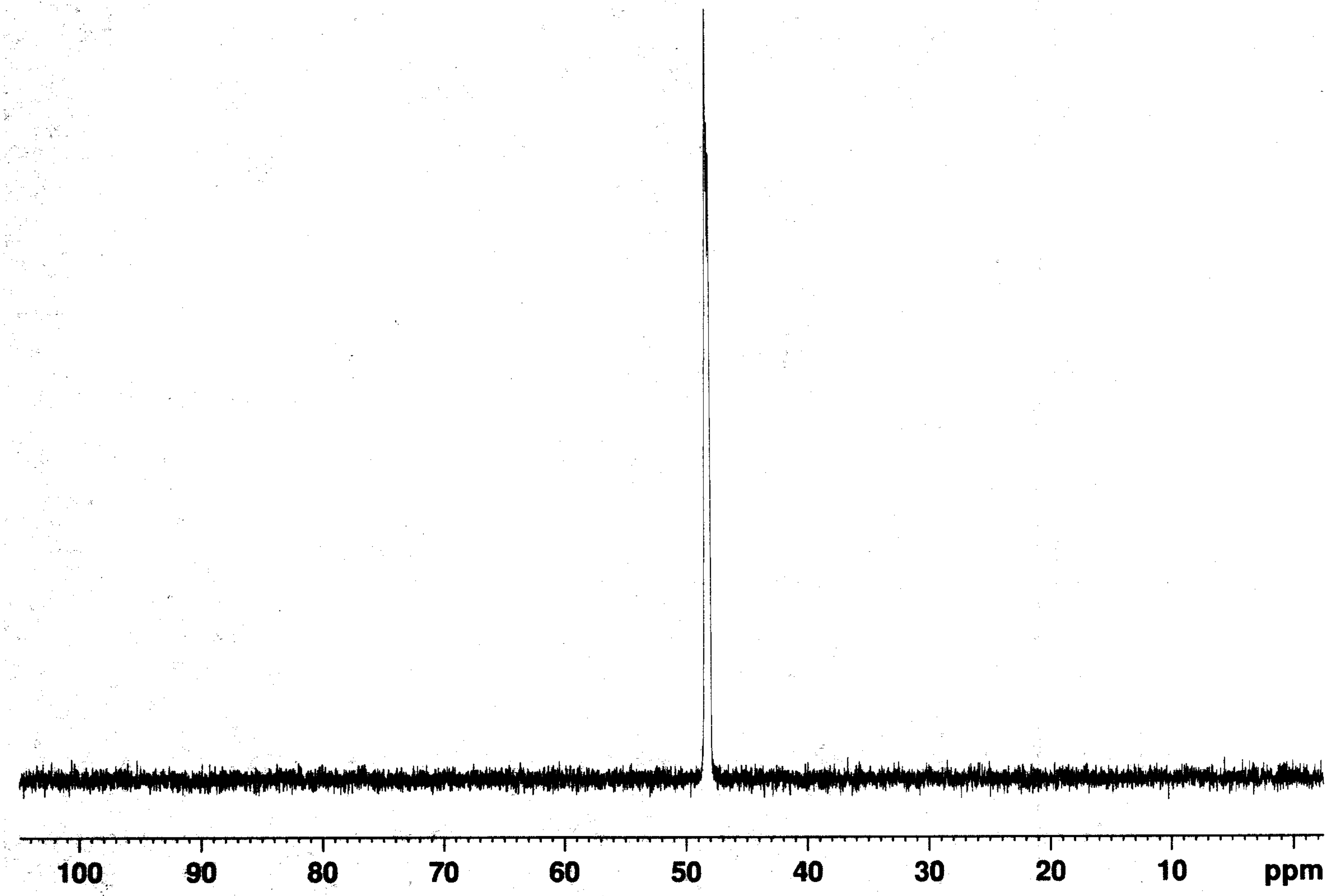


```
Current Data Parameters
NAME     OB 2071 1st crystallization
EXPNO                     2
PROCNO                    1

F2 - Acquisition Parameters
Date_             20150825
Time                 10.10
INSTRUM              spect
PROBHD   5 mm PABBO BB/
PULPROG               zg30
TD                   65536
SOLVENT              CDCl3
NS                      27
DS                       4
SWH              64102.563 Hz
FIDRES            0.978127 Hz
AQ               0.5111808 sec
RG                  203.57
DW                   7.800 usec
DE                    6.50 usec
TE                   294.5 K
D1              2.00000000 sec
TD0                      1

======== CHANNEL f1 ========
SFO1         161.9674942 MHz
NUC1                 31P
P1                 14.25 usec
PLW1         15.00000000 W

F2 - Processing parameters
SI                 32768
SF           161.9755930 MHz
WDW                   EM
SSB      0
LB                  1.00 Hz
GB       0
PC                  1.40
```

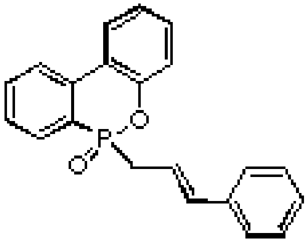

Table 2, entry 11
$^{31}P/^{1}H$ NMR decoupled

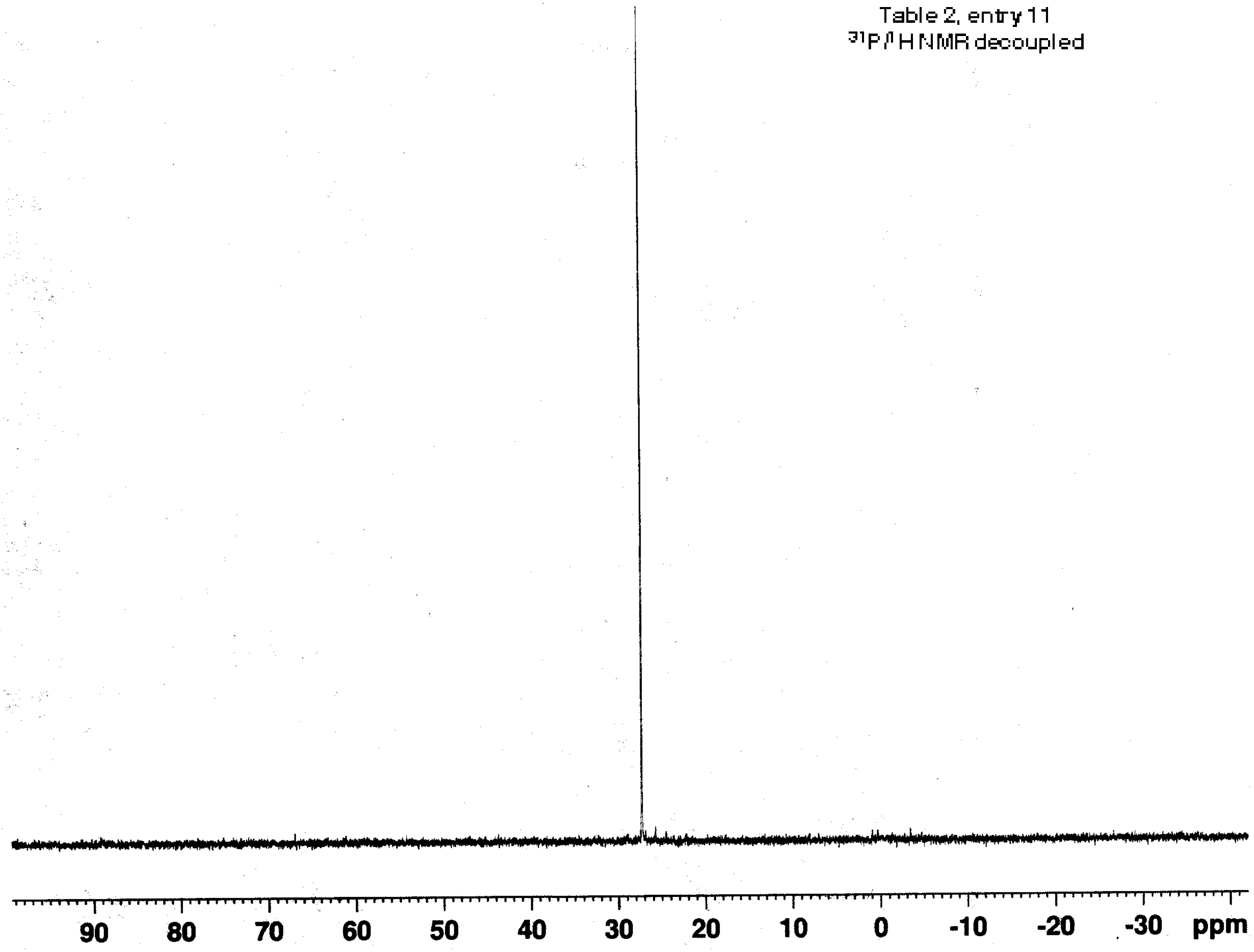


```
Current Data Parameters
NAME              AFL005
EXPNO                  1
PROCNO                 1

F2 - Acquisition Parameters
Date_           20150605
Time               16.35
INSTRUM            spect
PROBHD   5 mm PABBO BB/
PULPROG          zgpg30
TD                 65536
SOLVENT            CDCl3
NS                    16
DS                     4
SWH           64102.563 Hz
FIDRES         0.978127 Hz
AQ            0.5111808 sec
RG                203.57
DW                 7.800 usec
DE                  6.50 usec
TE                 294.7 K
D1            2.00000000 sec
D11           0.03000000 sec
TD0                    1

======== CHANNEL f1 ========
SFO1         161.9674942 MHz
NUC1                 31P
P1                 14.25 usec
PLW1         15.00000000 W

======== CHANNEL f2 ========
SFO2         400.1316005 MHz
NUC2                  1H
CPDPRG[2         waltz16
PCPD2              90.00 usec
PLW2         10.00000000 W
PLW12         0.31604999 W
PLW13         0.25600001 W

F2 - Processing parameters
SI                 32768
SF           161.9755930 MHz
WDW                   EM
SSB      0
LB                  1.00 Hz
GB       0
PC                  1.40
```

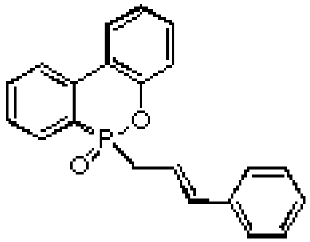

Table 2, entry 11
$^{31}P/^{1}H$ NMR coupled

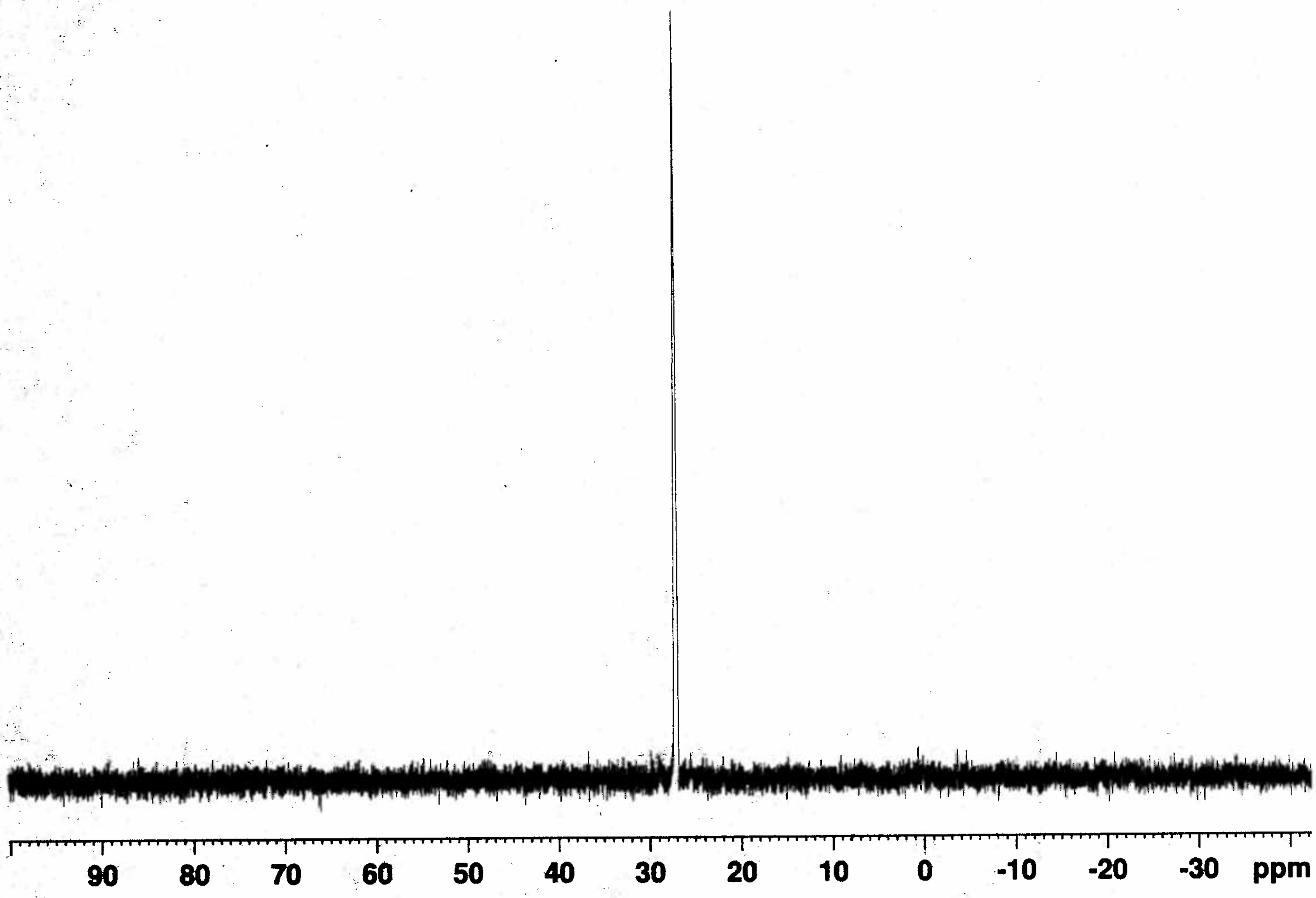

Current Data Parameters
NAME AFL005
EXPNO 2
PROCNO 1

F2 - Acquisition Parameters
Date_ 20150605
Time 16.37
INSTRUM spect
PROBHD 5 mm PABBO BB/
PULPROG zg30
TD 65536
SOLVENT CDCl3
NS 24
DS 4
SWH 64102.563 Hz
FIDRES 0.978127 Hz
AQ 0.5111808 sec
RG 203.57
DW 7.800 usec
DE 6.50 usec
TE 294.4 K
D1 2.00000000 sec
TD0 1

======== CHANNEL f1 ========
SFO1 161.9674942 MHz
NUC1 31P
P1 14.25 usec
PLW1 15.00000000 W

F2 - Processing parameters
SI 32768
SF 161.9755930 MHz
WDW EM
SSB 0
LB 1.00 Hz
GB 0
PC 1.40

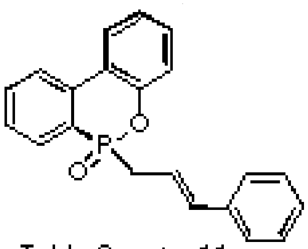

Table 2, entry 11
^{1}H NMR

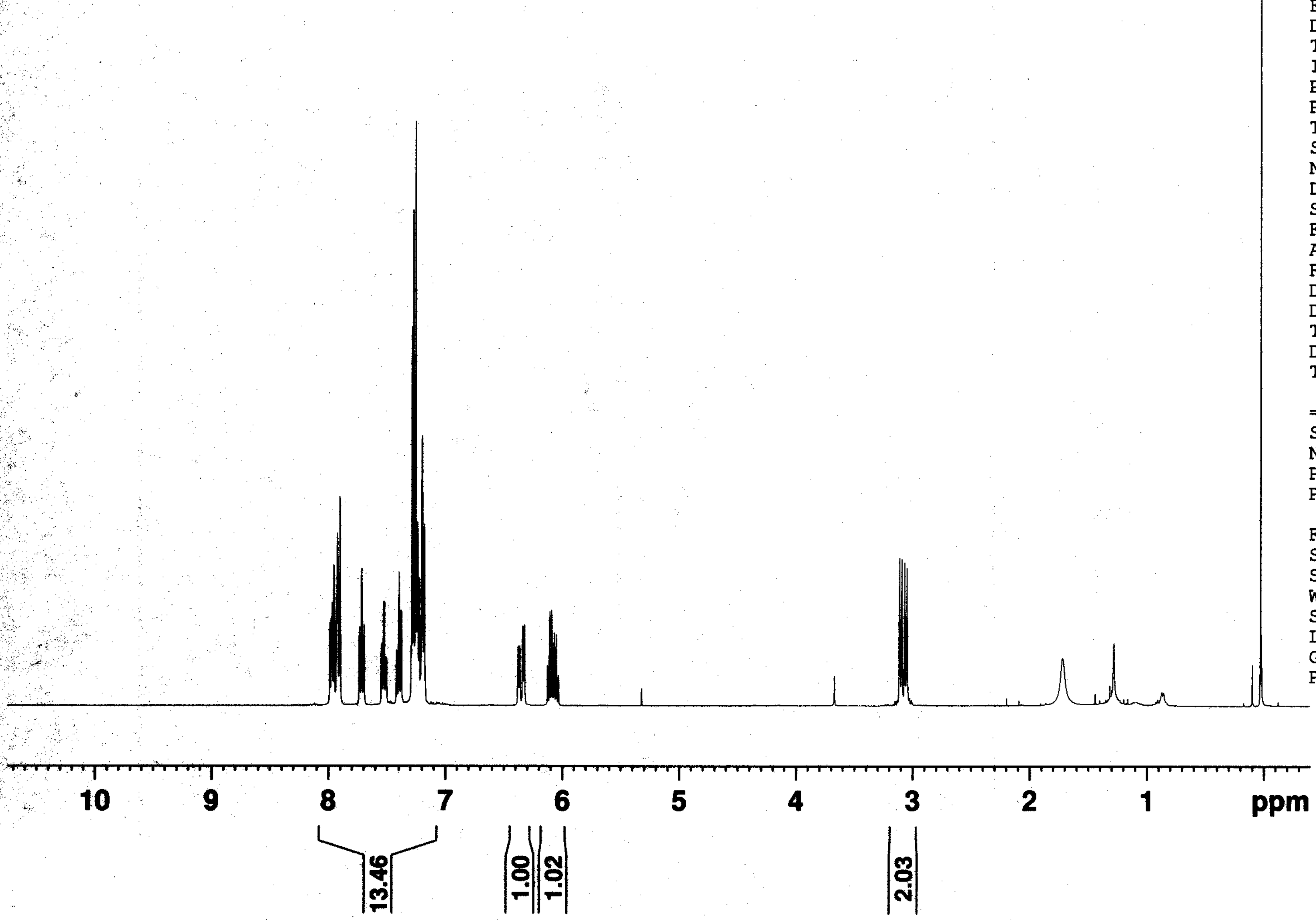


```
Current Data Parameters
NAME                AFL005
EXPNO                    6
PROCNO                   1

F2 - Acquisition Parameters
Date_             20150814
Time                 13.49
INSTRUM              spect
PROBHD   5 mm PABBO BB/
PULPROG               zg30
TD                   65536
SOLVENT              CDCl3
NS                      16
DS                       2
SWH              8012.820 Hz
FIDRES           0.122266 Hz
AQ              4.0894465 sec
RG                  144.26
DW                  62.400 usec
DE                    6.50 usec
TE                   295.0 K
D1              1.00000000 sec
TD0                      1

======== CHANNEL f1 ========
SFO1        400.1324710 MHz
NUC1                    1H
P1                   10.00 usec
PLW1         25.00300026 W

F2 - Processing parameters
SI                   65536
SF          400.1300000 MHz
WDW                     EM
SSB      0
LB                    0.30 Hz
GB       0
PC                    1.00
```

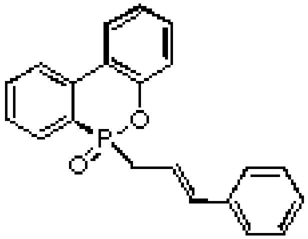

Table 2, entry 11
^{13}C NMR

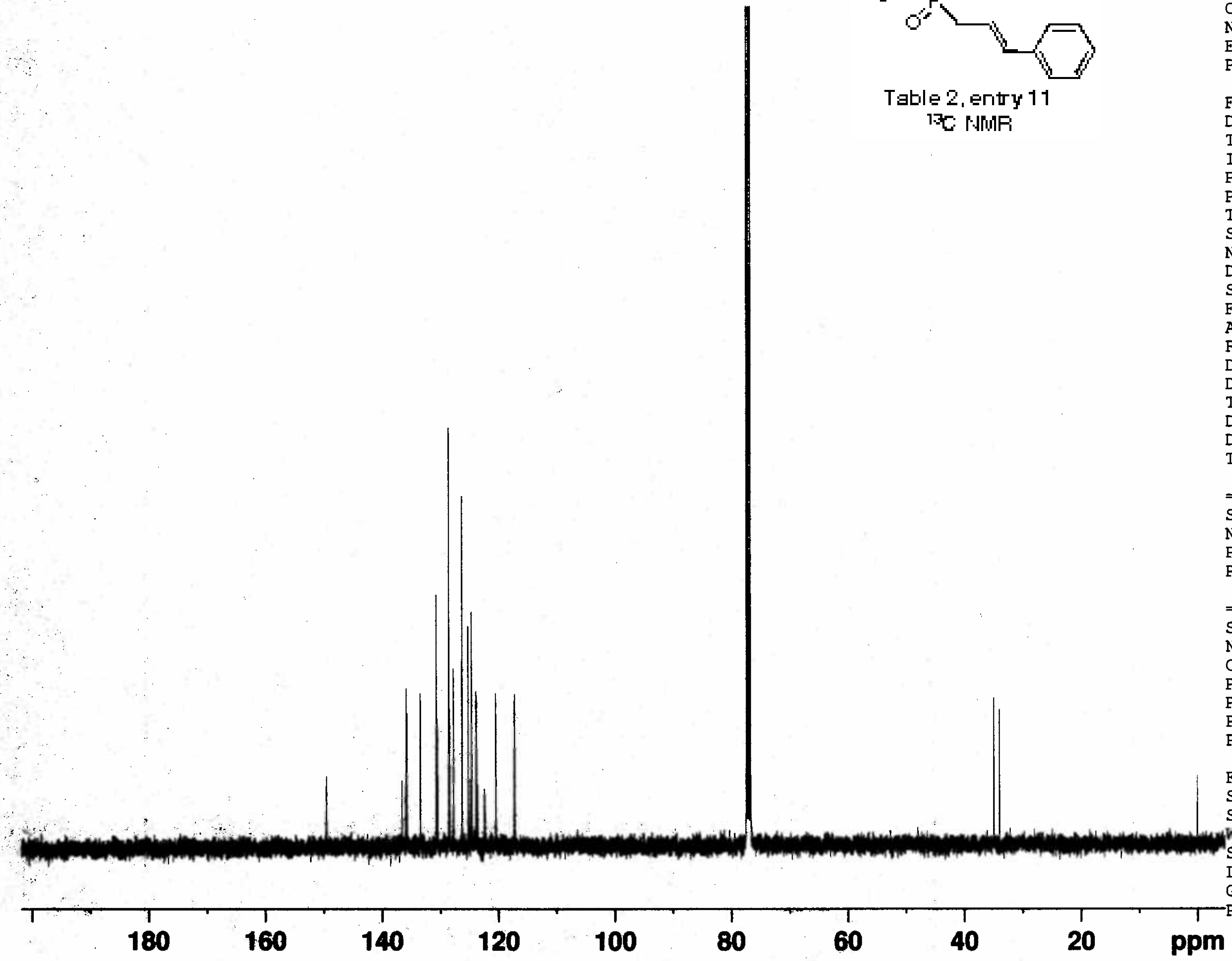


```
Current Data Parameters
NAME              AFL005
EXPNO                  7
PROCNO                 1

F2 - Acquisition Parameters
Date_           20150814
Time               14.26
INSTRUM            spect
PROBHD   5 mm PABBO BB/
PULPROG           zgpg30
TD                 65536
SOLVENT            CDCl3
NS                   553
DS                     4
SWH            24038.461 Hz
FIDRES          0.366798 Hz
AQ             1.3631488 sec
RG                203.57
DW                20.800 usec
DE                  6.50 usec
TE                 295.9 K
D1            2.00000000 sec
D11           0.03000000 sec
TD0                    1

======== CHANNEL f1 ========
SFO1         100.6228293 MHz
NUC1                 13C
P1                 10.00 usec
PLW1         45.00000000 W

======== CHANNEL f2 ========
SFO2         400.1316005 MHz
NUC2                  1H
CPDPRG[2         waltz16
PCPD2              90.00 usec
PLW2         10.00000000 W
PLW12         0.31604999 W
PLW13         0.25600001 W

F2 - Processing parameters
SI                 32768
SF           100.6127685 MHz
WDW                   EM
SSB      0
LB                  1.00 Hz
GB       0
PC                  1.40
```

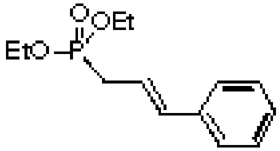

Table 2, entry 12

$^{31}P/^{1}H$ NMR decoupled

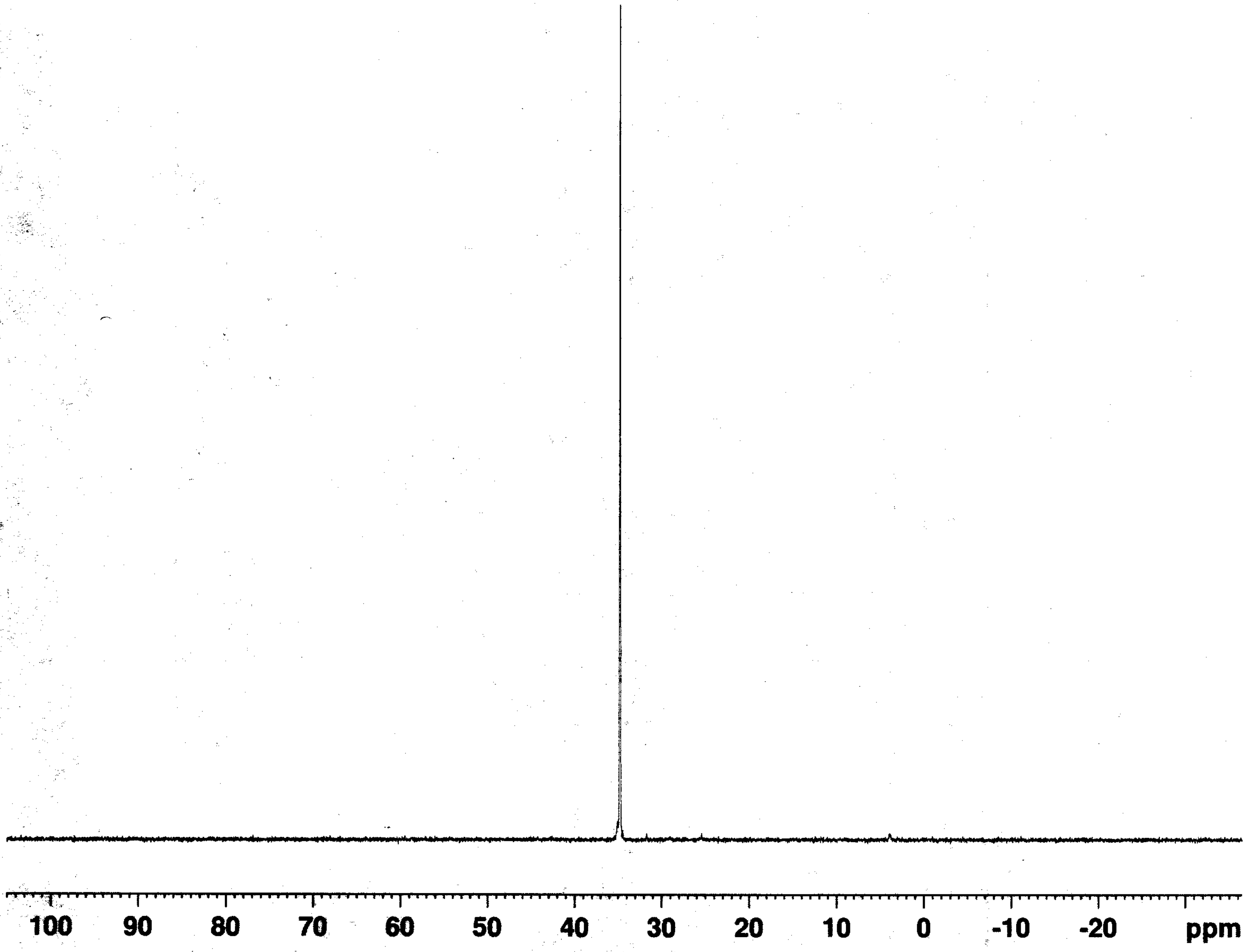


```
Current Data Parameters
NAME              AFL004
EXPNO                  1
PROCNO                 1

F2 - Acquisition Parameters
Date_           20150608
Time               14.10
INSTRUM            spect
PROBHD   5 mm PABBO BB/
PULPROG          zgpg30
TD                 65536
SOLVENT            CDCl3
NS                    16
DS                     4
SWH           64102.563 Hz
FIDRES         0.978127 Hz
AQ            0.5111808 sec
RG                203.57
DW                 7.800 usec
DE                  6.50 usec
TE                 294.7 K
D1            2.00000000 sec
D11           0.03000000 sec
TD0                    1

======== CHANNEL f1 ========
SFO1        161.9674942 MHz
NUC1                 31P
P1                 14.25 usec
PLW1         15.00000000 W

======== CHANNEL f2 ========
SFO2        400.1316005 MHz
NUC2                  1H
CPDPRG[2         waltz16
PCPD2              90.00 usec
PLW2         10.00000000 W
PLW12         0.31604999 W
PLW13         0.25600001 W

F2 - Processing parameters
SI                 32768
SF          161.9755930 MHz
WDW                   EM
SSB      0
LB                  1.00 Hz
GB       0
PC                  1.40
```

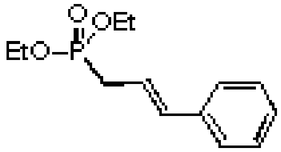

Table 2, entry 12

$^{31}P/^{1}H$ NMR coupled

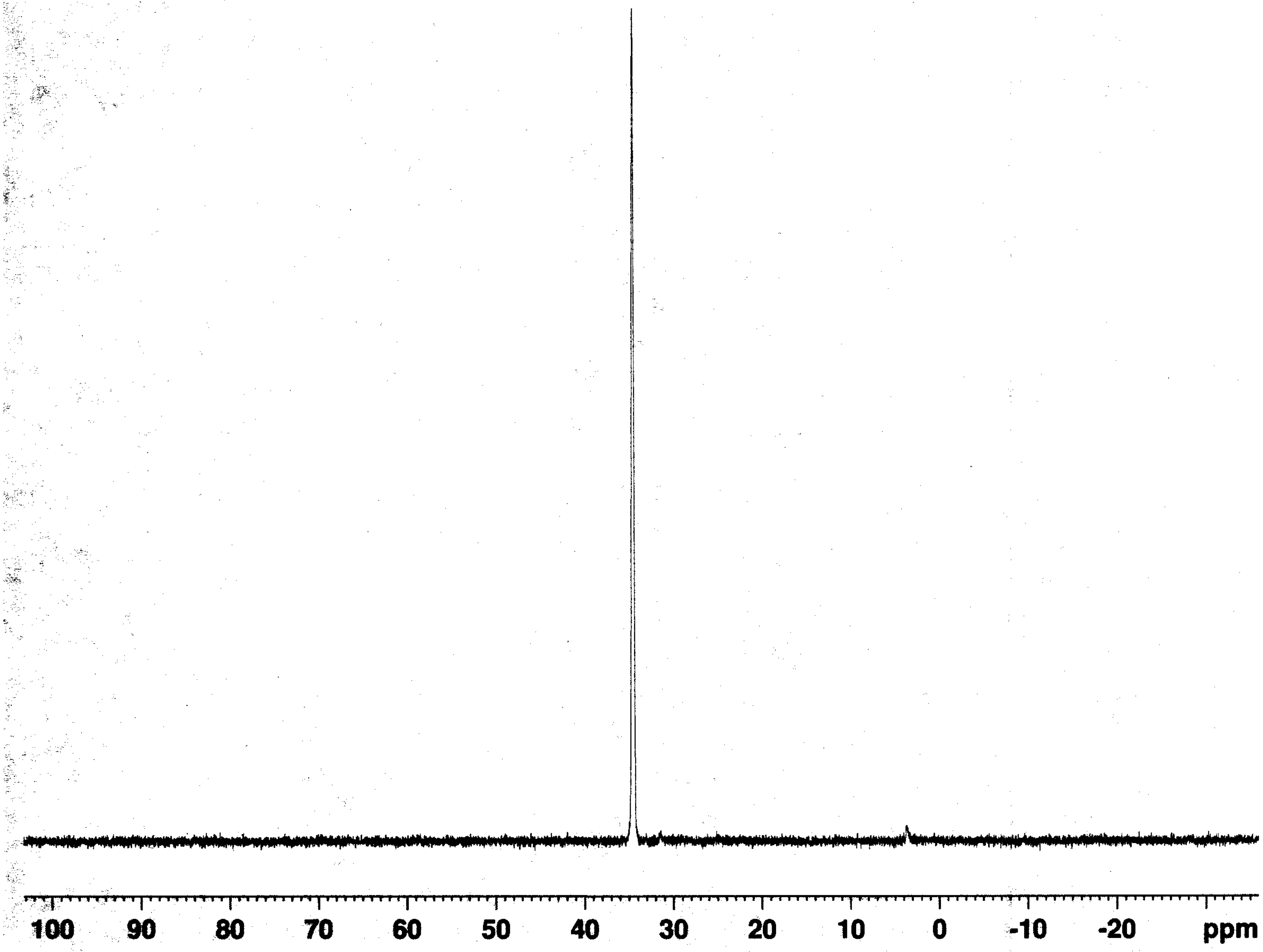

Current Data Parameters

NAME	AFL004
EXPNO	2
PROCNO	1

F2 - Acquisition Parameters

Date_	20150608
Time	14.12
INSTRUM	spect
PROBHD	5 mm PABBO BB/
PULPROG	zg30
TD	65536
SOLVENT	CDCl3
NS	32
DS	4
SWH	64102.563 Hz
FIDRES	0.978127 Hz
AQ	0.5111808 sec
RG	203.57
DW	7.800 usec
DE	6.50 usec
TE	294.2 K
D1	2.00000000 sec
TD0	1

======== CHANNEL f1 ========

SFO1	161.9674942 MHz
NUC1	31P
P1	14.25 usec
PLW1	15.00000000 W

F2 - Processing parameters

SI	32768
SF	161.9755930 MHz
WDW	EM
SSB	0
LB	1.00 Hz
GB	0
PC	1.40

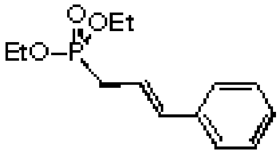

Table 2, entry 12
^{1}H NMR

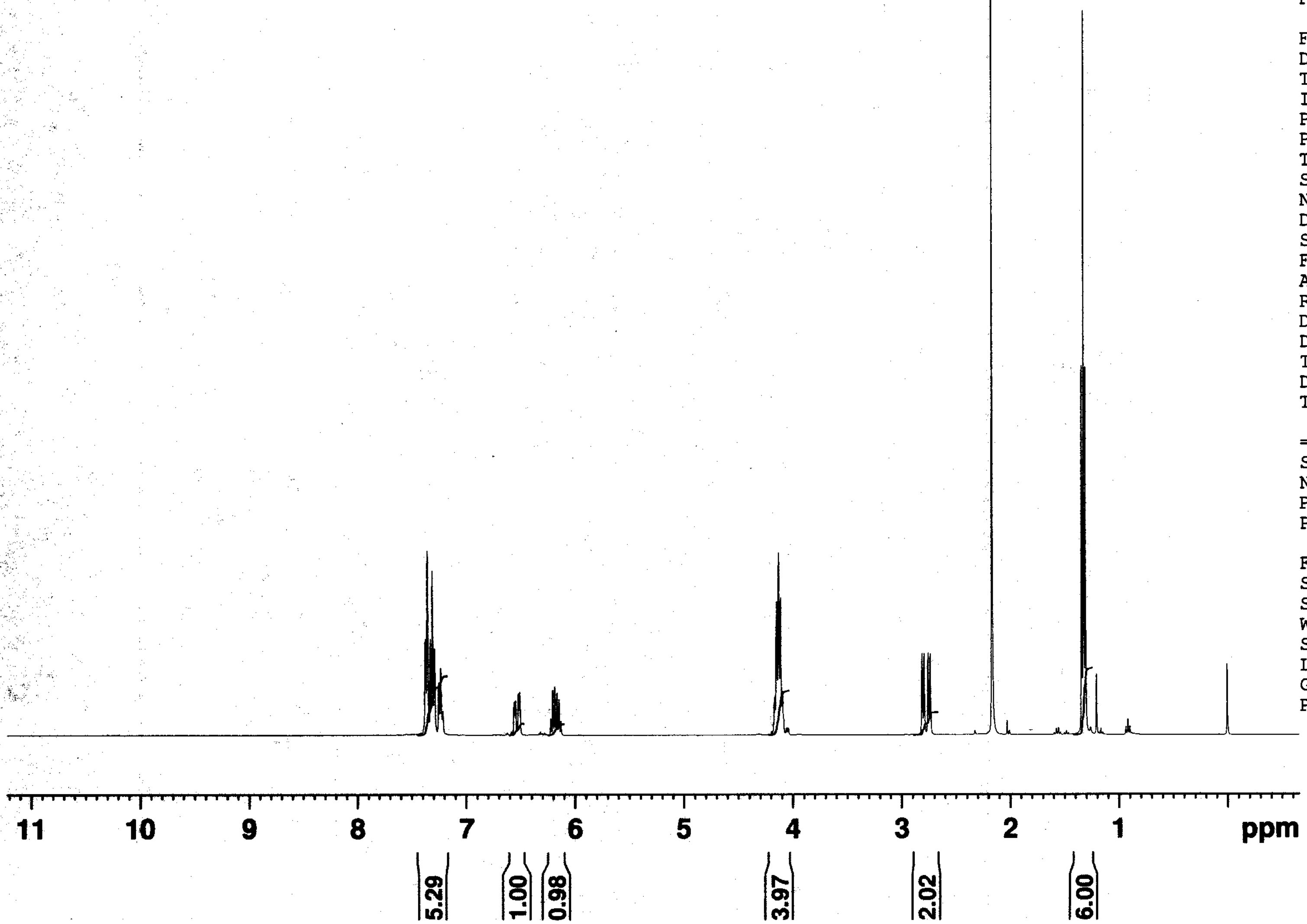

Current Data Parameters
NAME AFL004aftercolumn
EXPNO 1
PROCNO 1

F2 - Acquisition Parameters
Date_ 20150611
Time 11.21
INSTRUM spect
PROBHD 5 mm PABBO BB/
PULPROG zg30
TD 65536
SOLVENT CDCl3
NS 16
DS 2
SWH 8012.820 Hz
FIDRES 0.122266 Hz
AQ 4.0894465 sec
RG 32.38
DW 62.400 usec
DE 6.50 usec
TE 293.6 K
D1 1.00000000 sec
TD0 1

======== CHANNEL f1 ========
SFO1 400.1324710 MHz
NUC1 1H
P1 10.00 usec
PLW1 25.00300026 W

F2 - Processing parameters
SI 65536
SF 400.1300000 MHz
WDW EM
SSB 0
LB 0.30 Hz
GB 0
PC 1.00

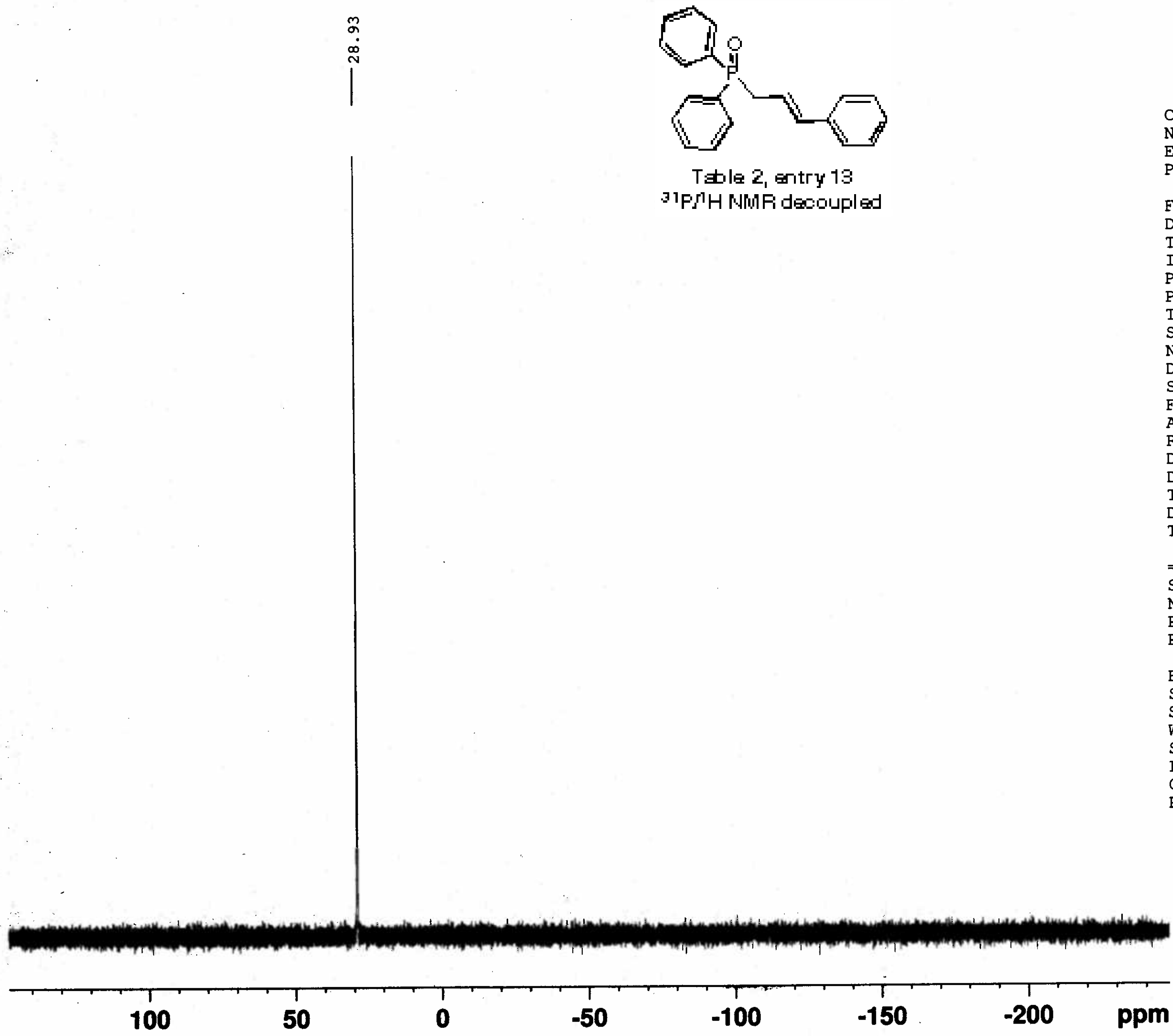


```
Current Data Parameters
NAME                  AFL010
EXPNO                      1
PROCNO                     1

F2 - Acquisition Parameters
Date_               20150615
Time                   15.32
INSTRUM                spect
PROBHD     5 mm PABBO BB/
PULPROG                 zg30
TD                     65536
SOLVENT              Acetone
NS                        32
DS                         4
SWH               64102.563 Hz
FIDRES             0.978127 Hz
AQ                0.5111808 sec
RG                    203.57
DW                     7.800 usec
DE                      6.50 usec
TE                     294.3 K
D1                2.00000000 sec
TD0                        1

======== CHANNEL f1 ========
SFO1            161.9674942 MHz
NUC1                     31P
P1                     14.25 usec
PLW1             15.00000000 W

F2 - Processing parameters
SI                     32768
SF               161.9755930 MHz
WDW                       EM
SSB         0
LB                      1.00 Hz
GB          0
PC                      1.40
```

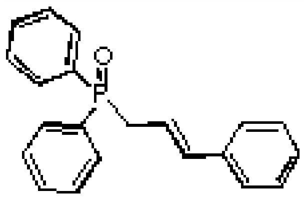

Table 2, entry 13
$^{31}P/^{1}H$ NMR coupled

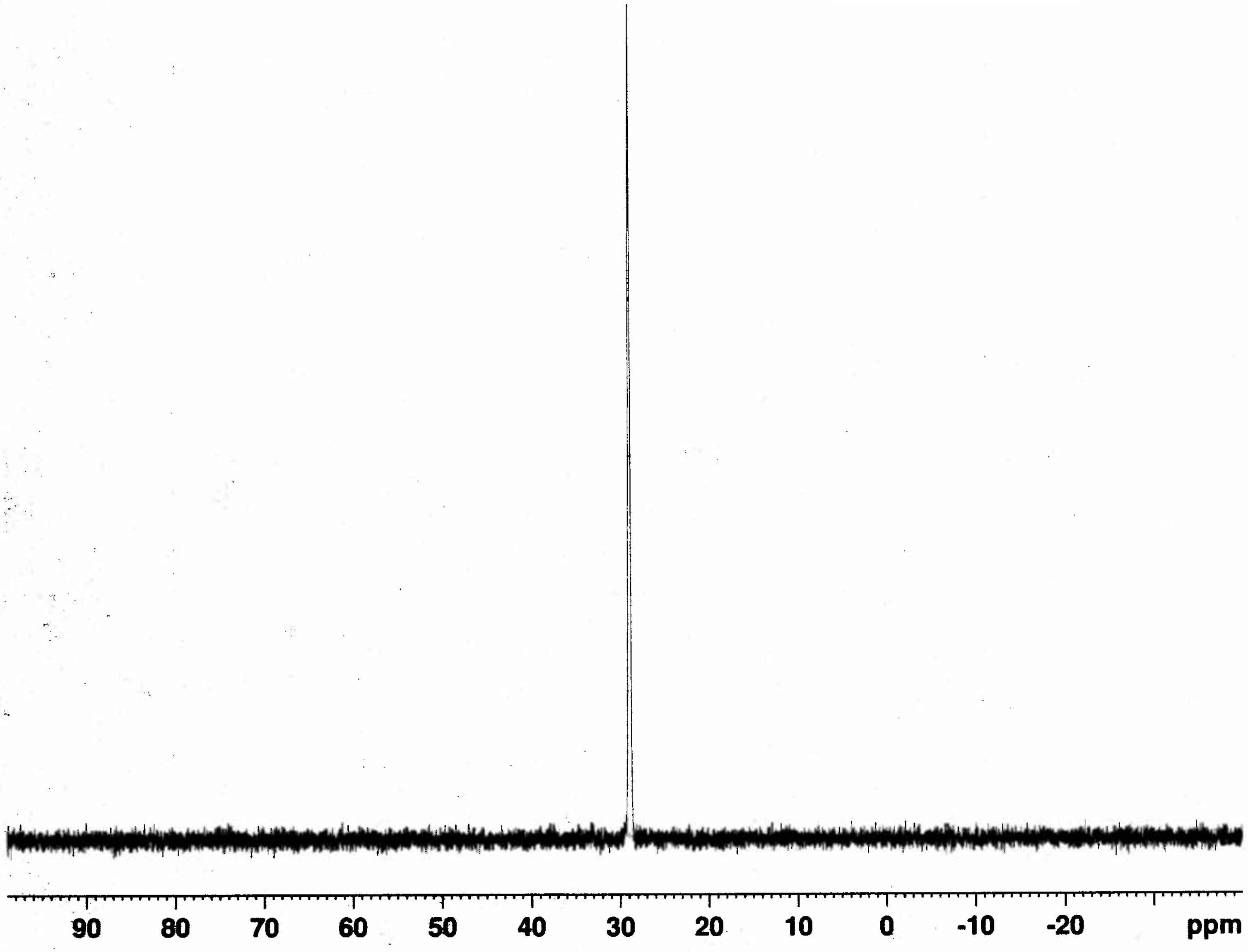


```
Current Data Parameters
NAME              AFL010
EXPNO                  1
PROCNO                 1

F2 - Acquisition Parameters
Date_           20150615
Time               15.32
INSTRUM            spect
PROBHD    5 mm PABBO BB/
PULPROG             zg30
TD                 65536
SOLVENT          Acetone
NS                    32
DS                     4
SWH            64102.563 Hz
FIDRES          0.978127 Hz
AQ             0.5111808 sec
RG                203.57
DW                 7.800 usec
DE                  6.50 usec
TE                 294.3 K
D1            2.00000000 sec
TD0                    1

======== CHANNEL f1 ========
SFO1         161.9674942 MHz
NUC1                 31P
P1                 14.25 usec
PLW1         15.00000000 W

F2 - Processing parameters
SI                 32768
SF           161.9755930 MHz
WDW                   EM
SSB       0
LB                  1.00 Hz
GB        0
PC                  1.40
```

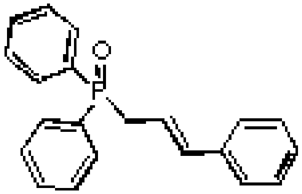

Table 2, entry 13
^{1}H NMR

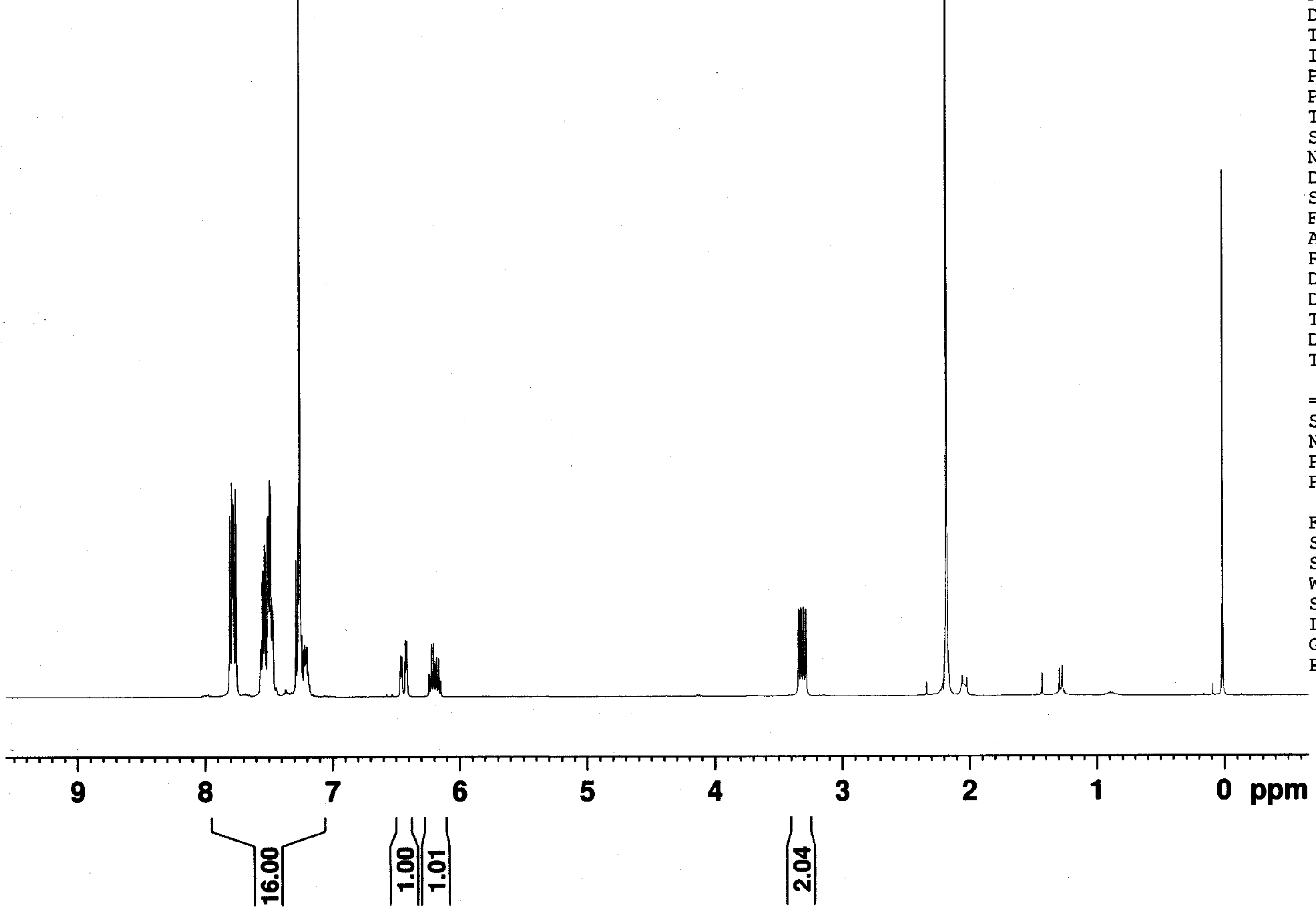


```
Current Data Parameters
NAME               AFL010
EXPNO                   2
PROCNO                  1

F2 - Acquisition Parameters
Date_            20150615
Time                15.56
INSTRUM             spect
PROBHD   5 mm PABBO BB/
PULPROG              zg30
TD                  65536
SOLVENT             CDCl3
NS                     15
DS                      2
SWH              8012.820 Hz
FIDRES           0.122266 Hz
AQ              4.0894465 sec
RG                  81.67
DW                 62.400 usec
DE                   6.50 usec
TE                  294.4 K
D1             1.00000000 sec
TD0                     1

======== CHANNEL f1 ========
SFO1          400.1324710 MHz
NUC1                   1H
P1                  10.00 usec
PLW1          25.00300026 W

F2 - Processing parameters
SI                  65536
SF            400.1300000 MHz
WDW                    EM
SSB      0
LB                   0.30 Hz
GB       0
PC                   1.00
```

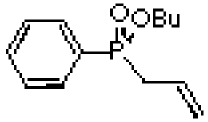

Table 3, entry 1

$^{31}P/^{1}H$ NMR decoupled

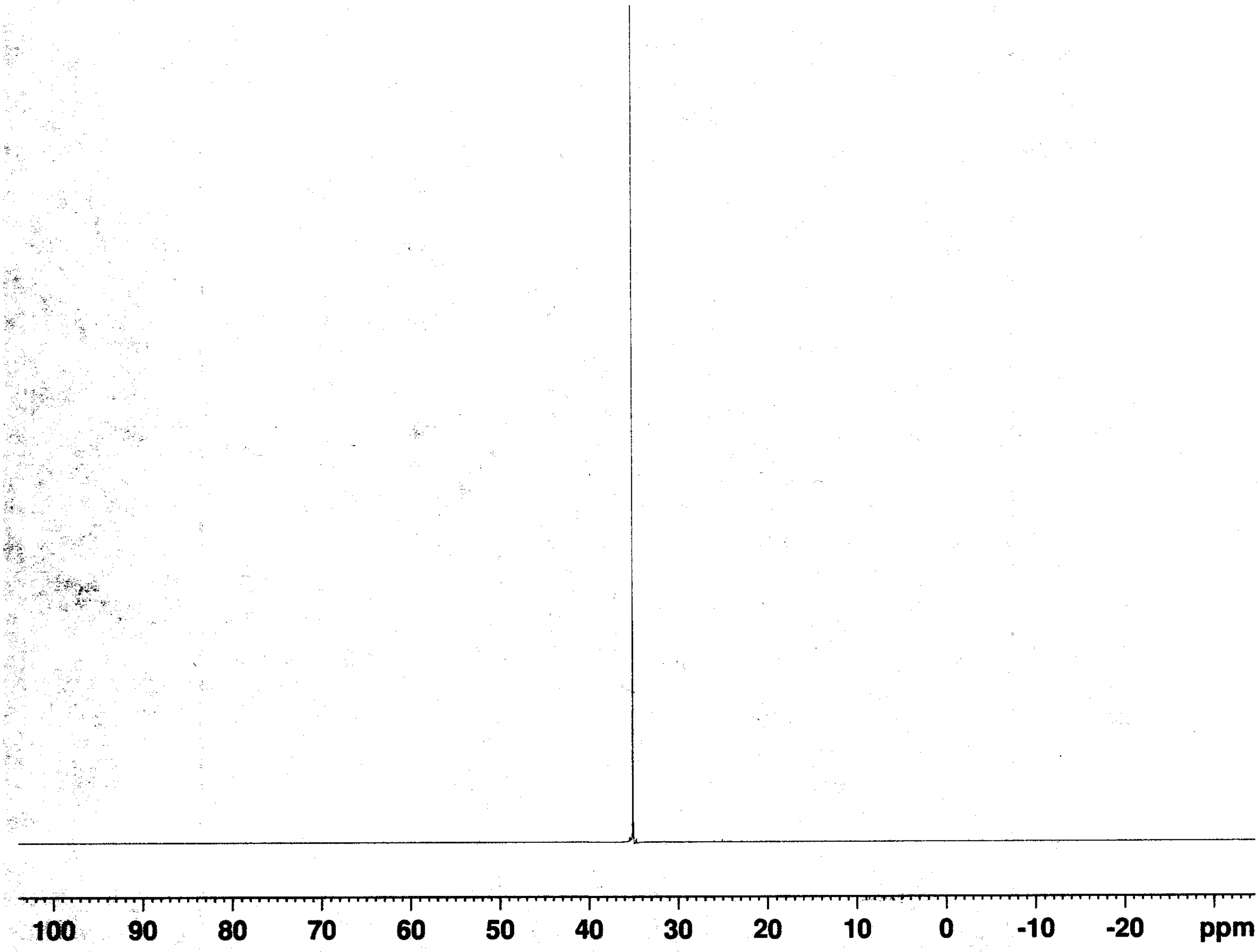

Current Data Parameters
NAME AFL013
EXPNO 10
PROCNO 1

F2 - Acquisition Parameters
Date_ 20150825
Time 10.53
INSTRUM spect
PROBHD 5 mm PABBO BB/
PULPROG zgpg30
TD 65536
SOLVENT Acetone
NS 6
DS 4
SWH 64102.563 Hz
FIDRES 0.978127 Hz
AQ 0.5111808 sec
RG 203.57
DW 7.800 usec
DE 6.50 usec
TE 294.8 K
D1 2.00000000 sec
D11 0.03000000 sec
TD0 1

======== CHANNEL f1 ========
SFO1 161.9674942 MHz
NUC1 31P
P1 14.25 usec
PLW1 15.00000000 W

======== CHANNEL f2 ========
SFO2 400.1316005 MHz
NUC2 1H
CPDPRG[2 waltz16
PCPD2 90.00 usec
PLW2 10.00000000 W
PLW12 0.31604999 W
PLW13 0.25600001 W

F2 - Processing parameters
SI 32768
SF 161.9755930 MHz
WDW EM
SSB 0
LB 1.00 Hz
GB 0
PC 1.40

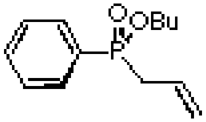

Table 3, entry 1
$^{31}P/^{1}H$ NMR coupled

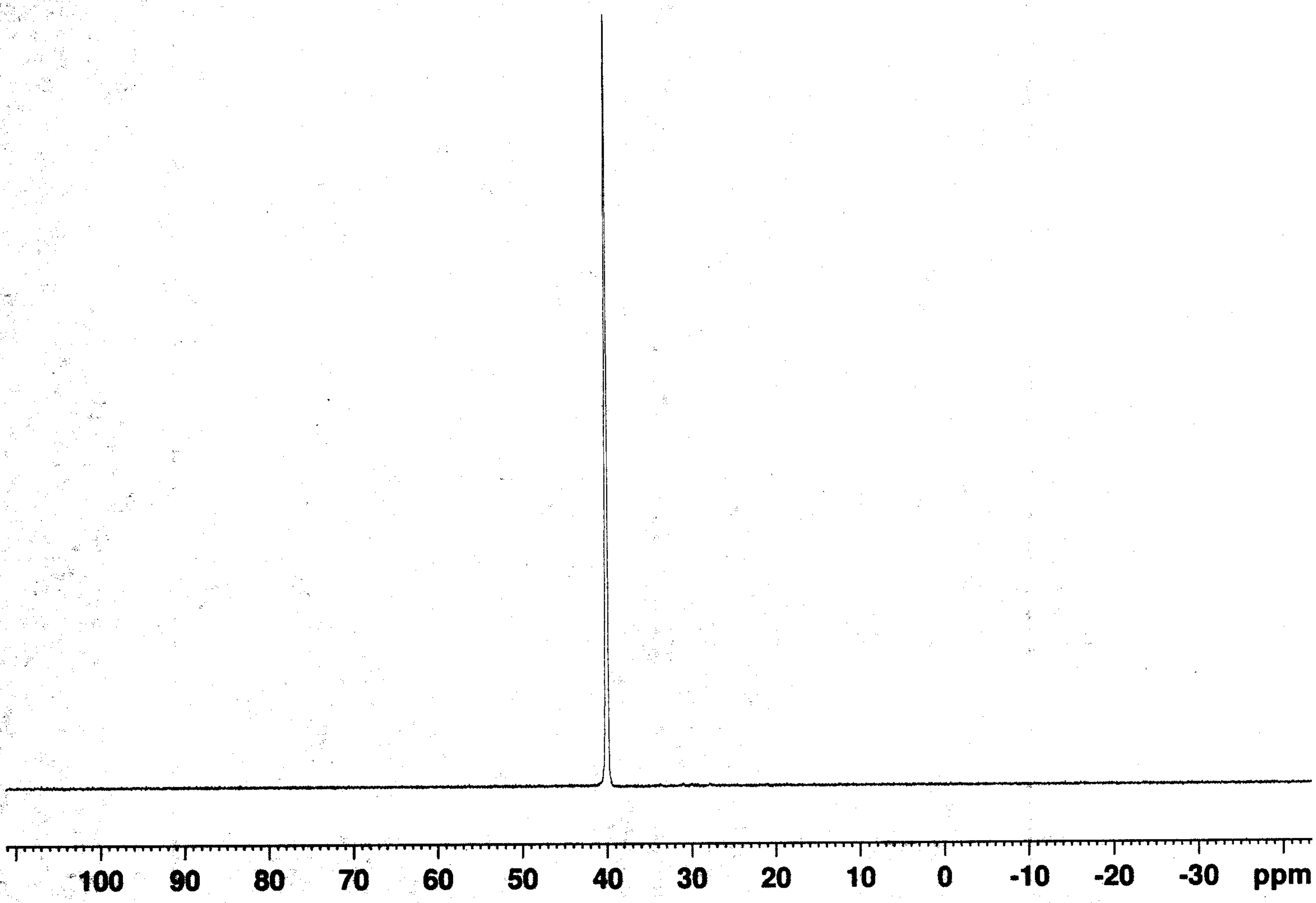

Current Data Parameters
NAME AFL013
EXPNO 3
PROCNO 1

F2 - Acquisition Parameters
Date_ 20150616
Time 9.58
INSTRUM spect
PROBHD 5 mm PABBO BB/
PULPROG zg30
TD 65536
SOLVENT CDCl3
NS 32
DS 4
SWH 64102.563 Hz
FIDRES 0.978127 Hz
AQ 0.5111808 sec
RG 203.57
DW 7.800 usec
DE 6.50 usec
TE 294.1 K
D1 2.00000000 sec
TD0 1

======== CHANNEL f1 ========
SFO1 161.9674942 MHz
NUC1 31P
P1 14.25 usec
PLW1 15.00000000 W

F2 - Processing parameters
SI 32768
SF 161.9755930 MHz
WDW EM
SSB 0
LB 1.00 Hz
GB 0
PC 1.40

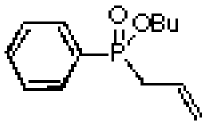

Table 3, entry 1
^{1}H NMR

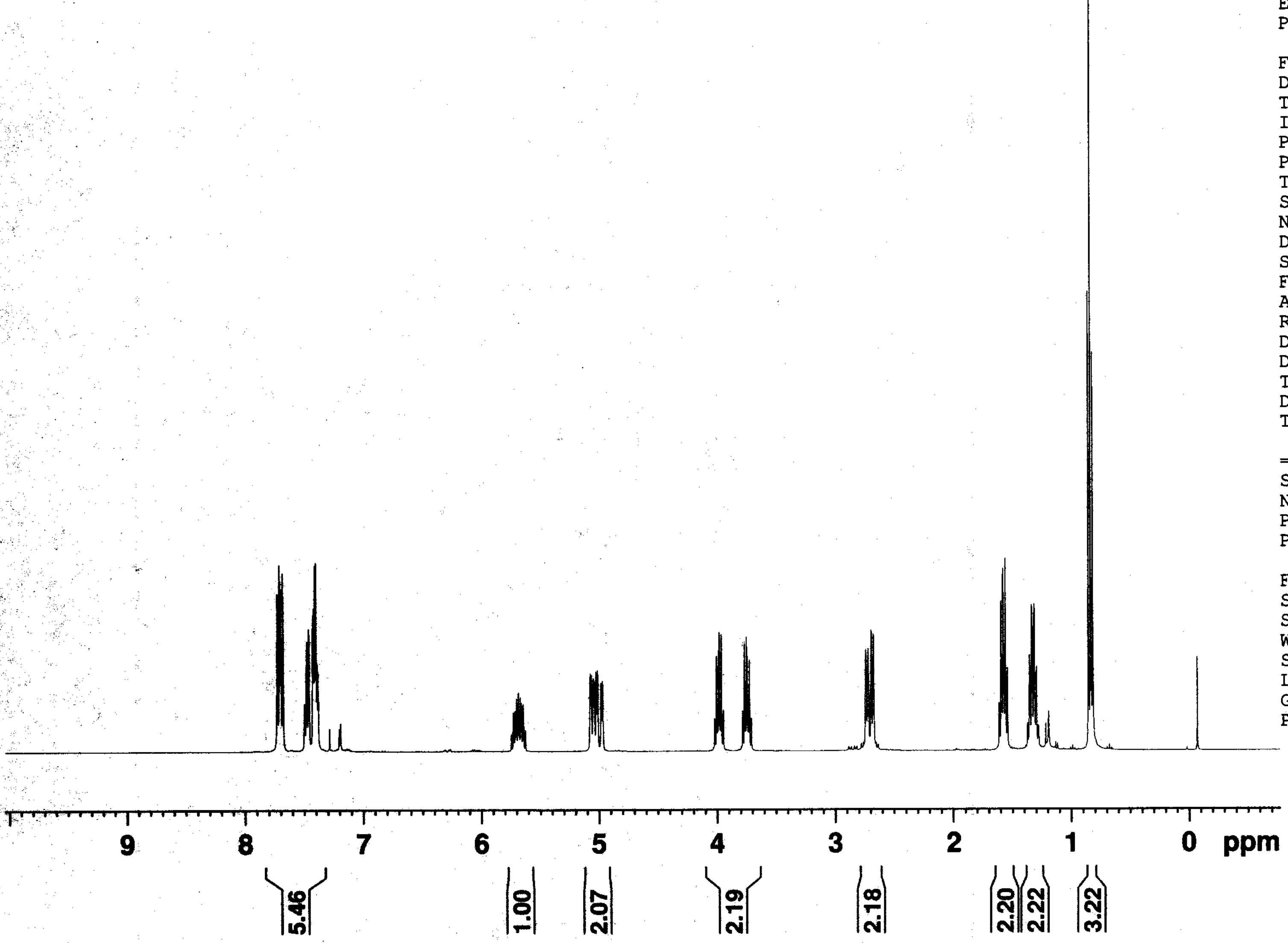


```
Current Data Parameters
NAME                AFL013
EXPNO                    1
PROCNO                   1

F2 - Acquisition Parameters
Date_             20150616
Time                  9.45
INSTRUM              spect
PROBHD   5 mm PABBO BB/
PULPROG               zg30
TD                   65536
SOLVENT              CDCl3
NS                      16
DS                       2
SWH               8012.820 Hz
FIDRES            0.122266 Hz
AQ               4.0894465 sec
RG                   17.56
DW                  62.400 usec
DE                    6.50 usec
TE                   293.8 K
D1              1.00000000 sec
TD0                      1

======== CHANNEL f1 ========
SFO1           400.1324710 MHz
NUC1                    1H
P1                   10.00 usec
PLW1           25.00300026 W

F2 - Processing parameters
SI                   65536
SF             400.1300000 MHz
WDW                     EM
SSB       0
LB                    0.30 Hz
GB        0
PC                    1.00
```

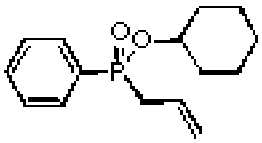

Table 3, entry 2
$^{31}P/^{1}H$ NMR decoupled

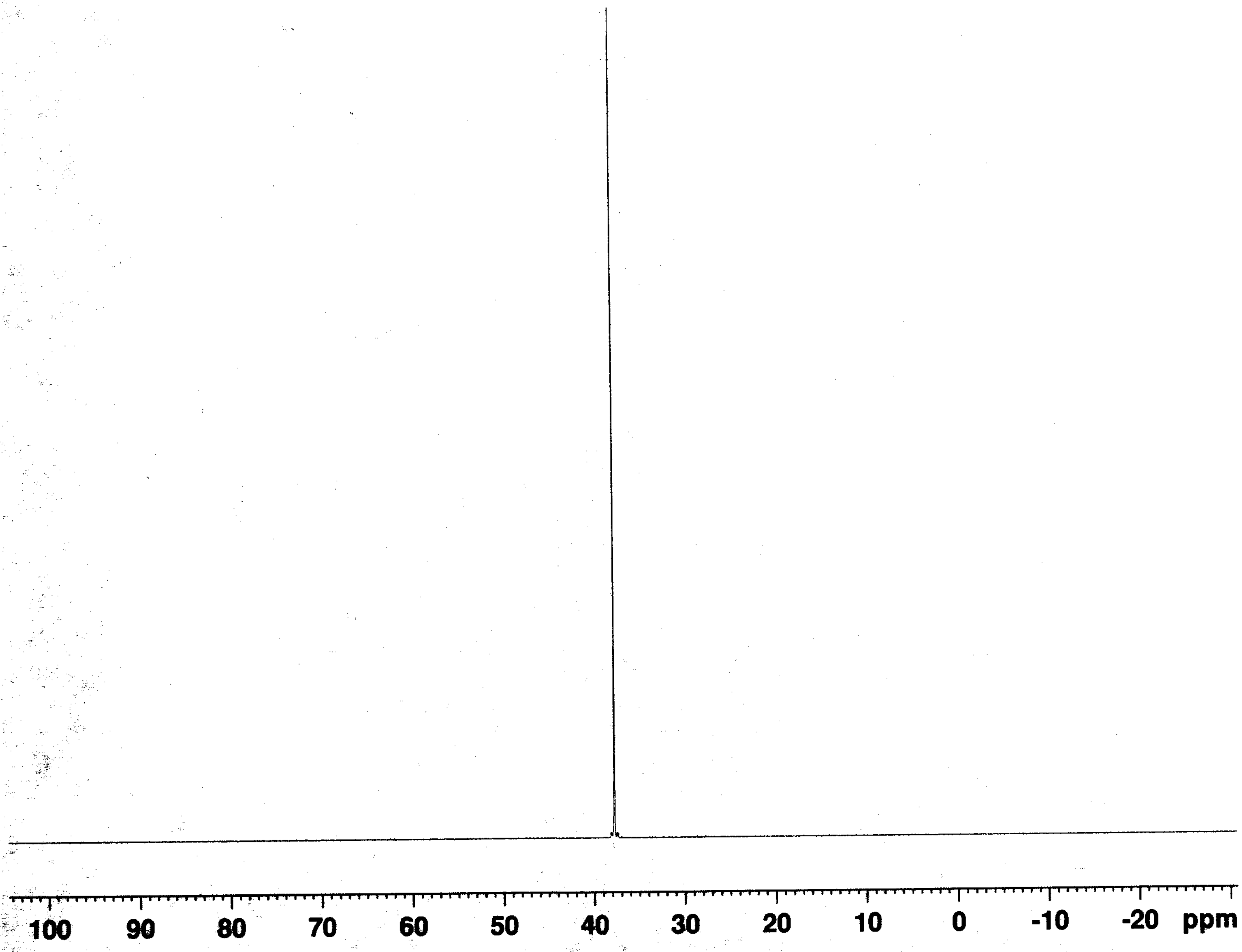

Current Data Parameters

NAME	AFL065
EXPNO	3
PROCNO	1

F2 - Acquisition Parameters

Date_	20150730
Time	11.17
INSTRUM	spect
PROBHD	5 mm PABBO BB/
PULPROG	zgpg30
TD	65536
SOLVENT	Acetone
NS	16
DS	4
SWH	64102.563 Hz
FIDRES	0.978127 Hz
AQ	0.5111808 sec
RG	203.57
DW	7.800 usec
DE	6.50 usec
TE	294.9 K
D1	2.00000000 sec
D11	0.03000000 sec
TD0	1

======== CHANNEL f1 ========

SFO1	161.9674942 MHz
NUC1	31P
P1	14.25 usec
PLW1	15.00000000 W

======== CHANNEL f2 ========

SFO2	400.1316005 MHz
NUC2	1H
CPDPRG[2	waltz16
PCPD2	90.00 usec
PLW2	10.00000000 W
PLW12	0.31604999 W
PLW13	0.25600001 W

F2 - Processing parameters

SI	32768
SF	161.9755930 MHz
WDW	EM
SSB	0
LB	1.00 Hz
GB	0
PC	1.40

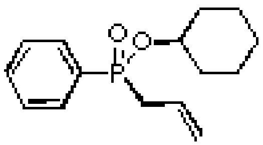

Table 3, entry 2
$^{31}P/^{1}H$ NMR coupled

Current Data Parameters
NAME AFL065
EXPNO 4
PROCNO 1

F2 - Acquisition Parameters
Date_ 20150730
Time 11.19
INSTRUM spect
PROBHD 5 mm PABBO BB/
PULPROG zg30
TD 65536
SOLVENT Acetone
NS 21
DS 4
SWH 64102.563 Hz
FIDRES 0.978127 Hz
AQ 0.5111808 sec
RG 203.57
DW 7.800 usec
DE 6.50 usec
TE 294.5 K
D1 2.00000000 sec
TD0 1

======== CHANNEL f1 ========
SFO1 161.9674942 MHz
NUC1 31P
P1 14.25 usec
PLW1 15.00000000 W

F2 - Processing parameters
SI 32768
SF 161.9755930 MHz
WDW EM
SSB 0
LB 1.00 Hz
GB 0
PC 1.40

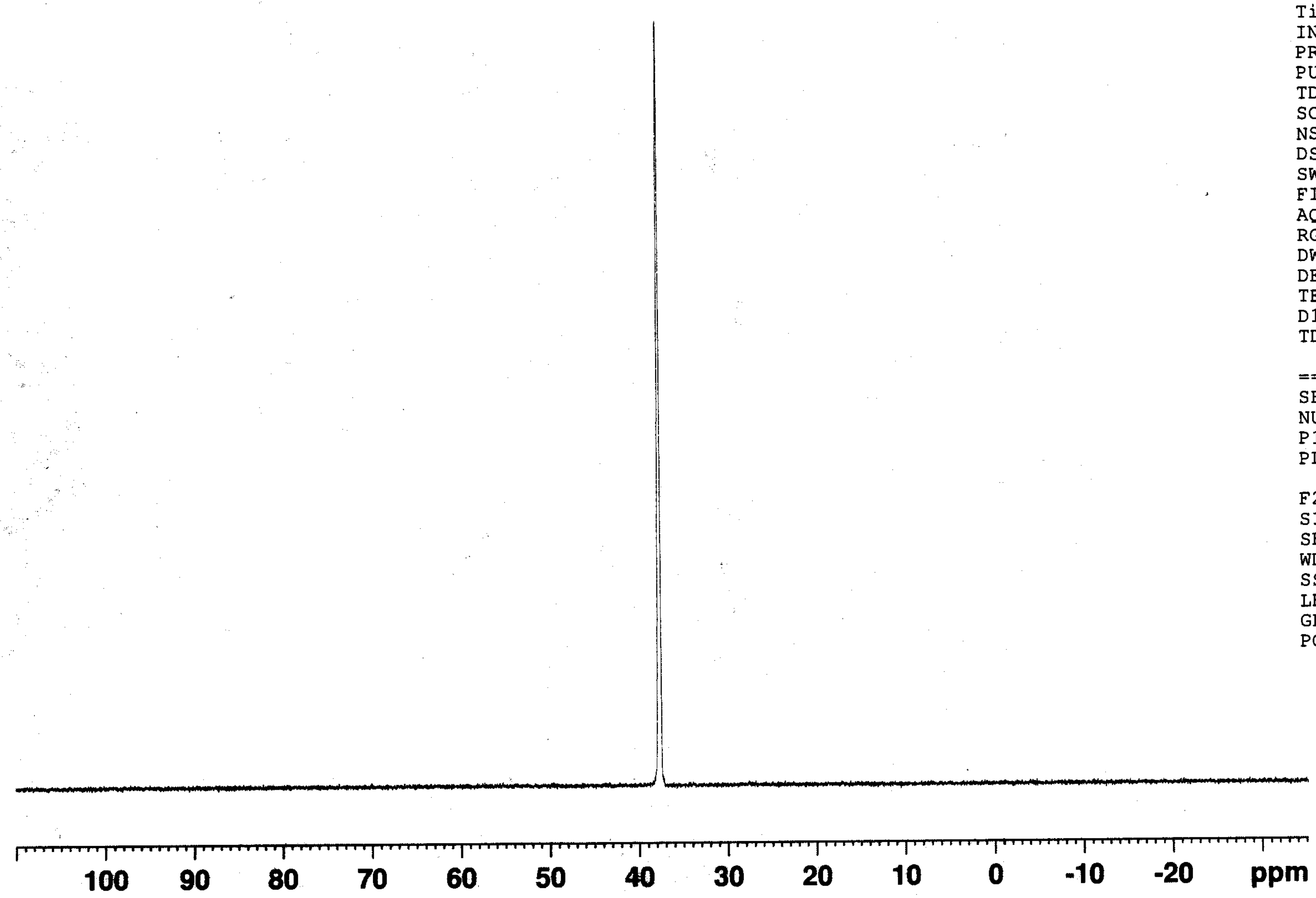

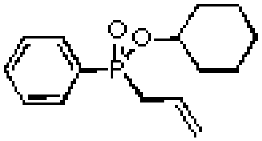

Table 3, entry 2
^1H NMR

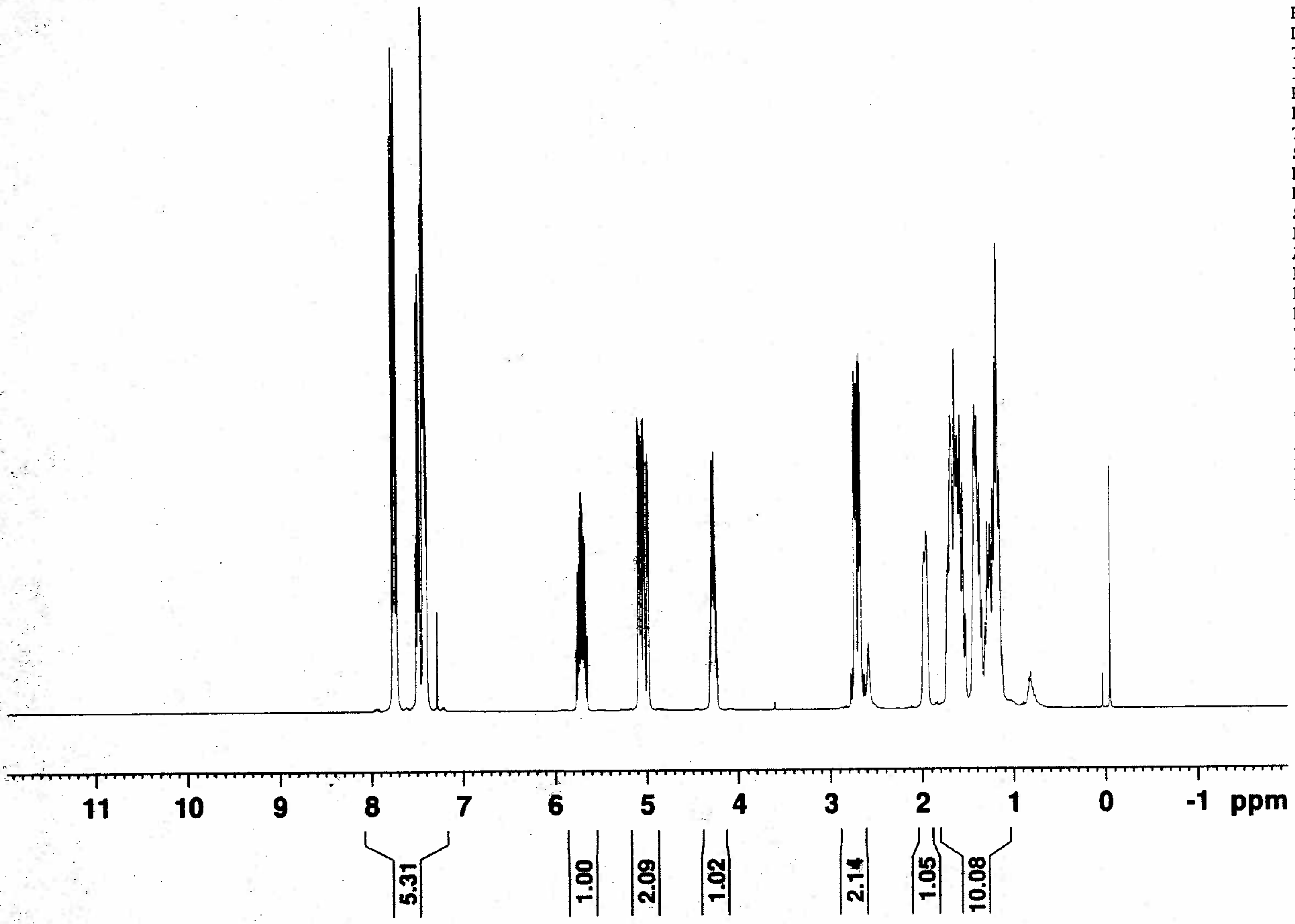

Current Data Parameters
NAME AFL065
EXPNO 5
PROCNO 1

F2 - Acquisition Parameters
Date_ 20150730
Time 11.25
INSTRUM spect
PROBHD 5 mm PABBO BB/
PULPROG zg30
TD 65536
SOLVENT CDCl3
NS 11
DS 2
SWH 8012.820 Hz
FIDRES 0.122266 Hz
AQ 4.0894465 sec
RG 18.98
DW 62.400 usec
DE 6.50 usec
TE 294.5 K
D1 1.00000000 sec
TD0 1

======== CHANNEL f1 ========
SFO1 400.1324710 MHz
NUC1 1H
P1 10.00 usec
PLW1 25.00300026 W

F2 - Processing parameters
SI 65536
SF 400.1300000 MHz
WDW EM
SSB 0
LB 0.60 Hz
GB 0
PC 1.00

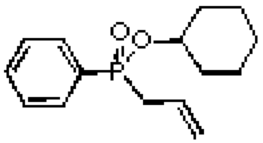

Table 3, entry 2

^{13}C NMR

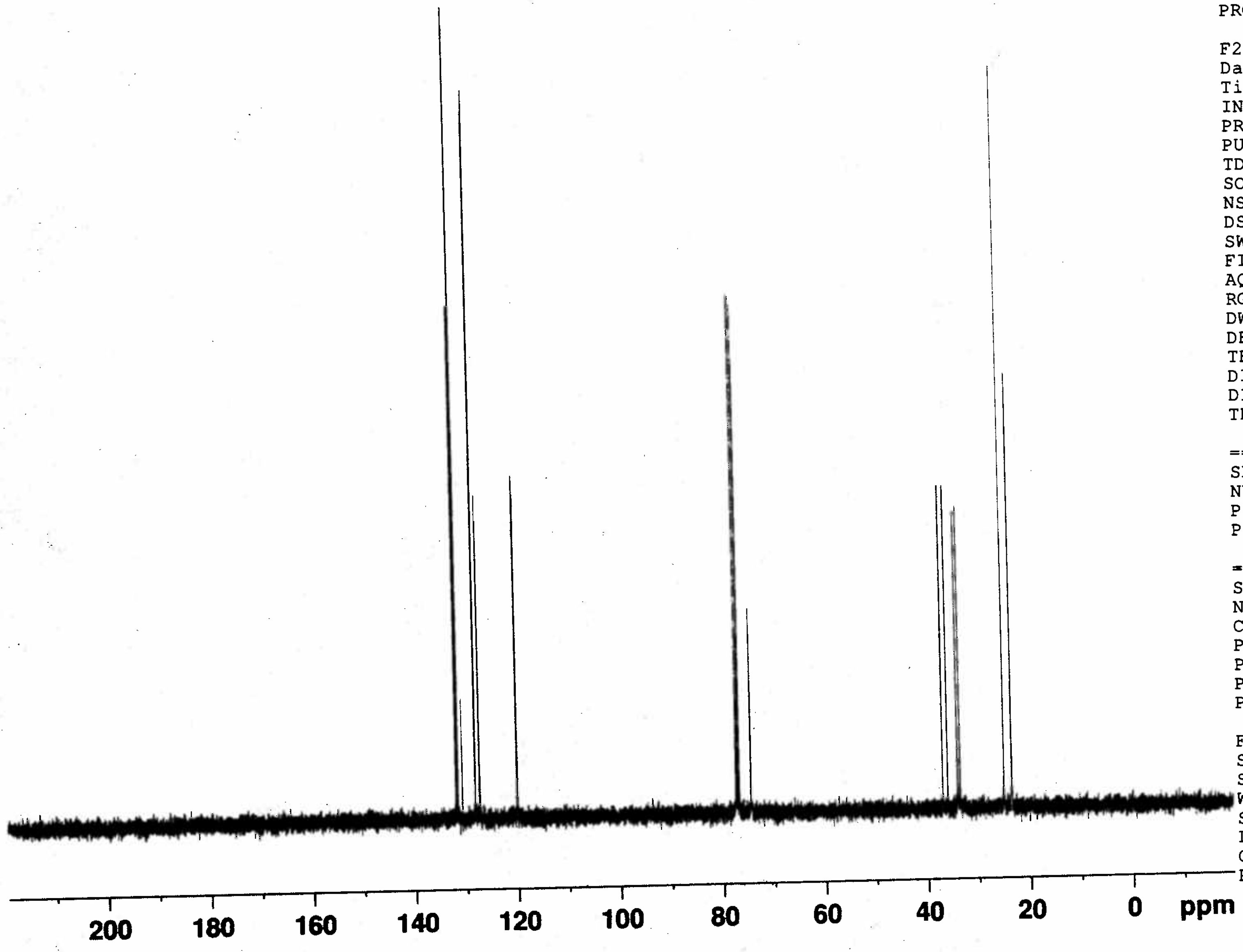


```
Current Data Parameters
NAME                 AFL065
EXPNO                     6
PROCNO                    1

F2 - Acquisition Parameters
Date_              20150730
Time                  11.31
INSTRUM               spect
PROBHD     5 mm PABBO BB/
PULPROG             zgpg30
TD                   65536
SOLVENT              CDCl3
NS                      36
DS                       4
SWH              24038.461 Hz
FIDRES            0.366798 Hz
AQ               1.3631488 sec
RG                  203.57
DW                  20.800 usec
DE                    6.50 usec
TE                   295.2 K
D1              2.00000000 sec
D11             0.03000000 sec
TD0                      1

======== CHANNEL f1 ========
SFO1           100.6228293 MHz
NUC1                   13C
P1                   10.00 usec
PLW1           45.00000000 W

======== CHANNEL f2 ========
SFO2           400.1316005 MHz
NUC2                    1H
CPDPRG[2           waltz16
PCPD2                90.00 usec
PLW2           10.00000000 W
PLW12           0.31604999 W
PLW13           0.25600001 W

F2 - Processing parameters
SI                   32768
SF             100.6127685 MHz
WDW                     EM
SSB       0
LB                    1.00 Hz
GB        0
PC                    1.40
```

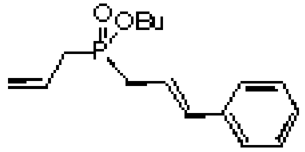

Table 3, entry 3

$^{31}P/^{1}H$ NMR decoupled

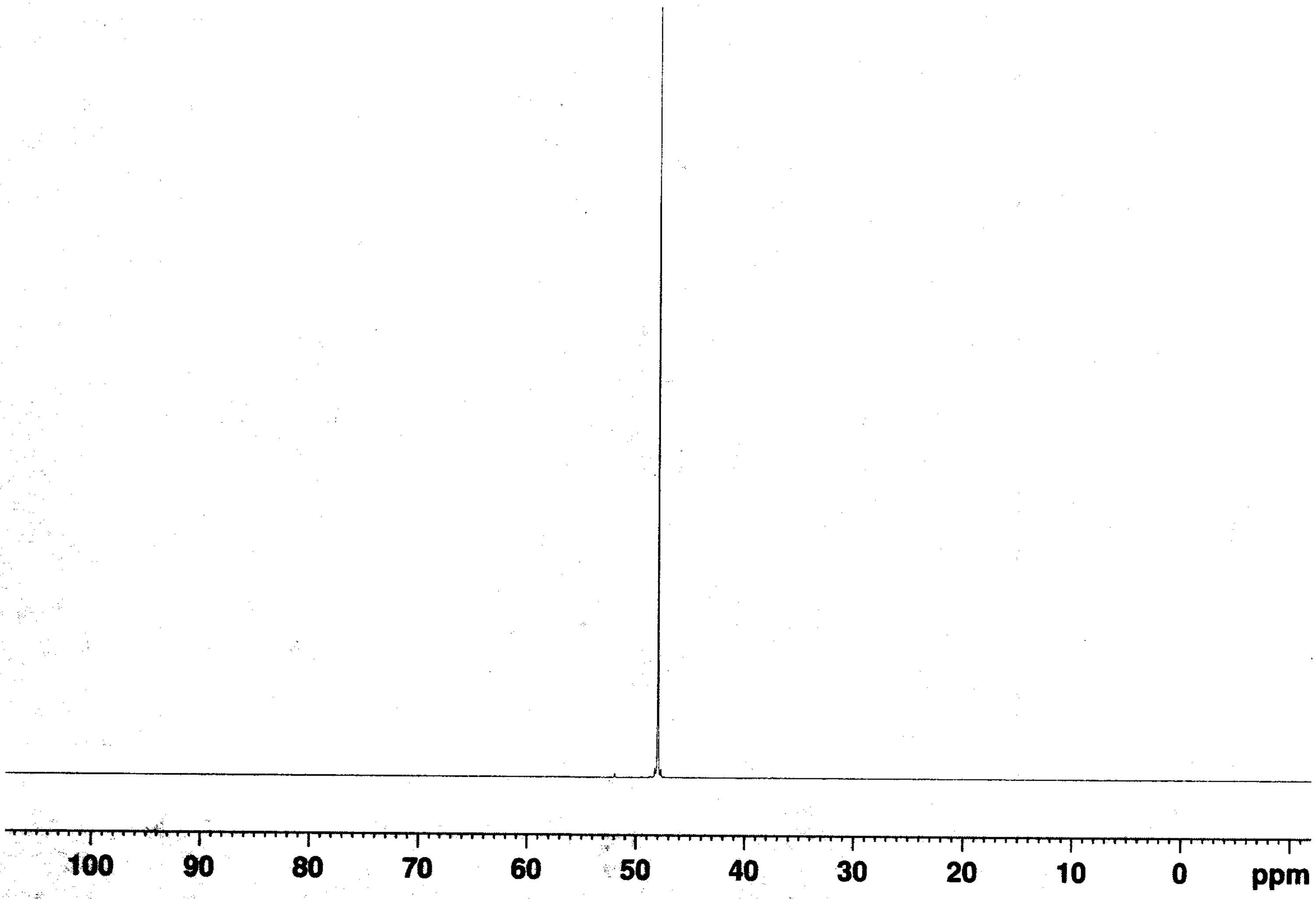

Current Data Parameters
NAME AFL072
EXPNO 3
PROCNO 1

F2 - Acquisition Parameters
Date_ 20150804
Time 14.20
INSTRUM spect
PROBHD 5 mm PABBO BB/
PULPROG zgpg30
TD 65536
SOLVENT Acetone
NS 16
DS 4
SWH 64102.563 Hz
FIDRES 0.978127 Hz
AQ 0.5111808 sec
RG 203.57
DW 7.800 usec
DE 6.50 usec
TE 295.5 K
D1 2.00000000 sec
D11 0.03000000 sec
TD0 1

======== CHANNEL f1 ========
SFO1 161.9674942 MHz
NUC1 31P
P1 14.25 usec
PLW1 15.00000000 W

======== CHANNEL f2 ========
SFO2 400.1316005 MHz
NUC2 1H
CPDPRG[2 waltz16
PCPD2 90.00 usec
PLW2 10.00000000 W
PLW12 0.31604999 W
PLW13 0.25600001 W

F2 - Processing parameters
SI 32768
SF 161.9755930 MHz
WDW EM
SSB 0
LB 1.00 Hz
GB 0
PC 1.40

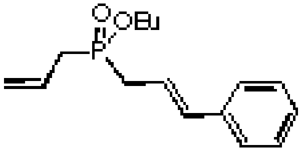

Table 3, entry 3
$^{31}P/^{1}H$ NMR coupled

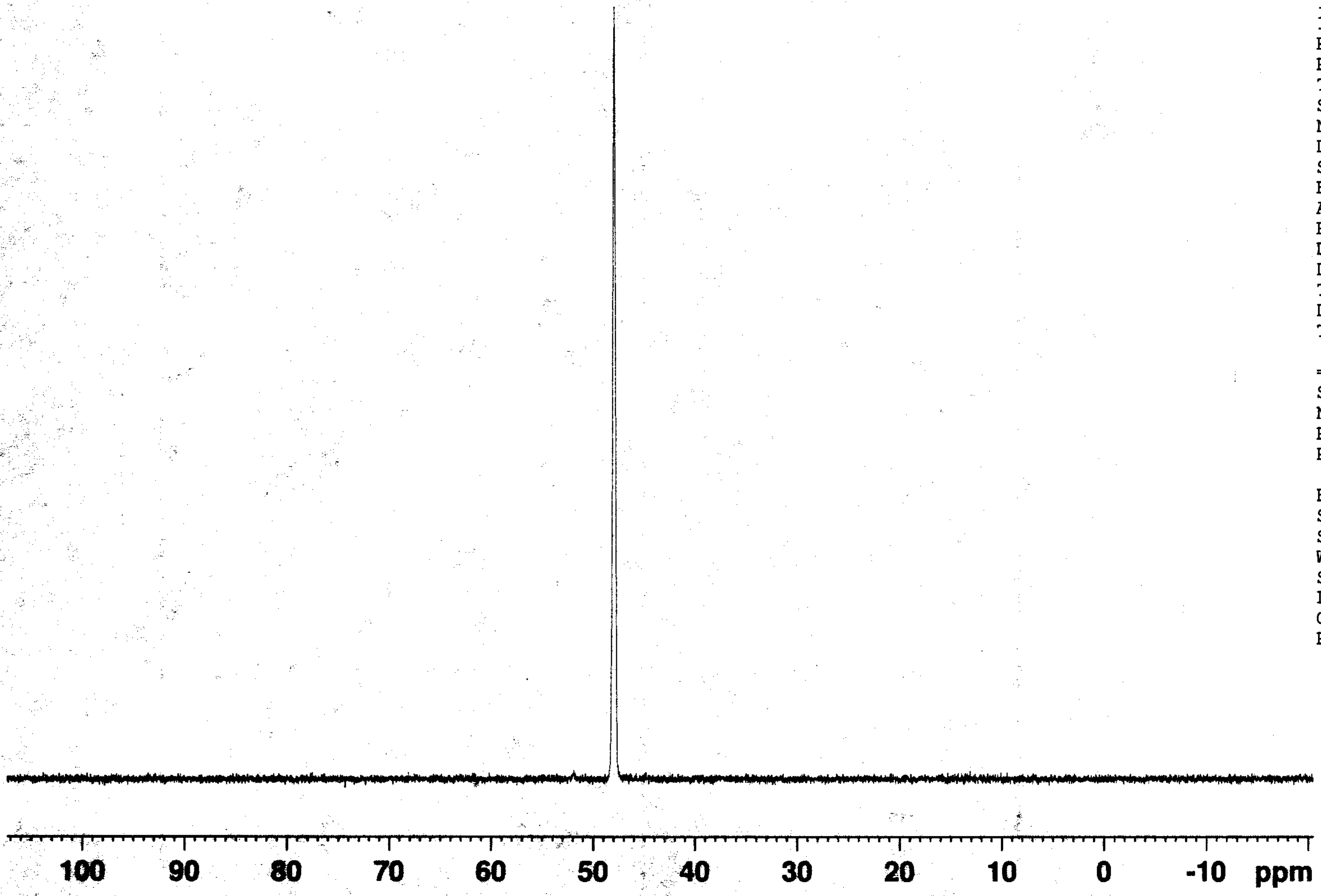


```
Current Data Parameters
NAME                AFL072
EXPNO                    4
PROCNO                   1

F2 - Acquisition Parameters
Date_             20150804
Time                 14.21
INSTRUM              spect
PROBHD   5 mm PABBO BB/
PULPROG               zg30
TD                   65536
SOLVENT            Acetone
NS                       4
DS                       4
SWH              64102.563 Hz
FIDRES            0.978127 Hz
AQ               0.5111808 sec
RG                  203.57
DW                   7.800 usec
DE                    6.50 usec
TE                   295.1 K
D1              2.00000000 sec
TD0                      1

======== CHANNEL f1 ========
SFO1           161.9674942 MHz
NUC1                   31P
P1                   14.25 usec
PLW1           15.00000000 W

F2 - Processing parameters
SI                   32768
SF             161.9755930 MHz
WDW                     EM
SSB      0
LB                    1.00 Hz
GB       0
PC                    1.40
```

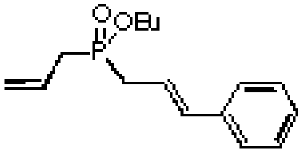

Table 3, entry 3

^1H NMR

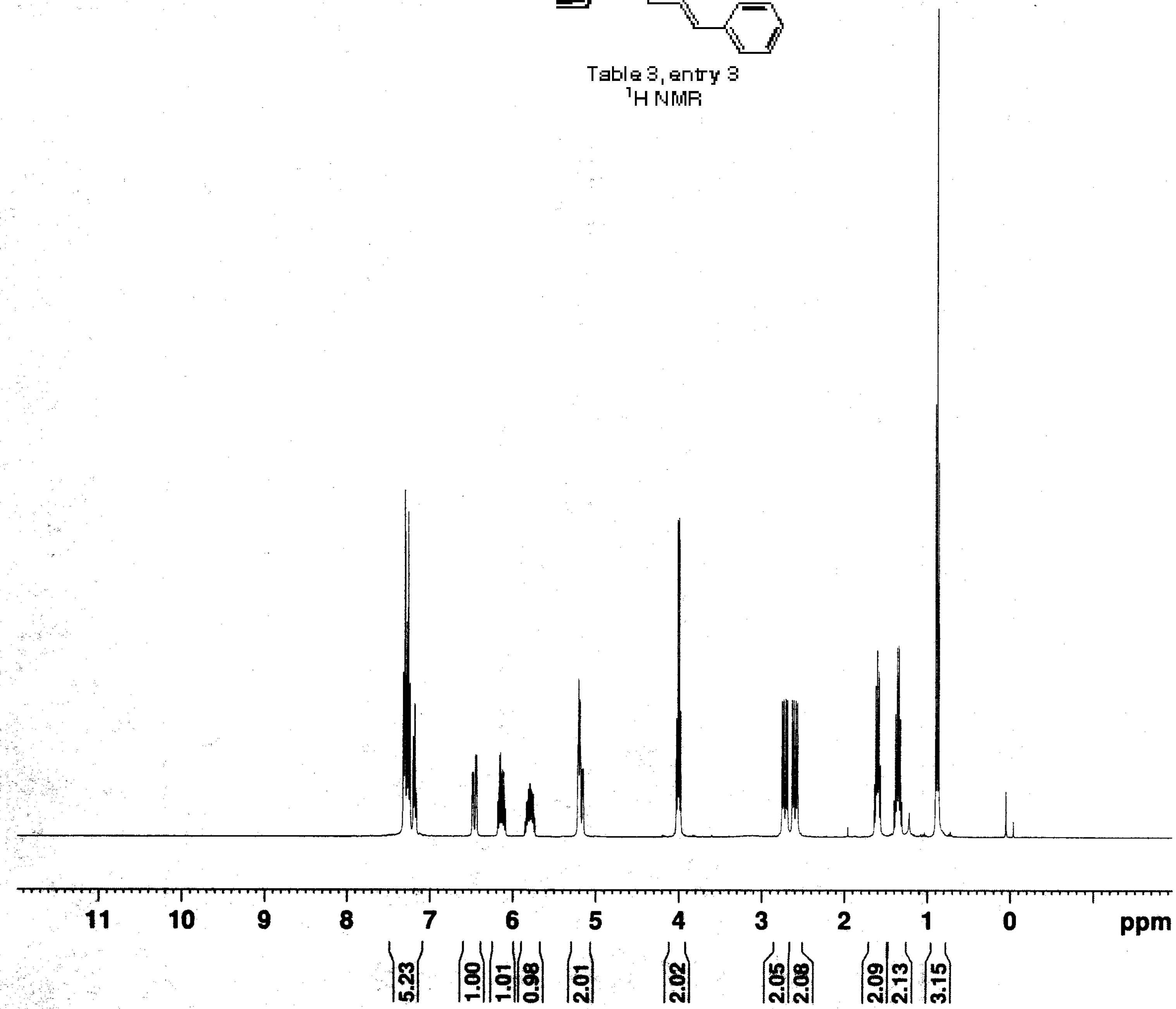

Current Data Parameters
NAME AFL072
EXPNO 5
PROCNO 1

F2 - Acquisition Parameters
Date_ 20150804
Time 14.27
INSTRUM spect
PROBHD 5 mm PABBO BB/
PULPROG zg30
TD 65536
SOLVENT CDCl3
NS 11
DS 2
SWH 8012.820 Hz
FIDRES 0.122266 Hz
AQ 4.0894465 sec
RG 13.94
DW 62.400 usec
DE 6.50 usec
TE 295.1 K
D1 1.00000000 sec
TD0 1

======== CHANNEL f1 ========
SFO1 400.1324710 MHz
NUC1 1H
P1 10.00 usec
PLW1 25.00300026 W

F2 - Processing parameters
SI 65536
SF 400.1300000 MHz
WDW EM
SSB 0
LB 0.30 Hz
GB 0
PC 1.00

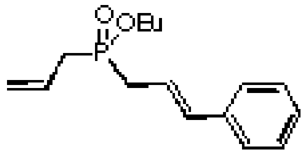

Table 3, entry 3
^{13}C NMR

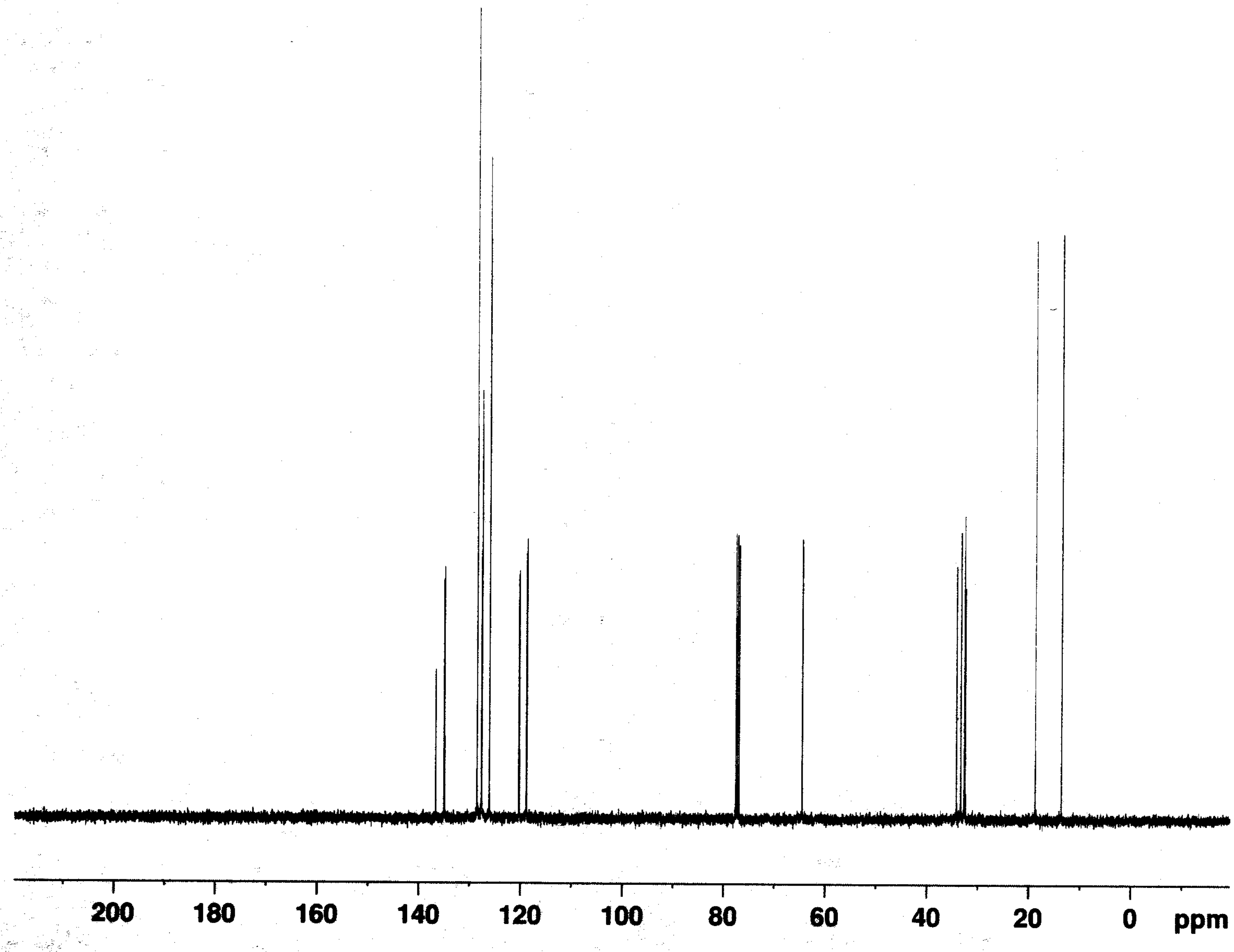

Current Data Parameters
NAME AFL072
EXPNO 6
PROCNO 1

F2 - Acquisition Parameters
Date_ 20150804
Time 14.33
INSTRUM spect
PROBHD 5 mm PABBO BB/
PULPROG zgpg30
TD 65536
SOLVENT CDCl3
NS 29
DS 4
SWH 24038.461 Hz
FIDRES 0.366798 Hz
AQ 1.3631488 sec
RG 203.57
DW 20.800 usec
DE 6.50 usec
TE 295.8 K
D1 2.00000000 sec
D11 0.03000000 sec
TD0 1

======== CHANNEL f1 ========
SFO1 100.6228293 MHz
NUC1 13C
P1 10.00 usec
PLW1 45.00000000 W

======== CHANNEL f2 ========
SFO2 400.1316005 MHz
NUC2 1H
CPDPRG[2 waltz16
PCPD2 90.00 usec
PLW2 10.00000000 W
PLW12 0.31604999 W
PLW13 0.25600001 W

F2 - Processing parameters
SI 32768
SF 100.6127685 MHz
WDW EM
SSB 0
LB 1.00 Hz
GB 0
PC 1.40

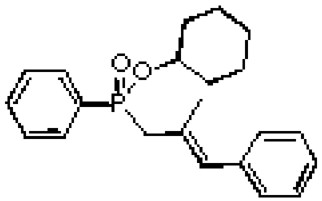

Table 3, entry 4

$^{31}P/^{1}H$ NMR decoupled

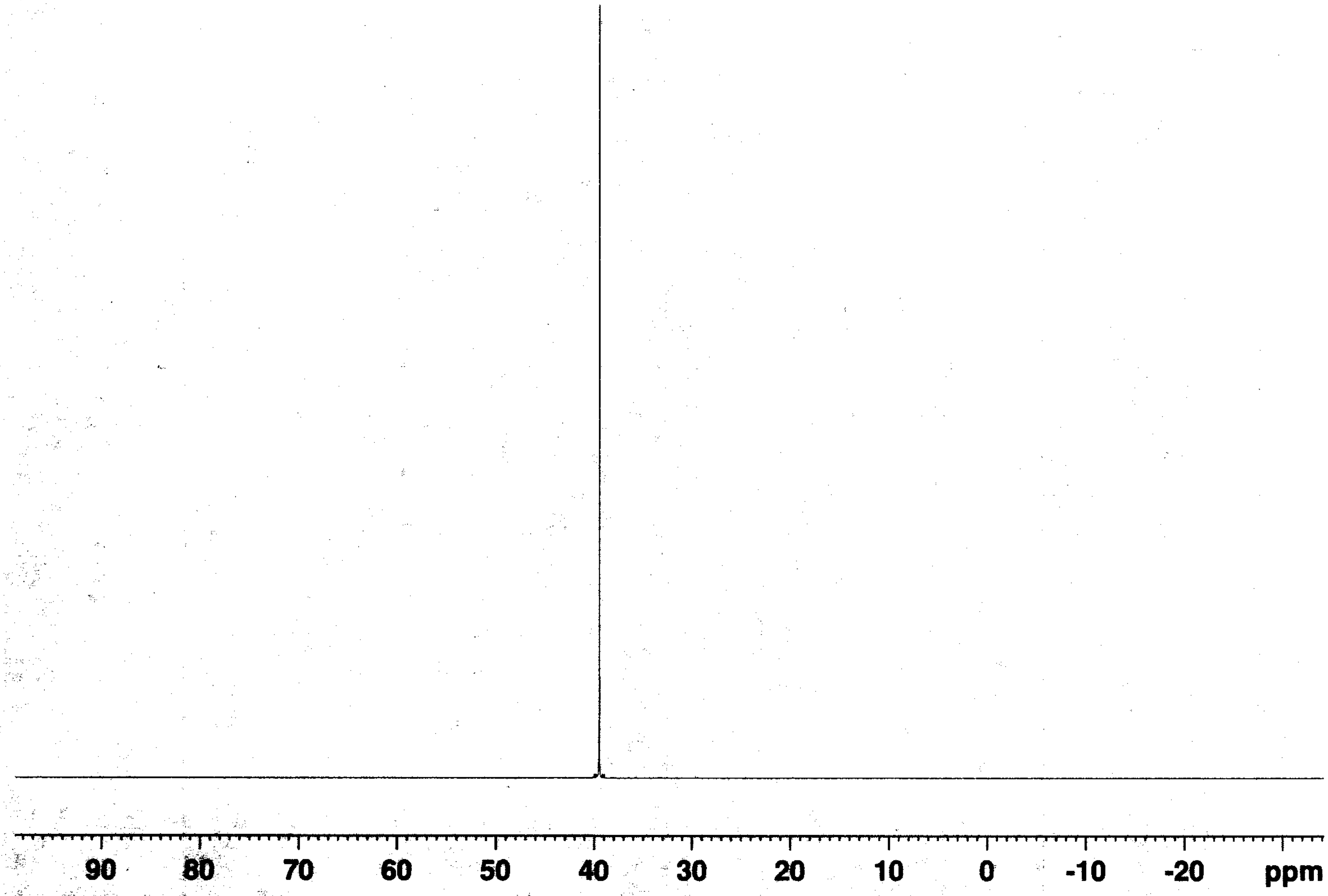

Current Data Parameters

NAME	AFL066	
EXPNO	3	
PROCNO	1	

F2 - Acquisition Parameters

Date_	20150730	
Time	11.35	
INSTRUM	spect	
PROBHD	5 mm PABBO BB/	
PULPROG	zgpg30	
TD	65536	
SOLVENT	CDCl3	
NS	16	
DS	4	
SWH	64102.563	Hz
FIDRES	0.978127	Hz
AQ	0.5111808	sec
RG	203.57	
DW	7.800	usec
DE	6.50	usec
TE	295.1	K
D1	2.00000000	sec
D11	0.03000000	sec
TD0	1	

======== CHANNEL f1 ========

SFO1	161.9674942	MHz
NUC1	31P	
P1	14.25	usec
PLW1	15.00000000	W

======== CHANNEL f2 ========

SFO2	400.1316005	MHz
NUC2	1H	
CPDPRG[2	waltz16	
PCPD2	90.00	usec
PLW2	10.00000000	W
PLW12	0.31604999	W
PLW13	0.25600001	W

F2 - Processing parameters

SI	32768	
SF	161.9755930	MHz
WDW	EM	
SSB	0	
LB	1.00	Hz
GB	0	
PC	1.40	

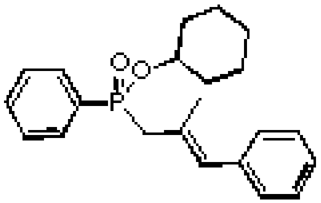

Table 3, entry 4
$^{31}P/^{1}H$ NMR coupled

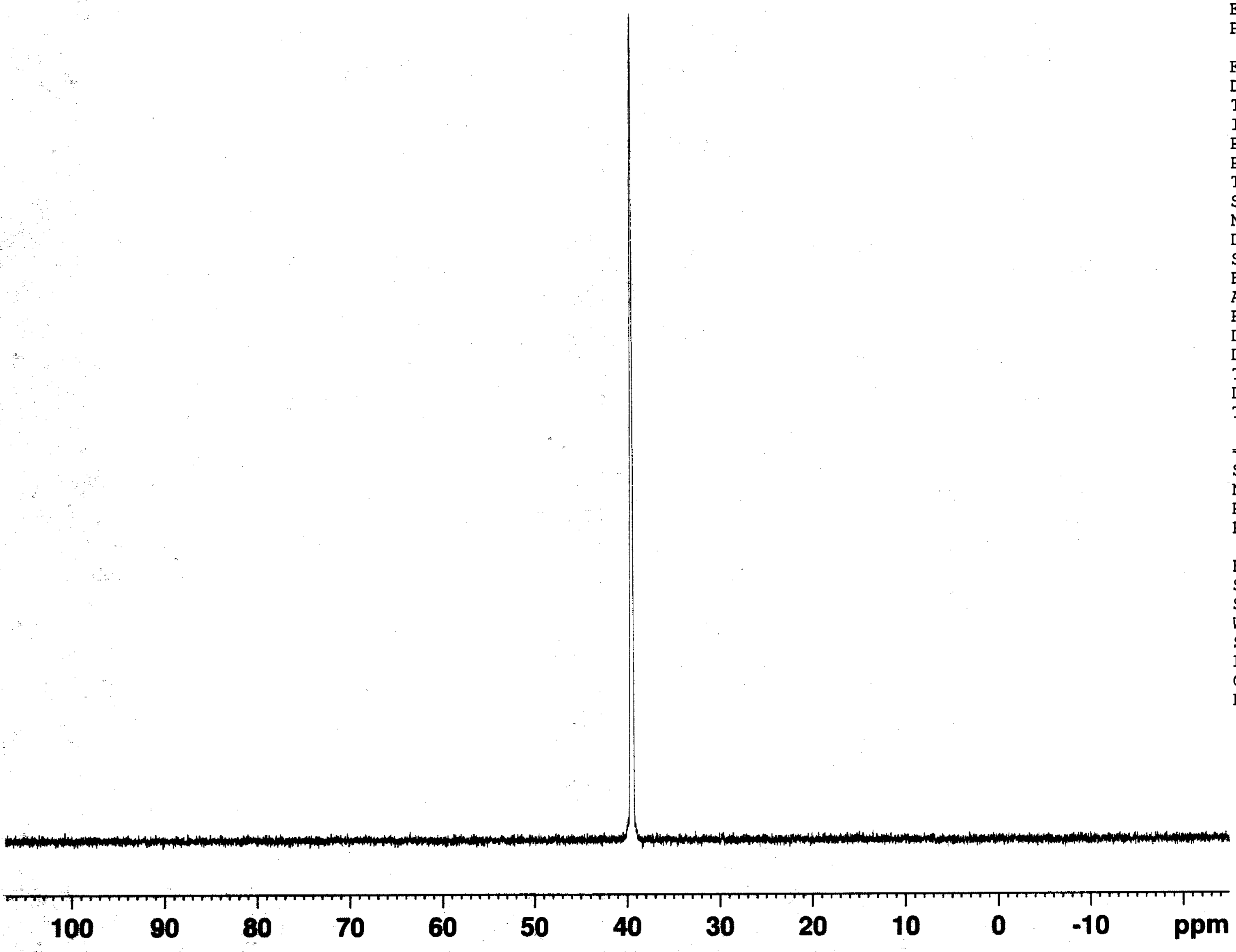

Current Data Parameters

NAME	AFL066
EXPNO	4
PROCNO	1

F2 - Acquisition Parameters

Date_	20150730
Time	11.37
INSTRUM	spect
PROBHD	5 mm PABBO BB/
PULPROG	zg30
TD	65536
SOLVENT	CDCl3
NS	18
DS	4
SWH	64102.563 Hz
FIDRES	0.978127 Hz
AQ	0.5111808 sec
RG	203.57
DW	7.800 usec
DE	6.50 usec
TE	294.7 K
D1	2.00000000 sec
TD0	1

======== CHANNEL f1 ========

SFO1	161.9674942 MHz
NUC1	31P
P1	14.25 usec
PLW1	15.00000000 W

F2 - Processing parameters

SI	32768
SF	161.9755930 MHz
WDW	EM
SSB	0
LB	1.00 Hz
GB	0
PC	1.40

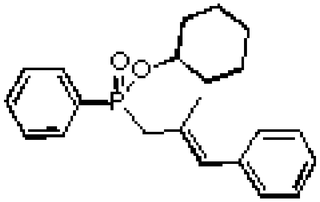

Table 3, entry 4

^{1}H NMR

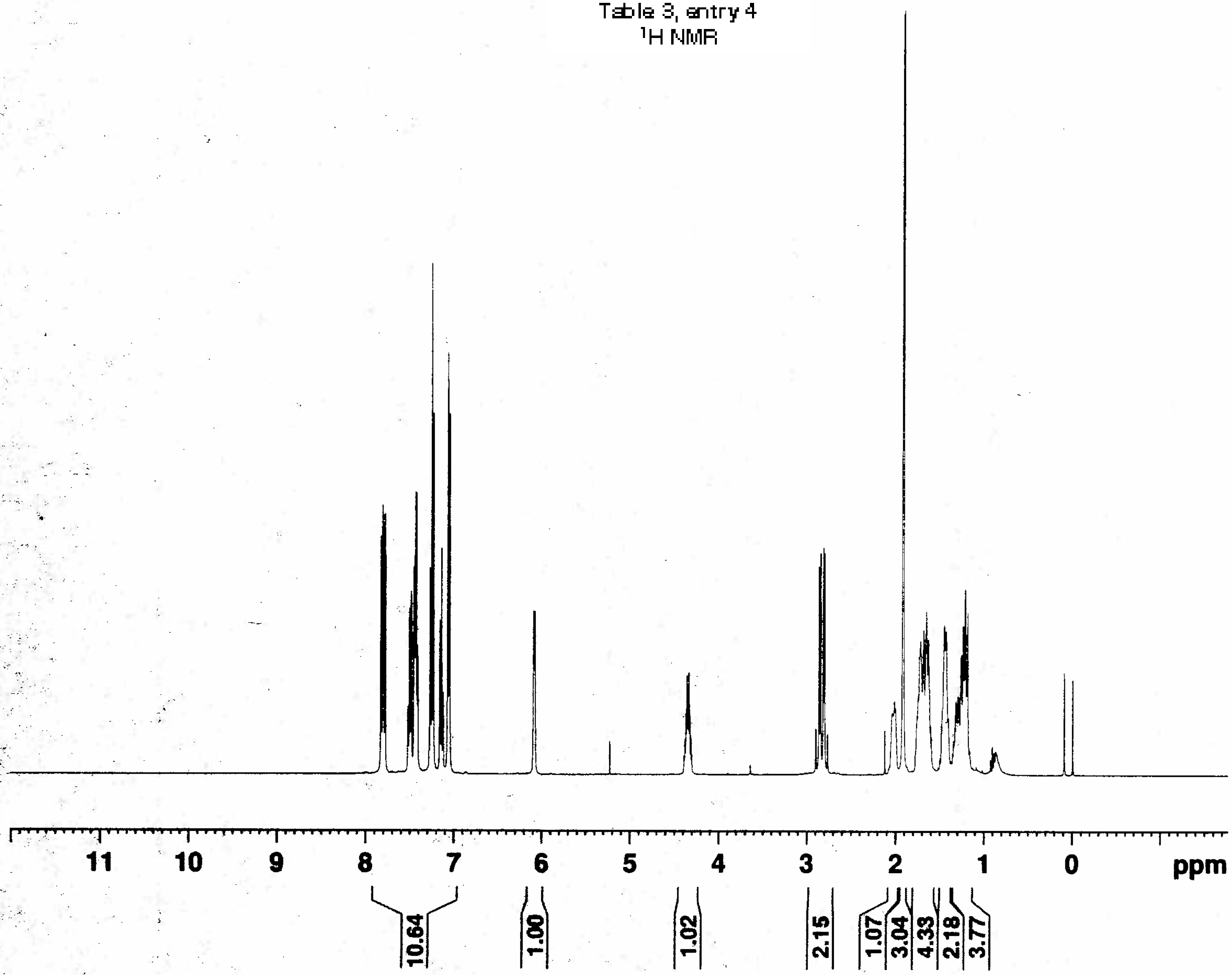

BRUKER

Current Data Parameters

NAME	AFL066
EXPNO	10
PROCNO	1

F2 - Acquisition Parameters

Date_	20150827
Time	11.32
INSTRUM	spect
PROBHD	5 mm PABBO BB/
PULPROG	zg30
TD	65536
SOLVENT	CDCl3
NS	16
DS	2
SWH	8012.820 Hz
FIDRES	0.122266 Hz
AQ	4.0894465 sec
RG	13.94
DW	62.400 usec
DE	6.50 usec
TE	294.5 K
D1	1.00000000 sec
TD0	1

======== CHANNEL f1 ========

SFO1	400.1324710 MHz
NUC1	1H
P1	10.00 usec
PLW1	25.00300026 W

F2 - Processing parameters

SI	65536
SF	400.1300000 MHz
WDW	EM
SSB	0
LB	0.30 Hz
GB	0
PC	1.00

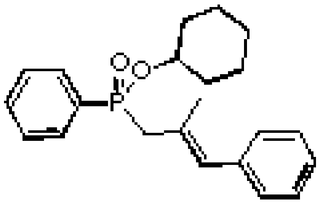

Table 3, entry 4
^{13}C NMR

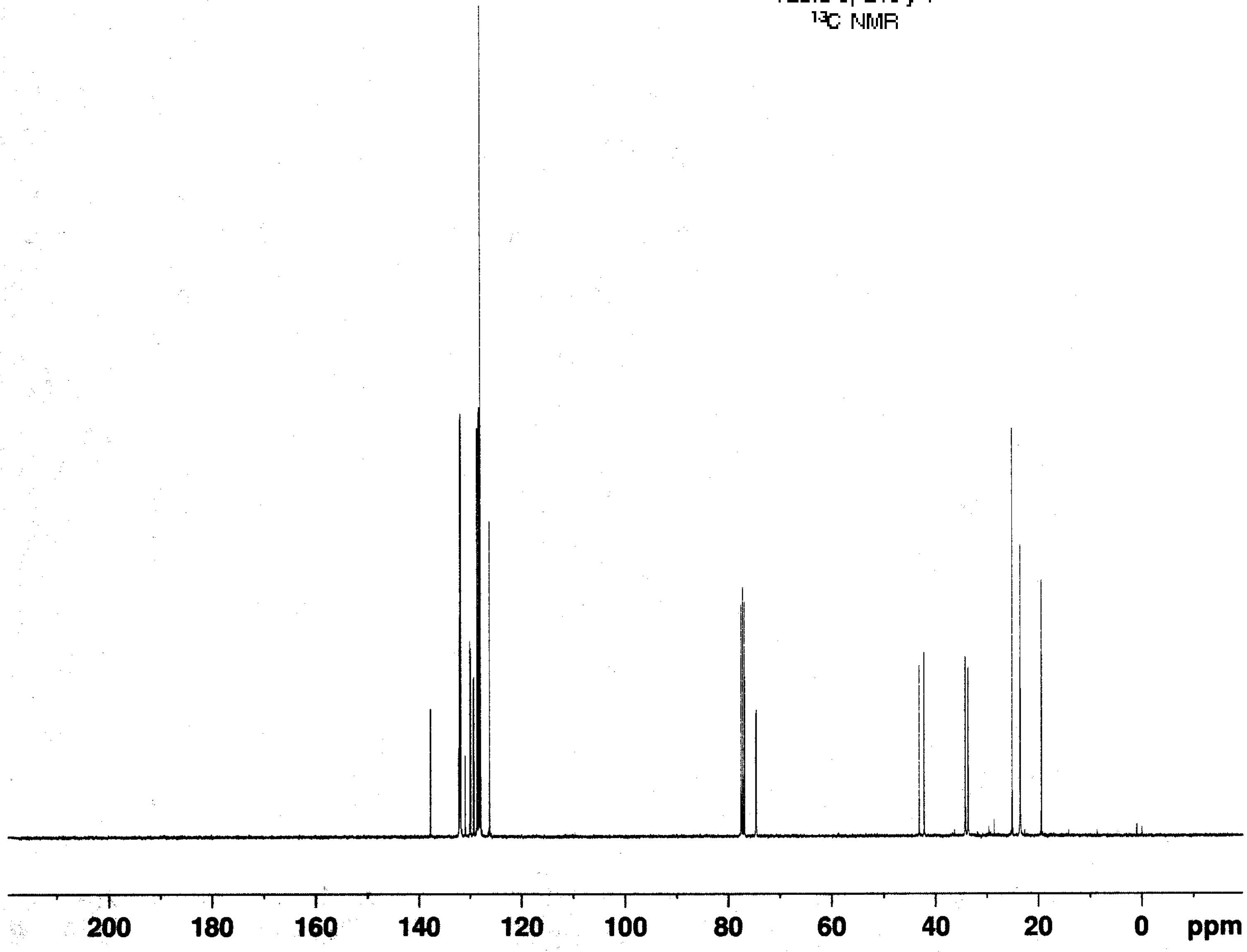

Current Data Parameters
NAME AFL066
EXPNO 11
PROCNO 1

F2 - Acquisition Parameters
Date_ 20150827
Time 11.55
INSTRUM spect
PROBHD 5 mm PABBO BB/
PULPROG zgpg30
TD 65536
SOLVENT CDCl3
NS 293
DS 4
SWH 24038.461 Hz
FIDRES 0.366798 Hz
AQ 1.3631488 sec
RG 203.57
DW 20.800 usec
DE 6.50 usec
TE 295.2 K
D1 2.00000000 sec
D11 0.03000000 sec
TD0 1

======== CHANNEL f1 ========
SFO1 100.6228293 MHz
NUC1 13C
P1 10.00 usec
PLW1 45.00000000 W

======== CHANNEL f2 ========
SFO2 400.1316005 MHz
NUC2 1H
CPDPRG[2 waltz16
PCPD2 90.00 usec
PLW2 10.00000000 W
PLW12 0.31604999 W
PLW13 0.25600001 W

F2 - Processing parameters
SI 32768
SF 100.6127685 MHz
WDW EM
SSB 0
LB 1.00 Hz
GB 0
PC 1.40

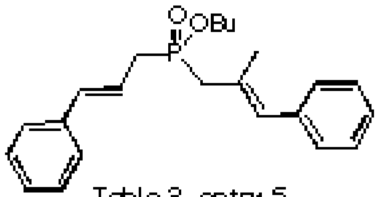

Table 3, entry 5

$^{31}P/^{1}H$ NMR decoupled

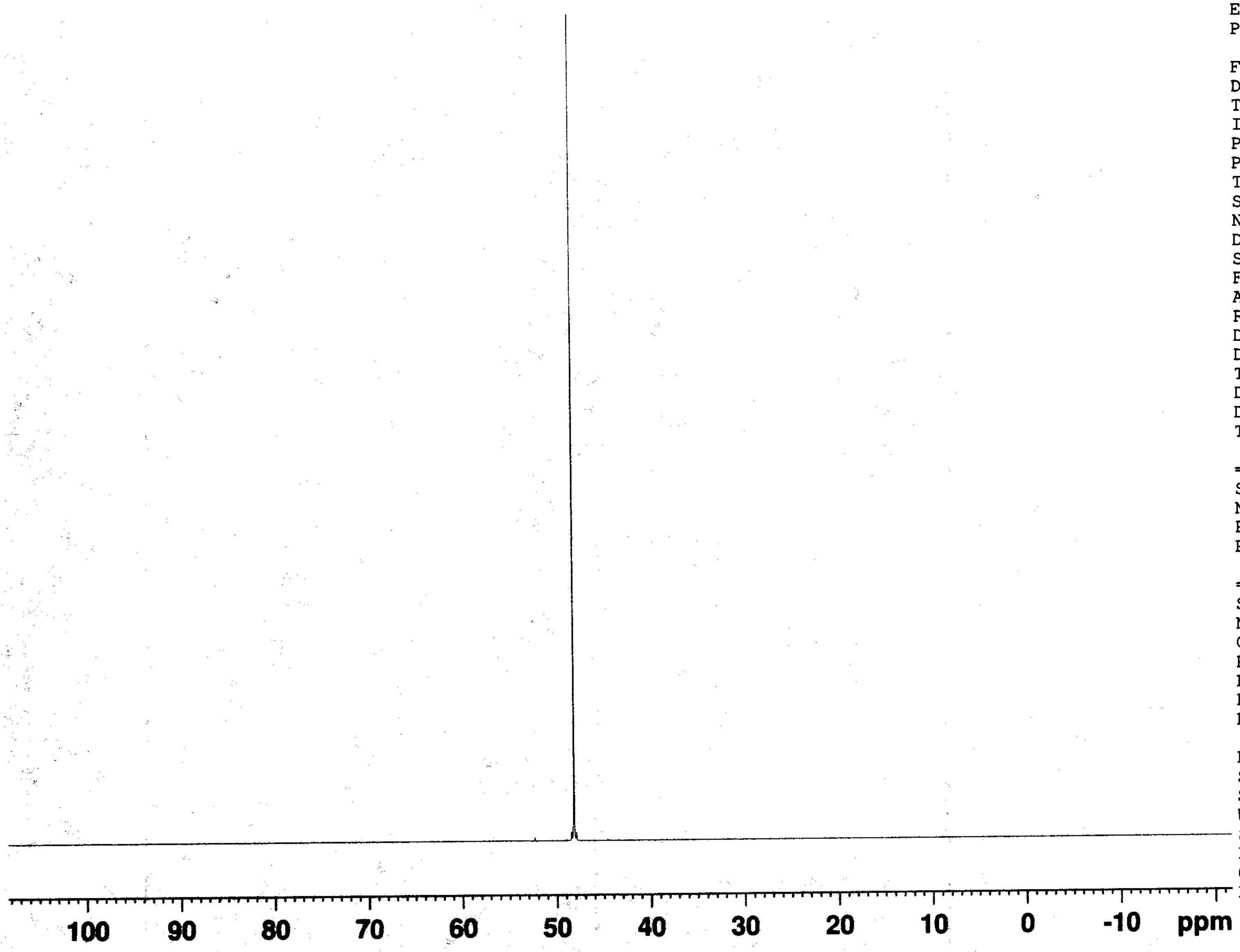

Current Data Parameters

NAME	AFL092	
EXPNO	3	
PROCNO	1	

F2 - Acquisition Parameters

Date_	20150813	
Time	11.37	
INSTRUM	spect	
PROBHD	5 mm PABBO BB/	
PULPROG	zgpg30	
TD	65536	
SOLVENT	Acetone	
NS	9	
DS	4	
SWH	64102.563	Hz
FIDRES	0.978127	Hz
AQ	0.5111808	sec
RG	203.57	
DW	7.800	usec
DE	6.50	usec
TE	295.8	K
D1	2.00000000	sec
D11	0.03000000	sec
TD0	1	

======== CHANNEL f1 ========

SFO1	161.9674942	MHz
NUC1	31P	
P1	14.25	usec
PLW1	15.00000000	W

======== CHANNEL f2 ========

SFO2	400.1316005	MHz
NUC2	1H	
CPDPRG[2	waltz16	
PCPD2	90.00	usec
PLW2	10.00000000	W
PLW12	0.31604999	W
PLW13	0.25600001	W

F2 - Processing parameters

SI	32768	
SF	161.9755930	MHz
WDW	EM	
SSB	0	
LB	1.00	Hz
GB	0	
PC	1.40	

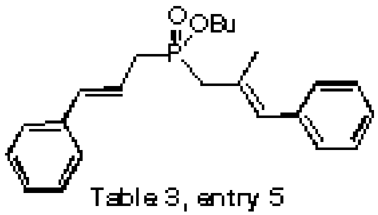

Table 3, entry 5
$^{31}P/^{1}H$ NMR coupled

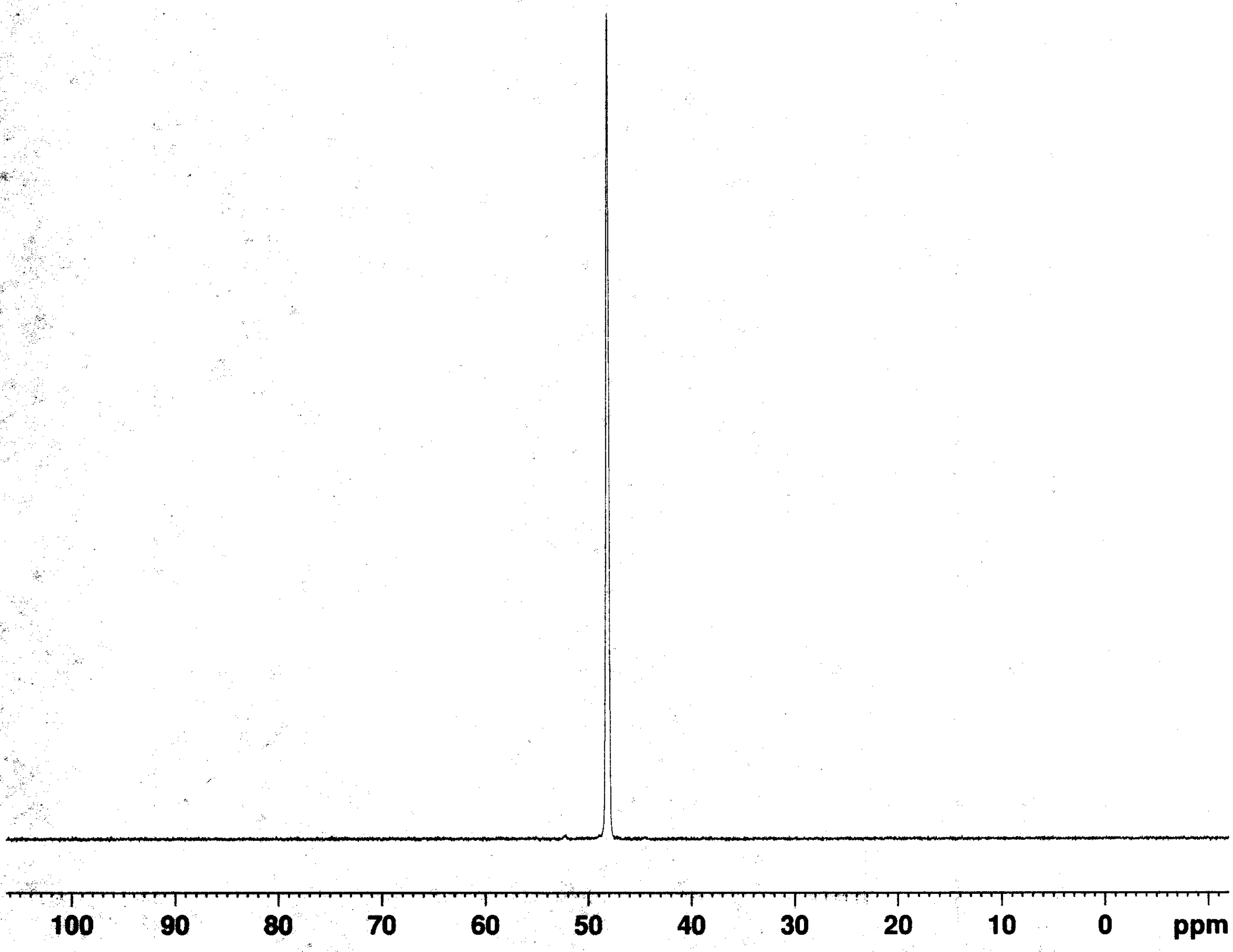


```
Current Data Parameters
NAME               AFL092
EXPNO                   4
PROCNO                  1

F2 - Acquisition Parameters
Date_            20150813
Time                11.39
INSTRUM              spect
PROBHD    5 mm PABBO BB/
PULPROG              zg30
TD                  65536
SOLVENT            Acetone
NS                     29
DS                      4
SWH             64102.563 Hz
FIDRES           0.978127 Hz
AQ              0.5111808 sec
RG                 203.57
DW                  7.800 usec
DE                   6.50 usec
TE                  295.5 K
D1             2.00000000 sec
TD0                     1

======== CHANNEL f1 ========
SFO1          161.9674942 MHz
NUC1                  31P
P1                  14.25 usec
PLW1          15.00000000 W

F2 - Processing parameters
SI                  32768
SF            161.9755930 MHz
WDW                    EM
SSB      0
LB                   1.00 Hz
GB       0
PC                   1.40
```

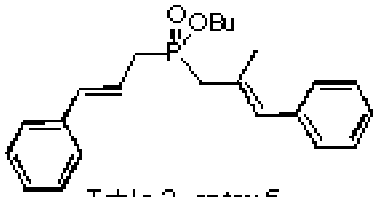

Table 3, entry 5
^{1}H NMR

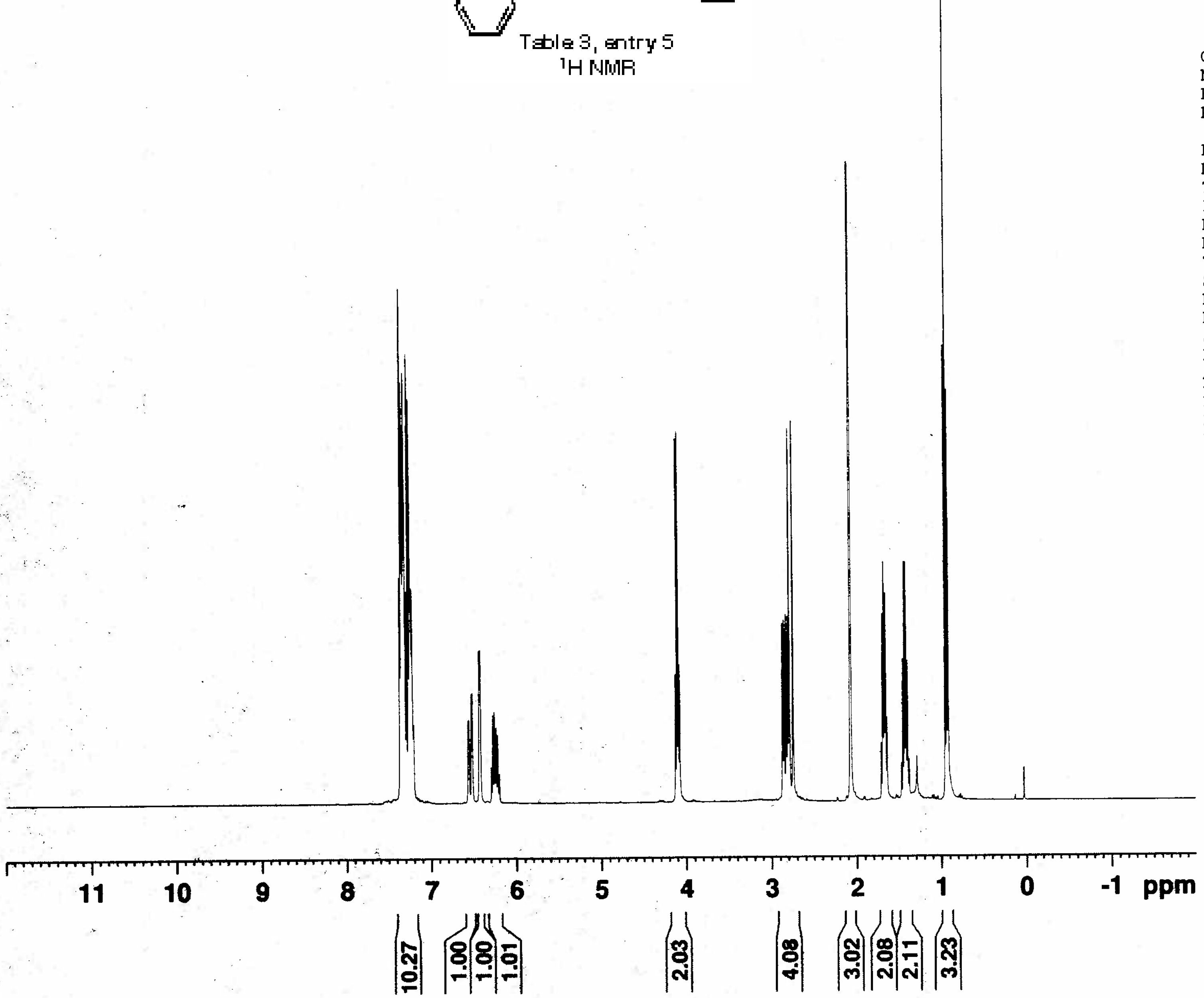


```
Current Data Parameters
NAME                 AFL092
EXPNO                     5
PROCNO                    1

F2 - Acquisition Parameters
Date_              20150813
Time                  11.43
INSTRUM               spect
PROBHD     5 mm PABBO BB/
PULPROG                zg30
TD                    65536
SOLVENT               CDCl3
NS                       16
DS                        2
SWH                8012.820 Hz
FIDRES             0.122266 Hz
AQ                4.0894465 sec
RG                    13.94
DW                   62.400 usec
DE                     6.50 usec
TE                    295.5 K
D1               1.00000000 sec
TD0                       1

======== CHANNEL f1 ========
SFO1            400.1324710 MHz
NUC1                     1H
P1                    10.00 usec
PLW1            25.00300026 W

F2 - Processing parameters
SI                    65536
SF              400.1300000 MHz
WDW                      EM
SSB      0
LB                     0.30 Hz
GB       0
PC                     1.00
```

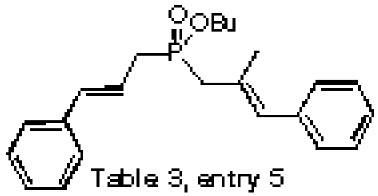

Table 3, entry 5
^{13}C NMR

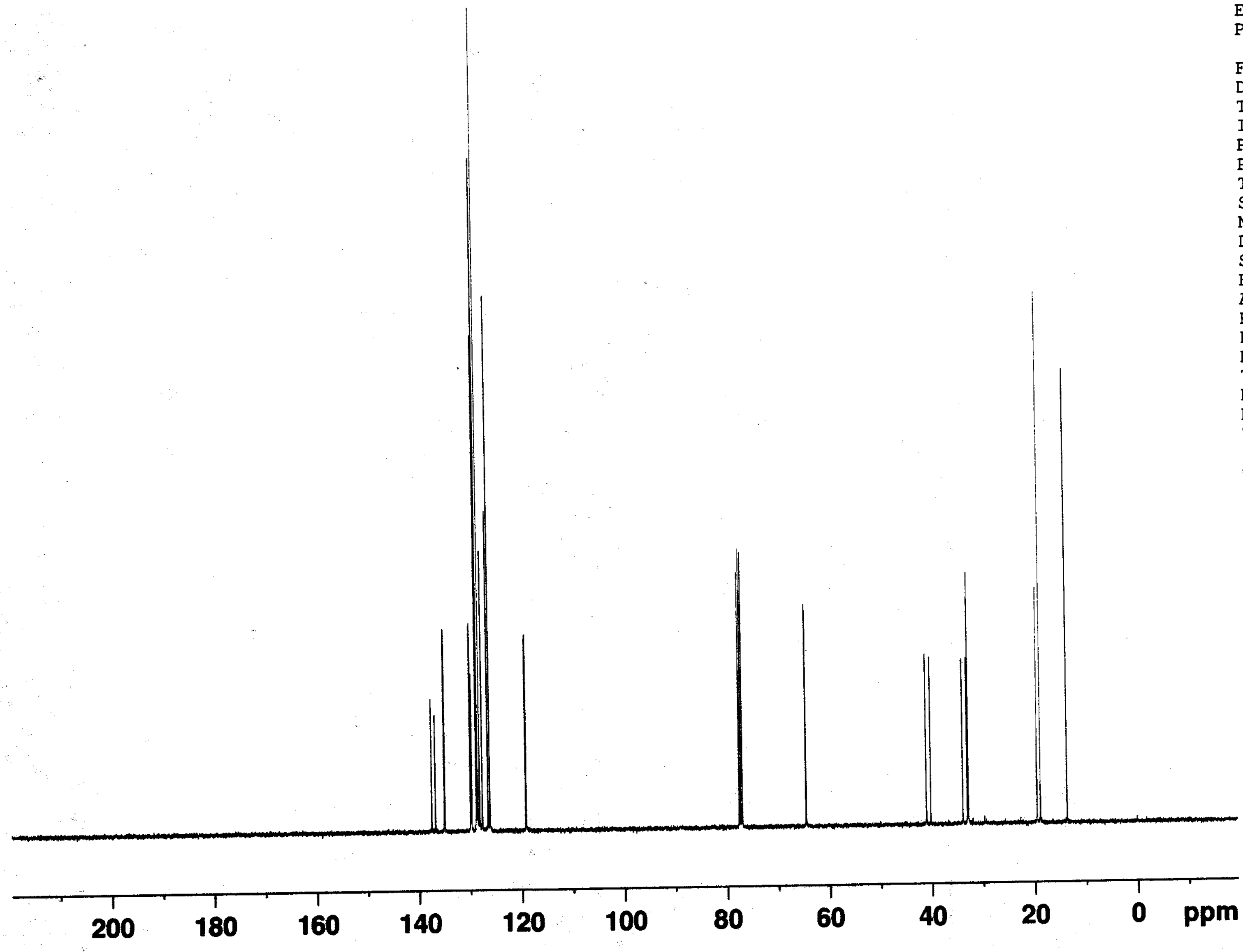

Current Data Parameters
NAME AFL092
EXPNO 6
PROCNO 1

F2 - Acquisition Parameters
Date_ 20150813
Time 11.57
INSTRUM spect
PROBHD 5 mm PABBO BB/
PULPROG zgpg30
TD 65536
SOLVENT CDCl3
NS 156
DS 4
SWH 24038.461 Hz
FIDRES 0.366798 Hz
AQ 1.3631488 sec
RG 203.57
DW 20.800 usec
DE 6.50 usec
TE 296.2 K
D1 2.00000000 sec
D11 0.03000000 sec
TD0 1

======== CHANNEL f1 ========
SFO1 100.6228293 MHz
NUC1 13C
P1 10.00 usec
PLW1 45.00000000 W

======== CHANNEL f2 ========
SFO2 400.1316005 MHz
NUC2 1H
CPDPRG[2 waltz16
PCPD2 90.00 usec
PLW2 10.00000000 W
PLW12 0.31604999 W
PLW13 0.25600001 W

F2 - Processing parameters
SI 32768
SF 100.6127685 MHz
WDW EM
SSB 0
LB 1.00 Hz
GB 0
PC 1.40

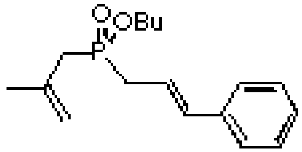

Table 3, entry 6

$^{31}P/^{1}H$ NMR decoupled

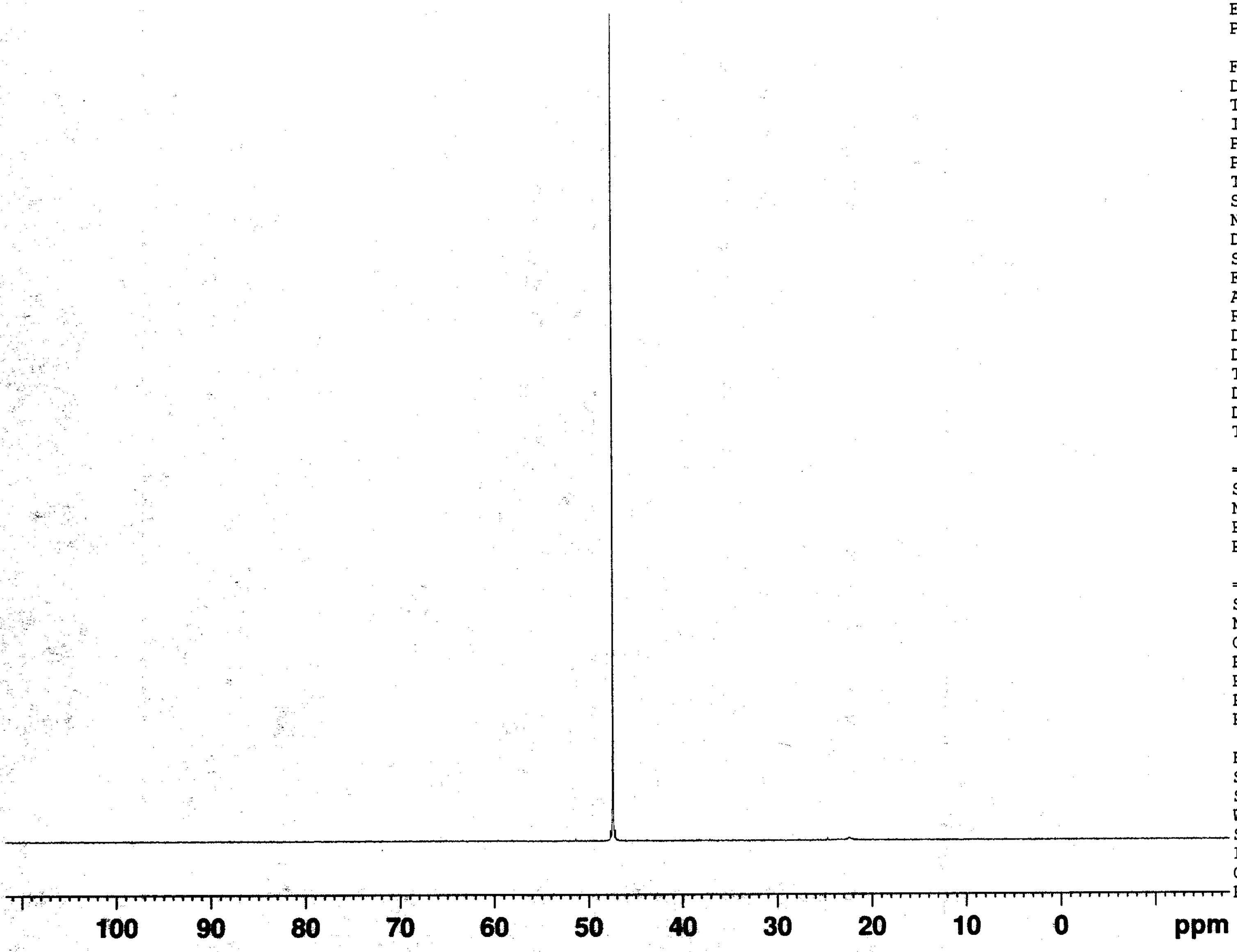

Current Data Parameters

NAME	AFL083
EXPNO	3
PROCNO	1

F2 - Acquisition Parameters

Date_	20150811
Time	9.54
INSTRUM	spect
PROBHD	5 mm PABBO BB/
PULPROG	zgpg30
TD	65536
SOLVENT	D2O
NS	16
DS	4
SWH	64102.563 Hz
FIDRES	0.978127 Hz
AQ	0.5111808 sec
RG	203.57
DW	7.800 usec
DE	6.50 usec
TE	295.2 K
D1	2.00000000 sec
D11	0.03000000 sec
TD0	1

======== CHANNEL f1 ========

SFO1	161.9674942 MHz
NUC1	31P
P1	14.25 usec
PLW1	15.00000000 W

======== CHANNEL f2 ========

SFO2	400.1316005 MHz
NUC2	1H
CPDPRG[2	waltz16
PCPD2	90.00 usec
PLW2	10.00000000 W
PLW12	0.31604999 W
PLW13	0.25600001 W

F2 - Processing parameters

SI	32768
SF	161.9755930 MHz
WDW	EM
SSB	0
LB	1.00 Hz
GB	0
PC	1.40

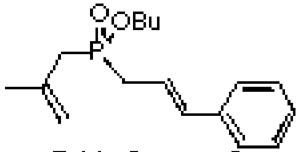

Table 3, entry 6

$^{31}P/^{1}H$ NMR coupled

Current Data Parameters
NAME AFL083
EXPNO 4
PROCNO 1

F2 - Acquisition Parameters
Date_ 20150811
Time 9.56
INSTRUM spect
PROBHD 5 mm PABBO BB/
PULPROG zg30
TD 65536
SOLVENT D2O
NS 18
DS 4
SWH 64102.563 Hz
FIDRES 0.978127 Hz
AQ 0.5111808 sec
RG 203.57
DW 7.800 usec
DE 6.50 usec
TE 294.8 K
D1 2.00000000 sec
TD0 1

======== CHANNEL f1 ========
SFO1 161.9674942 MHz
NUC1 31P
P1 14.25 usec
PLW1 15.00000000 W

F2 - Processing parameters
SI 32768
SF 161.9755930 MHz
WDW EM
SSB 0
LB 1.00 Hz
GB 0
PC 1.40

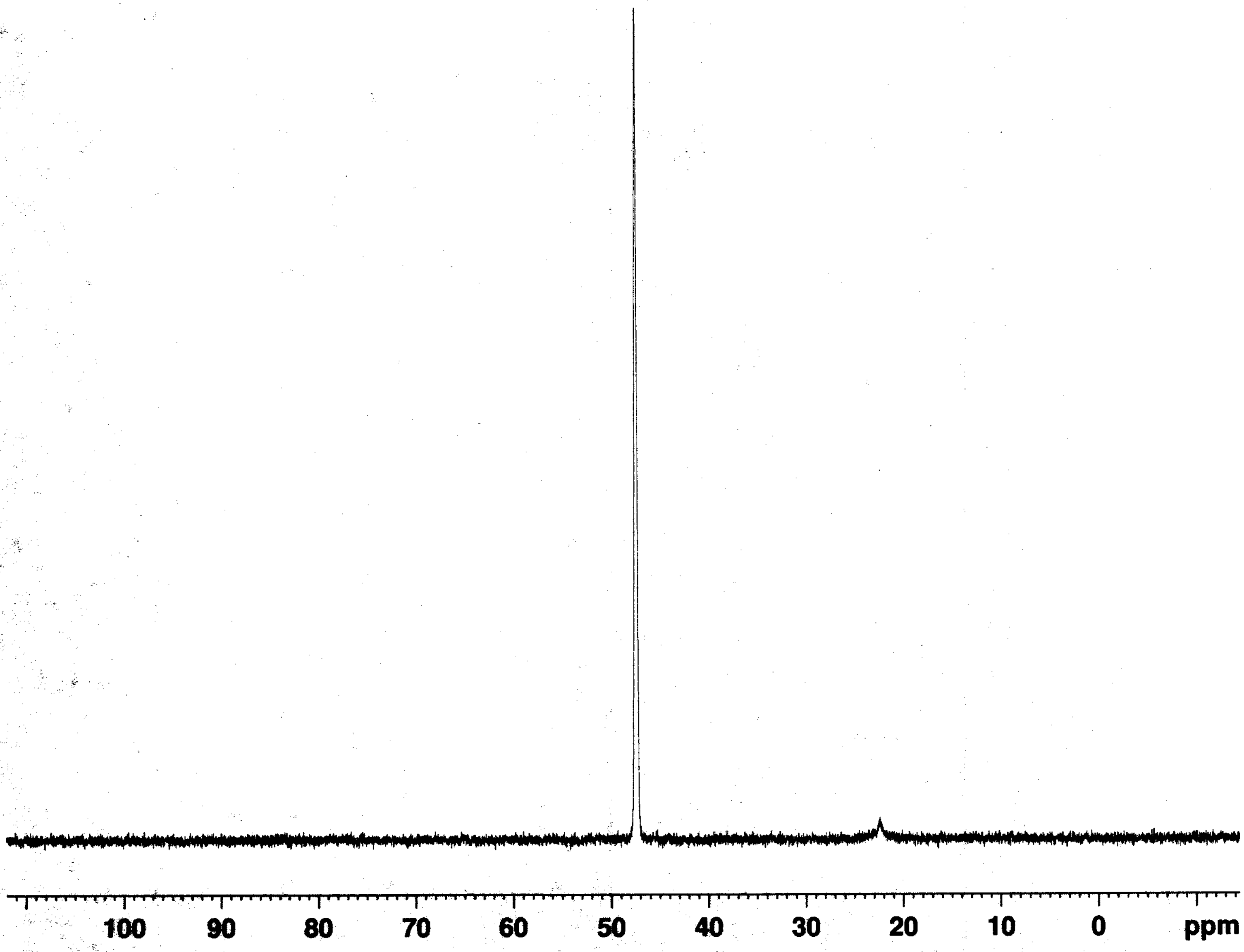

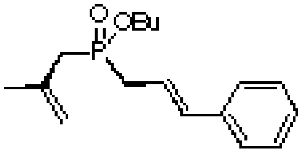

Table 3, entry 6
^{1}H NMR

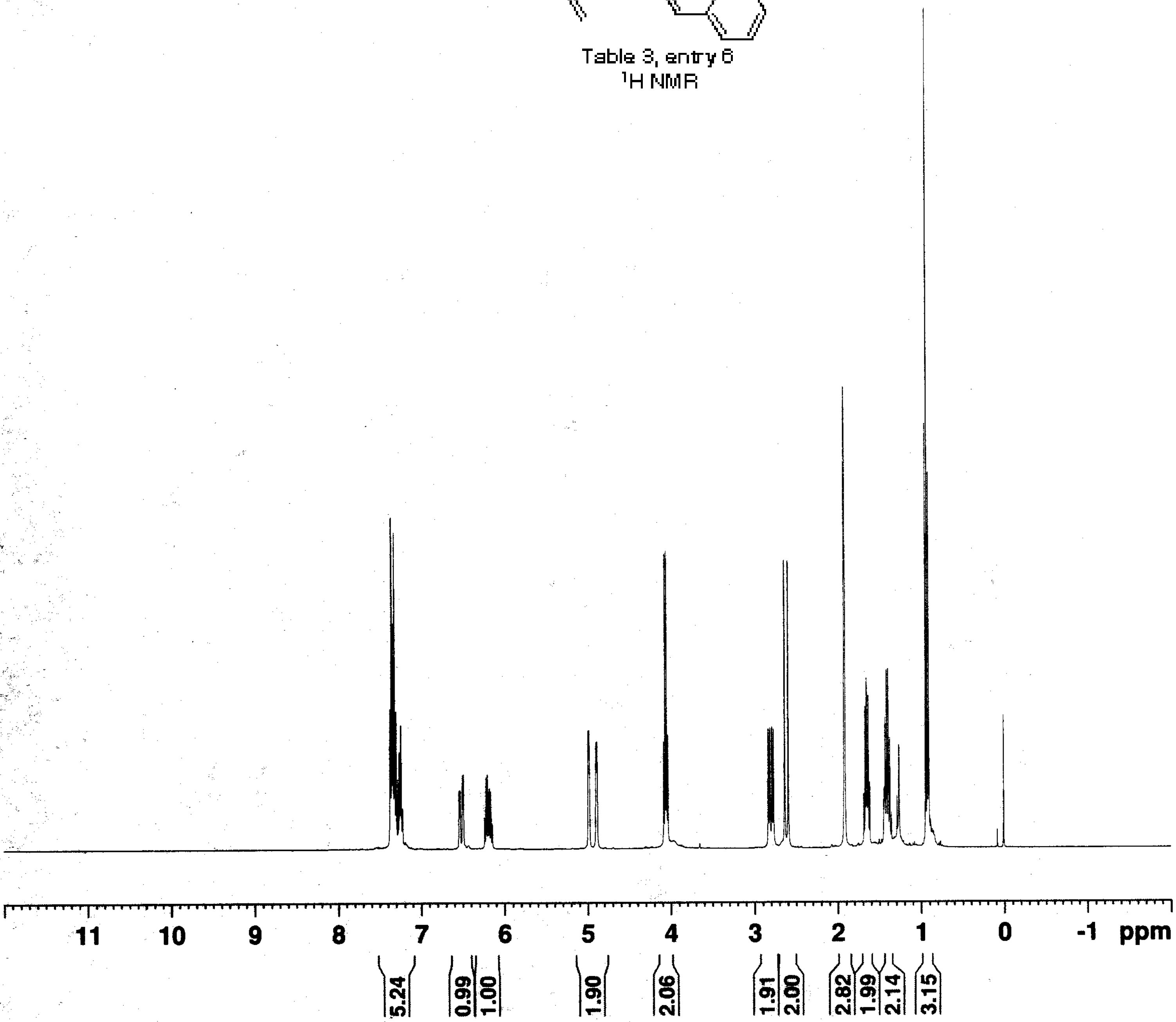

Current Data Parameters

NAME	AFL083
EXPNO	5
PROCNO	1

F2 - Acquisition Parameters

Date_	20150811
Time	10.03
INSTRUM	spect
PROBHD	5 mm PABBO BB/
PULPROG	zg30
TD	65536
SOLVENT	CDCl3
NS	16
DS	2
SWH	8012.820 Hz
FIDRES	0.122266 Hz
AQ	4.0894465 sec
RG	32.38
DW	62.400 usec
DE	6.50 usec
TE	294.9 K
D1	1.00000000 sec
TD0	1

======== CHANNEL f1 ========

SFO1	400.1324710 MHz
NUC1	1H
P1	10.00 usec
PLW1	25.00300026 W

F2 - Processing parameters

SI	65536
SF	400.1300000 MHz
WDW	EM
SSB	0
LB	0.30 Hz
GB	0
PC	1.00

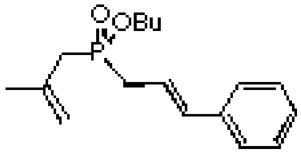

Table 3, entry 6
^{13}C NMR

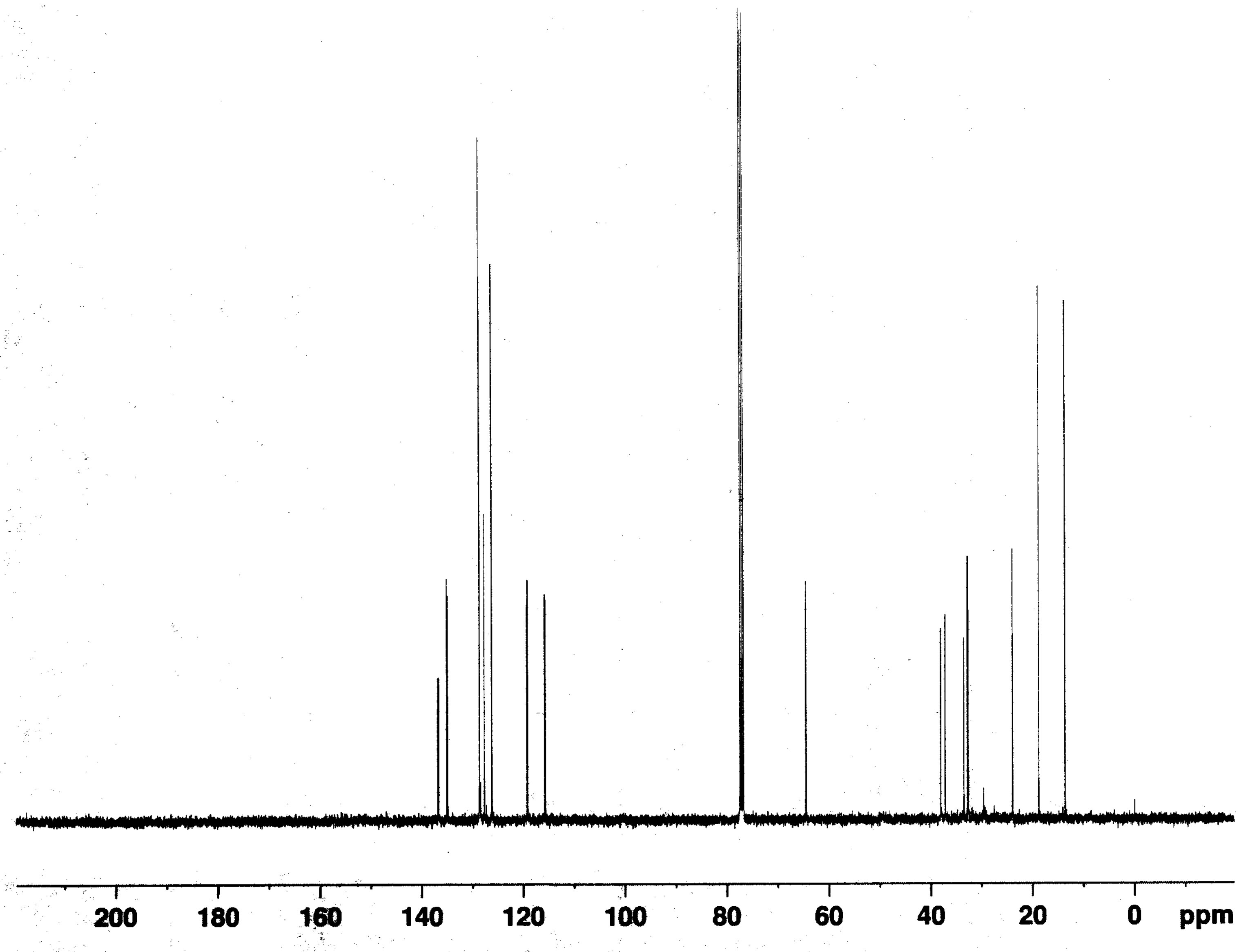


```
Current Data Parameters
NAME              AFL083
EXPNO                  6
PROCNO                 1

F2 - Acquisition Parameters
Date_           20150811
Time               10.27
INSTRUM            spect
PROBHD   5 mm PABBO BB/
PULPROG          zgpg30
TD                65536
SOLVENT           CDCl3
NS                  210
DS                    4
SWH           24038.461 Hz
FIDRES         0.366798 Hz
AQ            1.3631488 sec
RG               203.57
DW               20.800 usec
DE                 6.50 usec
TE                295.9 K
D1           2.00000000 sec
D11          0.03000000 sec
TD0                   1

======== CHANNEL f1 ========
SFO1        100.6228293 MHz
NUC1                13C
P1                10.00 usec
PLW1        45.00000000 W

======== CHANNEL f2 ========
SFO2        400.1316005 MHz
NUC2                 1H
CPDPRG[2        waltz16
PCPD2             90.00 usec
PLW2        10.00000000 W
PLW12        0.31604999 W
PLW13        0.25600001 W

F2 - Processing parameters
SI                32768
SF          100.6127685 MHz
WDW                  EM
SSB      0
LB                 1.00 Hz
GB       0
PC                 1.40
```

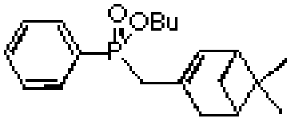

Table 3, entry 7

$^{31}P/^{1}H$ NMR decoupled

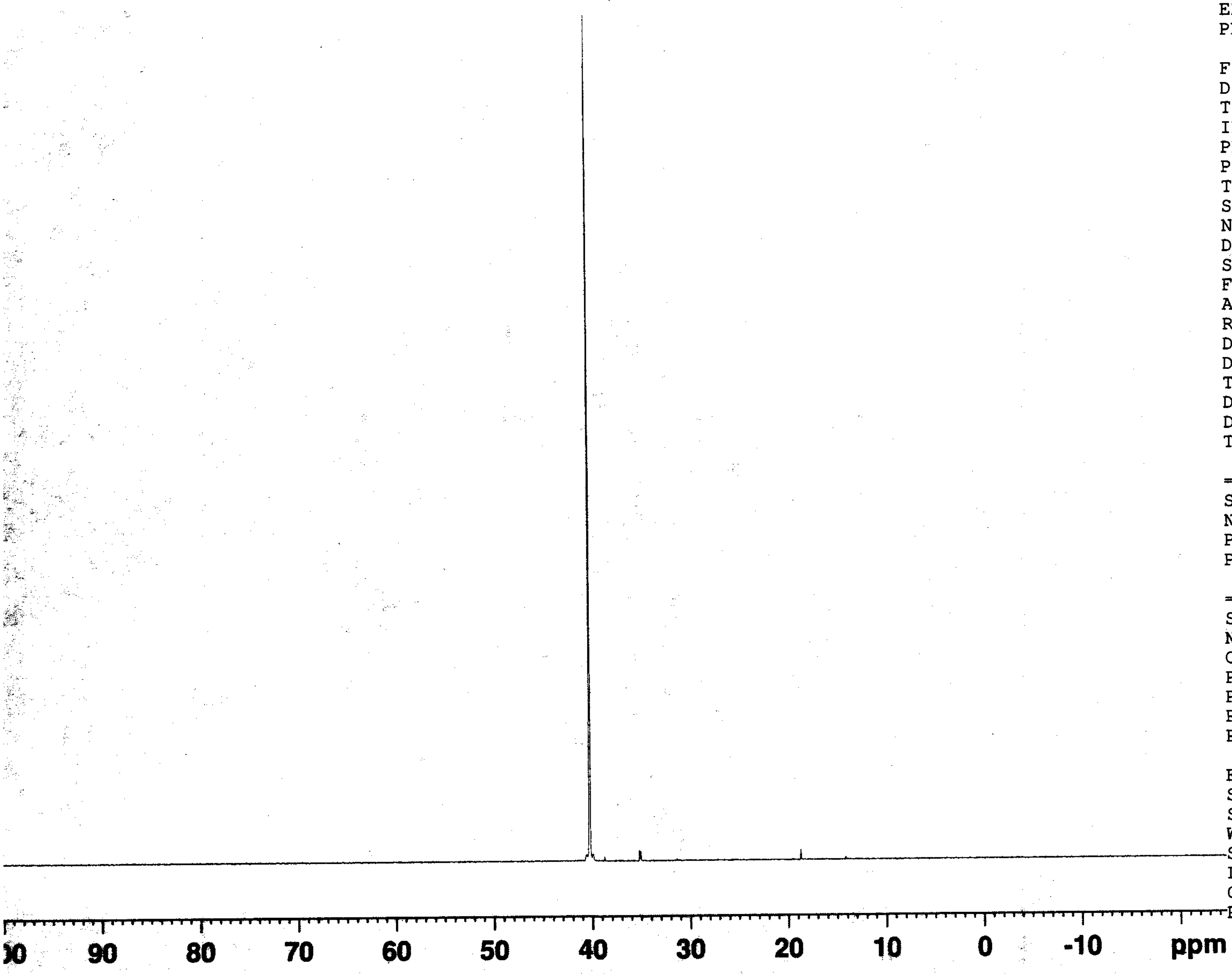

Current Data Parameters
NAME AFL071
EXPNO 3
PROCNO 1

F2 - Acquisition Parameters
Date_ 20150804
Time 14.42
INSTRUM spect
PROBHD 5 mm PABBO BB/
PULPROG zgpg30
TD 65536
SOLVENT CDCl3
NS 16
DS 4
SWH 64102.563 Hz
FIDRES 0.978127 Hz
AQ 0.5111808 sec
RG 203.57
DW 7.800 usec
DE 6.50 usec
TE 295.7 K
D1 2.00000000 sec
D11 0.03000000 sec
TD0 1

======== CHANNEL f1 ========
SFO1 161.9674942 MHz
NUC1 31P
P1 14.25 usec
PLW1 15.00000000 W

======== CHANNEL f2 ========
SFO2 400.1316005 MHz
NUC2 1H
CPDPRG[2 waltz16
PCPD2 90.00 usec
PLW2 10.00000000 W
PLW12 0.31604999 W
PLW13 0.25600001 W

F2 - Processing parameters
SI 32768
SF 161.9755930 MHz
WDW EM
SSB 0
LB 1.00 Hz
GB 0
PC 1.40

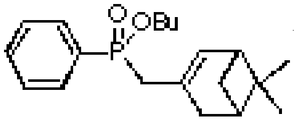

Table 3, entry 7
$^{31}P/^{1}H$ NMR coupled

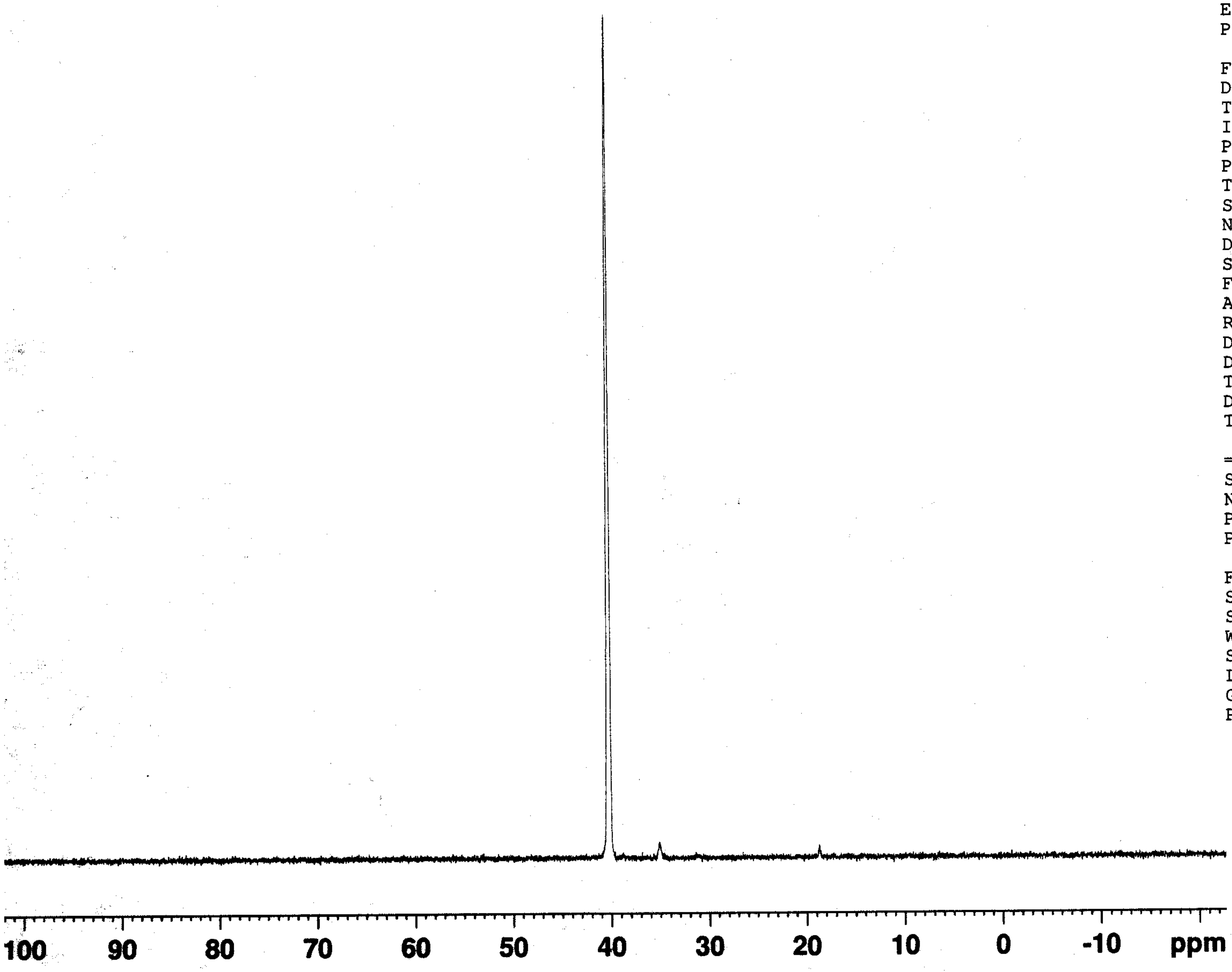

Current Data Parameters
NAME AFL071
EXPNO 4
PROCNO 1

F2 - Acquisition Parameters
Date_ 20150804
Time 14.44
INSTRUM spect
PROBHD 5 mm PABBO BB/
PULPROG zg30
TD 65536
SOLVENT CDCl3
NS 12
DS 4
SWH 64102.563 Hz
FIDRES 0.978127 Hz
AQ 0.5111808 sec
RG 203.57
DW 7.800 usec
DE 6.50 usec
TE 295.3 K
D1 2.00000000 sec
TD0 1

======== CHANNEL f1 ========
SFO1 161.9674942 MHz
NUC1 31P
P1 14.25 usec
PLW1 15.00000000 W

F2 - Processing parameters
SI 32768
SF 161.9755930 MHz
WDW EM
SSB 0
LB 1.00 Hz
GB 0
PC 1.40

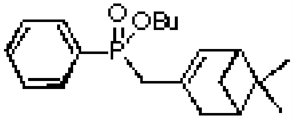

Table 3, entry 7
^{1}H NMR

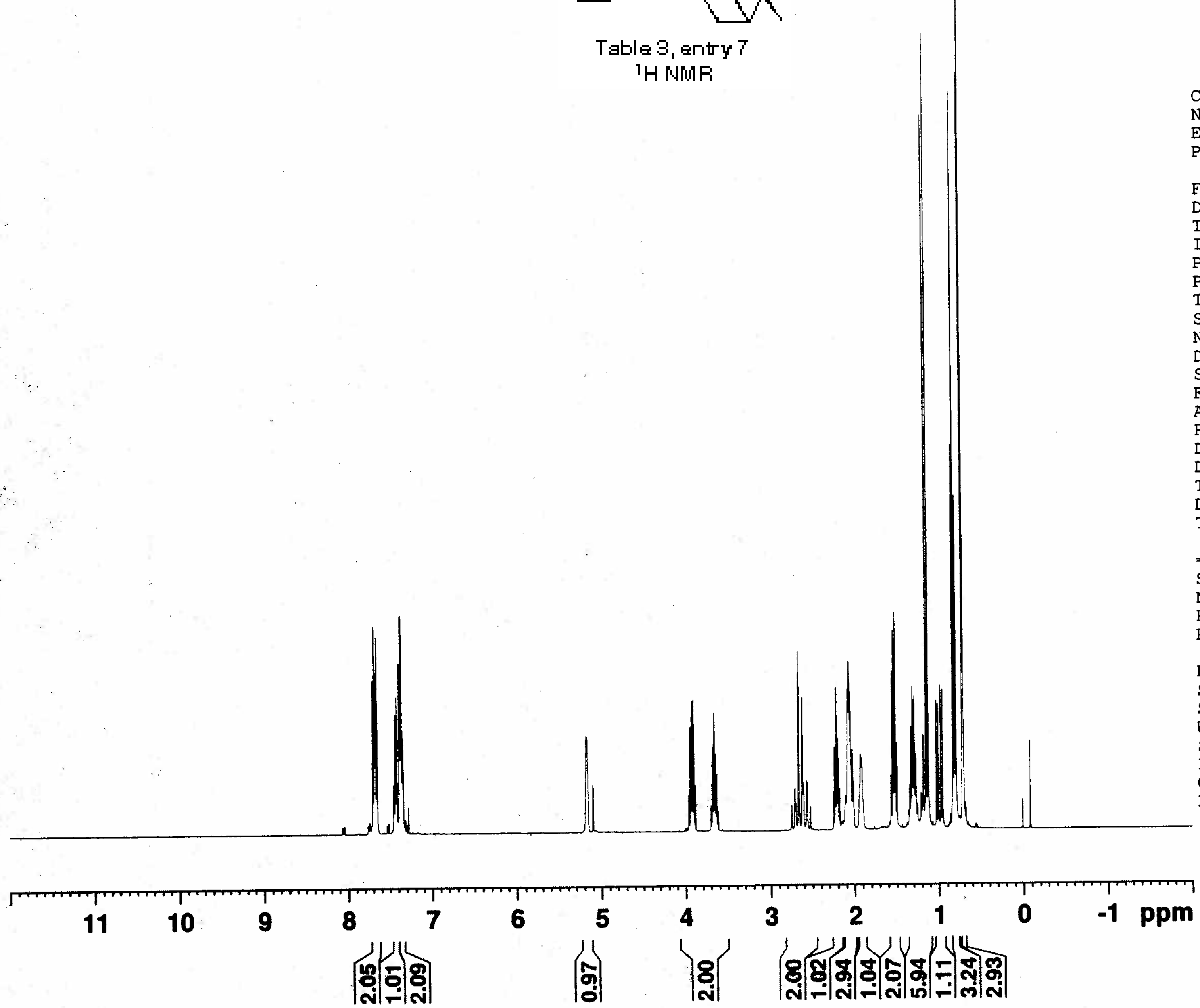

BRUKER

Current Data Parameters
NAME AFL071
EXPNO 10
PROCNO 1

F2 - Acquisition Parameters
Date_ 20150826
Time 15.10
INSTRUM spect
PROBHD 5 mm PABBO BB/
PULPROG zg30
TD 65536
SOLVENT CDCl3
NS 16
DS 2
SWH 8012.820 Hz
FIDRES 0.122266 Hz
AQ 4.0894465 sec
RG 12.96
DW 62.400 usec
DE 6.50 usec
TE 294.9 K
D1 1.00000000 sec
TD0 1

======== CHANNEL f1 ========
SFO1 400.1324710 MHz
NUC1 1H
P1 10.00 usec
PLW1 25.00300026 W

F2 - Processing parameters
SI 65536
SF 400.1300000 MHz
WDW EM
SSB 0
LB 0.30 Hz
GB 0
PC 1.00

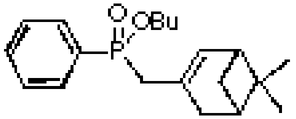

Table 3, entry 7
^{13}C NMR

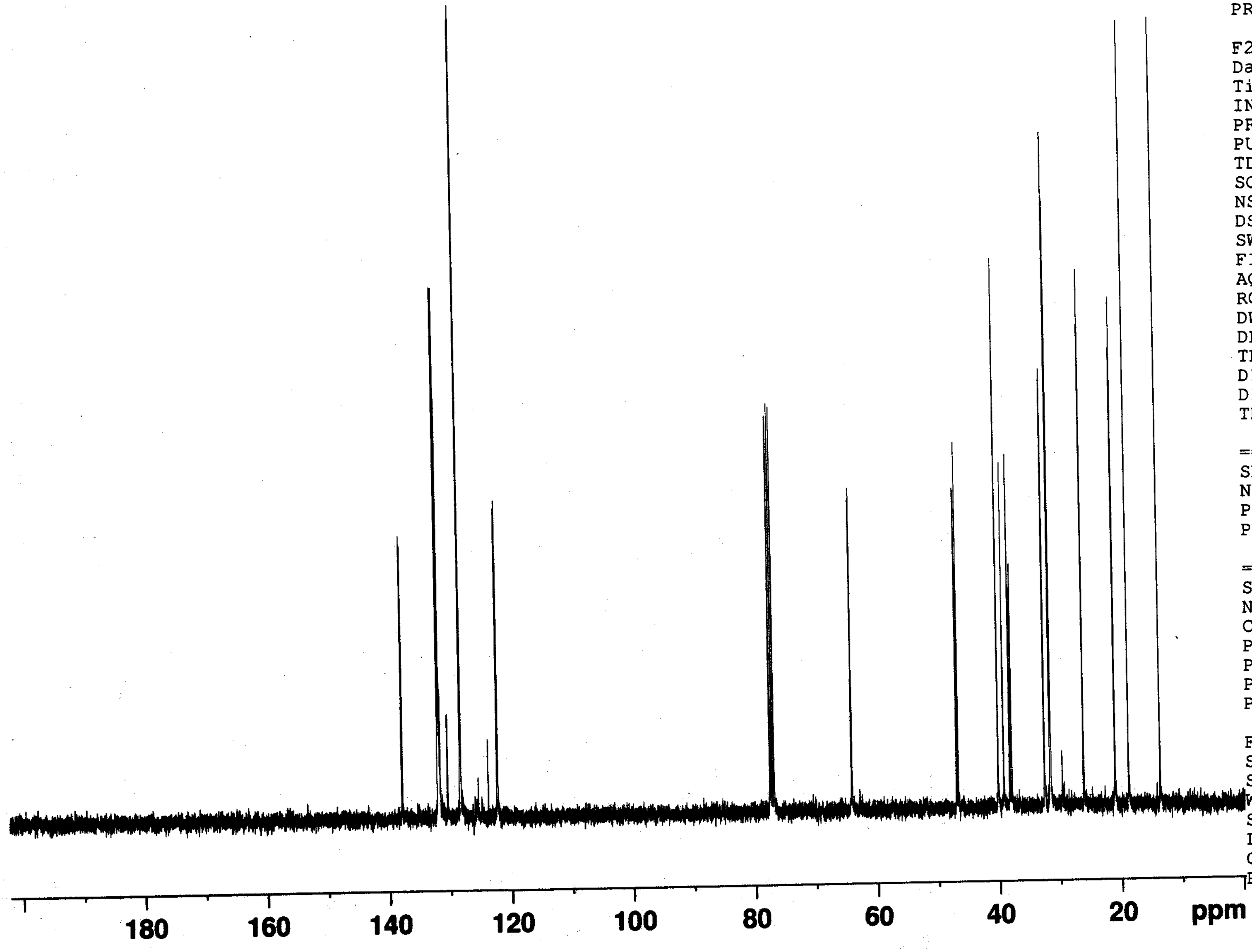

Current Data Parameters
NAME AFL071
EXPNO 6
PROCNO 1

F2 - Acquisition Parameters
Date_ 20150804
Time 15.07
INSTRUM spect
PROBHD 5 mm PABBO BB/
PULPROG zgpg30
TD 65536
SOLVENT CDCl3
NS 147
DS 4
SWH 24038.461 Hz
FIDRES 0.366798 Hz
AQ 1.3631488 sec
RG 203.57
DW 20.800 usec
DE 6.50 usec
TE 296.1 K
D1 2.00000000 sec
D11 0.03000000 sec
TD0 1

======== CHANNEL f1 ========
SFO1 100.6228293 MHz
NUC1 13C
P1 10.00 usec
PLW1 45.00000000 W

======== CHANNEL f2 ========
SFO2 400.1316005 MHz
NUC2 1H
CPDPRG[2 waltz16
PCPD2 90.00 usec
PLW2 10.00000000 W
PLW12 0.31604999 W
PLW13 0.25600001 W

F2 - Processing parameters
SI 32768
SF 100.6127685 MHz
WDW EM
SSB 0
LB 1.00 Hz
GB 0
PC 1.40

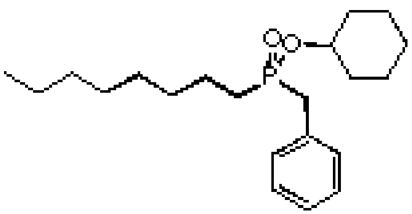

Table 3, entry 8
$^{31}P/^{1}H$ NMR decoupled

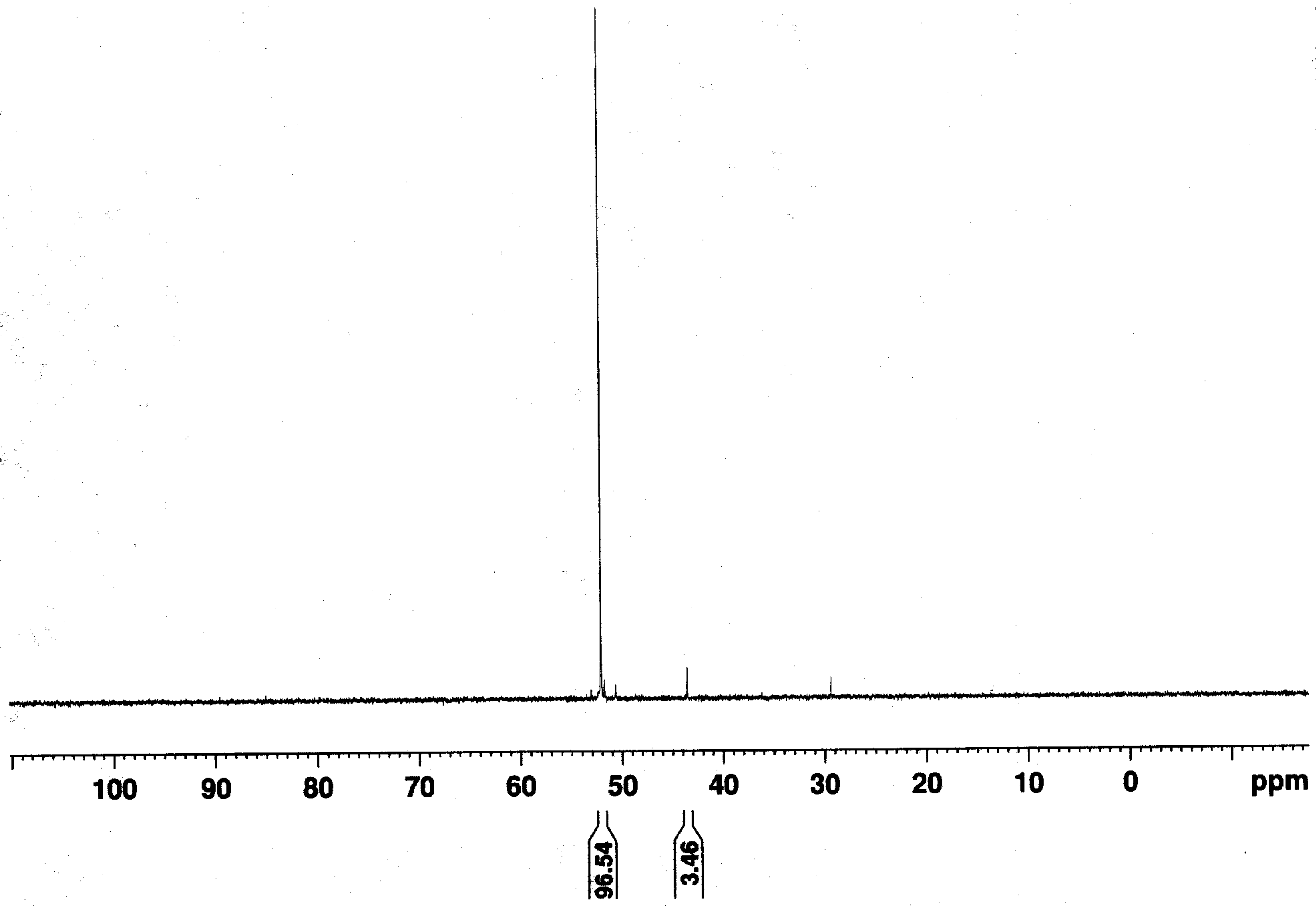


```
Current Data Parameters
NAME              AFL012un
EXPNO                    1
PROCNO                   1

F2 - Acquisition Parameters
Date_             20150625
Time                 14.34
INSTRUM              spect
PROBHD   5 mm PABBO BB/
PULPROG            zgpg30
TD                   65536
SOLVENT              CDCl3
NS                      16
DS                       4
SWH              64102.563 Hz
FIDRES            0.978127 Hz
AQ               0.5111808 sec
RG                  203.57
DW                   7.800 usec
DE                    6.50 usec
TE                   294.2 K
D1              2.00000000 sec
D11             0.03000000 sec
TD0                      1

======== CHANNEL f1 ========
SFO1           161.9674942 MHz
NUC1                   31P
P1                   14.25 usec
PLW1           15.00000000 W

======== CHANNEL f2 ========
SFO2           400.1316005 MHz
NUC2                    1H
CPDPRG[2            waltz16
PCPD2                90.00 usec
PLW2           10.00000000 W
PLW12           0.31604999 W
PLW13           0.25600001 W

F2 - Processing parameters
SI                   32768
SF             161.9755930 MHz
WDW                     EM
SSB      0
LB                    1.00 Hz
GB       0
PC                    1.40
```

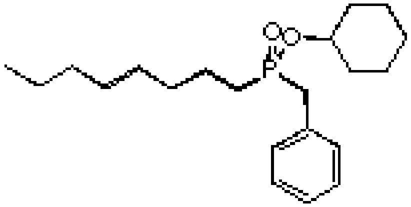

Table 3, entry 8
$^{31}P/^{1}H$ NMR coupled

Current Data Parameters
NAME AFL012un
EXPNO 2
PROCNO 1

F2 - Acquisition Parameters
Date_ 20150625
Time 14.36
INSTRUM spect
PROBHD 5 mm PABBO BB/
PULPROG zg30
TD 65536
SOLVENT CDCl3
NS 32
DS 4
SWH 64102.563 Hz
FIDRES 0.978127 Hz
AQ 0.5111808 sec
RG 203.57
DW 7.800 usec
DE 6.50 usec
TE 293.8 K
D1 2.00000000 sec
TD0 1

======== CHANNEL f1 ========
SFO1 161.9674942 MHz
NUC1 31P
P1 14.25 usec
PLW1 15.00000000 W

F2 - Processing parameters
SI 32768
SF 161.9755930 MHz
WDW EM
SSB 0
LB 1.00 Hz
GB 0
PC 1.40

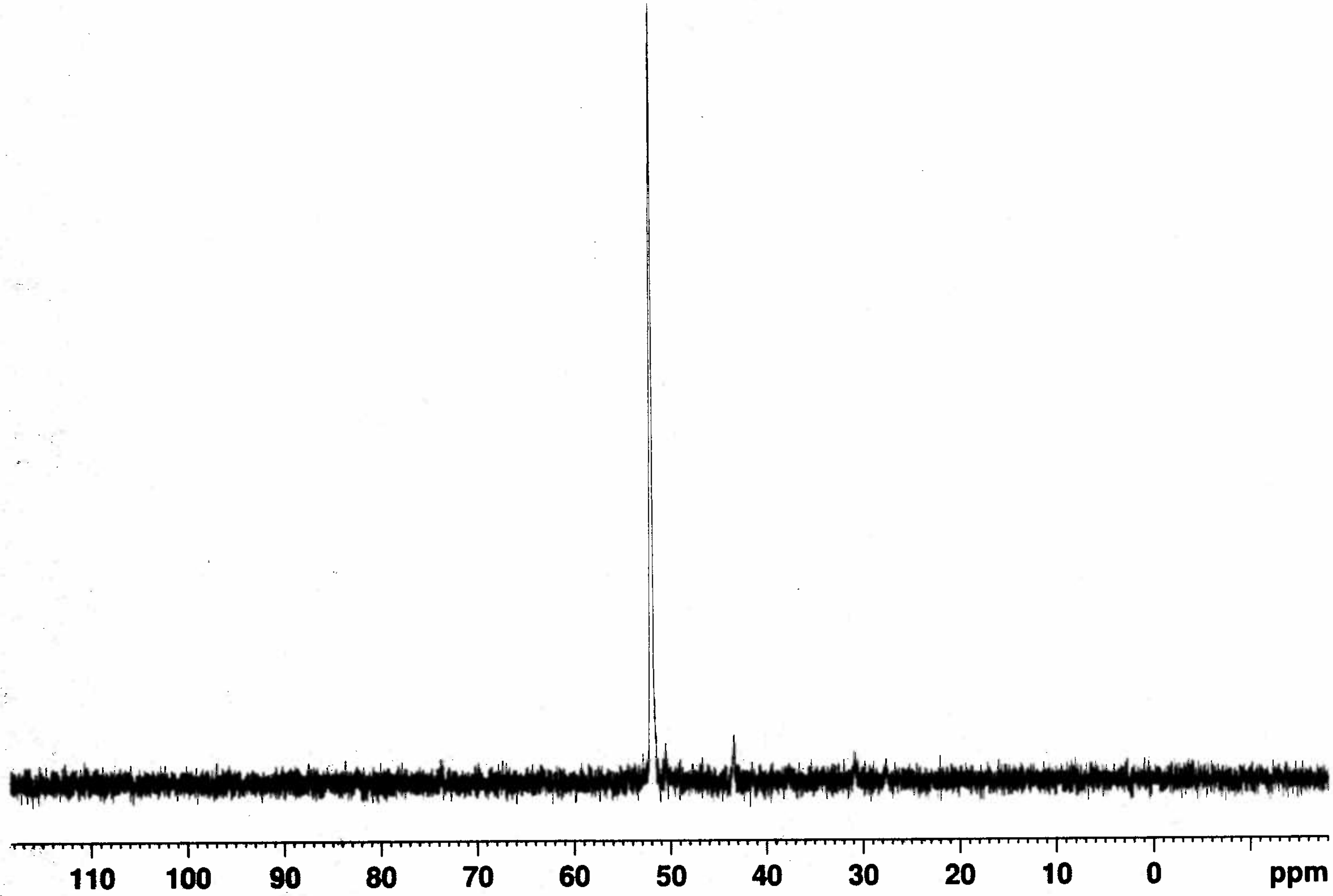

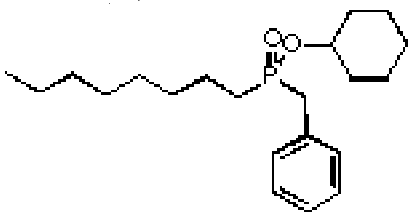

Table 3, entry 8
^{1}H NMR

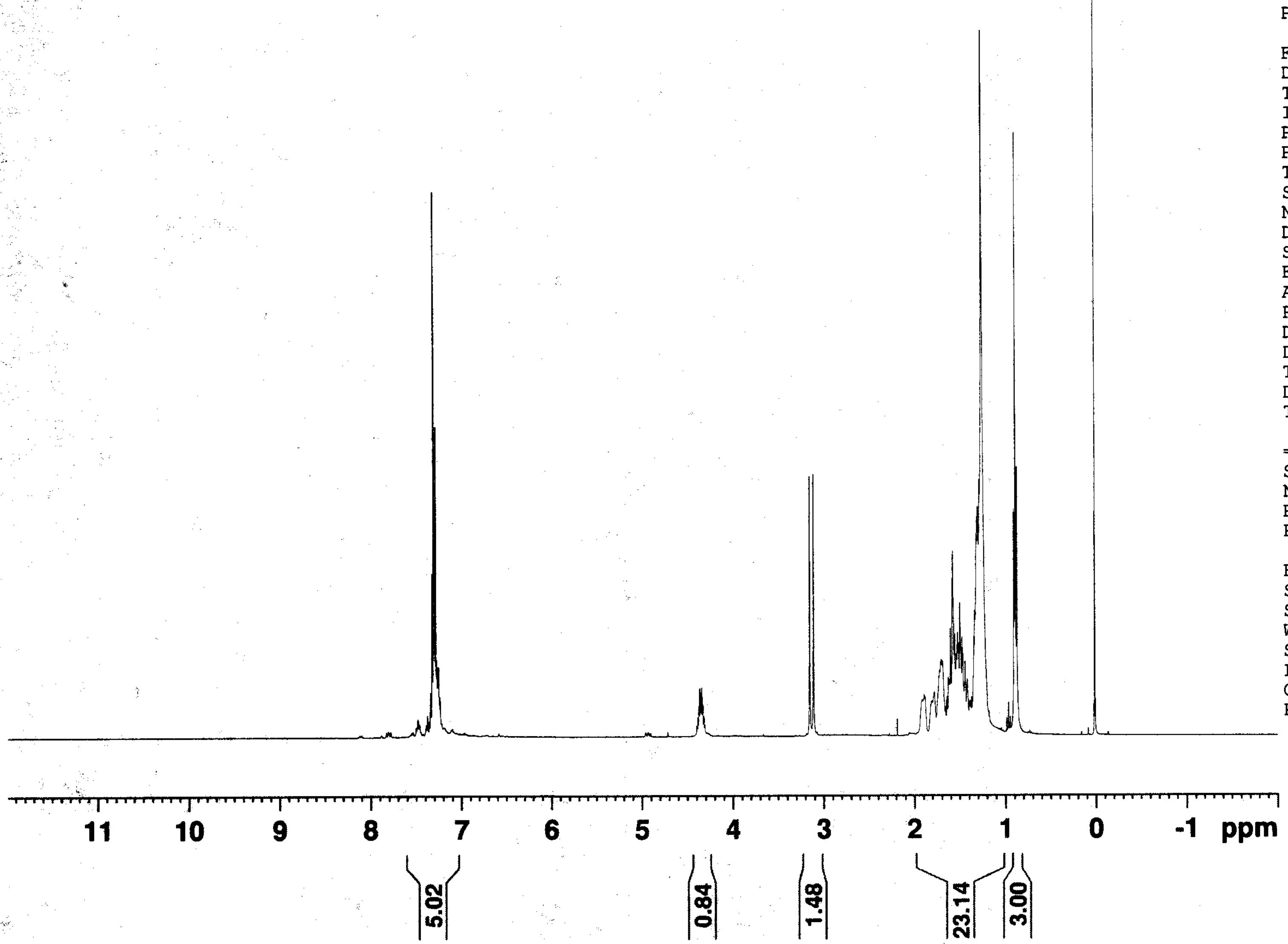

Current Data Parameters
NAME AFL012un
EXPNO 3
PROCNO 1

F2 - Acquisition Parameters
Date_ 20150625
Time 14.41
INSTRUM spect
PROBHD 5 mm PABBO BB/
PULPROG zg30
TD 65536
SOLVENT CDCl3
NS 16
DS 2
SWH 8012.820 Hz
FIDRES 0.122266 Hz
AQ 4.0894465 sec
RG 81.67
DW 62.400 usec
DE 6.50 usec
TE 293.8 K
D1 1.00000000 sec
TD0 1

======== CHANNEL f1 ========
SFO1 400.1324710 MHz
NUC1 1H
P1 10.00 usec
PLW1 25.00300026 W

F2 - Processing parameters
SI 65536
SF 400.1300000 MHz
WDW EM
SSB 0
LB 0.30 Hz
GB 0
PC 1.00

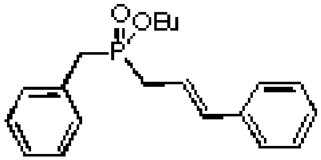

Table 3, entry 9

$^{31}P/^{1}H$ NMR decoupled

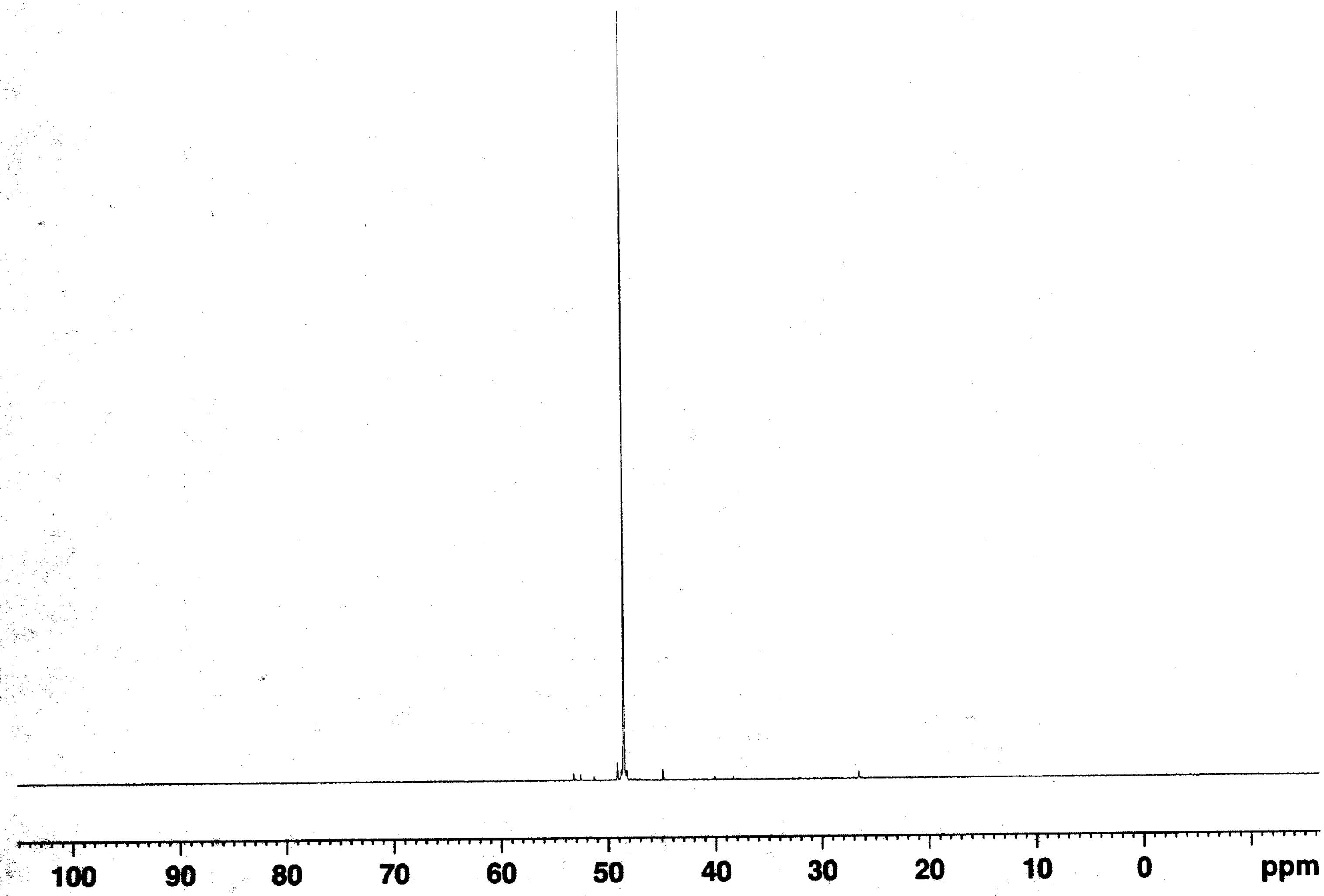

Current Data Parameters
NAME AFL063
EXPNO 3
PROCNO 1

F2 - Acquisition Parameters
Date_ 20150729
Time 13.30
INSTRUM spect
PROBHD 5 mm PABBO BB/
PULPROG zgpg30
TD 65536
SOLVENT CDCl3
NS 16
DS 4
SWH 64102.563 Hz
FIDRES 0.978127 Hz
AQ 0.5111808 sec
RG 203.57
DW 7.800 usec
DE 6.50 usec
TE 295.5 K
D1 2.00000000 sec
D11 0.03000000 sec
TD0 1

======== CHANNEL f1 ========
SFO1 161.9674942 MHz
NUC1 31P
P1 14.25 usec
PLW1 15.00000000 W

======== CHANNEL f2 ========
SFO2 400.1316005 MHz
NUC2 1H
CPDPRG[2 waltz16
PCPD2 90.00 usec
PLW2 10.00000000 W
PLW12 0.31604999 W
PLW13 0.25600001 W

F2 - Processing parameters
SI 32768
SF 161.9755930 MHz
WDW EM
SSB 0
LB 1.00 Hz
GB 0
PC 1.40

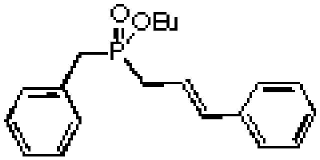

Table 3, entry 9

$^{31}P/^{1}H$ NMR coupled

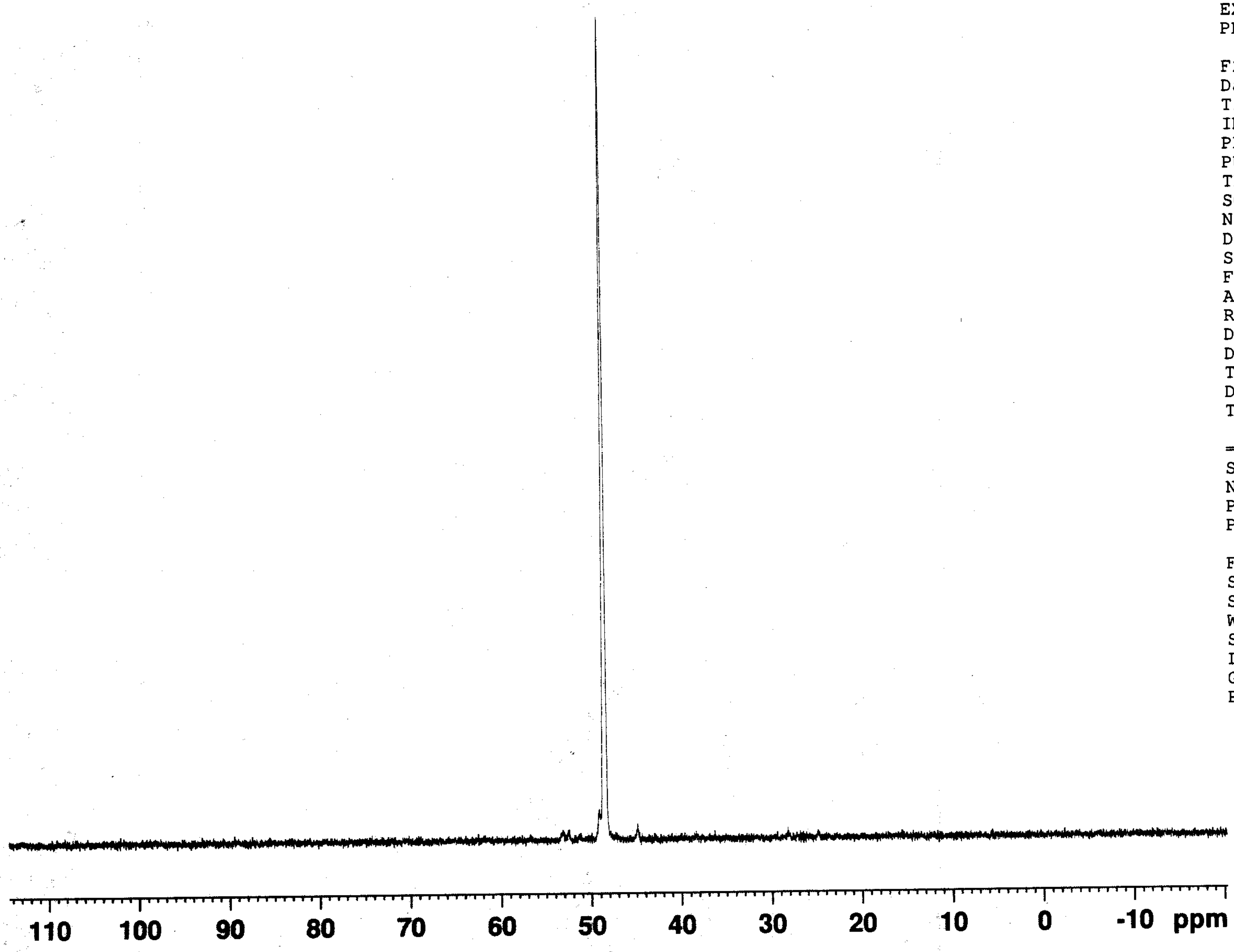


```
Current Data Parameters
NAME              AFL063
EXPNO                  4
PROCNO                 1

F2 - Acquisition Parameters
Date_           20150729
Time               13.34
INSTRUM            spect
PROBHD   5 mm PABBO BB/
PULPROG             zg30
TD                 65536
SOLVENT            CDCl3
NS                    20
DS                     4
SWH            64102.563 Hz
FIDRES          0.978127 Hz
AQ             0.5111808 sec
RG                203.57
DW                 7.800 usec
DE                  6.50 usec
TE                 295.1 K
D1            2.00000000 sec
TD0                    1

======== CHANNEL f1 ========
SFO1         161.9674942 MHz
NUC1                 31P
P1                 14.25 usec
PLW1         15.00000000 W

F2 - Processing parameters
SI                 32768
SF           161.9755930 MHz
WDW                   EM
SSB      0
LB                  1.00 Hz
GB       0
PC                  1.40
```

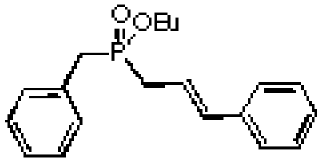

Table 3, entry 9
^{1}H NMR

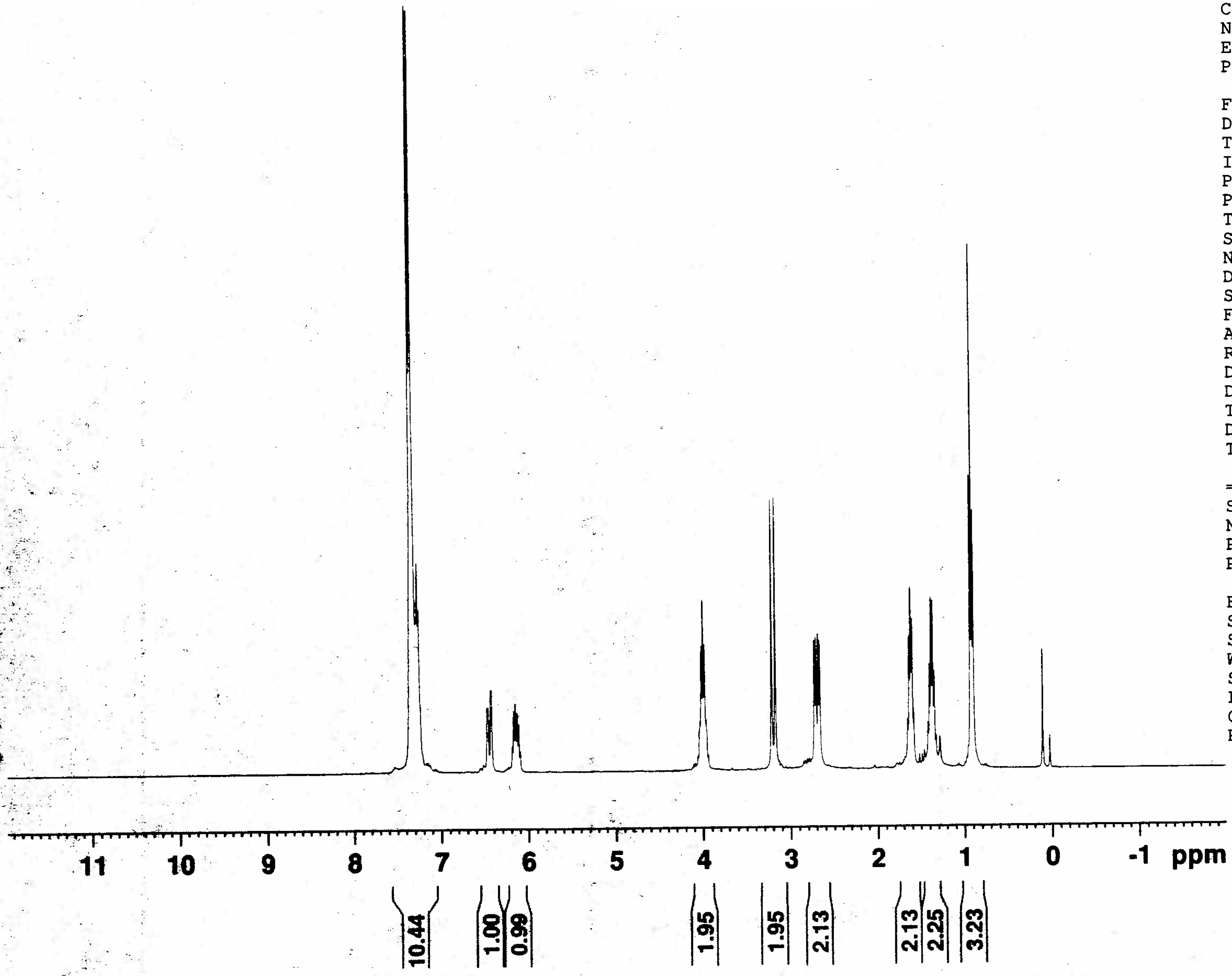

BRUKER

Current Data Parameters
NAME AFL063
EXPNO 5
PROCNO 1

F2 - Acquisition Parameters
Date_ 20150729
Time 13.39
INSTRUM spect
PROBHD 5 mm PABBO BB/
PULPROG zg30
TD 65536
SOLVENT CDCl3
NS 13
DS 2
SWH 8012.820 Hz
FIDRES 0.122266 Hz
AQ 4.0894465 sec
RG 32.38
DW 62.400 usec
DE 6.50 usec
TE 295.1 K
D1 1.00000000 sec
TD0 1

======== CHANNEL f1 ========
SFO1 400.1324710 MHz
NUC1 1H
P1 10.00 usec
PLW1 25.00300026 W

F2 - Processing parameters
SI 65536
SF 400.1300000 MHz
WDW EM
SSB 0
LB 0.30 Hz
GB 0
PC 1.00

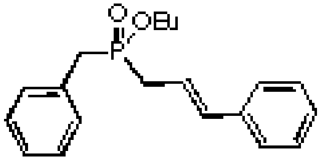

Table 3, entry 9
^{13}C NMR

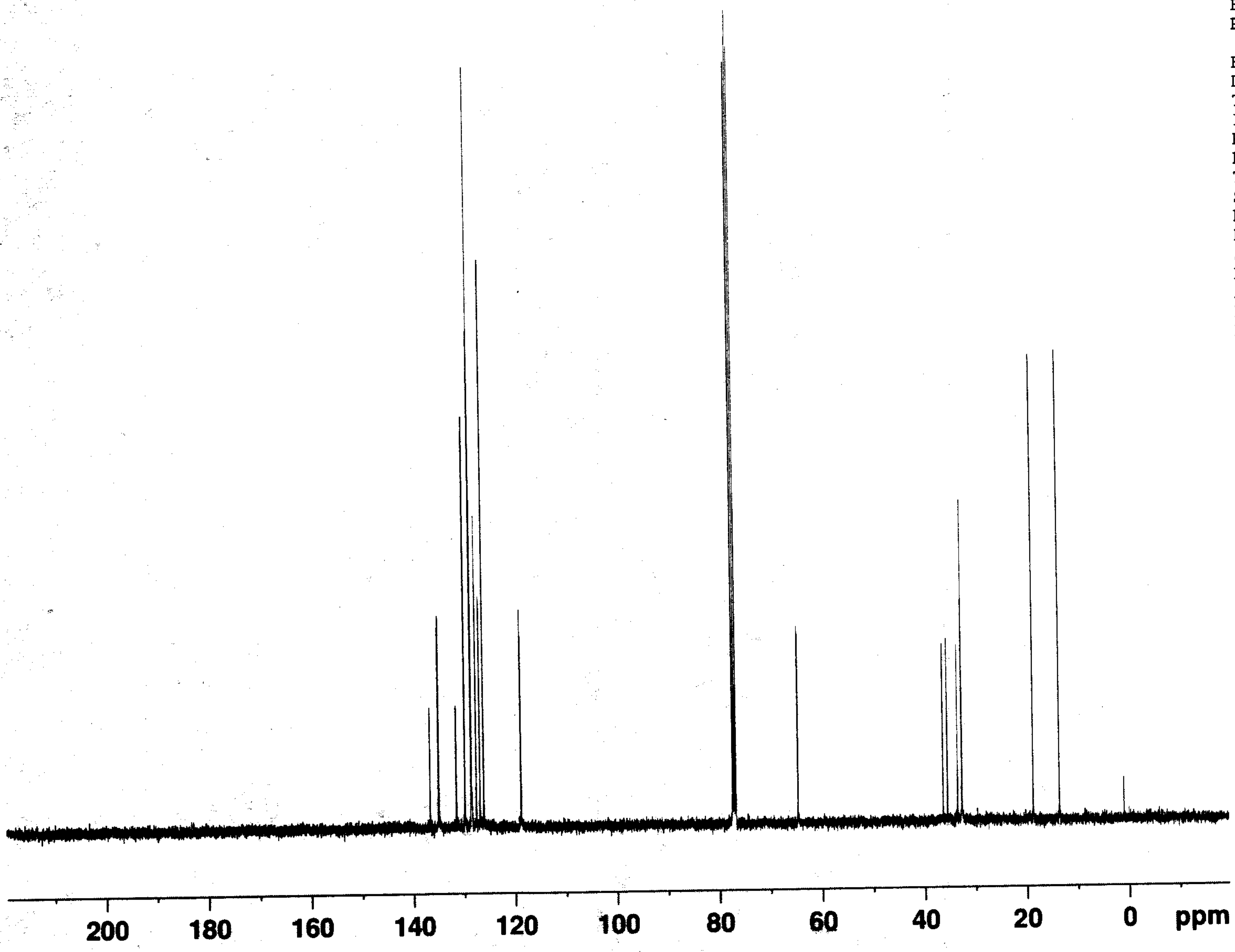


```
Current Data Parameters
NAME              AFL063
EXPNO                  6
PROCNO                 1

F2 - Acquisition Parameters
Date_           20150729
Time               13.49
INSTRUM            spect
PROBHD   5 mm PABBO BB/
PULPROG          zgpg30
TD                 65536
SOLVENT            CDCl3
NS                   135
DS                     4
SWH            24038.461 Hz
FIDRES          0.366798 Hz
AQ             1.3631488 sec
RG                203.57
DW                20.800 usec
DE                  6.50 usec
TE                 295.9 K
D1            2.00000000 sec
D11           0.03000000 sec
TD0                    1

======== CHANNEL f1 ========
SFO1         100.6228293 MHz
NUC1                 13C
P1                 10.00 usec
PLW1         45.00000000 W

======== CHANNEL f2 ========
SFO2         400.1316005 MHz
NUC2                  1H
CPDPRG[2         waltz16
PCPD2              90.00 usec
PLW2         10.00000000 W
PLW12         0.31604999 W
PLW13         0.25600001 W

F2 - Processing parameters
SI                 32768
SF           100.6127685 MHz
WDW                   EM
SSB      0
LB                  1.00 Hz
GB       0
PC                  1.40
```

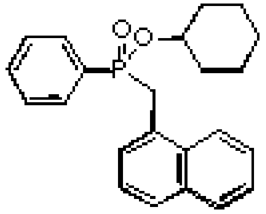

Table 3, entry 10
$^{31}P/^{1}H$ NMR decoupled

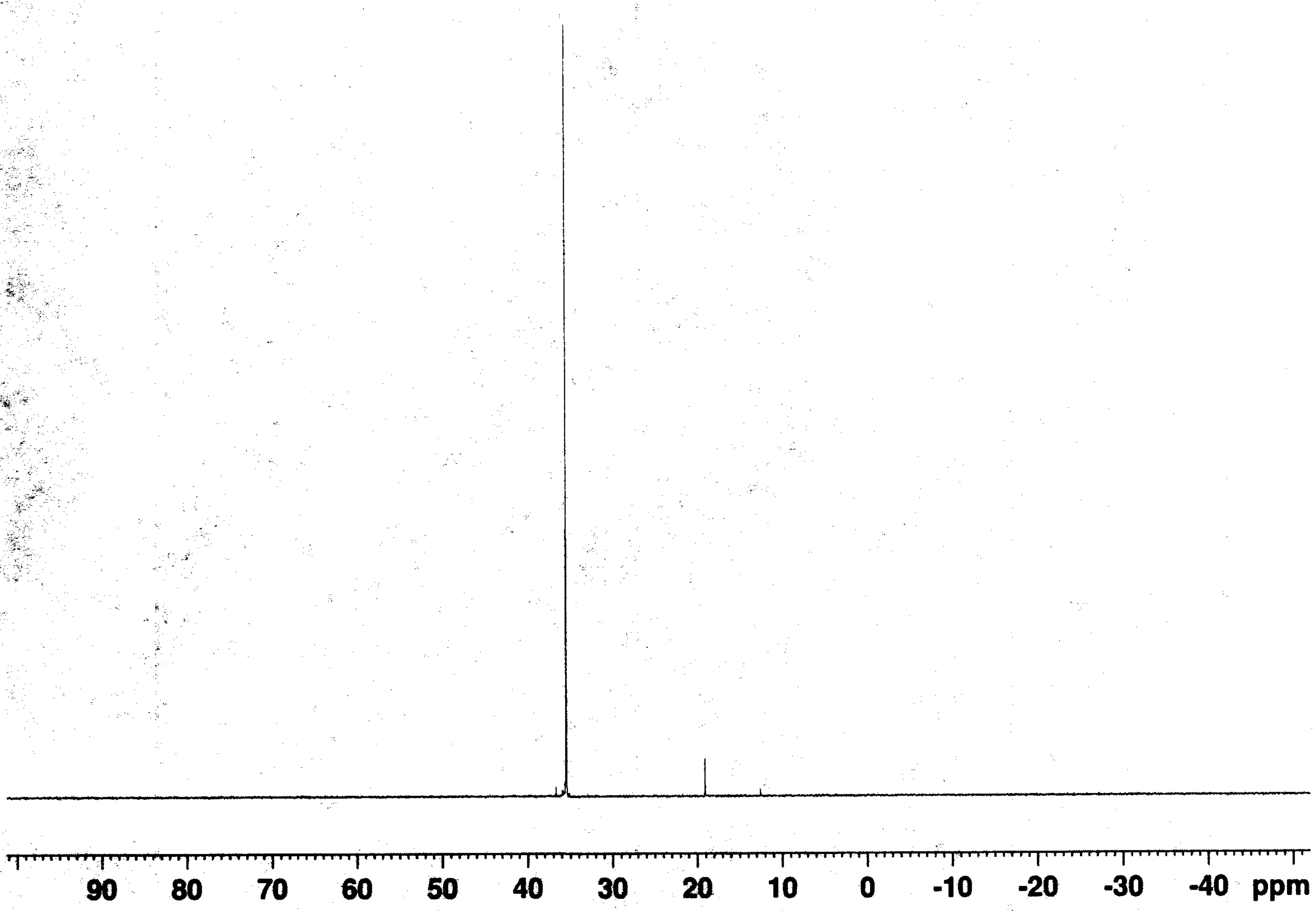


```
Current Data Parameters
NAME              AFL069
EXPNO                 14
PROCNO                 1

F2 - Acquisition Parameters
Date_           20150806
Time               15.14
INSTRUM            spect
PROBHD   5 mm PABBO BB/
PULPROG          zgpg30
TD                 65536
SOLVENT          Acetone
NS                    16
DS                     4
SWH          64102.563 Hz
FIDRES        0.978127 Hz
AQ           0.5111808 sec
RG              203.57
DW               7.800 usec
DE                6.50 usec
TE               296.1 K
D1          2.00000000 sec
D11         0.03000000 sec
TD0                    1

======== CHANNEL f1 ========
SFO1       161.9674942 MHz
NUC1                31P
P1               14.25 usec
PLW1       15.00000000 W

======== CHANNEL f2 ========
SFO2       400.1316005 MHz
NUC2                 1H
CPDPRG[2         waltz16
PCPD2            90.00 usec
PLW2       10.00000000 W
PLW12       0.31604999 W
PLW13       0.25600001 W

F2 - Processing parameters
SI                 32768
SF         161.9755930 MHz
WDW                   EM
SSB      0
LB                1.00 Hz
GB       0
PC                1.40
```

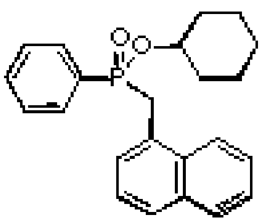

Table 3, entry 10
$^{31}P/^{1}H$ NMR coupled

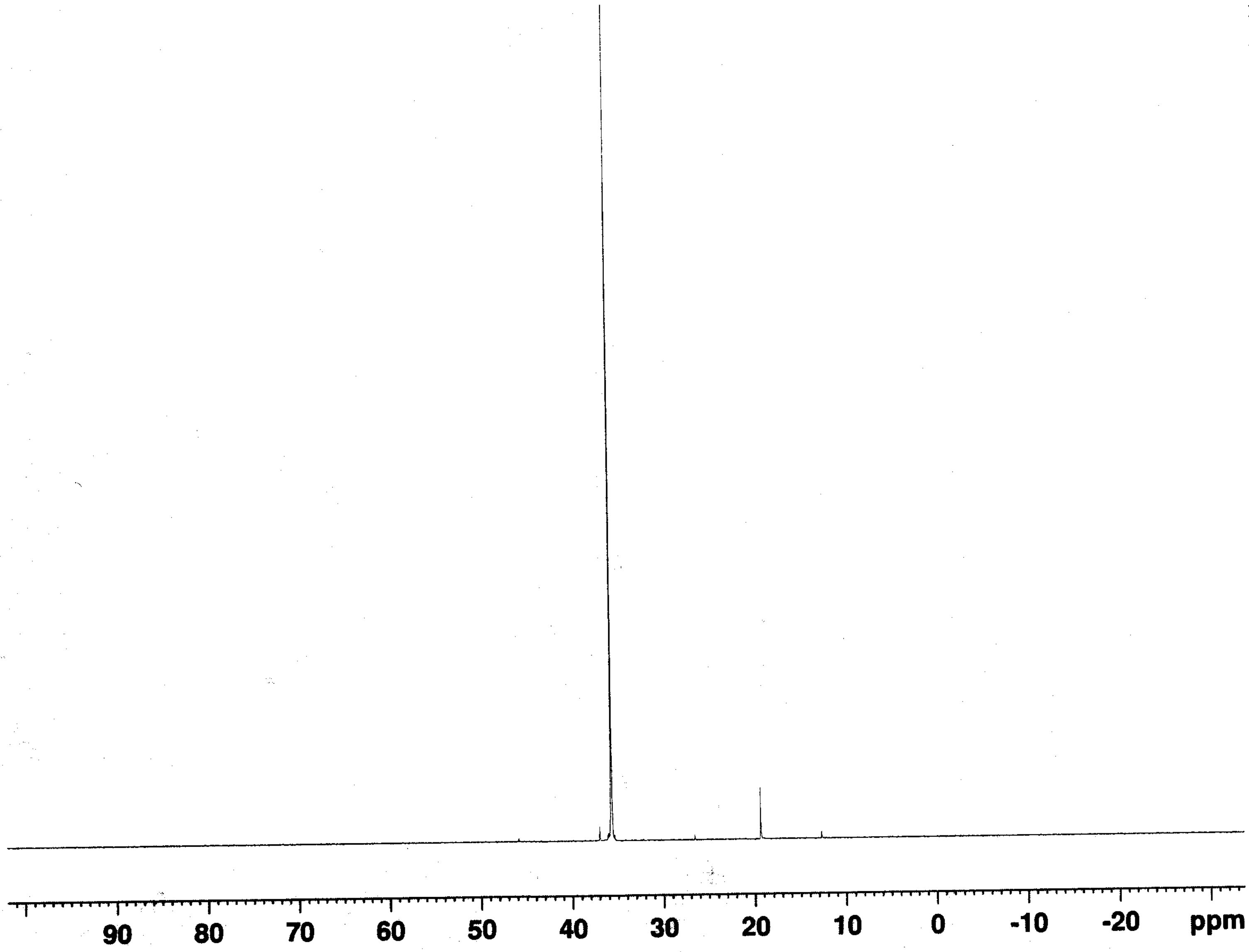

Current Data Parameters
NAME AFL069
EXPNO 15
PROCNO 1

F2 - Acquisition Parameters
Date_ 20150806
Time 16.06
INSTRUM spect
PROBHD 5 mm PABBO BB/
PULPROG zgpg30
TD 65536
SOLVENT Acetone
NS 16
DS 4
SWH 64102.563 Hz
FIDRES 0.978127 Hz
AQ 0.5111808 sec
RG 203.57
DW 7.800 usec
DE 6.50 usec
TE 296.2 K
D1 2.00000000 sec
D11 0.03000000 sec
TD0 1

======== CHANNEL f1 ========
SFO1 161.9674942 MHz
NUC1 31P
P1 14.25 usec
PLW1 15.00000000 W

======== CHANNEL f2 ========
SFO2 400.1316005 MHz
NUC2 1H
CPDPRG[2 waltz16
PCPD2 90.00 usec
PLW2 10.00000000 W
PLW12 0.31604999 W
PLW13 0.25600001 W

F2 - Processing parameters
SI 32768
SF 161.9755930 MHz
WDW EM
SSB 0
LB 1.00 Hz
GB 0
PC 1.40

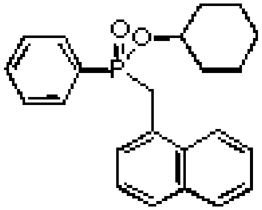

Table 3, entry 10
^{1}H NMR

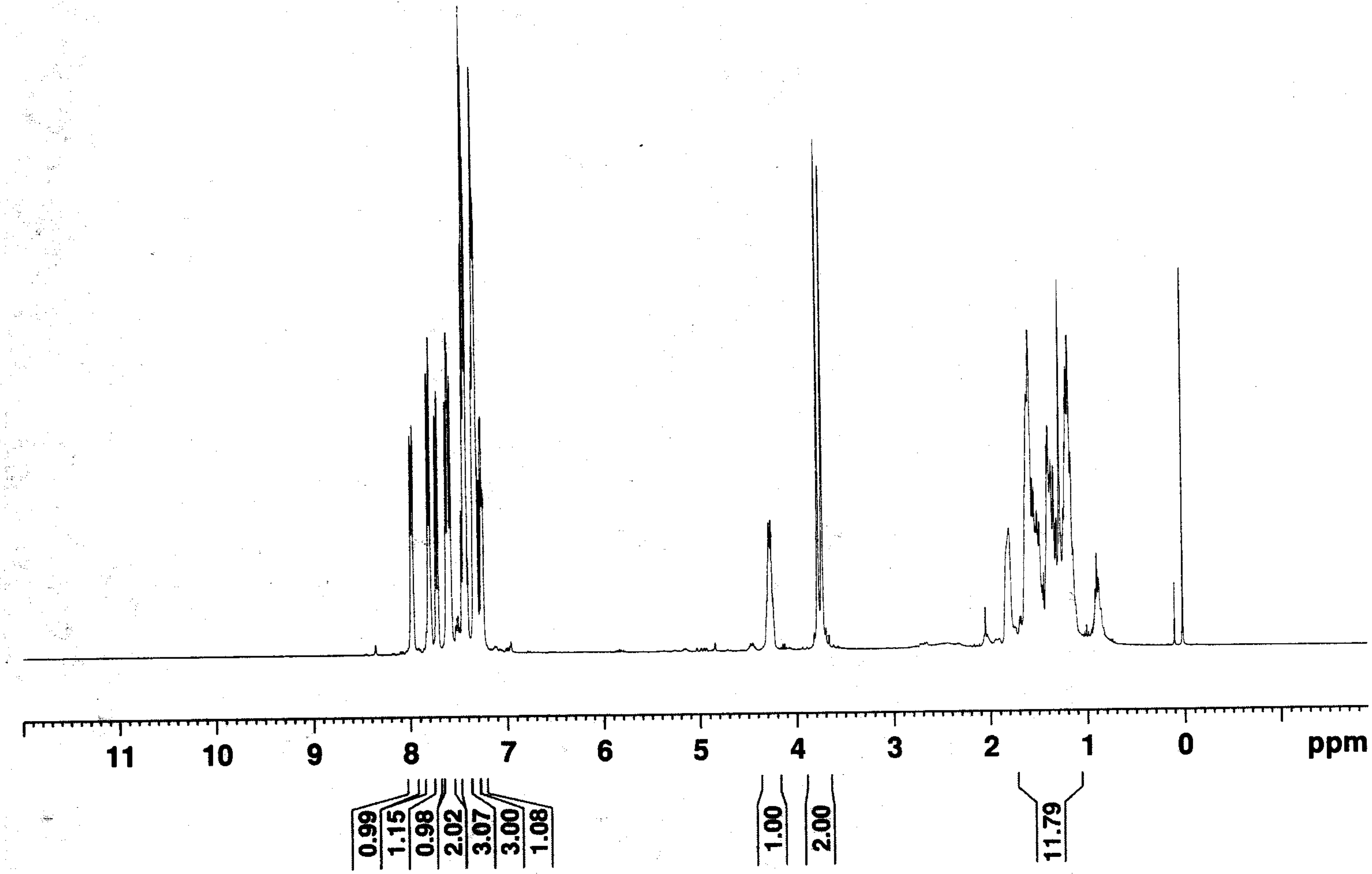


```
Current Data Parameters
NAME              AFL069
EXPNO                 17
PROCNO                 1

F2 - Acquisition Parameters
Date_           20150827
Time               16.00
INSTRUM            spect
PROBHD   5 mm PABBO BB/
PULPROG             zg30
TD                 65536
SOLVENT            CDCl3
NS                    16
DS                     2
SWH             8012.820 Hz
FIDRES          0.122266 Hz
AQ             4.0894465 sec
RG                 32.38
DW                62.400 usec
DE                  6.50 usec
TE                 295.0 K
D1            1.00000000 sec
TD0                    1

======== CHANNEL f1 ========
SFO1         400.1324710 MHz
NUC1                  1H
P1                 10.00 usec
PLW1         25.00300026 W

F2 - Processing parameters
SI                 65536
SF           400.1300000 MHz
WDW                   EM
SSB       0
LB                  0.30 Hz
GB        0
PC                  1.00
```

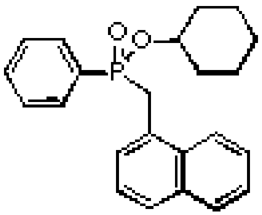

Table 3, entry 10
^{13}C NMR

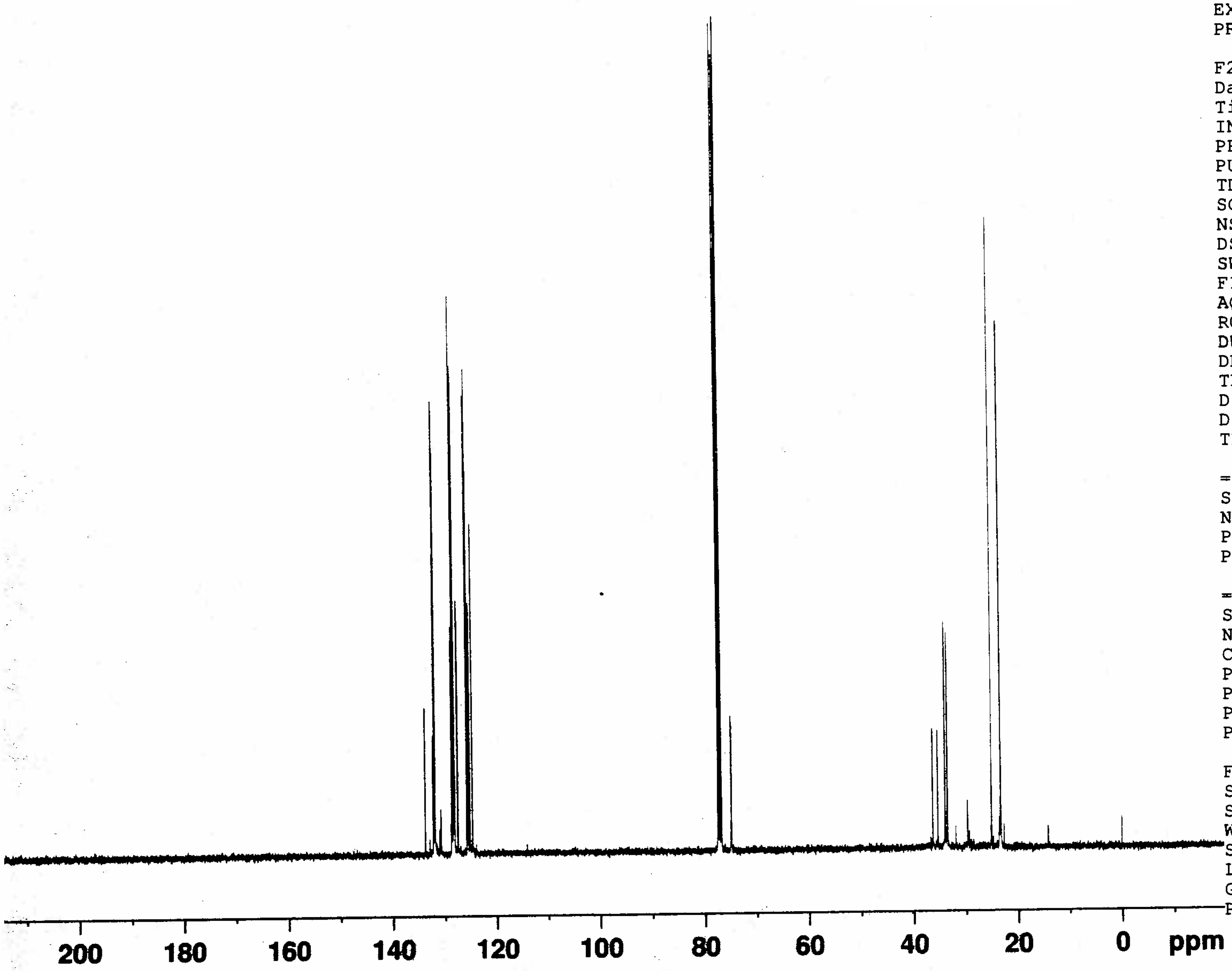

Current Data Parameters
NAME AFL069
EXPNO 16
PROCNO 1

F2 - Acquisition Parameters
Date_ 20150827
Time 15.54
INSTRUM spect
PROBHD 5 mm PABBO BB/
PULPROG zgpg30
TD 65536
SOLVENT CDCl3
NS 701
DS 4
SWH 24038.461 Hz
FIDRES 0.366798 Hz
AQ 1.3631488 sec
RG 203.57
DW 20.800 usec
DE 6.50 usec
TE 295.7 K
D1 2.00000000 sec
D11 0.03000000 sec
TD0 1

======== CHANNEL f1 ========
SFO1 100.6228293 MHz
NUC1 13C
P1 10.00 usec
PLW1 45.00000000 W

======== CHANNEL f2 ========
SFO2 400.1316005 MHz
NUC2 1H
CPDPRG[2 waltz16
PCPD2 90.00 usec
PLW2 10.00000000 W
PLW12 0.31604999 W
PLW13 0.25600001 W

F2 - Processing parameters
SI 32768
SF 100.6127685 MHz
WDW EM
SSB 0
LB 1.00 Hz
GB 0
PC 1.40

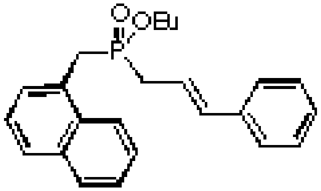

Table 3, entry 11
$^{31}P/^{1}H$ NMR decoupled

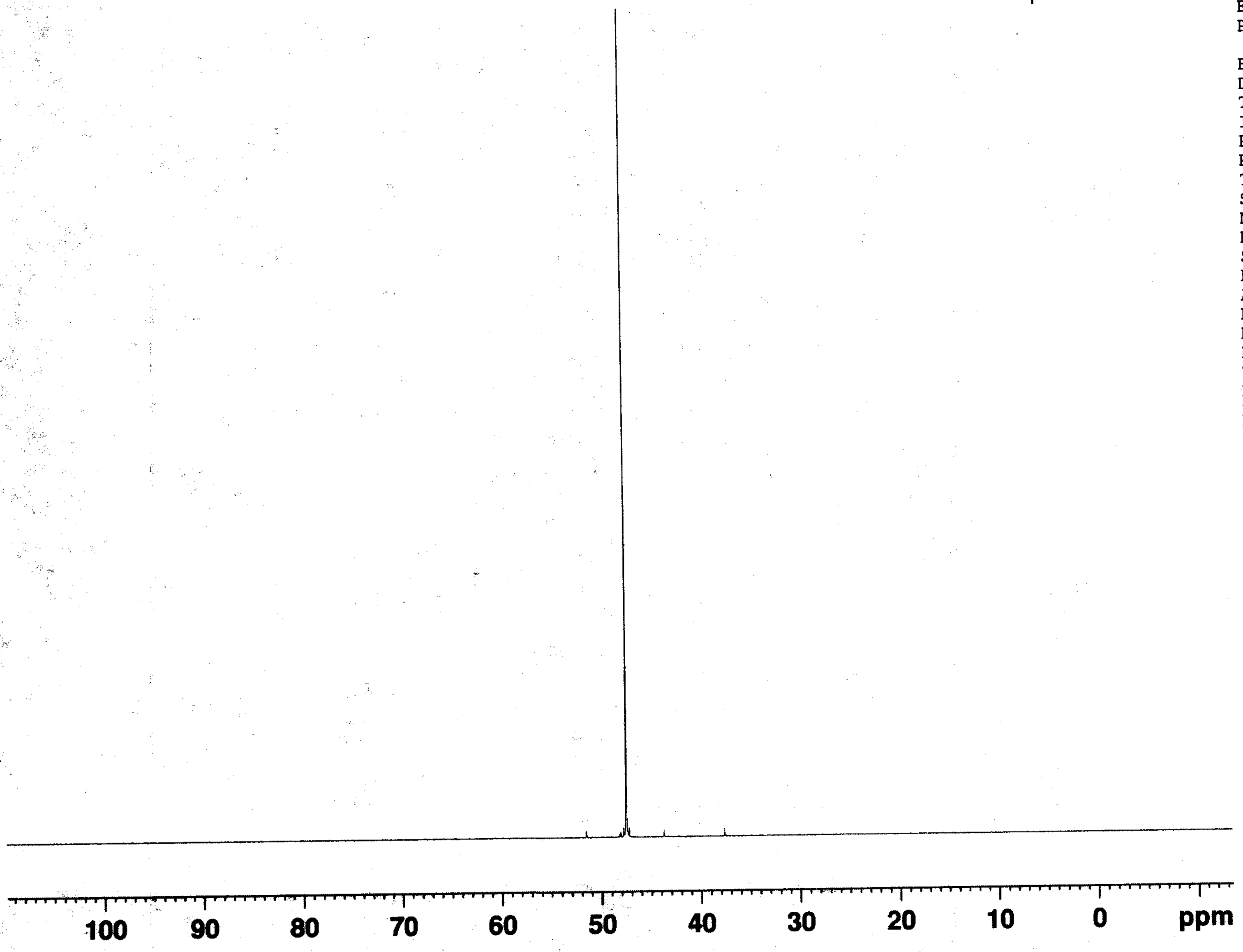


```
Current Data Parameters
NAME              AFL089
EXPNO                  3
PROCNO                 1

F2 - Acquisition Parameters
Date_           20150812
Time                9.22
INSTRUM            spect
PROBHD   5 mm PABBO BB/
PULPROG          zgpg30
TD                65536
SOLVENT          Acetone
NS                    16
DS                     4
SWH           64102.563 Hz
FIDRES         0.978127 Hz
AQ            0.5111808 sec
RG                203.57
DW                 7.800 usec
DE                  6.50 usec
TE                 295.1 K
D1            2.00000000 sec
D11           0.03000000 sec
TD0                    1

======== CHANNEL f1 ========
SFO1         161.9674942 MHz
NUC1                 31P
P1                 14.25 usec
PLW1         15.00000000 W

======== CHANNEL f2 ========
SFO2         400.1316005 MHz
NUC2                  1H
CPDPRG[2          waltz16
PCPD2              90.00 usec
PLW2         10.00000000 W
PLW12         0.31604999 W
PLW13         0.25600001 W

F2 - Processing parameters
SI                 32768
SF           161.9755930 MHz
WDW                   EM
SSB      0
LB                  1.00 Hz
GB       0
PC                  1.40
```

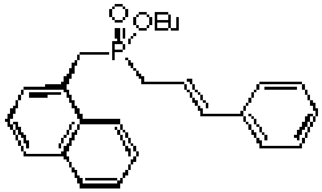

Table 3, entry 11

$^{31}P/^{1}H$ NMR coupled

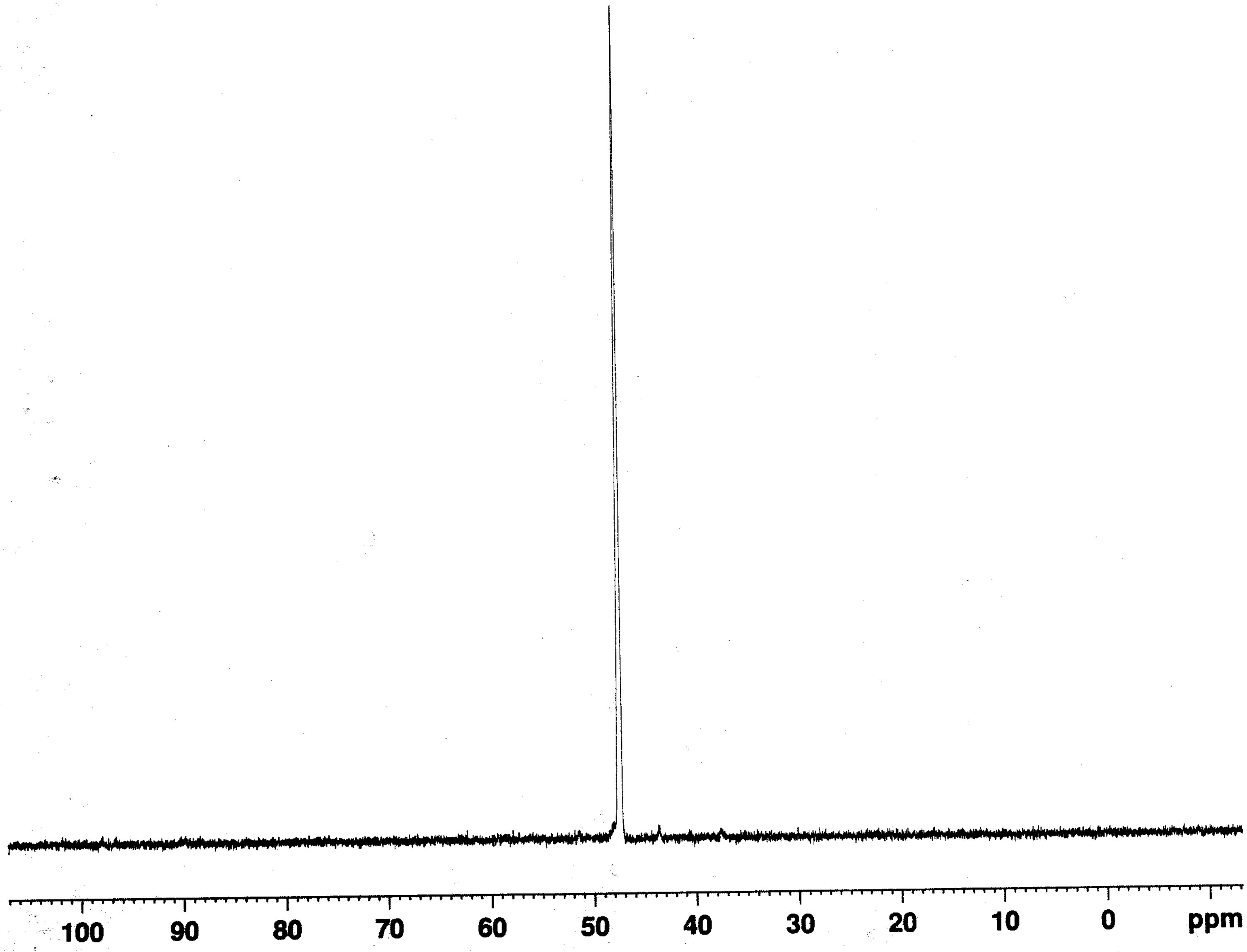

Current Data Parameters
NAME AFL089
EXPNO 4
PROCNO 1

F2 - Acquisition Parameters
Date_ 20150812
Time 9.23
INSTRUM spect
PROBHD 5 mm PABBO BB/
PULPROG zg30
TD 65536
SOLVENT Acetone
NS 10
DS 4
SWH 64102.563 Hz
FIDRES 0.978127 Hz
AQ 0.5111808 sec
RG 203.57
DW 7.800 usec
DE 6.50 usec
TE 294.7 K
D1 2.00000000 sec
TD0 1

======== CHANNEL f1 ========
SFO1 161.9674942 MHz
NUC1 31P
P1 14.25 usec
PLW1 15.00000000 W

F2 - Processing parameters
SI 32768
SF 161.9755930 MHz
WDW EM
SSB 0
LB 1.00 Hz
GB 0
PC 1.40

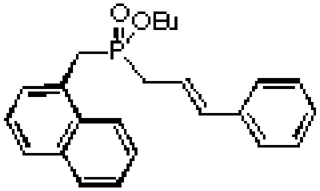

Table 3, entry 11
^{1}H NMR

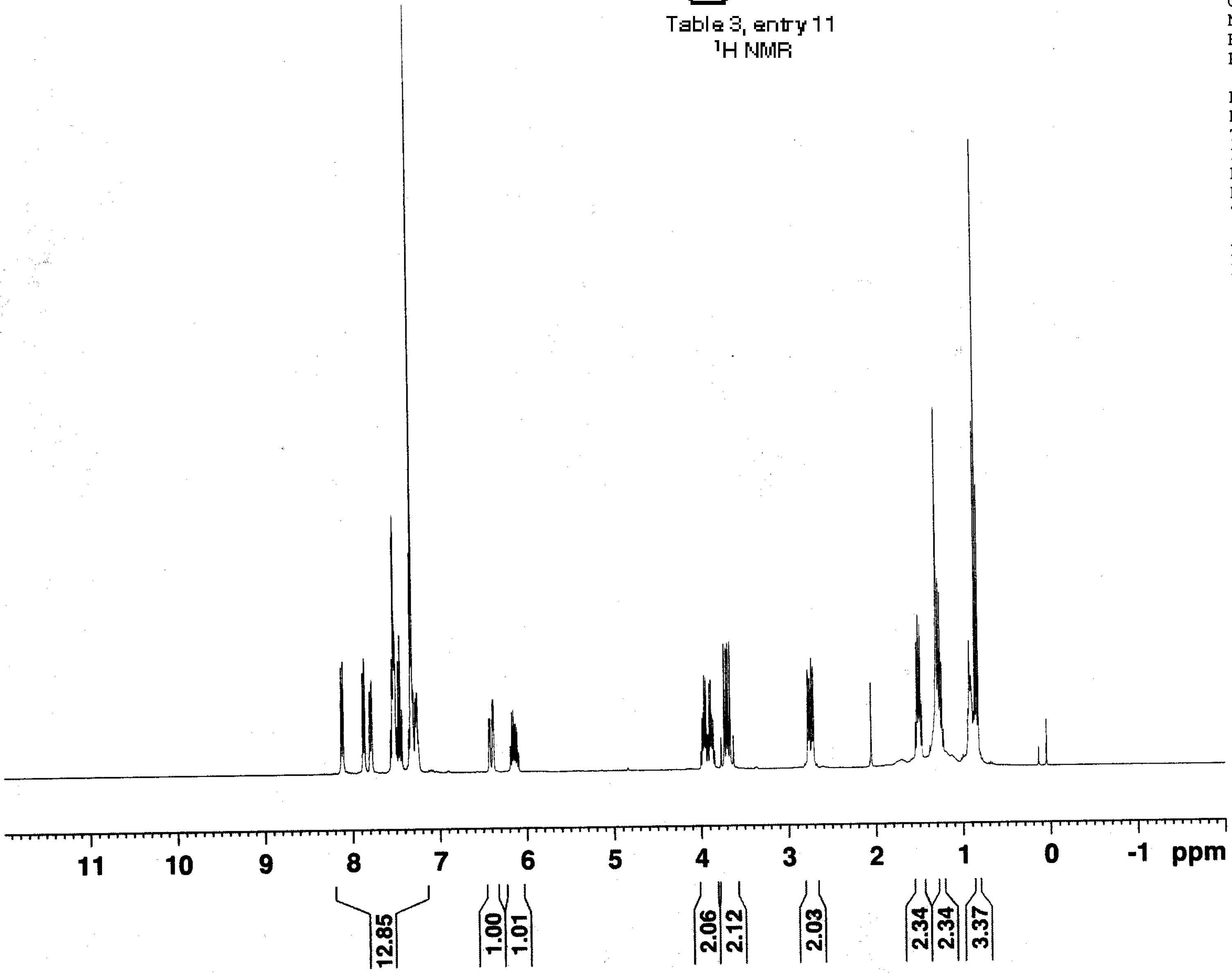


```
Current Data Parameters
NAME              AFL089
EXPNO                  5
PROCNO                 1

F2 - Acquisition Parameters
Date_           20150812
Time                9.30
INSTRUM            spect
PROBHD   5 mm PABBO BB/
PULPROG             zg30
TD                 65536
SOLVENT            CDCl3
NS                    16
DS                     2
SWH             8012.820 Hz
FIDRES          0.122266 Hz
AQ             4.0894465 sec
RG                 17.56
DW                62.400 usec
DE                  6.50 usec
TE                 294.6 K
D1            1.00000000 sec
TD0                    1

======== CHANNEL f1 ========
SFO1         400.1324710 MHz
NUC1                  1H
P1                 10.00 usec
PLW1         25.00300026 W

F2 - Processing parameters
SI                 65536
SF           400.1300000 MHz
WDW                   EM
SSB      0
LB                  0.30 Hz
GB       0
PC                  1.00
```

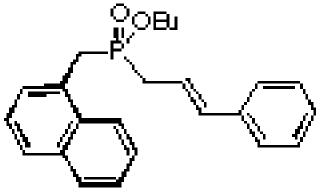

Table 3, entry 11
^{13}C NMR

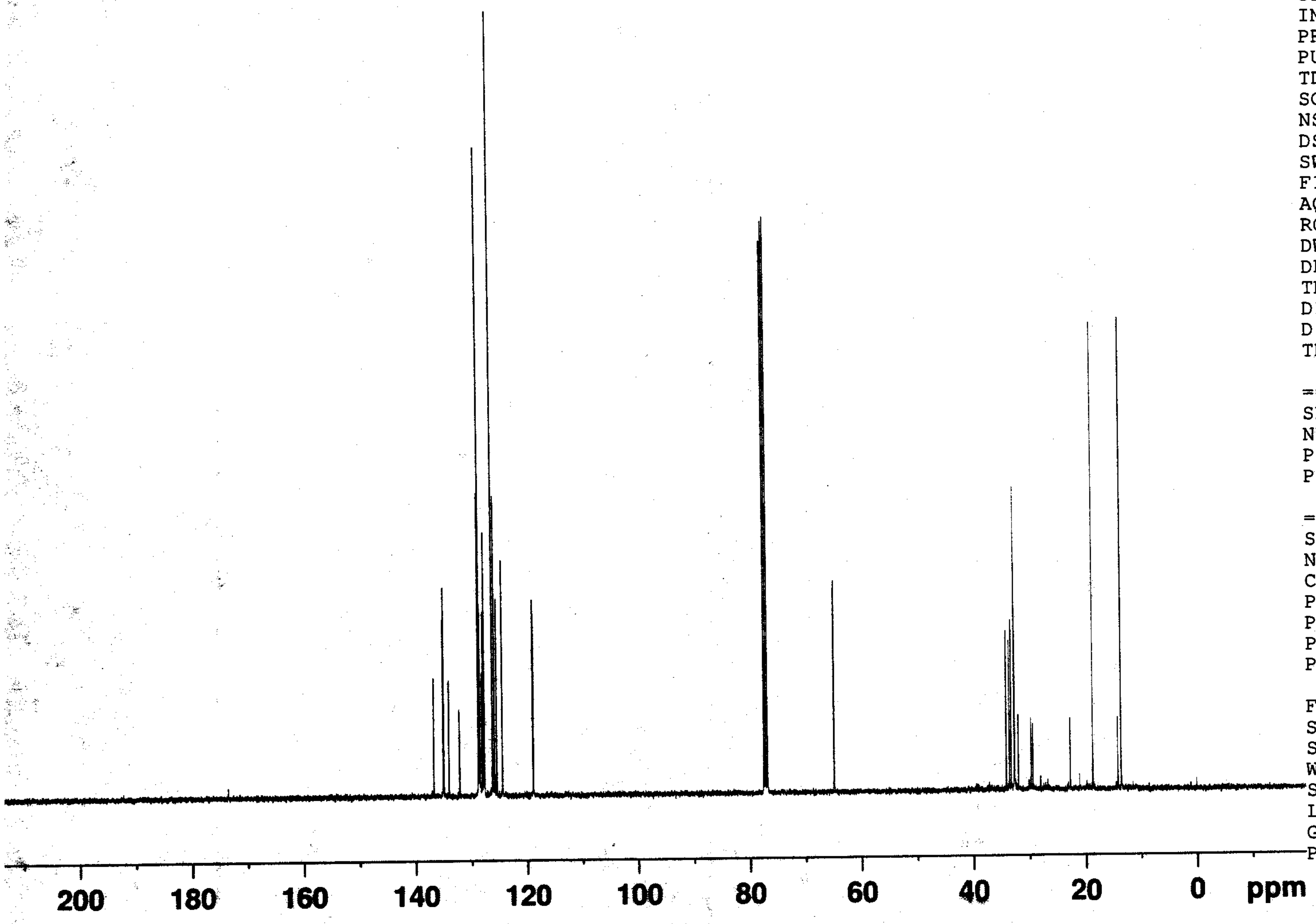

Current Data Parameters
NAME AFL089
EXPNO 6
PROCNO 1

F2 - Acquisition Parameters
Date_ 20150812
Time 9.55
INSTRUM spect
PROBHD 5 mm PABBO BB/
PULPROG zgpg30
TD 65536
SOLVENT CDCl3
NS 297
DS 4
SWH 24038.461 Hz
FIDRES 0.366798 Hz
AQ 1.3631488 sec
RG 203.57
DW 20.800 usec
DE 6.50 usec
TE 295.6 K
D1 2.00000000 sec
D11 0.03000000 sec
TD0 1

======== CHANNEL f1 ========
SFO1 100.6228293 MHz
NUC1 13C
P1 10.00 usec
PLW1 45.00000000 W

======== CHANNEL f2 ========
SFO2 400.1316005 MHz
NUC2 1H
CPDPRG[2 waltz16
PCPD2 90.00 usec
PLW2 10.00000000 W
PLW12 0.31604999 W
PLW13 0.25600001 W

F2 - Processing parameters
SI 32768
SF 100.6127685 MHz
WDW EM
SSB 0
LB 1.00 Hz
GB 0
PC 1.40

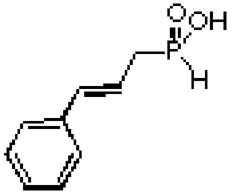

Scheme 3c

$^{31}P/^{1}H$ NMR decoupled

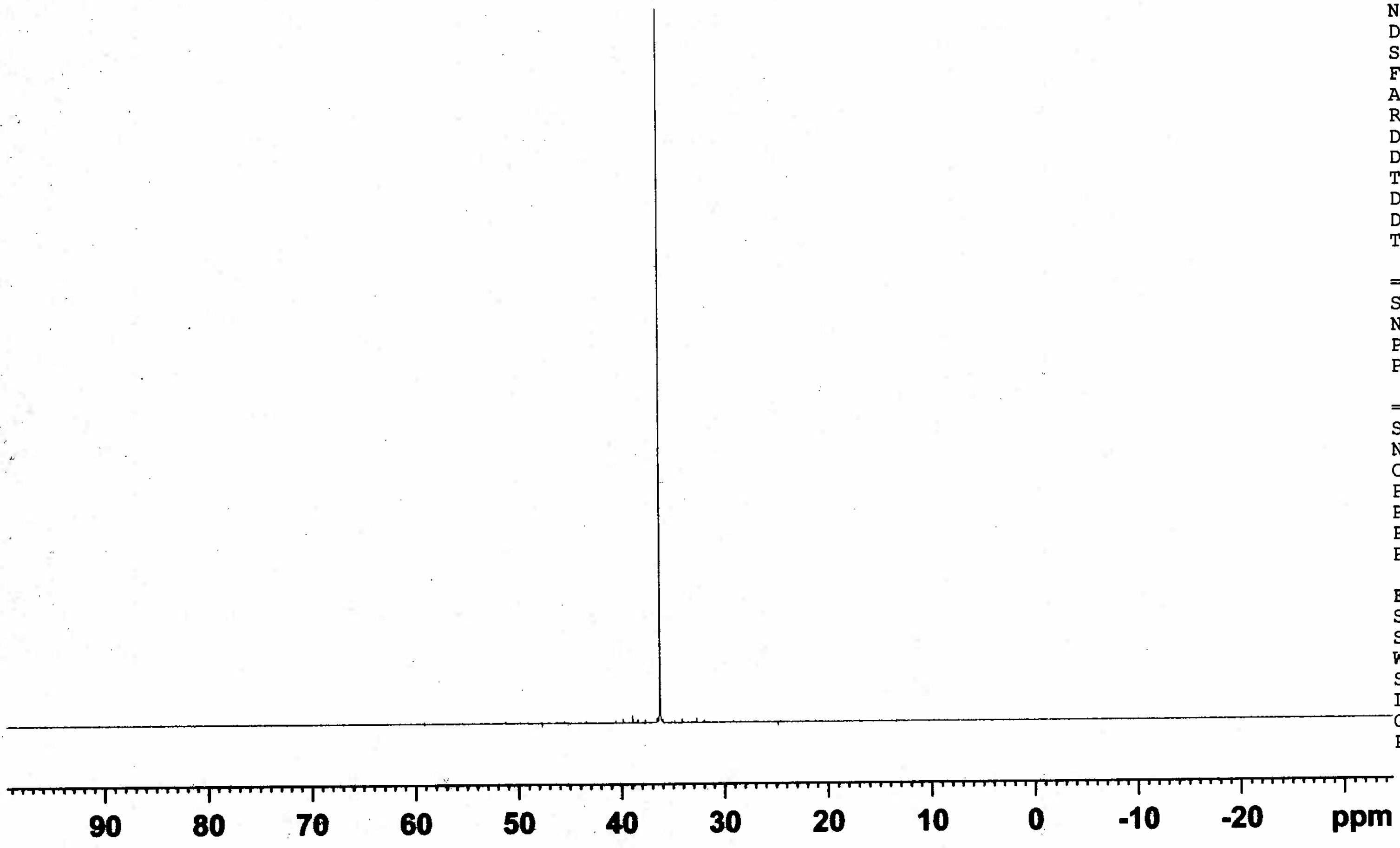

Current Data Parameters
NAME OB 1971 after column
EXPNO 1
PROCNO 1

F2 - Acquisition Parameters
Date_ 20150325
Time 17.06
INSTRUM spect
PROBHD 5 mm PABBO BB/
PULPROG zgpg30
TD 65536
SOLVENT CDCl3
NS 16
DS 4
SWH 64102.563 Hz
FIDRES 0.978127 Hz
AQ 0.5111808 sec
RG 203.57
DW 7.800 usec
DE 6.50 usec
TE 294.4 K
D1 2.00000000 sec
D11 0.03000000 sec
TD0 1

======== CHANNEL f1 ========
SFO1 161.9674942 MHz
NUC1 31P
P1 14.25 usec
PLW1 15.00000000 W

======== CHANNEL f2 ========
SFO2 400.1316005 MHz
NUC2 1H
CPDPRG[2 waltz16
PCPD2 90.00 usec
PLW2 10.00000000 W
PLW12 0.31604999 W
PLW13 0.25600001 W

F2 - Processing parameters
SI 32768
SF 161.9755930 MHz
WDW EM
SSB 0
LB 1.00 Hz
GB 0
PC 1.40

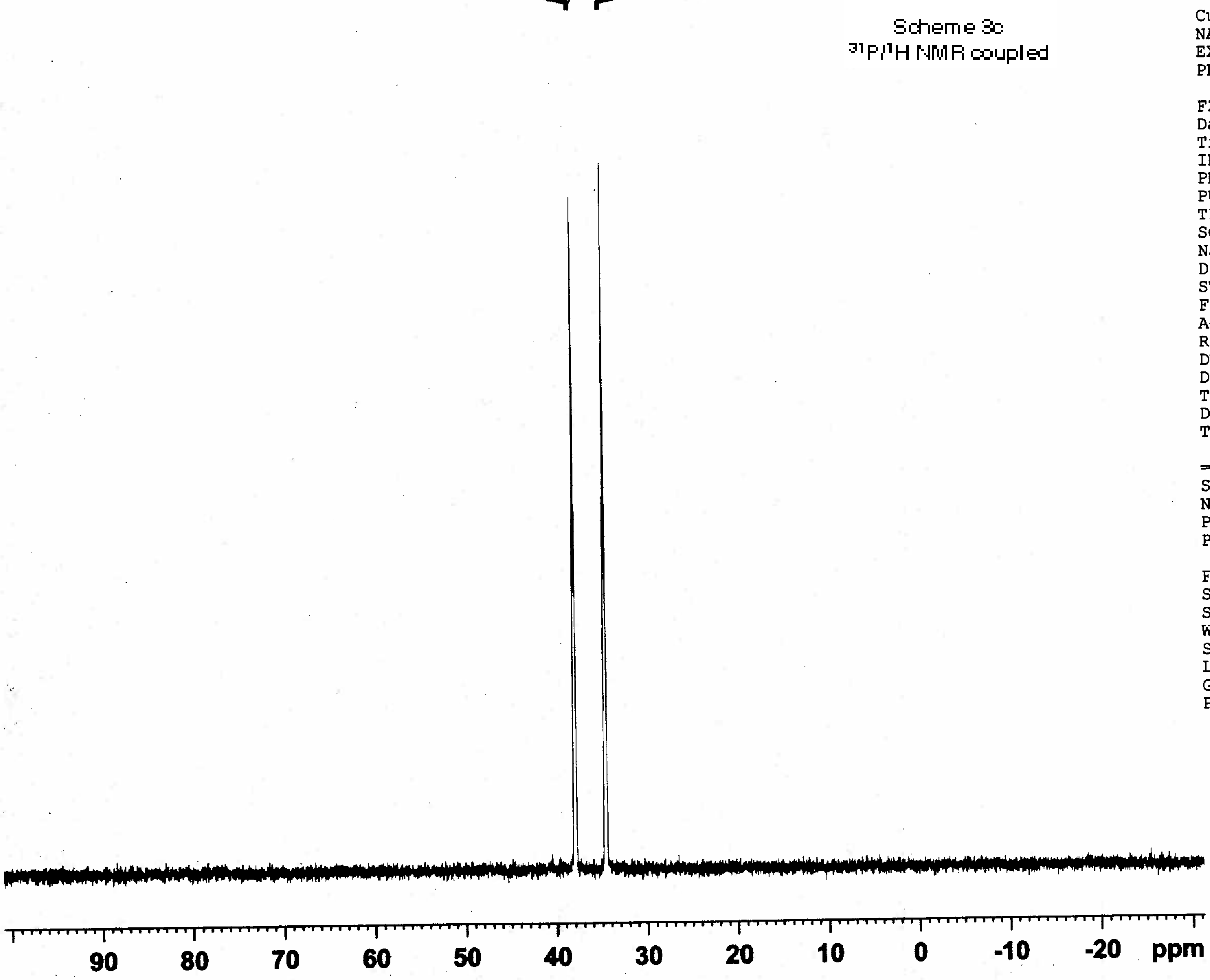

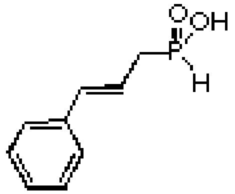

Scheme 3c

$^{31}P/^{1}H$ NMR coupled

BRUKER

Current Data Parameters
NAME OB 1971 after column
EXPNO 2
PROCNO 1

F2 - Acquisition Parameters

Date_	20150325
Time	17.08
INSTRUM	spect
PROBHD	5 mm PABBO BB/
PULPROG	zg30
TD	65536
SOLVENT	CDCl3
NS	32
DS	4
SWH	64102.563 Hz
FIDRES	0.978127 Hz
AQ	0.5111808 sec
RG	203.57
DW	7.800 usec
DE	6.50 usec
TE	293.8 K
D1	2.00000000 sec
TD0	1

======== CHANNEL f1 ========

SFO1	161.9674942 MHz
NUC1	31P
P1	14.25 usec
PLW1	15.00000000 W

F2 - Processing parameters

SI	32768
SF	161.9755930 MHz
WDW	EM
SSB	0
LB	1.00 Hz
GB	0
PC	1.40

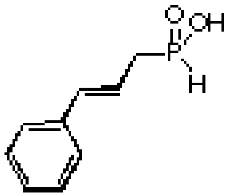

Scheme 3c
^1H NMR

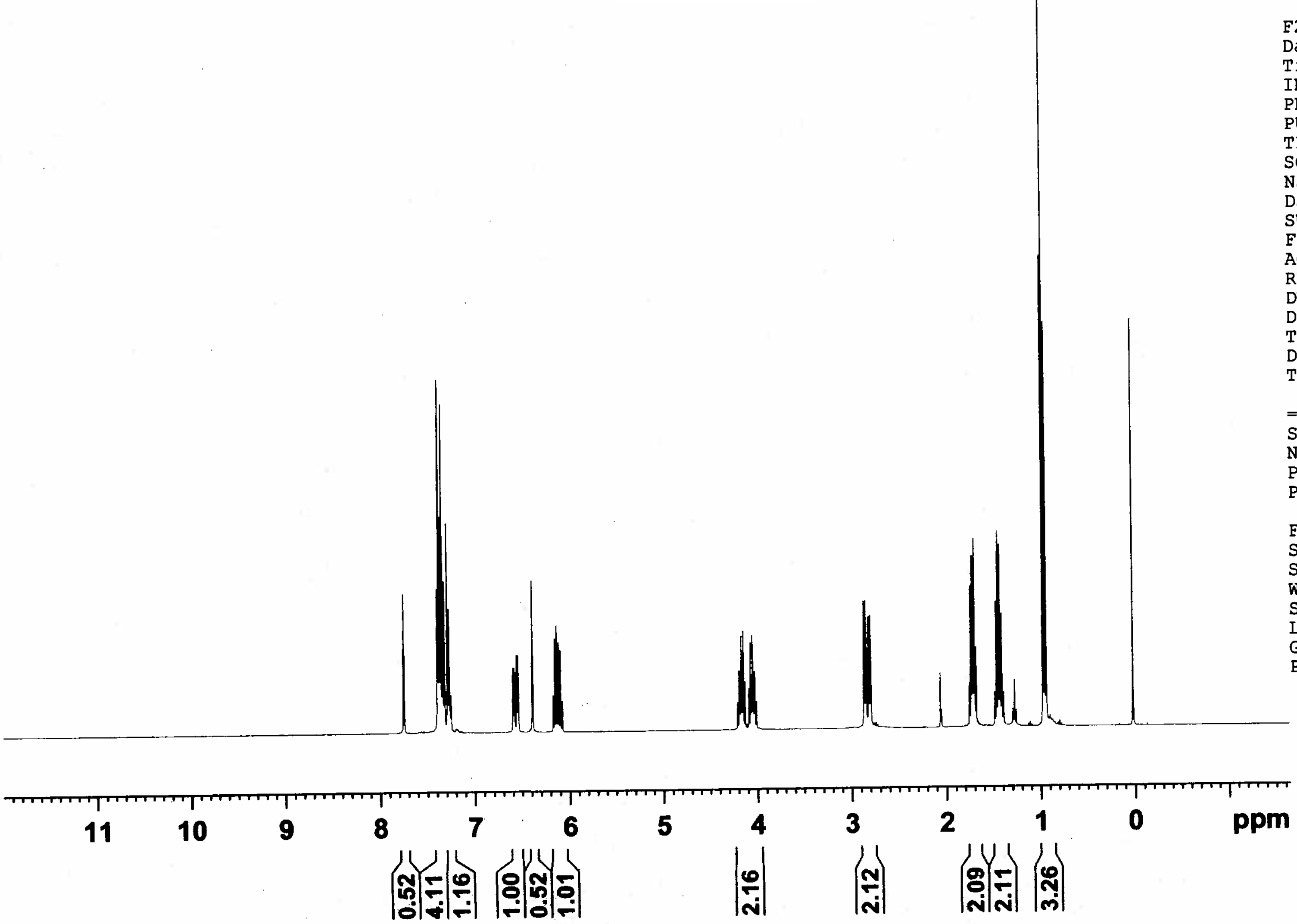

BRUKER

```
Current Data Parameters
NAME      OB 1971 after column
EXPNO                    3
PROCNO                   1

F2 - Acquisition Parameters
Date_             20150325
Time                 17.09
INSTRUM              spect
PROBHD    5 mm PABBO BB/
PULPROG               zg30
TD                   65536
SOLVENT              CDCl3
NS                       9
DS                       2
SWH            8012.820 Hz
FIDRES         0.122266 Hz
AQ           4.0894465 sec
RG                   81.67
DW             62.400 usec
DE               6.50 usec
TE                 293.7 K
D1          1.00000000 sec
TD0                      1

======== CHANNEL f1 ========
SFO1       400.1324710 MHz
NUC1                    1H
P1              10.00 usec
PLW1        25.00300026 W

F2 - Processing parameters
SI                   65536
SF         400.1300000 MHz
WDW                     EM
SSB       0
LB                 0.30 Hz
GB        0
PC                    1.00
```

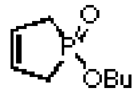

Scheme 3c

$^{31}P/^{1}H$ NMR decoupled

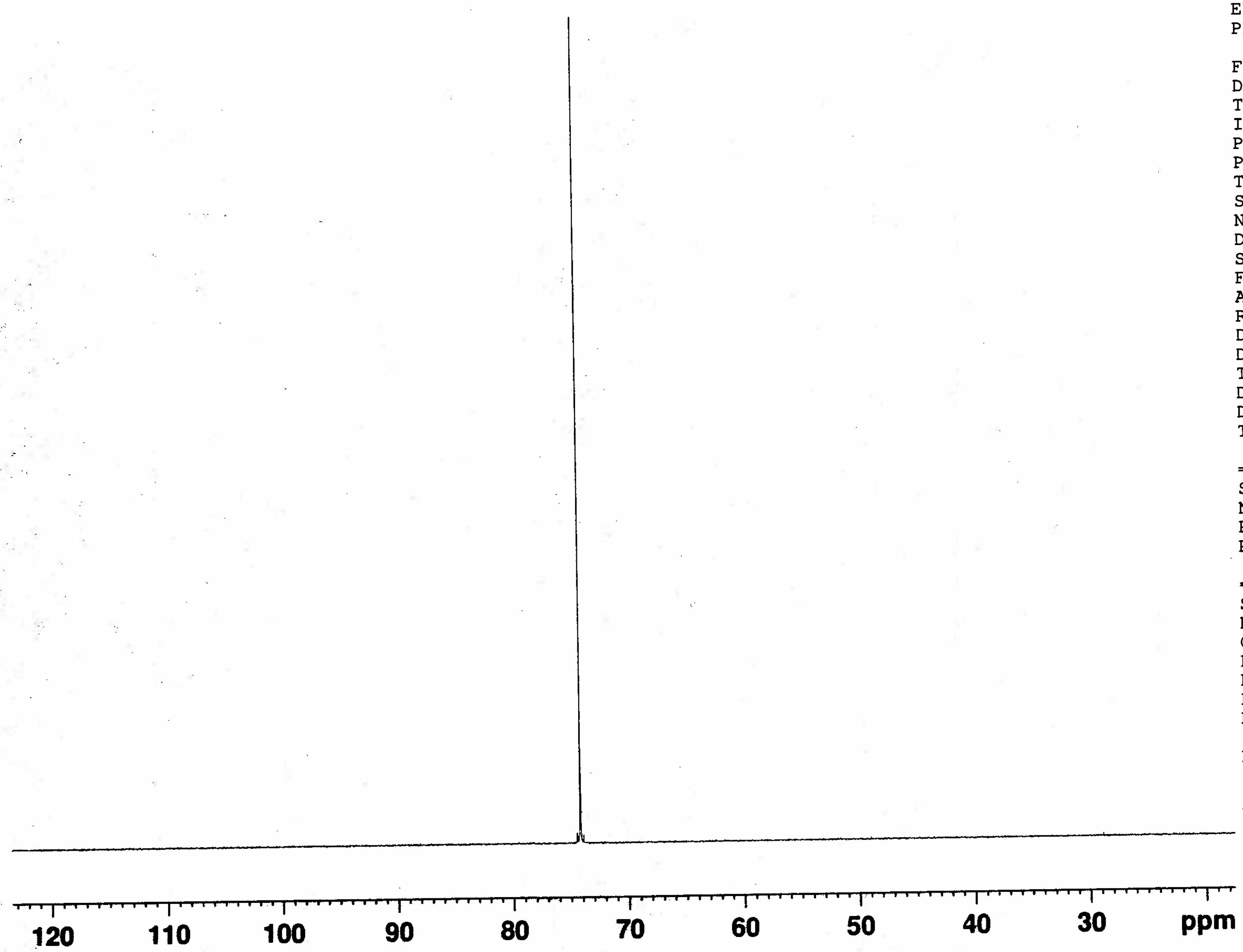

Current Data Parameters

NAME	AFL105
EXPNO	3
PROCNO	1

F2 - Acquisition Parameters

Date_	20150827
Time	13.59
INSTRUM	spect
PROBHD	5 mm PABBO BB/
PULPROG	zgpg30
TD	65536
SOLVENT	CDCl3
NS	8
DS	4
SWH	64102.563 Hz
FIDRES	0.978127 Hz
AQ	0.5111808 sec
RG	203.57
DW	7.800 usec
DE	6.50 usec
TE	294.7 K
D1	2.00000000 sec
D11	0.03000000 sec
TD0	1

======== CHANNEL f1 ========

SFO1	161.9674942 MHz
NUC1	31P
P1	14.25 usec
PLW1	15.00000000 W

======== CHANNEL f2 ========

SFO2	400.1316005 MHz
NUC2	1H
CPDPRG[2	waltz16
PCPD2	90.00 usec
PLW2	10.00000000 W
PLW12	0.31604999 W
PLW13	0.25600001 W

F2 - Processing parameters

SI	32768
SF	161.9755930 MHz
WDW	EM
SSB	0
LB	1.00 Hz
GB	0
PC	1.40

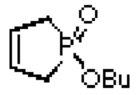

Scheme 3c
$^{31}P/^{1}H$ NMR coupled

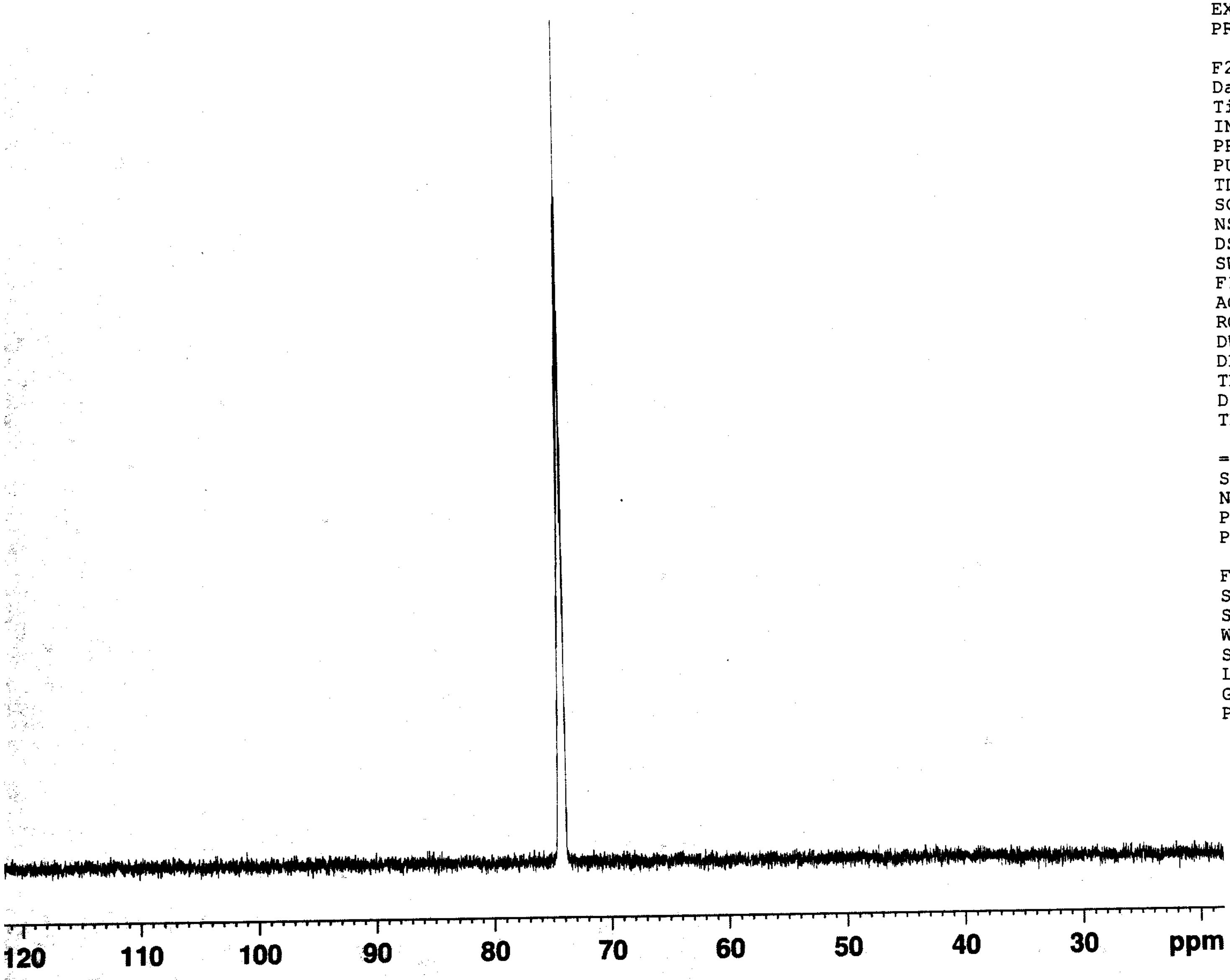

Current Data Parameters
NAME AFL105
EXPNO 4
PROCNO 1

F2 - Acquisition Parameters
Date_ 20150827
Time 14.00
INSTRUM spect
PROBHD 5 mm PABBO BB/
PULPROG zg30
TD 65536
SOLVENT CDCl3
NS 13
DS 4
SWH 64102.563 Hz
FIDRES 0.978127 Hz
AQ 0.5111808 sec
RG 203.57
DW 7.800 usec
DE 6.50 usec
TE 294.4 K
D1 2.00000000 sec
TD0 1

======== CHANNEL f1 ========
SFO1 161.9674942 MHz
NUC1 31P
P1 14.25 usec
PLW1 15.00000000 W

F2 - Processing parameters
SI 32768
SF 161.9755930 MHz
WDW EM
SSB 0
LB 1.00 Hz
GB 0
PC 1.40

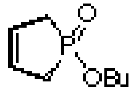

Scheme 3c

^{1}H NMR

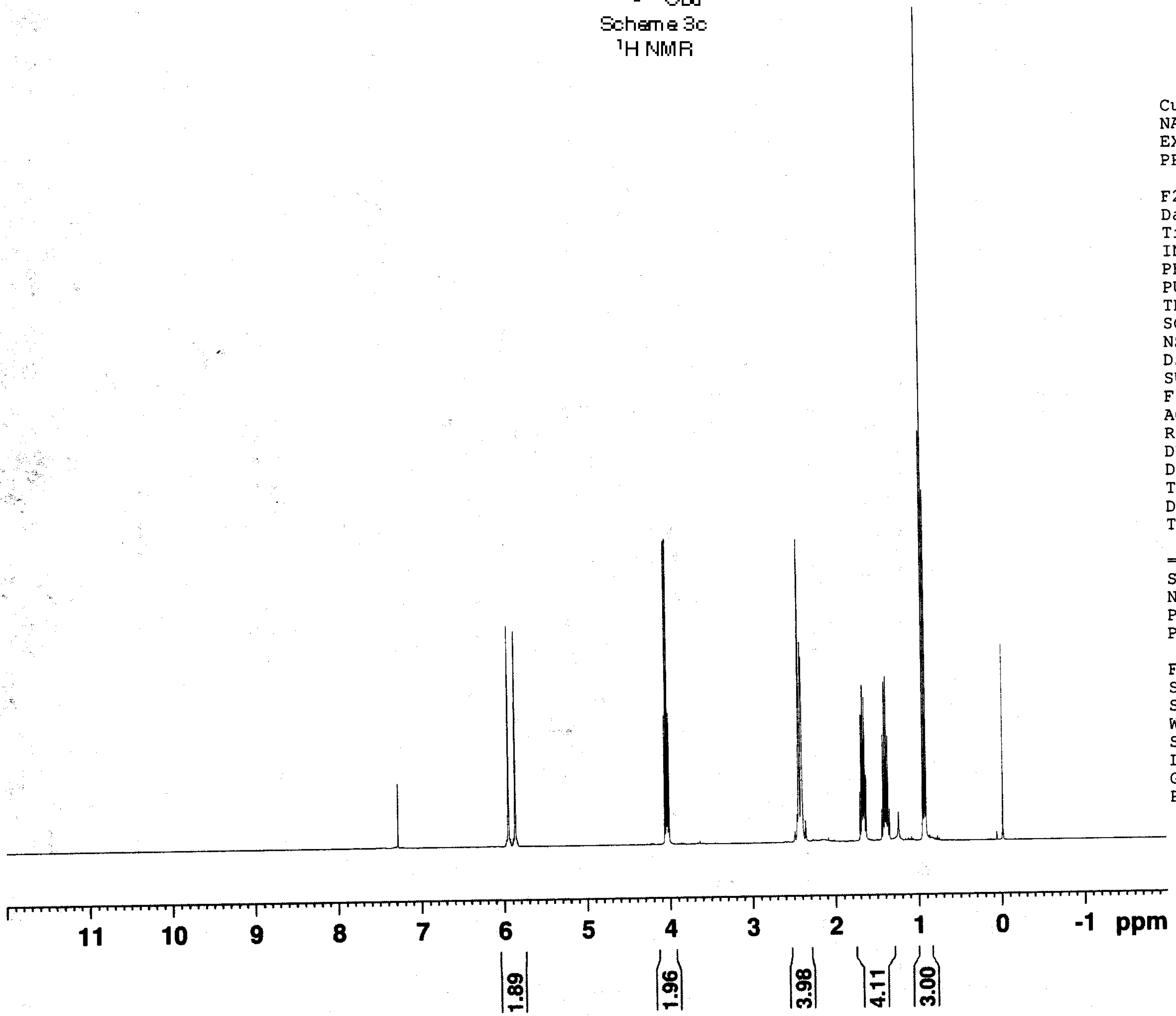

Current Data Parameters
NAME AFL105
EXPNO 5
PROCNO 1

F2 - Acquisition Parameters
Date_ 20150827
Time 14.05
INSTRUM spect
PROBHD 5 mm PABBO BB/
PULPROG zg30
TD 65536
SOLVENT CDCl3
NS 14
DS 2
SWH 8012.820 Hz
FIDRES 0.122266 Hz
AQ 4.0894465 sec
RG 66.4
DW 62.400 usec
DE 6.50 usec
TE 294.4 K
D1 1.00000000 sec
TD0 1

======== CHANNEL f1 ========
SFO1 400.1324710 MHz
NUC1 1H
P1 10.00 usec
PLW1 25.00300026 W

F2 - Processing parameters
SI 65536
SF 400.1300000 MHz
WDW EM
SSB 0
LB 0.30 Hz
GB 0
PC 1.00

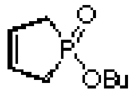

Scheme 3c

^{13}C NMR

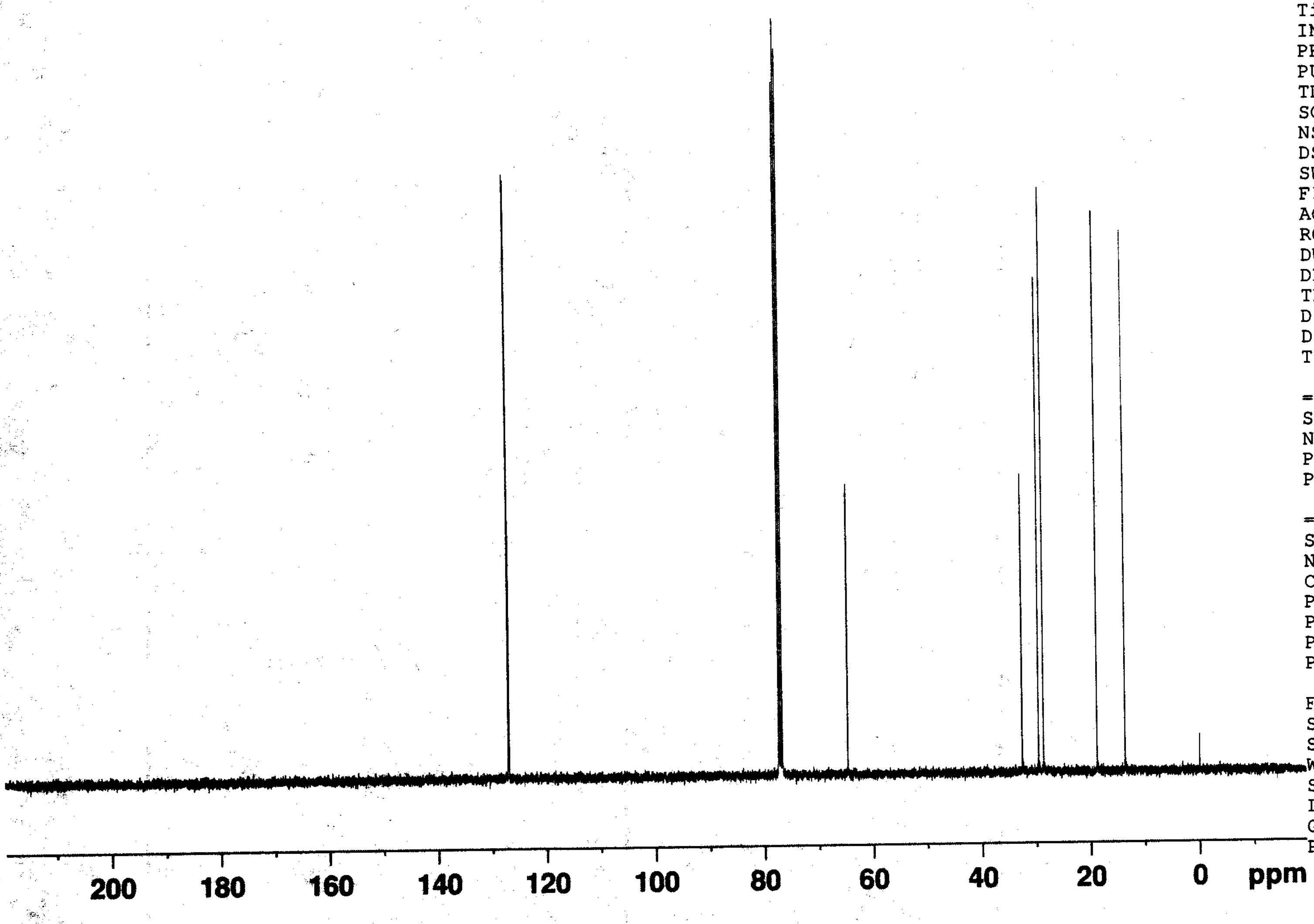

Current Data Parameters
NAME AFL105
EXPNO 6
PROCNO 1

F2 - Acquisition Parameters
Date_ 20150827
Time 16.47
INSTRUM spect
PROBHD 5 mm PABBO BB/
PULPROG zgpg30
TD 65536
SOLVENT CDCl3
NS 316
DS 4
SWH 24038.461 Hz
FIDRES 0.366798 Hz
AQ 1.3631488 sec
RG 203.57
DW 20.800 usec
DE 6.50 usec
TE 295.7 K
D1 2.00000000 sec
D11 0.03000000 sec
TD0 1

======== CHANNEL f1 ========
SFO1 100.6228293 MHz
NUC1 13C
P1 10.00 usec
PLW1 45.00000000 W

======== CHANNEL f2 ========
SFO2 400.1316005 MHz
NUC2 1H
CPDPRG[2 waltz16
PCPD2 90.00 usec
PLW2 10.00000000 W
PLW12 0.31604999 W
PLW13 0.25600001 W

F2 - Processing parameters
SI 32768
SF 100.6127685 MHz
WDW EM
SSB 0
LB 1.00 Hz
GB 0
PC 1.40

```
OB 909
pad=10 run with findz0 before acquisitio
n

exp1  Phosphorus

      SAMPLE                 SPECIAL
date   Feb  1 2013  temp        not used
solvent      cdcl3  gain              20
file /home/TCUuser~ spin              20
/vnmrsys/data/auto~ hst            0.008
_2013.01.20/s_2013~ pw90          18.300
0201_12/data/cdcl3~ alfa          10.000
           _02.fid          FLAGS
   ACQUISITION      il                 n
sw         15797.8  in                 n
at           1.600  dp                 y
np           50552  hs                ny
fb            8800        PROCESSING
bs              64  lb              1.00
d1           1.000  fn          not used
nt              16         DISPLAY
ct              16  sp           -3647.3
   TRANSMITTER      wp           15797.3
tn             P31  rfl           3647.8
sfrq       121.465  rfp                0
tof         7421.1  rp              24.6
tpwr            55  lp            -113.7
pw           9.150           PLOT
    DECOUPLER       wc               250
dn              H1  sc                 0
dof              0  vs                12
dm             yyy  th                 7
dmm              w  ai  cdc  ph
dpwr            35
dmf           6700
```

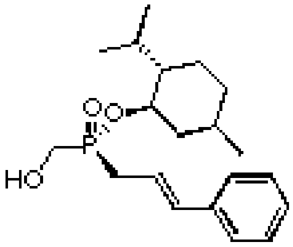

Scheme 4

^{31}P/^{1}H NMR decoupled

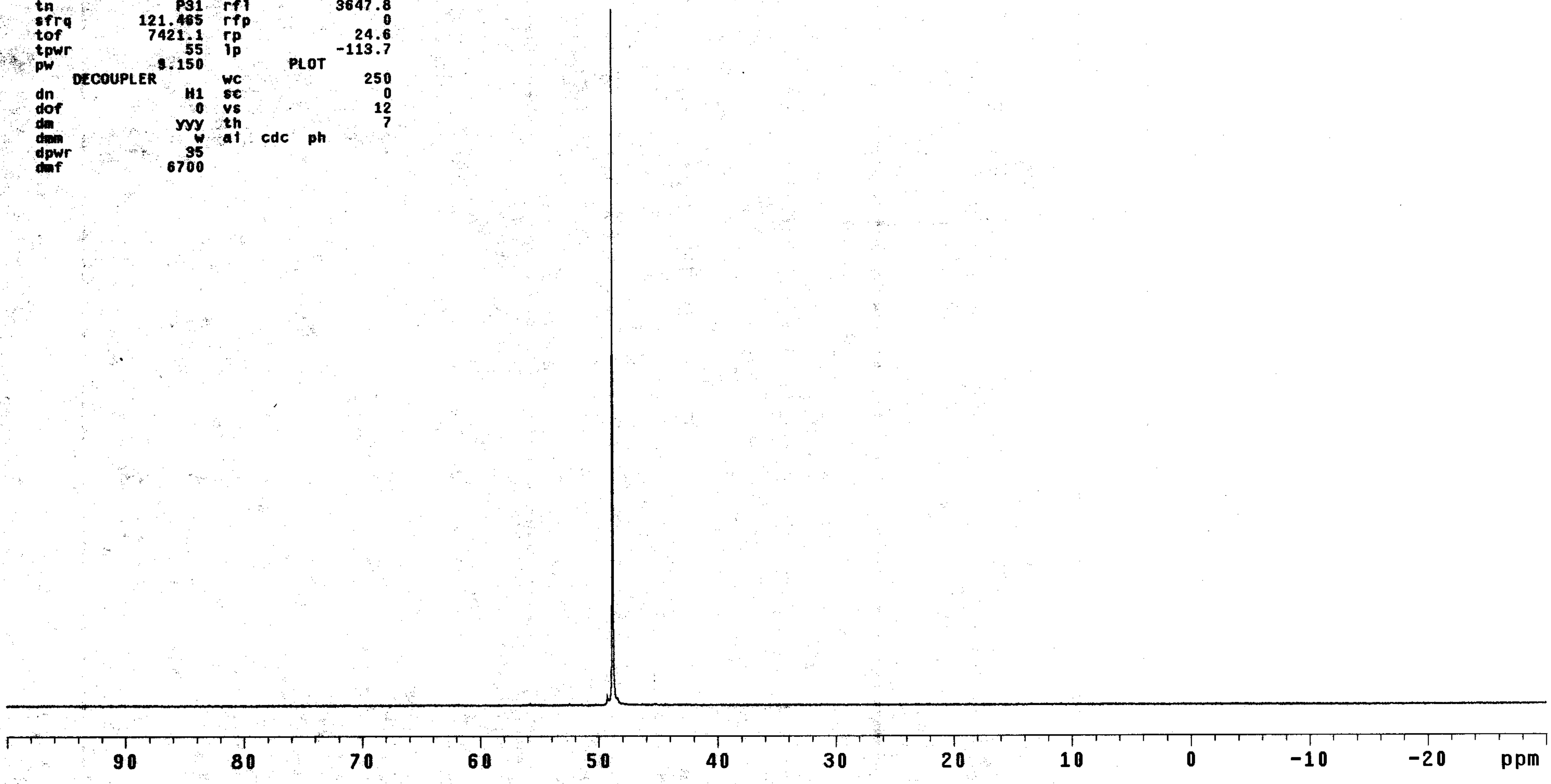

```
OB 900

exp1  Phosphorus

      SAMPLE                SPECIAL
date   Feb  1 2013  temp        not used
solvent      cdcl3  gain             20
file /home/TCUuser~ spin             20
/vnmrsys/data/auto~ hst           0.008
_2013.01.20/s_2013~ pw90         18.300
0201_12/data/cdcl3~ alfa         10.000
            _04.fid          FLAGS
   ACQUISITION      il                n
sw         15797.8  in                n
at           1.600  dp                y
np           50552  hs               ny
fb            8800       PROCESSING
bs              64  lb             1.00
d1           1.000  fn         not used
nt              16          DISPLAY
ct              16  sp          -3647.3
   TRANSMITTER      wp          15797.3
tn             P31  rfl          3647.8
sfrq       121.465  rfp               0
tof         7421.1  rp             15.6
tpwr            55  lp           -113.7
pw           9.150           PLOT
    DECOUPLER       wc              250
dn              H1  sc                0
dof              0  vs               35
dm             ynn  th                3
dmm              w  ai   cdc   ph
dpwr            35
dmf           6700
```

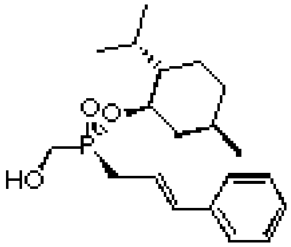

Scheme 4

$^{31}P/^{1}H$ NMR coupled

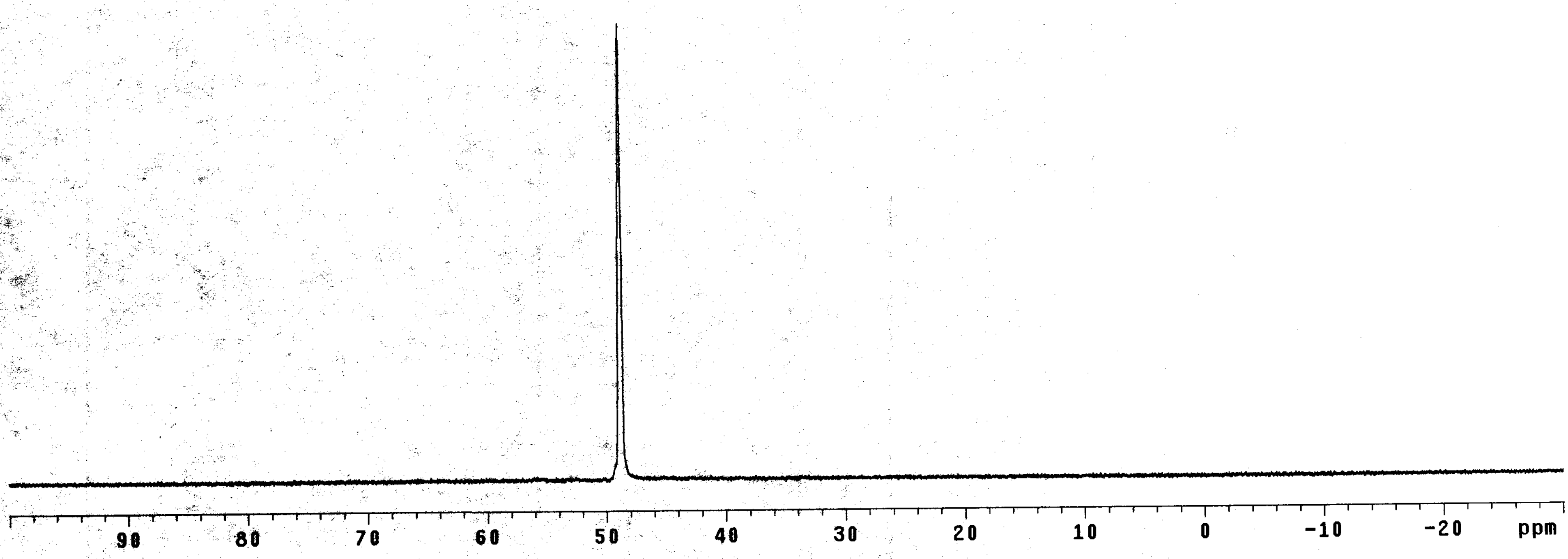

```
OB 900
pad=10 run with findz0 before acquisitio
n
exp1  Proton

     SAMPLE            DEC. & VT
date   Feb  1 2013  dfrq        75.454
solvent      cdcl3  dn             C13
file /home/TCUuser~ dpwr            43
/vnmrsys/data/auto~ dof              0
_2013.01.20/s_2013~ dm             nnn
0201_10/data/cdcl3~ dmm              c
            01.fid  dmf          13100
   ACQUISITION          PROCESSING
sfrq       300.047  wtfile
tn              H1  proc            ft
at           1.998  fn        not used
np           19184
sw          4800.8  werr       xmreact
fb            2600  wexp abortoff flus~
bs              16  h procplot aborton
tpwr            55  wbs
pw             7.9  wnt
d1           1.000
tof          277.9
nt              16
ct              16
alock            y
gain      not used
      FLAGS
il               n
in               n
dp               y
     DISPLAY
sp          -598.3
wp          4800.5
vs             378
sc               0
wc             240
hzmm         20.00
is          563.16
rfl          598.6
rfp              0
th               2
ins          1.000
ai   cdc  ph
```

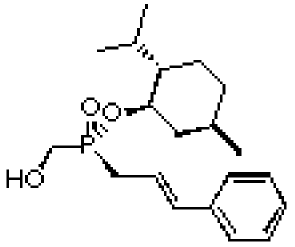

Scheme 4
^{1}H NMR

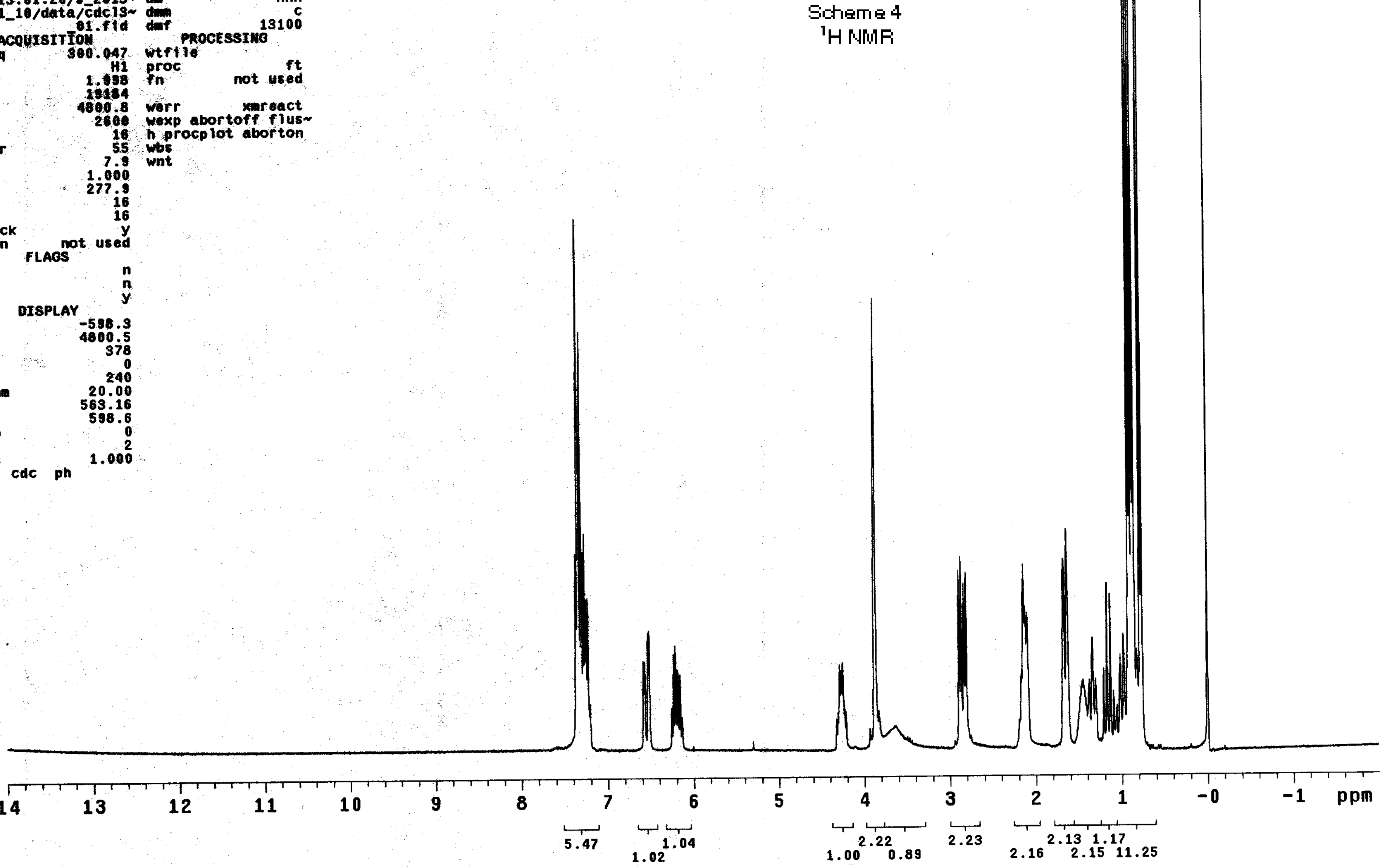

```
OB 900

exp1  Carbon

      SAMPLE                SPECIAL
date   Feb  1 2013  temp       not used
solvent      cdcl3  gain             20
file /home/TCUuser~ spin             20
/vnmrsys/data/auto~ hst           0.008
_2013.01.20/s_2013~ pw90         18.500
0201_12/data/cdcl3~ alfa         10.000
           05.fid           FLAGS
    ACQUISITION     il                n
sw         18115.9  in                n
at           1.301  dp                y
np           47120  hs               nn
fb           10000        PROCESSING
bs              64  lb             0.50
d1           2.000  fn         not used
nt             750         DISPLAY
ct             750  sp          -1135.5
    TRANSMITTER     wp          18115.4
tn             C13  rfl          1136.1
sfrq        75.454  rfp               0
tof          766.0  rp              9.2
tpwr            58  lp           -182.4
pw           9.250           PLOT
     DECOUPLER      wc              250
dn              H1  sc                0
dof              0  vs              332
dm             yyy  th                6
dmm              w  ai   cdc  ph
dpwr            35
dmf           6700
```

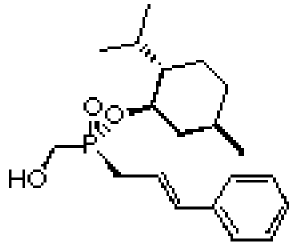

Scheme 4
^{13}C NMR

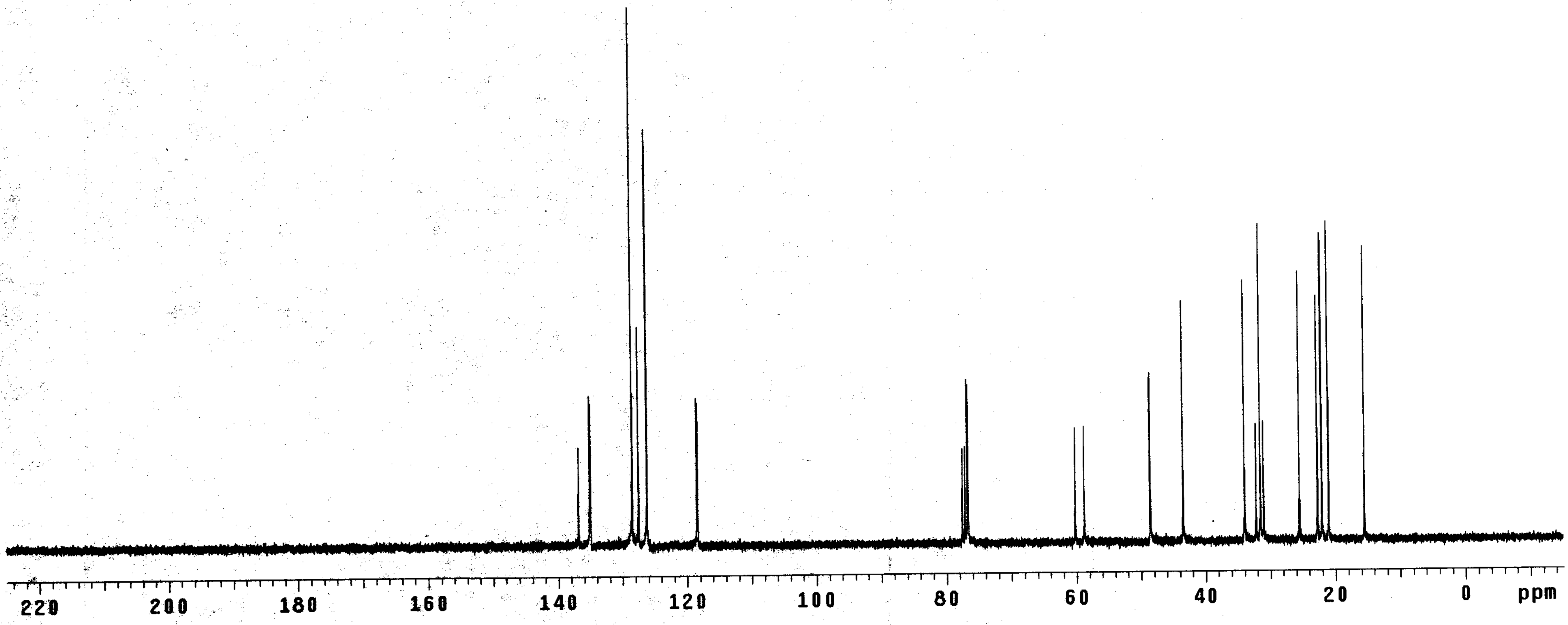

Supplement: Supplementary file 1 [file molecules-21-01295-s001.pdf]
